# Genome-wide detection of terpene synthase genes in holy basil (*Ocimum sanctum* L.)

**DOI:** 10.1371/journal.pone.0207097

**Published:** 2018-11-16

**Authors:** Yogesh Kumar, Feroz Khan, Shubhra Rastogi, Ajit Kumar Shasany

**Affiliations:** 1 Metabolic and Structural Biology Dept, CSIR-Central Institute of Medicinal and Aromatic Plants, Lucknow (U.P.), INDIA; 2 Biotechnology Division, CSIR-Central Institute of Medicinal and Aromatic Plants, Lucknow (U.P.), INDIA; National Cheng Kung University, TAIWAN

## Abstract

Holy basil (*Ocimum sanctum L*.) and sweet basil (*Ocimum basilicum L*.) are the most commonly grown basil species in India for essential oil production and biosynthesis of potentially volatile and non-volatile phytomolecules with commercial significance. The aroma, flavor and pharmaceutical value of *Ocimum* species is a significance of its essential oil, which contains most of the monoterpenes and sesquiterpenes. A large number of plants have been studied for characterization and identification of terpene synthase genes, involved in terpenoids biosynthesis. The goal of this study is to discover and identify the putative functional terpene synthase genes in *O*. *sanctum*. HMMER search was performed by using a set of 13 well sequenced and annotated plant genomes including the newly sequenced genome of *O*. *sanctum* with Pfam-A database locally, using HMMER 3.0 hmmsearch for the two Pfam domains (PF01397 and PF03936). Using this search method 81 putative terpene synthases genes (*OsaTPS*) were identified in *O*. *sanctum*; the study further reveals 47 *OsaTPS* were putatively functional genes, 19 partial *OsaTPS*, and 15 *OsaTPS* as probably pseudogenes. All these identified *OsaTPS* genes were compared with other plant species, and phylogenetic analysis reveals the subfamily classification of *OsaTPS* in TPS-a, -b, -c, -e, -f and TPS-g subfamilies clusters. This genome-wide identification of *OsaTPS* genes, their phylogenetic analysis and secondary metabolite pathway mapping predictions together provide a comprehensive understanding of the TPS gene family in *Ocimum sanctum* and offer opportunities for the characterization and functional validation of numbers of terpene synthase genes.

## Introduction

The terpenes are a large class of plant specialized secondary metabolites, which derived from 5 carbon (C_5_) isoprenoid unit; these precursors are produced by two biosynthetic pathways, the methylerythritol phosphate pathway (MEP) in the chloroplast and the classical mevalonate pathway (MVA). MVA is also known as the isoprenoid pathway or HMG-CoA reductase pathway present in the cytosol [1,2], the pathway produces five-carbon building blocks of terpenoids called isopentenyl pyrophosphate (IPP) and dimethylallyl pyrophosphate (DMAPP) [3,4], they are used to make isoprenoids. IPP and DMAPP combined by prenyltransferases and known as isoprenyl diphosphate synthases [5]. The condensation reaction by prenyltransferases produces geranyl diphosphate (GPP C_10_), farnesyl diphosphate (FPP C_15_) in the cytosol and geranylgeranyl pyrophosphate (GGPP C_20_) in the chloroplast. These all prenyl pyrophosphates work as a substrate for the terpene synthases, which further performed the catalysis for the production of different terpenoids: hemiterpene (C_5_) is produced directly by isopentyl pyrophosphate, monoterpene (C_10_) from GPP, sesquiterpene (C_15_) from FPP, diterpene (C_20_) form GGPP, and other terpenes like sesterterpenes (C_25_), triterpenes (C_30_), sesquarterpenes (C_35_), tetraterpenes (C_40_) and polyterpene (>C_40_) [6]. These terpenes are responsible for plant defense against biotic and abiotic stresses, used to attract insects for pollination, and used by human for their beneficial purposes as a natural flavor, for perfumery, and for cosmetics. These terpenoids are synthesized by specialized genes know as terpene synthases.

The terpene synthase genes are classified into different classes. According to their genomic structures (including intron and exon numbers), terpene synthase sequences (TPSs) can be classified into three classes Class I, Class II and Class III terpene synthases. The class I terpene synthase genes contain 12–14 introns, and 13–15 exons, class II genes possess nine introns and ten exons. Moreover, class III terpene synthase genes have six introns and seven exons [7–9].

**Fig 1:**
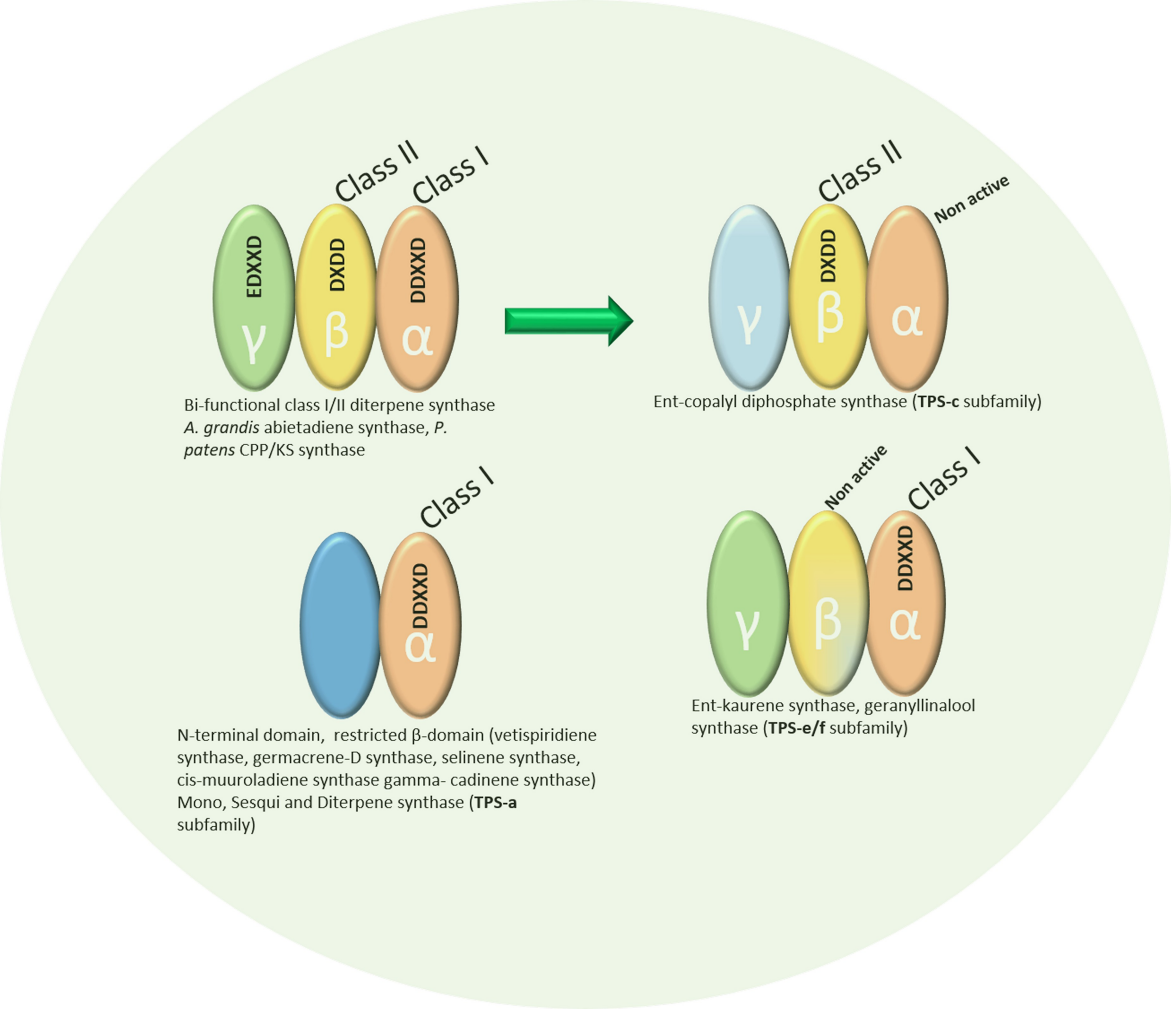
Representation of class I and class II domain within terpene synthase protein structure; class I consists of conserved DDXXD motif in its α-domain, whereas class II contains DXDD conserved motif.

Based on catalytic mechanism and product formed, TPSs genes are also classified into two classes: class I and class II. The class I and II TPSs have unique conserved amino acid motifs which are essential for catalysis [10,11]. Class I TPSs has a C-terminal domain (also referred to as α-domain or class I fold) which catalyzes the ionization of substrate (*i*.*e*., prenyl diphosphate) mediated by a divalent cation. This metal-dependent ionization of substrate can further lead to cyclizations, hydride shifts, and structural rearrangements to produce the end product. The α-domain present in this class TPSs adopt the α-helical protein fold and holds two metal binding motifs ‘DDXXD’ a highly conserved and less conserved ‘NSE/DTE’ positioned on opposing helices near the entrance of the active site. The class II terpene synthases comprise a functional N-terminal (β-domain), with a third “insertion” γ-domain forming a vestigial class II fold. The enzymes of this class contain conserved functional ‘DXDD’ motif which resides in a separate β-domain and accountable for the protonation-initiated cyclization of the substrate [11,12]. The γ-domain carries a highly acidic EDXXD like motif, which contributes to the activity of class II TPSs (Fig 1) [13]. Many terpene synthases also have a highly conserved RR(x)8W motif downstream of the N-terminal transit peptide while this motif does not require in monoterpene synthase activity.

According to recent subfamilies classification, TPS genes are classified into eight subfamilies: TPS-a to TPS-h based on sequence properties and functional characteristics. Class I TPSs family contains TPS-a, -b, -e/f, and TPS-g [10], where class II contains TPS-c only which comprises genes for copalyl diphosphate related diterpene synthases. The TPS-d family mostly consists of bifunctional enzymes that are capable of the protonation-initiated cyclization, but also catalyze class I catalysis and mostly occur in gymnosperms. Whereas TPS-h is specific to the spikemoss *Selaginella moellendorffii* [10].

The TPS subfamily clades have been studied and recognized through sequencing and functional studies in a wide range of plants *i*.*e*., *Arabidopsis thaliana* containing (32 functional and 8 pseudo genes) [14], *Vitis vinifera* (69 putative functional and 63 pseudo genes) [15,16], *Solanum lycopersicum* (29 functional or potential functional genes and 15 mutated genes) [17], *Oryza sativa* (40 putative functional TPS genes) [18], *Selaginella moellendorfii* (18 TPS genes) [19], *Cajanus cajan* [20] and *Populus trichocarpa* (38 putative functional TPS genes) [21], *Eucalyptus grandis* (113 putative functional gene) *Eucalyptus globulus* (106 putative functional genes, 37 putative pseudogenes) [7]. Genome-wide analysis of terpene synthase genes family in different plants shows that all the TPS gene family is the mid-size with the number ranging from approximately 20 to 150 in sequenced plant genomes, but with only one exception of the moss *Physcomitrella patens*, which possess single functional TPS genes shown in Table 1.

**Table 1:** Terpene synthase genes size in different plant species, including *O*. *sanctum* genome.

Plant species	Putative TPS genes identified by updated TPS HMM model^1^	Putative full-length TPS genes^2^	TPS genes included in *OsaTPS* phylogenetic tree	TPS Subfamily^3^					
				a	b	c	e/f	g	h
*A*. *thaliana*	40	32^$^	33	23	6	1	2	1	0
*V*. *vinifera*	91	69^$^	55	29	9	2	1	14	0
*O*. *sativa*	32	32^$^	32	19	0	3	9	1	0
*P*. *patens*	2	2^$^	0	0	0	2	0	0	0
*E*. *grandis*	120	113^$^	113	52	36	2	10	13	0
*E*. *globulus*	106	106^$^	69	32	30	2	2	3	0
*S*. *lycopersicum*	41	29^$^	29	12	8	2	5	2	0
*S*. *bicolor*	41	24^$^	24	15	2	1	3	3	0
*P*. *trichocarpa*	77	32^$^	31	13	11	2	3	2	0
*N*. *tabaccum*	96	14^@^	2	0	0	1	1	0	0
*C*. *cajan*	52	-	0	29	9	2	10	2	0
*S*. *moellendroffii*	40	14^$^	0	0	0	3	3	0	8
*O*. *basilicum*	-	2^$^	2	0	0	0	0	2	0
*O*. *sanctum*	47	-	47	18	16	5	3	5	0

^1^Total number of terpene synthase genes identified with the TPS HMM sequence models.

^2^Putative full-length genes retrieved from available data.

^3^PutativeTPS genes classified into subfamily by phylogenetic analysis.

^$^Numbers of TPS genes retrieved from the information based on literature data.

^@^TPS sequence data retrieved from the non-redundant protein sequence database from NCBI

*Ocimum sanctum* (Holy basil) due to its medicinal properties has great importance in Indian traditional medicine and Ayurveda. It is a sweet-scented, pubescent herb nearly 3 to 100cm in height, growing abundantly in tropical, sub-tropical and warm temperate regions. *Ocimum sanctum* plays a significant role in herbal as well as modern medicines. Many studies have focused on the medicinal prospect of this plant in the form of crude drugs, essential oil or pure compounds. *O*. *sanctum* shows antidiabetic, antimicrobial, antioxidant, anti-inflammatory, antinociceptive, antifertility, anticancer, anthelmintic and cardioprotective properties [22,23]. Several *Ocimum* species produce essential oil with methyl eugenol being a major constituent and reported to have anti-cancer properties [24,25]. Additionally, *Ocimum* terpenoids also used as biodegradable herbicide due to their phytotoxic activity [26]. The Lamiaceae family is the sixth most extensive family of the flowering plants and plays a significant role in the economy of natural products and used in foods, pharmaceuticals, flavor and perfumery industries. This family shows a remarkably high degree of secondary metabolite diversity specially terpenoids [27]. Because of their importance a number of *TPSs* genes which were responsible for terpenoids biosynthesis have been explored and characterized in many species of Lamiaceae family, these species were closely related to *Ocimum sanctum*, including *Rosmarinus officinalis*, *Mentha piperita* [28–30], *Mentha cablin* [31,32], *Mentha aquatic* [33], *Salvia miltiorrhiza* [34–36], *Isodon eriocalyx* [37,38], *Coleus forskohlii* [39], *Salvia fruticose* [40], *Salvia pomifera* [41], *Salvia sclarea* [42,43] *and Salvia divimorum* [44]. Although in prior studies at least 44 monoterpenes and 39 sesquiterpenes were reported in literature reports [27,45].

The volatile constituents of various *Ocimum sanctum* tissues such as leaves, flowers, and seeds have been previously examined, and constitute mostly monoterpenes, sesquiterpenes and diterpenes [46], but least number of Terpene synthases genes were characterized in *Ocimum* species. Some characterized *TPSs* genes of *Ocimum* are (Geraniol synthase [47], R-linalool synthase, (-)-Endo-fenchol synthase, Selinene synthase, Terpinolene synthase, β-myrcene synthase, α-Zingiberene synthase, γ-Cadinene synthase, and Germacrene-D synthase, all monoterpene and sesquiterpene synthases) [48,49] and one triterpene oxidosqualene synthases [50]. Due to unavailability of whole genome sequencing data, an actual number of TPS genes in *Ocimum sanctum* is still unknown. In this study, we have identified 47 putative functional terpene synthase genes in *O*. *sanctum* and classified the *TPSs* into subfamily. The comparative sequence study with closely related species reveals and suggest the possible biological role of these predicted *O*. *sanctum* terpene synthases. These results may provide insight for further characterizing the putatively functional terpene synthase genes in the Holy basil.

## Materials and methods

### Data retrieval and identification of TPSs in *O*. *sanctum*

The 13 plant's genome and proteome data were downloaded from (https://www.ncbi.nlm.nih.gov/genome/) and (https://www.ncbi.nlm.nih.gov/assembly/). The genome data of *Ocimum sanctum* was taken from the in-house sequenced genome repository. Two terpene synthase specific domains Pfam N-terminal (PF01397) (https://pfam.xfam.org/family/PF01397#tabview=tab6) and Pfam C-terminal domains (PF03936) (https://pfam.xfam.org/family/PF03936#tabview=tab6) were downloaded from the Pfam database [51]. Standalone tool, HMMER version 3.1b2 was downloaded [52] and used to search the *Ocimum sanctum* predicted proteome data including 13 downloaded proteomes using the PF03936 and PF01397 domains model data as a query for proteome; the significant (e-value <10^−3^) was set for identification of candidate TPS genes. The candidate genes were also inspected with a ScanProsite tool (https://prosite.expasy.org/scanprosite/) for terpene synthase motifs identification. FGENESH gene prediction tool (www.softberry.com) was also used for initial annotation of the predicted *Ocimum sanctum* terpene synthase (*OsaTPS)* genes. Predicted *OsaTPS* were then monitored for the full-length gene, to make them full-length the upstream and downstream areas of the conserved scaffold region were screened, and reverse BLASTx search was used to confirm the identity of the putative functional TPS gene. Out of 90 identified *OsaTPS* genes, 9 were excluded due to short sequence or stop codons in the translated gene, among the 81 selected *OsaTPS* genes, only 47 were taken for further analysis, rest 19 partial *OsaTPS* and 15 *OsaTPS* probably pseudogenes were retained separately for study shown in S1 and S2 Tables. Some of the genes, which do not cover the full-length gene sequence were also included in this study. The Gene structure intron-exon and motif representation of putative *OsaTPS* genes were determined by the Gene structure display server 2.0 version [53].

### Phylogenetic analysis of terpene synthase genes from *O*. *sanctum* and other plants

The terpene synthase genes from different plants were retrieved, i.e., *Arabidopsis thaliana* (http://arabidopsis.org/), *Oryza sativa* (http://www.plantgdb.org/OsGDB/) [54], *Vitis vinifera* (http://www.plantgdb.org/VvGDB), *Solanum lycopersicum* Potato Genome Sequencing Consortium [55], *Populus trichocarpa* (http://www.plantgdb.org/PtGDB) [56], *Sorghum bicolor* (http://www.plantgdb.org/SbGDB/) [57], *Ocimum basilicum* (http://www.uniprot.org), *Nicotiana tabaccum* (http://www.uniprot.org), *Eucalyptus grandis* [58] and *Eucalyptus globulus* [59] as shown in Table 1 and TPS sequences shown in S1 File, and further classified into *TPS* subfamily as depicted in S3 Table. Predicted 47 *OsaTPS* genes were included for protein alignment, which was prepared using MUSCLE [60] integrated into MEGA version 6 [61] with standard parameters. The multiple sequence alignment was then manually adjusted to concentrate on various diagnostic conserved motifs of terpene synthases, *i*.*e*., RDR, RR(X)_8_W, DDXXD, DXDD and NSE/ DTE. The neighbor-joining method with 1,000 number of bootstrap replications was used for phylogenetic tree reconstruction; substitution model was checked for best phylogenetic tree construction, the p-distance model was considered with pairwise deletion gaps/missing data treatment. The phylogenetic tree was visualized by Figtree 1.4.2 [62].

### *OsaTPS* sequence annotation and secondary metabolite pathway genes prediction

The Blast search of *OsaTPS* genes was performed against UniProt database, and GO (Gene Ontology) terms were assigned for each unigene based on the GO term annotated to its corresponding homolog in the UniProt database. The protein sequences of *Arabidopsis thaliana*, *Solanum lycopersicum*, *Ocimum basilicum*, *Salvia officinalis*, *Ricinus communis*, *Lavandula angustifolia*, *Pisum sativum*, *Nicotiana tabacum*, *Mentha x piperita*, *Antirrhinum majus*, *Fragaria x ananassa*, *Phyla dulcis*, *Solanum tuberosum*, *Pogostemon cablin*, *Origanum vulgare*, *Hyoscymus muticus*,*and Populus tremuloides* were used for sequence alignment as shown in S4 Table. The predicted *OsaTPS* genes were mapped for annotation and prediction of putative functional genes involved in the biosynthesis of secondary metabolites against KEGG metabolic pathways using BlastKOALA (KEGG Orthology And Links Annotation), which is freely available at (http://www.kegg.jp/blastkoala/) [51].

## Results

### *In-silico* identification of putative terpene synthase genes in *O*. *sanctum*

In the study 81, putative TPS genes in *Ocimum sanctum* genome were identified using a high sequence similarity screening search for HMM PF01397 and PF03936 TPS domain models including 13 other plant species genomes. Out of 81 putative *OsaTPS* genes, 19 *OsaTPS* were predicted as partial genes and 15 probably pseudogenes. The *OsaTPS* genes which are partial and having multiple frameshifts or stop codons were not considered in this study. The 34 partial and pseudogenes separately analyzed for terpene synthase subfamily classification, where out of these, 15 partial genes falls in TPS-a, and 3 in TPS-b subfamily group and 1 partial gene falls in TPS-e subfamily, rest 8 pseudogenes genes fall in TPS-a subfamily, 3 in TPS-b, 2 in TPS-c, 1 in TPS-e and 1 gene in TPS-f subfamily group. All the partial and pseudogenes were retained for further study as shown in S2 Table. Maximum pseudogenes were found among the TPS-a subfamily, after all, manual curation and sequence filtration, only 47 putative functional *OsaTPS* genes were considered for further analysis Table 2.

**Table 2:** Sequence annotation data of predicted genes from *Ocimum sanctum*.

S. No	Gene code	TPS subfamily	Protein length	Gene ID (gi)	Organism
1	*OsaTPS*1	TPSa	507	75251482	*Ocimum basilicum*
2	*OsaTPS*2	TPSa	535	75251483	*Ocimum basilicum*
3	*OsaTPS*3	TPSa	320	939319666	*Solanum lycopersicum*
4	*OsaTPS*4	TPSa	577	75267592	*Solanum tuberosum*
5	*OsaTPS*5	TPSa	566	75251484	*Ocimum basilicum*
6	*OsaTPS*6	TPSa	383	403399735	*Origanum vulgare*
7	*OsaTPS*7	TPSa	545	122219293	*Pogostemon cablin*
8	*OsaTPS*8	TPSa	550	122219293	*Pogostemon cablin*
9	*OsaTPS*9	TPSa	543	122219293	*Pogostemon cablin*
10	*OsaTPS*10	TPSa	540	476007192	*Phyla dulcis*
11	*OsaTPS*11	TPSa	521	75251482	*Ocimum basilicum*
12	*OsaTPS*12	TPSa	551	75251484	*Ocimum basilicum*
13	*OsaTPS*13	TPSa	468	476007821	*Hyoscyamus muticus*
14	*OsaTPS*14	TPSa	383	75252096	*Mentha x piperita*
15	*OsaTPS*15	TPSa	274	122219293	*Pogostemon cablin*
16	*OsaTPS*16	TPSa	379	75252096	*Mentha x piperita*
17	*OsaTPS*17	TPSa	507	75251484	*Ocimum basilicum*
18	*OsaTPS*18	TPSa	388	75251482	*Ocimum basilicum*
19	*OsaTPS*19	TPSb	533	75232269	*Populus tremuloides*
20	*OsaTPS*20	TPSb	530	487524480	*Ricinus communis*
21	*OsaTPS*21	TPSb	316	487524480	*Ricinus communis*
22	*OsaTPS*22	TPSb	546	476007226	*Phyla dulcis*
23	*OsaTPS*23	TPSb	512	75251481	*Ocimum basilicum*
24	*OsaTPS*24	TPSb	558	122210944	*Lavandula angustifolia*
25	*OsaTPS*25	TPSb	356	62900763	*Salvia officinalis*
26	*OsaTPS*26	TPSb	524	122210944	*Lavandula angustifolia*
27	*OsaTPS*27	TPSb	589	62900763	*Salvia officinalis*
28	*OsaTPS*28	TPSb	575	62900763	*Salvia officinalis*
29	*OsaTPS*29	TPSb	622	75251478	*Ocimum basilicum*
30	*OsaTPS*30	TPSb	650	75251478	*Ocimum basilicum*
31	*OsaTPS*31	TPSb	350	75251477	*Ocimum basilicum*
32	*OsaTPS*32	TPSb	512	75251477	*Ocimum basilicum*
33	*OsaTPS*33	TPSb	601	75251479	*Ocimum basilicum*
34	*OsaTPS*34	TPSb	556	75251479	*Ocimum basilicum*
35	*OsaTPS*35	TPSc	826	62900382	*Pisum sativum*
36	*OsaTPS*36	TPSc	783	62900382	*Pisum sativum*
37	*OsaTPS*37	TPSc	824	544602148	*Nicotiana tabacum*
38	*OsaTPS*38	TPSc	826	544602148	*Nicotiana tabacum*
39	*OsaTPS*39	TPSc	821	544602148	*Nicotiana tabacum*
40	*OsaTPS*40	TPSe	812	62900385	*Cucurbita maxima*
41	*OsaTPS*41	TPSe	642	544602165	*Nicotiana tabacum*
42	*OsaTPS*42	TPSf	829	75163668	*Arabidopsis thaliana*
43	*OsaTPS*43	TPSg	426	75242446	*Antirrhinum majus*
44	*OsaTPS*44	TPSg	529	332319699	*Fragaria x ananassa*
45	*OsaTPS*45	TPSg	560	75224312	*Ocimum basilicum*
46	*OsaTPS*46	TPSg	467	75224312	*Ocimum basilicum*
47	*OsaTPS*47	TPSg	395	75251480	*Ocimum basilicum*

### Intron-exon structure and organization of *OsaTPS* genes

**Fig 2:**
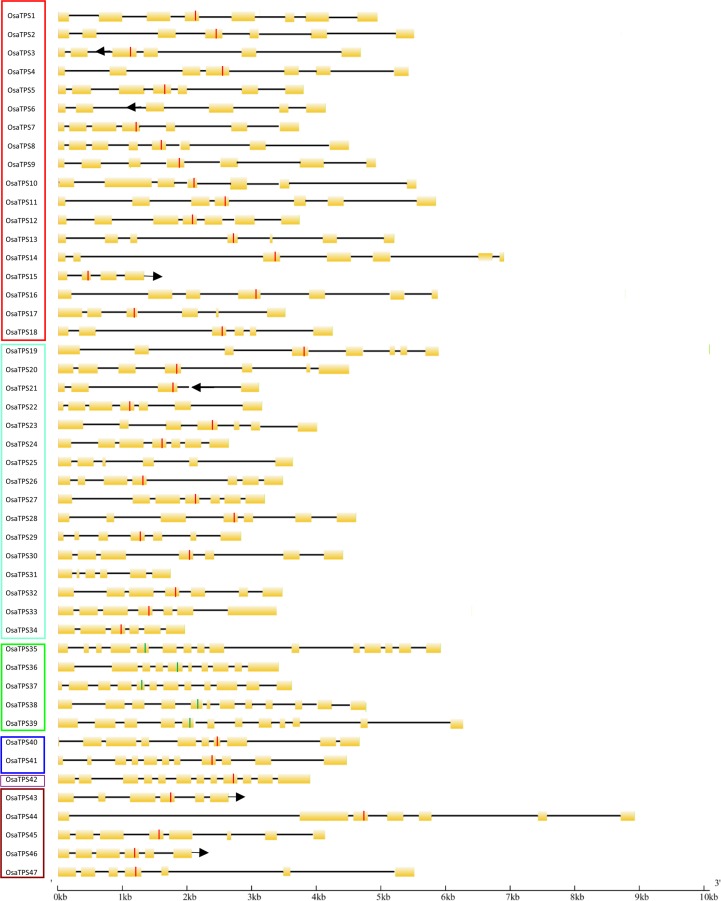
Representation of the gene structure of putative functional TPS genes from *Ocimum sanctum*. Genes were predicted by FGENESH tool and Augustus but also determined manually by mapping to previously TPS characterized genes. The black line indicates the introns; yellow rectangles indicate exons, red lines show conserved DDXXD motif and the green line shows DxDD motif. Arrow indicated in the figure shows the incomplete gene sequence or loss of sequence. Color boxes indicate the TPS subfamily TPS-a (from *OsaTPS*1 to *OsaTPS*19), TPS-b from (*OsaTPS*-19 to *OsaTPS*34), TPS-c from (*OsaTPS*35 to *OsaTPS*39), TPS-e (*OsaTPS*-40) and *OsaTPS*41, TPS-f (*OsaTPS*42) and TPS-g from (*OsaTPS*43 to *OsaTPS*47).

In this study, two forms of TPS gene classification was represented, one according to the presence of number of introns and exons in the TPS genes and another according to the presence of conserved motif. The intron-exon classification shows three classes, class I, class II and class III terpene synthases [59,63]. The *OsaTPS* genes of the subfamilies TPS-a, TPS-b, and TPS-g, contain 5 to 7 introns and 6 to 8 exons; most of the *OsaTPS* of TPS-a subfamily contains six, introns and seven exons in their gene structure and expected to fall in class III TPSs, except *OsaTPS*4 which contains only three introns and four exons and supposed to be an incomplete sequence. *OsaTPS* genes are containing seven introns and eight exons likely to have disrupted 3’exon by an extensive or probably insertion of the new intron. *OsaTPS* of TPS-b subfamily genes contain 5, 6 and 7 introns with 6, 7 and 8 exons, except *OsaTPS*21 which contains only four exons due to incomplete sequence, while *OsaTPS* of TPS-g subfamily genes contain 5, 6 and 7 introns and 6, 7 and 8 exons and fall in class III TPSs. Genes of *O*. *sanctum* from the remaining subfamilies; TPS-c, TPS-e, and TPS-f contain introns between 9 to 14 and exons from 10 to 15 which fall with in class I TPS gene family. Here TPS-d is not observed in the study, as it is specific to gymnosperm which falls in class II TPS gene family (Fig 2).

According to the conserved motif based TPSs classification, in *O*. *sanctum* an 18 *OsaTPS* falls in TPS-a subfamily, 16 *OsaTPS* falls in TPS-b, two *OsaTPS* in TPS-e, one *OsaTPS* in TPS-f, and five *OsaTPS* fall in TPS-g subfamily, these all subfamilies of terpene synthases are of class I TPSs. While five *OsaTPS* falls with in TPS-c subfamily which is of class II terpene synthases.

### Phylogenetic analysis of terpene synthase gene in *Ocimum sanctum*

**Fig 3:**
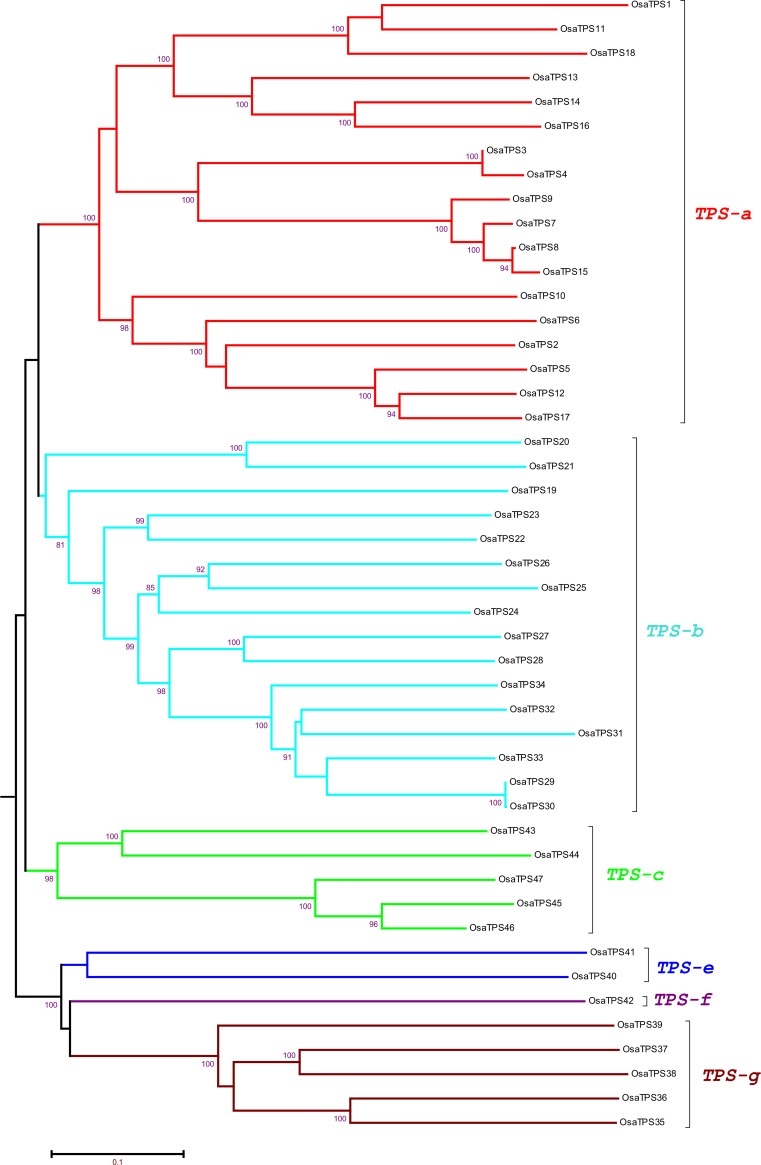
Discovered 47 putative functional TPS genes from *O*. *sanctum*: Neighbor-joining phylogenetic tree was constructed using 1,000 bootstrap value and classified into terpene synthase gene subfamily as TPS-a, -b, -c, -e, -f and TPS-g. Bootstrap values assigned in the tree, which was higher than 80%, values below 80% were not shown in the figure.

**Fig 4:**
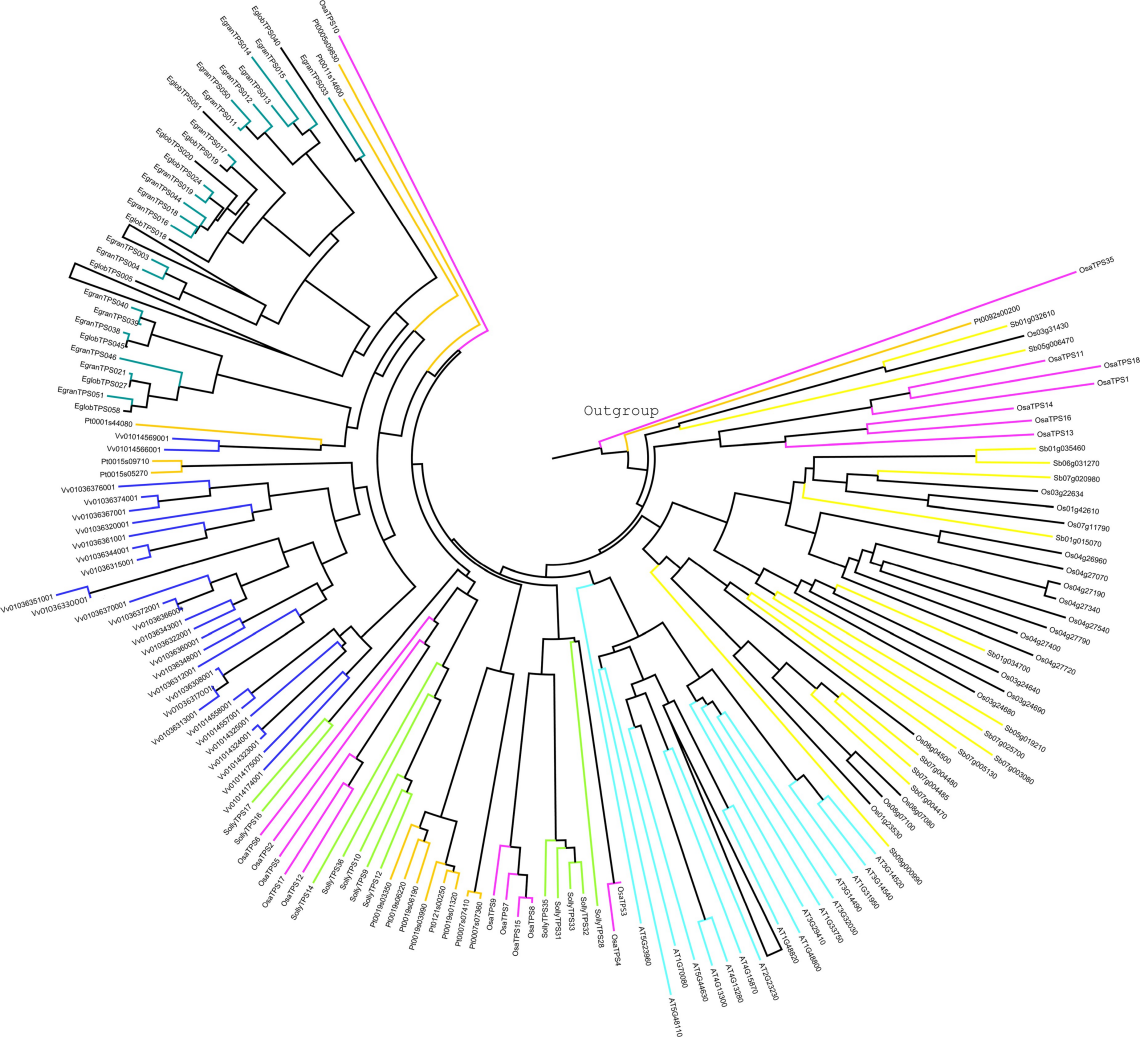
Representation of phylogenetic tree of *O*. *sanctum* compared to other species of TPS-a subfamily. 18 *OsaTPS* represented in the tree were compared with the neighboring species. The Neighbor-joining method was used with 1,000 replicates for bootstrap values. The values = 80% and = 95% were designated in S2 Fig. *OsaTPS*35 and Pt0092s00200, two TPS gene were used as an out-group.

**Fig 5:**
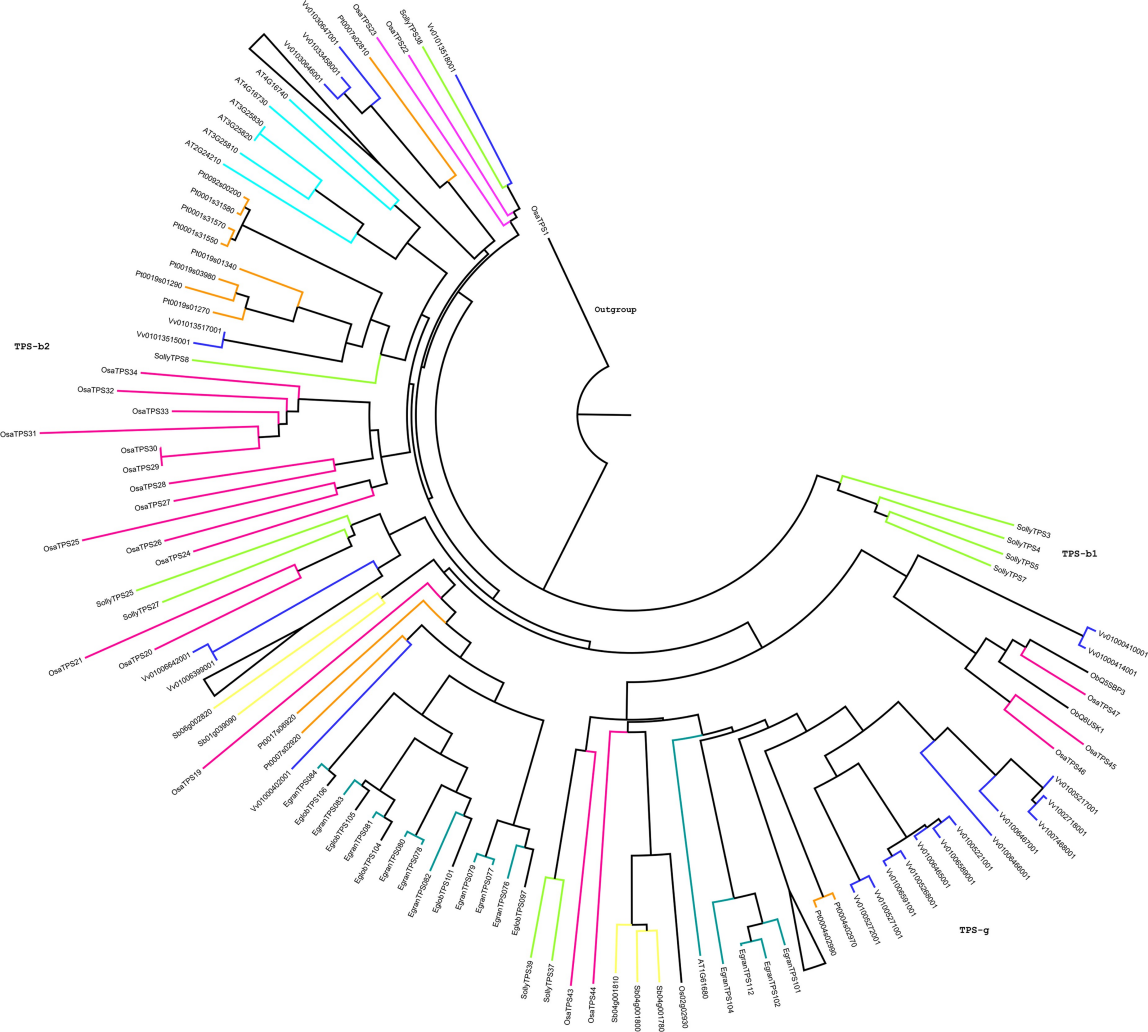
Phylogenetic tree of putative functional TPS genes from *O*. *sanctum* were represented, here TPS-b and TPS-g were grouped in a single tree. TPS-b was sub-classified into TPS-b1 and TPS-b2, where TPS-b1 shows only *S*. *lycopersicum* species TPS genes and TPS-b2 shows mixed species in which 16 *OsaTPS* were shown. In TPS-g subfamily 5 *OsaTPS* were represented. The phylogenetic tree was constructed with 1,000 replicates for bootstrap values using the neighbor-joining method.

**Fig 6:**
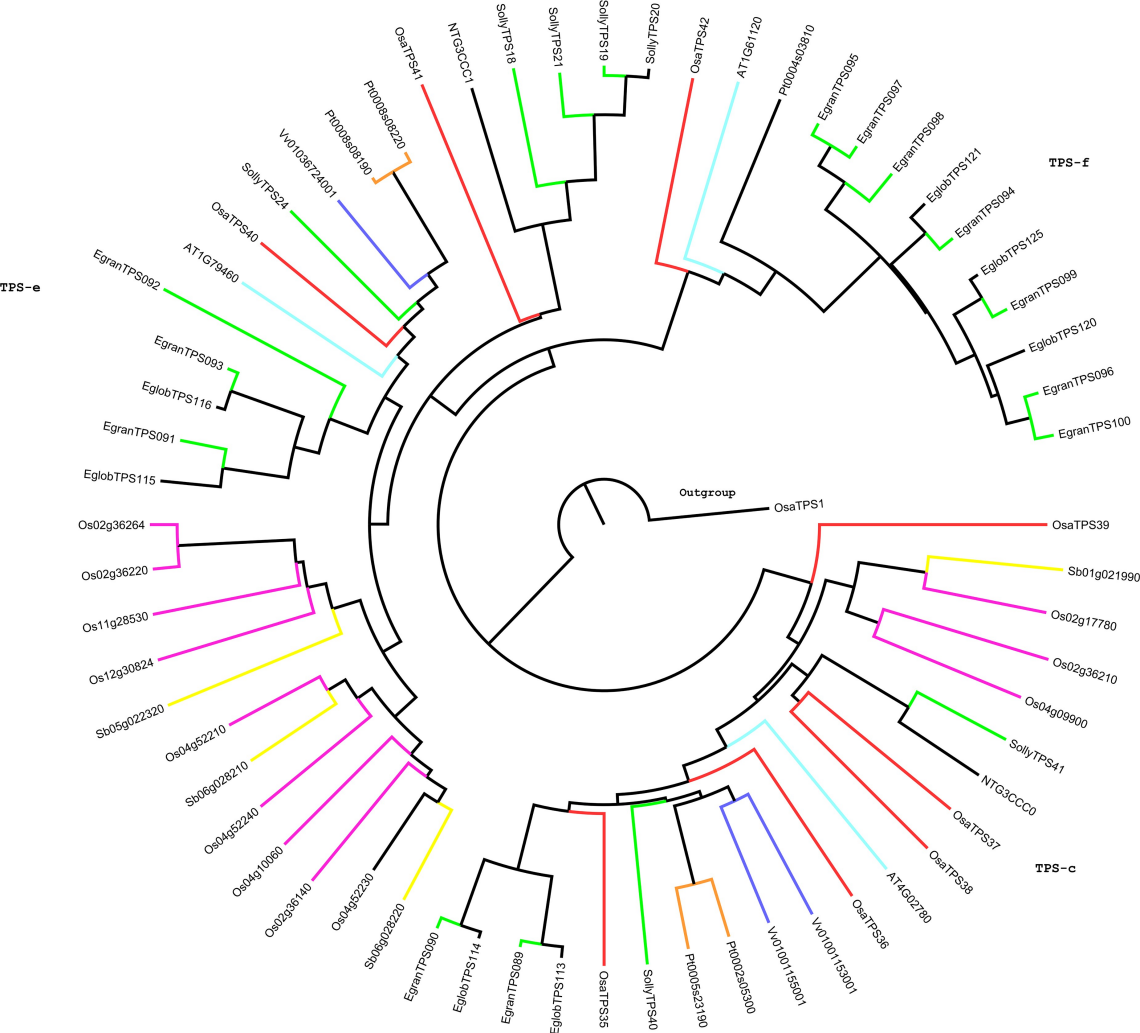
TPS-c, -e, and TPS-f, combined phylogenetic tree were represented from *O*. *sanctum* putatively functional TPS genes compared with other species. The phylogenetic tree was constructed with 1,000 replicates for bootstrap values using the neighbor-joining method. Here in TPS-c clade five *OsaTPS* were shown and compared, TPS-e and TPS-f show two *OsaTPS* and single *OsaTPS42*. Whereas *OsaTPS1* was shown as outgroup.

The phylogenetic analysis of putative functional 47 *OsaTPS* genes was performed to know the evolutionary relationship between the *O*. *sanctum* and other plant species terpene synthase sequences from, *Arabidopsis thaliana*, *Oryza sativa*, *Vitis vinifera*, *Solanum lycopersicum*, *Populus trichocarpa*, *Sorghum bicolor*, *Ocimum basilicum*, *Nicotiana tabaccum*, *Eucalyptus grandis* and *Eucalyptus globulus* shown in S1 Fig and S1 File. The previous phylogenetic tree of TPS protein sequences from gymnosperms and angiosperms was divided into seven major subfamily clades designated as TPS-a through TPS-g [9,64,65] (Fig 3). The phylogenetic analysis of *OsaTPS* gene formed only six clades as; (i) TPS-a (Fig 4) (ii) TPS-b and -g (Fig 5) (iii) TPS-c, -e and–f (Fig 6). TPS-d is missing from this analysis as it is gymnosperm specific and TPS-h is specific to the spikemoss *Selaginella moellendorffii* [10].

In TPS-a subfamily clade, *O*. *sanctum* TPS genes formed orthologous pairs with closely related species *Sorghum bicolor* and *Solanum lycopersicum* and speciation clusters with distantly related species as depicted in (Fig 4 and S2 Fig). In the tree, it was observed that *Arabidopsis thaliana* and *Sorghum bicolor* have a longer branch length than *Ocimum sanctum* and *Solanum lycopersicum*, which suggest a prolonged period of gene differentiation without gene duplication events. TPS-a group consists of all the known angiosperm sesquiterpene and diterpene synthase proteins, along with predicted *OsaTPS* genes, it is, therefore, most likely that 18 *OsaTPS* clustered with this group are also members of sesquiterpene and diterpene synthases.

TPS-b and -g phylogenetic tree clusters, shows 16 and 5 members of *OsaTPSs*, respectively, these clades consist of known angiosperm monoterpene or isoprenes synthase genes (Fig 5 and S3 Fig). The subfamily TPS-b and TPS-g were shown in a combined state with the representation of separate clusters; it was observed that all 5 *OsaTPS*-g subfamily genes formed an orthologous pair with *S*. *lycopersicum* and *O*. *basilicum* genes. TPS-b further divided into two subclades; TPS-b1 and TPS-b2, only Solly TPS3, 4, 5 and 7 lies in the TPS-b1 subclade, these TPS belongs to monoterpene synthase, while TPS-b2 sub-cluster contains multiple species, in which *OsaTPS*24, 25, 26, 27, 28, 29, 30, 31, 32, 33, and *OsaTPS*34 formed a separate cluster with longer branch length, where *OsaTPS*20, 21, 22, and *OsaTPS*23 form a cluster with *S*. *lycopersicum* genes.

The TPS-c, -e, and -f of *O*. *sanctum* shared five members of genes with TPS-c, two with TPS-e and one with TPS-f clades. These all subfamilies were shown in a single phylogenetic tree (Fig 6) and represented with the labeling of different TPS subfamily clades as shown in S4 Fig. All the *OsaTPS* were indicated in the red color branch, *OsaTPS*42 lies in the subfamily of TPS-f with *A*. *thaliana*, *Poplar* and *Eucalyptus* species, where TPS-e consist of *OsaTPS*40 and *OsaTPS*41 sharing the cluster with orthologous species *S*. *lycopersicum* and *N*. *tabacum*. TPS-c cluster shared five members of *OsaTPS* genes, and formed an orthologous pair with multiple species, *OsaTPS*37 and *OsaTPS*38 formed a pair with *S*. *lycopersicum* and *N*. *tabacum*, whereas *OsaTPS*35 and *OsaTP*35 paired with multiple species.

### Conserved sequence motif analysis of *O*. *sanctum* TPSs

**Fig 7:**
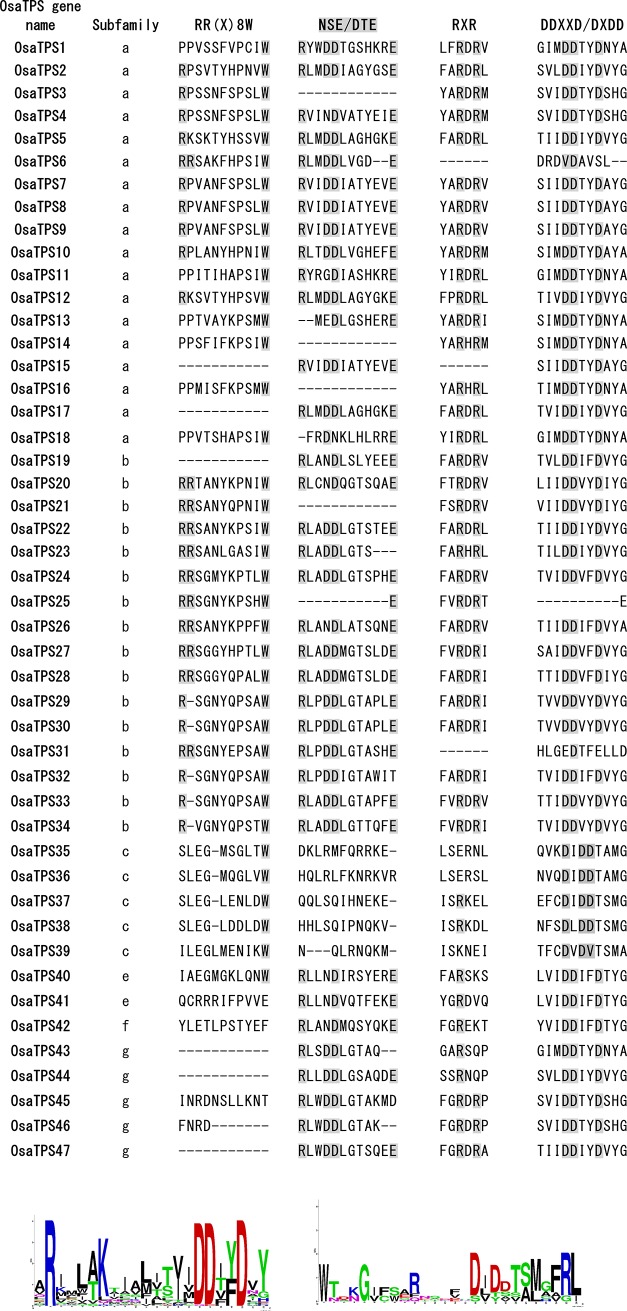
Conserved motifs of the TPS gene family presented in the *O*. *sanctum* putative functional TPS genes were shown. All the conserved amino acids were highlighted in the grey shades. 47 *OsaTPS* were shown and classified into TPS gene subfamily. Here RR(X)_8_W conserved motif is conserved in *OsaTPS*-b subfamily except for *OsaTPS*19 (angiosperm monoterpene synthase). Variation of the RR(X)_8_W motif is found in *OsaTPS*-a subfamily of sesquiterpene and diterpene synthase that have putative N-terminal plastid peptides, *OsaTPS*-c and–e lack twin-arginine residues but consist only tryptophan residue. NSE/DTE motifs were continuously seen conserved except *OsaTPS*-c subfamily. Motif RXR in all *OsaTPS* are shown genes either in same or modified form except some *OsaTPS*. Motif DDXXD and DXDD were shown highly conserved in all putative functional *O*. *sanctum* TPS genes, but in two genes, i.e., *OsaTPS*6 and *OsaTPS*25 DDXXD motif is absent, which might be due to sequence assembly error.

All the discovered *OsaTPS* were investigated for the presence of terpene synthase conserved motifs, i.e., RR(X)_8_W, RXR, DXDD, DDXXD, and NSE/DTE; except these motifs, 20 more motifs has been explored. Monoterpene and diterpene synthases typically contain an N-terminal plastidial targeting peptide upstream of the conserved or modified RR(x)_8_W motif, this conserved motif was predicted in 31 *OsaTPS* genes in exact or in modified form, which resemble sesquiterpene, diterpene and monoterpene synthases. In TPS-b clade RR(x)_8_W motif was not found to be conserved in all *OsaTPS* genes (Fig 7). The most conserved DDXXD motif of the class I TPS gene family was observed in 39 members of *OsaTPS*, also represented in sequence motif logo (Fig 7). The DDXXD motif is present on the N-terminal region of positional 35 amino acid downstream to RXR/RDR motif; it plays a role in the complexation of the diphosphate group after ionization of substrate. At the c-terminal, a fully conserved RDR motif was found in most of the *OsaTPS* sequences, with some variations: R(D/H)R, some of the sequences have variation of this motif as R(D/H)(R/K/D/V/Q) and tree sequences were shown missing this motif due to sequencing error. The additional metal cofactor binding motif NSE/DTE, is less conserved in TPS, evolved from a second aspartate-rich region in prenyl transferases to form a consensus sequence of (L,V)(V,L,A)(N,D)D(L,I,V)x(S,T)xxxE [66]. In our prediction motif: NSE/DTE form consensus sequence of (L,V,Y)(I,A,M,P,W,S,C)(N,D,G)D(L,I,V,M,K,Q)x(T,G,S)xxx(E,T) and it was observed in all the *OsaTPS* gene sequences except *OsaTPS*3, 14, 16, 21, and 25 (Fig 7). Motif DXDD was found only in *OsaTPS*-c subfamily (Fig 7), with sequence similarity range of 62% to 68% with TPS-c specific ent-copalyl diphosphate synthase, this motif was conserved in class II TPS genes, this class of TPS lack DDXXD motif and have non-active α-domain [66].

### Functional annotation and pathway mapping of *Ocimum sanctum* TPS genes

Discovered 47 *OsaTPS* of *O*. *sanctum* were annotated with the UniProt protein database to know their sequence similarity to the well-characterized terpene synthase genes available till date Table 2. The classified *OsaTPS* subfamily substantially mapped with the TPS genes of different species of Lamiaceae family; *Ocimum basilicum*, *Origanum vulgare*, *Pogostemon cablin*, *Mentha x piperita*, *Salvia officinalis*, *Lavandula angustifolia*, and with other species.

**Fig 8:**
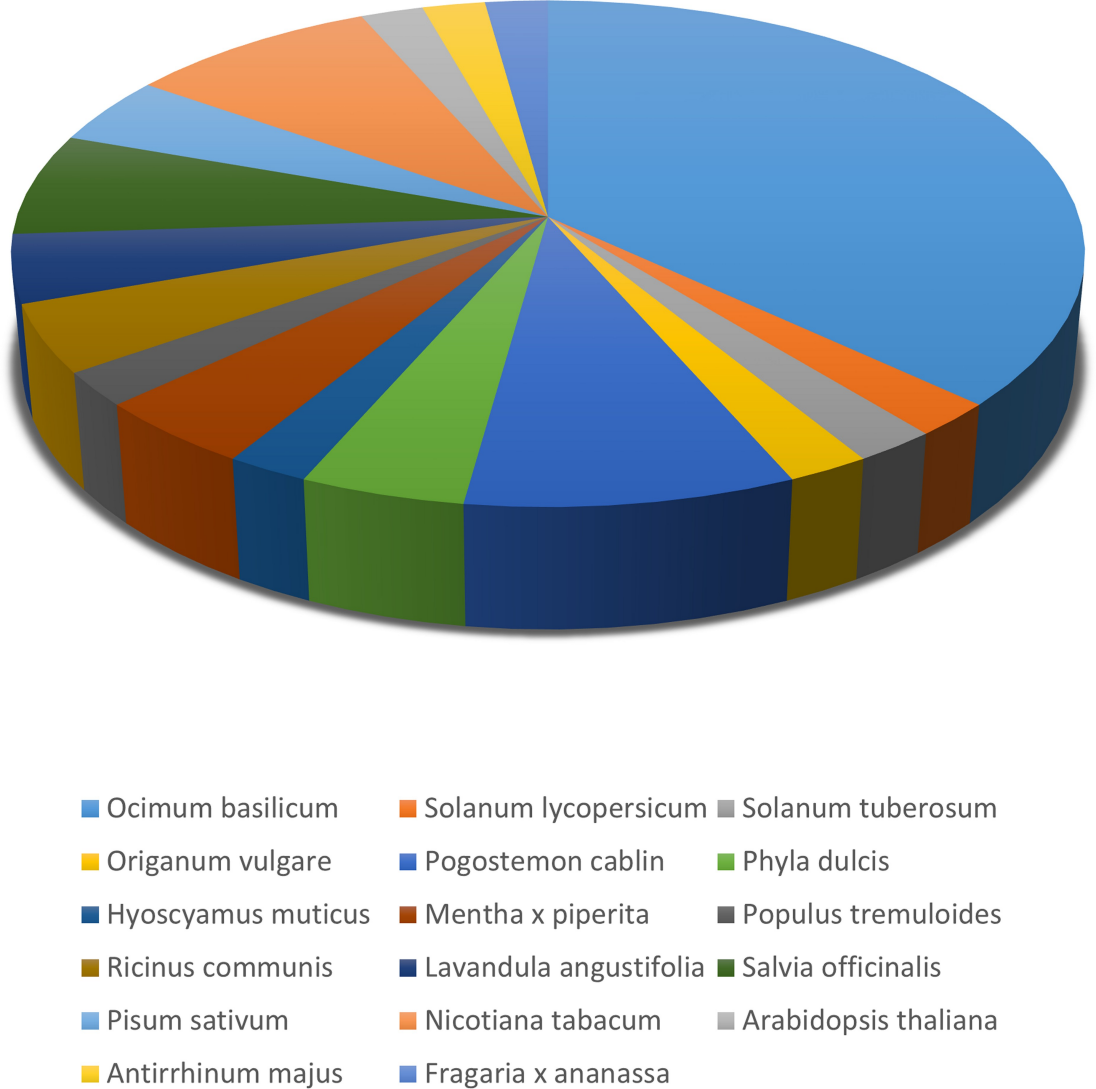
Annotation result of 47*OsaTPS* protein sequences, show higher sequence homology with *Ocimum basilicum* followed by *Nicotiana tabacum*, *Pogostemon cablinsesamum* and *Salvia officinalis* species. The pie chart represents the sequence similarity matching of *OsaTPS* with different plant species.

Gene ontology search was done to search the associated hits by sequence homology for their respective Gene Ontology (GO), Kyto Encyclopaedia of Genes and Genomes (KEGG) and Enzyme commission codes (EC) for each query sequence and the highest bit score hit were selected. Annotation against the GO database yielded significant annotation for 47 *OsaTPS* gene representing the best hits. All the *OsaTPS* genes were classified into three major components, the Biological Process (BP), Cellular Component (CC) and Molecular Function (MF). In (BP) all 47 *OsaTPS* genes were representing their participation in biosynthetic process and lipid metabolism process, whereas 35 *OsaTPS* among 47 tend to participate in the catabolic process, 28 *OsaTPS* among 47 participate for another process. *OsaTPS* distribution in (CC) was represented in three components as 29 *OsaTPS* shown in the plastid, 6 *OsaTPS* in the cytosol and 5 *OsaTPS* in the cytoplasm. In Molecular function (MF) all 47 *OsaTPS* show lyase activity, where 41 among the 47 shows ion binding activity, and 12 *OsaTPS* genes show isomerase activity, along with this analysis (Fig 8 and S4 Table).

**Fig 9:**
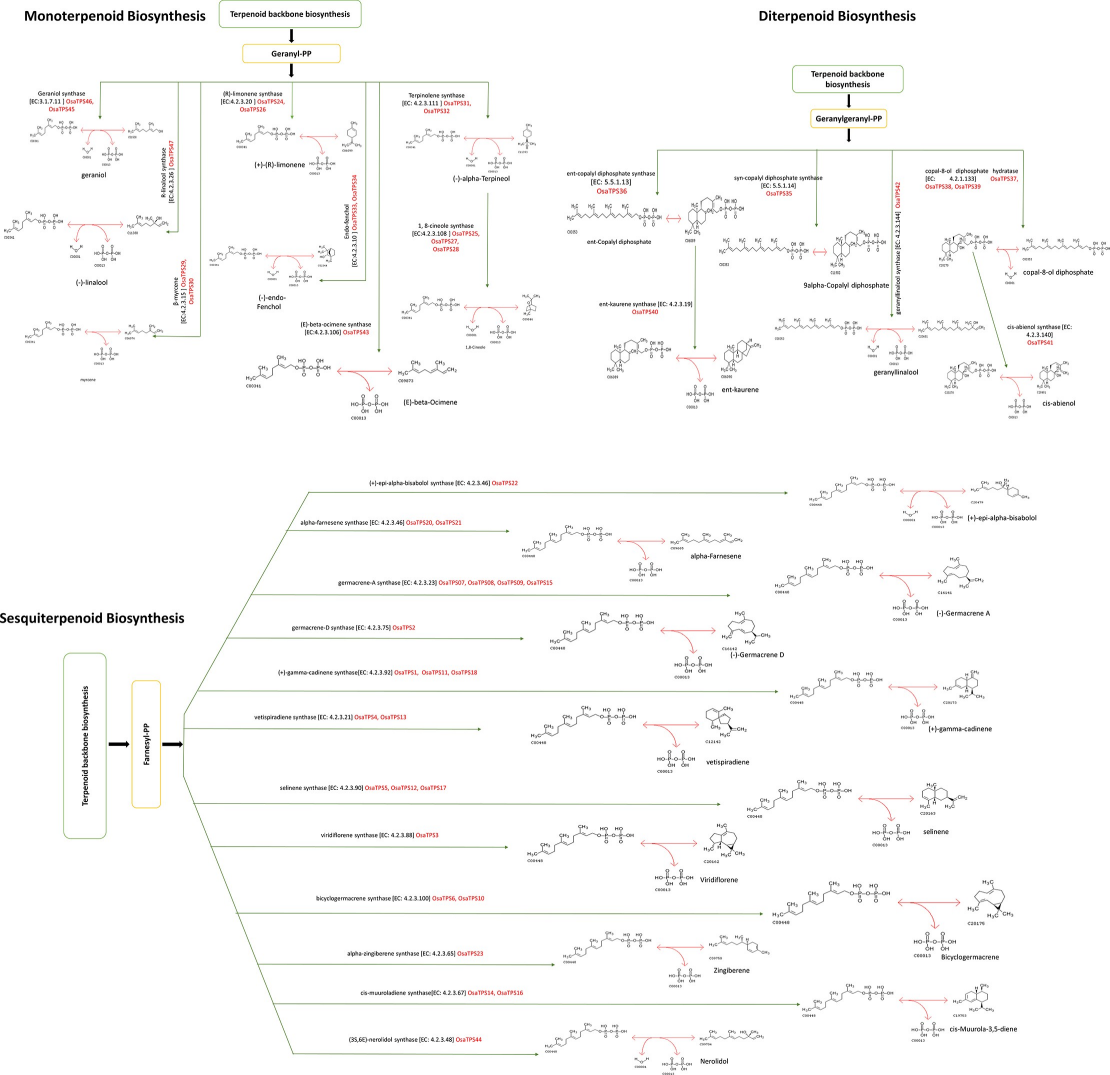
Proposed secondary metabolite synthesis pathway was constructed for *O*. *sanctum*. Monoterpenoid, Diterpenoid and Sesquiterpenoid pathways show the presence of putatively functional *OsaTPS* genes.

KEGG pathway mapping against the *OsaTPS* genes was performed to predict and hypothesize the secondary metabolite biosynthesis pathway. In monoterpenoid biosynthesis pathway, total 15 *OsaTPS* genes were found, which may be involved in the synthesis of the particular secondary metabolite. Similarly, in the diterpenoid pathway, 8 *OsaTPS* genes show their presence, whereas the maximum 23 *OsaTPS* genes were observed in the sesquiterpenoid biosynthesis pathway (Fig 9). *OsaTPS19* shows homology with the isoprene synthase gene, which is used to synthesize the isoprene from dimethylallyl diphosphate.

## Discussion

*Ocimum* genus is a significant genus in the Lamiaceae family; it was considered as an important medicinal plant from centuries and at present also used in flavor and fragrance industries due to the occurrence of some aroma compounds like α-Pinene, camphene, eugenol, limonene, camphor in their essential oil. The *Ocimum sanctum* is rich in mono and sesquiterpenes, which are essential and interested in their contribution to industries. Alot of research is done in *Ocimum* species on phenylpropene class for characterization of the responsible genes for synthesis of eugenol, methyl chavicol, chavicol and their derivatives [25]. Being such an essential plant for human life, this species has not been explored much in the context of molecular characterization of terpene synthases genes. In this study, we have used genome data of *Ocimum sanctum* [67] which is 386 Mb in size along with the plastid genome of 142,245 bp and known to be smallest in Lamiaceae family till date. In this study, the newly sequenced genome of *O*. *sanctum* was compared with *Salvia miltiorrhiza*. The comparative study reveals the two genomes to be most similar and also share identical diploid number of chromosome (2n = 16). It also reveals that *O*. *sanctum* genome is almost half the size of *S*. *miltiorrhiza* and appears to be relatively compact with quite fewer repeat sequences, while it falls in the identical phylogenetic clade with *S*. *miltiorrhiza*.

*S*. *miltiorrhiza* and *O*. *sanctum* both are rich in phenylpropanoids and their derivatives, which are well known for their therapeutic activities in Chinese and Indian traditional medicine systems. Although in *O*. *sanctum* the presence of a large number of terpene synthases homologs emphasizes to explore more specific and medicinally important terpene synthase genes in the plants. From this previous study, we have methodically identified the 47 putatively functional terpene synthases in *O*. *sanctum* which were responsible for the biosynthesis of volatile terpenes. These predicted *O*. *sanctum* TPSs were divided into seven TPS sub-families according to the standard TPSs subfamily classification methods [6,68]. We now extended the analysis by including newly identified TPS sequences from the sequenced genome of *Ocimum sanctum*. Out of 47 *OsaTPS* genes maximum number of genes is the member of TPS-a subfamily from *OsaTPS*1 to *OsaTPS*18. This clade is composed explicitly of angiosperm sesquiterpene and diterpene synthases and contains conserved DDxxD motif in the c-terminal domain of TPS which is supposed to combining metal ions, with less conserved RR(X)_8_W motif presents at downstream of the N-terminal transit which confirms the presence of typical sesquiterpene and diterpene synthases. *OsaTPS* of TPS-a group that lack RR(X)_8_W motif does not have the twin-arginine residue but have conserved tryptophan residues.

In TPS-a, subfamily, members of *O*. *sanctum* either formed separate cluster or share clusters with *S*. *lycopersicum* (Fig 4 and S2 Fig) and shows orthologous relationship with Class I specific sesquiterpene synthases of TPS-a subfamily genes i.e., cadinene synthase, Germacrene-D-synthase, Viridiflorene synthase, Selinene synthase, Bi-cyclogermacrene synthase and *Cis*-muuroladiene synthase. The previous subfamily classification suggests that TPS-b contains conserved RR(X)_8_W belongs to monoterpene synthases and synthesizes angiosperm monoterpenoids, while in *O*. *sanctum* it was seen that *OsaTPS*20 to *OsaTPS*28 and *OsaTPS*31 genes contain highly conserved RR(X)_8_W motif which confirms the predicted *OsaTPS* genes to be monoterpene synthases. Other *OsaTPS* of this class lack conservation of this motif. In TPS-b subfamily, *OsaTPS* shared common clusters with *S*. *lycopersicum*, *S*. *bicolor* and with *O*. *basilicum*, all *O*. *sanctum* TPS genes from *OsaTPS*19 to *OsaTPS*34 shows an orthologous relationship with a subfamily of TPS-b terpene synthase genes as represented in S3 Fig. Five *OsaTPS* genes were observed in TPS-c subfamily from *OsaTPS*35 to *OsaTPS*39, these TPS genes contain only DxD(D/V) motifs and lack DDxxD motif, and suggested to be monofunctional gene of class II specifically Copalyl diphosphate related diterpene synthases, these features and functional sequence annotation of *OsaTPS* confirm the sequences to me Copalyl diphosphate synthases.

The TPS-c, -e and -f subfamilies of *OsaTPS* were closely related to each other and shared a common cluster with *N*. *tabacum* and *S*. *lycopersicum*. TPS-e/f contains all valid kaurene synthases proteins from angiosperms which encode and annotate *OsaTPS*40, the presence of DDxxD motif but not DxDD motif and annotation with kaurene synthase TPS gene confirm it to be a class I TPS gene as mentioned in S4 Fig. The TPS-g is a clade presented closely related to the TPS-b family as shown in (Fig 5) and lack RRx_8_W motif; it is defined and classified as monoterpene synthases which produce cyclic floral aroma compounds like Ocimene and Myrcene which firstly characterized in snap-dragon [64].

Plant secondary metabolism pathway produces a large number of specific compounds. These compounds do not play a direct role in the plant growth and development but do help plants to survive in its environment. These metabolites were synthesized by specialized enzymes know as terpene synthases. In this study, we discovered a large number of terpene synthase genes in *O*. *sanctum* genome although from a biological point of view it is not necessary that if the plant contains a large number of TPS genes it will synthesize the higher diversity of terpenoids because single terpene synthase gene can synthesize several terpenes from a single substrate. In a family Lamiaceae, some TPS genes have been characterized till date, so far only 11 TPS gene are reported in *Ocimum basilicum* of which, nine were characterized [25]. In *Lavandula amgustifolia*, only 10 TPS genes were found among which only three were characterized. Likewise in *Mentha x piperita*, only six TPS genes were found, and four were functionally characterized so far, this comparison among closely related species of *O*. *sanctum* shows that *O*. *sanctum* possesses a large number of putative functional TPS genes.

The functional annotation and pathway mapping of *OsaTPS* genes; reveals the putative function for production of secondary metabolites. In Fig 9, the predicted 47 *OsaTPS* genes mapped with secondary metabolite biosynthesis pathway, which represents the presence of biosynthetic genes in the species. *OsaTPS*46 and *OsaTPS*45 mapped with geraniol synthase which synthesises geraniol, also reported in *Ocimum basilicum* species. Similarly we identified R-linalool synthase (*OsaTPS*47), (-)-Endo-fenchol synthase (*OsaTPS*33, and *OsaTPS*34), Selinene synthase (*OsaTPS*5, *OsaTPS*12 and *OsaTPS*17), Terpinolene synthase (*OsaTPS*31, *OsaTPS*32), β-myrcene synthase (*OsaTPS*29 and *OsaTPS*30), α-zingiberene synthase (*OsaTPS*23), γ-cadinene synthase (*OsaTPS*1, O*saTPS*11 and *OsaTPS*18), and Germacrene-D synthase (*OsaTPS*2). Our study shows that a number of terpene synthases gene in *Ocimum* species (*Ocimum sanctum*) might be present and code other terpenoids of secondary metabolite pathway, especially in diterpene and sesquiterpene biosynthetic pathways. In diterpenoid biosynthesis *O*. *sanctum* predicted gene mapped with different TPS enzymes like Ent-copalyl diphosphate synthase (*OsaTPS*36), Syn-copalyl diphosphate synthase (*OsaTPS*35), Ent-kaurene synthase (*OsaTPS*40), Copal-8-ol diphosphate synthase (*OsaTPS*37, *OsaTPS*38, and *OsaTPS*39), and Cis-abienol synthase (*OsaTPS*41), these all diterpene synthase genes were still unexplored in this species. Similarly in sesquiterpenoid biosynthesis *OsaTPS* genes mapped with (+)-Epi-alpha bisabolol synthase (*OsaTPS*22), α-Farnesene synthase (*OsaTPS*20 and *OsaTPS*21), Germacrene-A synthase (*OsaTPS*07, *OsaTPS*08, *OsaTPS*09 and *OsaTPS*15), Vetispiradiene synthase (*OsaTPS*4, *OsaTPS*13), Viridiflorene synthase (*OsaTPS*3), Bicyclogermacrene synthase (*OsaTPS*6 and *OsaTPS*10), *Cis*-muuroladiene synthase (*OsaTPS*14 and *OsaTPS*16) and (3S, 6E)-Nerolidol synthase (*OsaTPS*44) (Fig 9).

The chemical composition of *O*. *sanctum* essential oil also suggests the presence of a large number of terpenoids in the plant, which were synthesized by specific TPS genes, but few TPS gene studies have been done at molecular level. Therefore, there is a need for characterization of more TPS genes to know their actual involvement in the biosynthesis of *Ocimum sanctum* secondary metabolites.

## Conclusion

In the present study, we have discovered and analyzed the terpene synthase genes families from *Ocimum sanctum* genome by using TPS gene HMM models, which is used to identify genome-wide terpene synthase genes. The study provides the first comprehensive prediction and annotation of the very large *OsaTPS* (*O*. *sanctum TPS*) gene family about genomic structure, phylogenetic subfamily classification, motif localization and enzyme pathway mapping. Results showed that *OsaTPS* gene family is one of the largest gene family of specialized secondary metabolism in *Ocimum* species as there is no prior information about *O*. *sanctum* TPS genes. The predictions are exploring and unfolding the most significant gene family, which has been expanded across the genome through gene duplication and functional diversifications. Remarkably, a large number of functionally diverse sesquiterpene, diterpene and monoterpene synthase, which are identified in our study may contribute to enlighten the way for researchers. The phylogenetic analysis of *OsaTPS* showed similar results in many of the plant species studied so far and lies within all six gymnosperm TPS genes classified subfamilies, *i*.*e*., TPS-a, TPS-b, TPS-c, TPS-e, TPS-f, and TPS-g. This study may provide a deeper understanding of the *O*. *sanctum* putative functional genes phylogenetic classification and may help the researchers in the characterization of discovered *OsaTPS* genes to enhance the metabolic process of *O*. *sanctum*. This study represents the first report for the identification of *O*. *sanctum* TPS genes and their subfamily classification. These findings are initial steps for a better understanding of *O*. *sanctum* TPSs genes. Further characterization is required to determine the proper function of enzymes in secondary metabolism.

## Supporting information

S1 Fig Multiple sequence alignment was performed and a phylogenetic tree was generated using neighbor joining method with 1,000 replicates for bootstrap values. Complete phylogenetic tree of 14 species terpene synthase genes, including newly sequenced *O*. *sanctum* and discovered putative functional 47 *OsaTPS* was generated. 437 TPS genes were included to generate the phylogenetic tree, with 47 *OsaTPS* gene, and categorized into the TPS-subfamily (TPS-a, -b, -c, -e, -f and TPS-g) as shown in the figure. All the newly discovered *OsaTPS* genes were designated in green color, where TPS subfamilies were assigned with different branch colors mentioned in figure legends.
(TIF)

S2 Fig Phylogenetic rooted tree of all TPS-a subfamily of different species was generated with the Neighbor-joining method, where 1,000 bootstraps replicated were considered and compared with *O*. *sanctum* TPS genes which were shown in pink color branches. The bootstrap values ≥80% were designated in red color triangles and values supporting ≥95% were designated in red circles above the branches, *OsaTPS*35 and Pt0092s00200 were shown as outgroup.
(TIF)

S3 Fig TPS-b and TPS-g subfamily phylogenetic tree were constructed using the Neighbor-joining method with 1,000 bootstrap replicates. O. sanctum TPS genes were shown in pink color branches. TPS-b was subdivided into TPS-b1 and TPS-b2, where TPS-b1 shows only *S*. *lycopersicum* species and TPS-b2 show multiple species with 16 TPS genes from *O*. *sanctum*. TPS-g subfamily shows 5 *OsaTPS* genes. Both the TPS subfamily were designated and colored in different colors, *OsTPS*1 was used as an outgroup.
(TIF)

S4 Fig The combined phylogenetic tree was generated using the Neighbor-joining method with 1,000 bootstrap replicates values. TPS-c, -e and TPS-f subfamily were joined together. *OsaTPS*1 is used as an outgroup. Terpene synthase genes of *O*. *sanctum* was shown in red branch color.
(TIF)

S1 Table Representation of *O*. *sanctum OsaTPS* genes with information of, genome localization, TPS subfamily clusters and gene length. (XLSX)

S2 Table Representation of *O*. *sanctum OsaTPS* partial genes and pseudogenes with subfamily classification. (XLSX)

S3 Table Cluster id of all TPS genes from different species included in phylogenetic tree analysis. (XLSX)

S4 Table Representation of *O*. *sanctum* OsaTPS genes with annotation, Gene ontology information, sequence alignment distribution and KEGG enzyme mapping. (XLSX)

S1 File All terpene synthase sequences retrieved from different plants species used for phylogenetic analysis. (DOCX)

## Abbreviations

OsaTPS: (*Ocimum sanctum* terpene synthase)
TPS: (terpene synthase)
