# Supplementary material for: Genome-wide detection of terpene synthase genes in holy basil (Ocimum sanctum L.)
Source: PLoS One. 2018 Nov 16;13(11):e0207097. doi: 10.1371/journal.pone.0207097 (PMC6239295; doi:10.1371/journal.pone.0207097)
Supplement: S1 File — (DOCX) [file pone.0207097.s009.docx]

**S2. All terpene synthase sequences retrieved from different plants species used for phylogenetic analysis**

>OsaTPS01-scaffold8511.g82728.t1

MDSSSSSPKVVDDVRNVRPPVSSFVPCIWTQTFSKFSFDEEEQRRYEEAISELKEEARGKLNAAATPIEQMVLVDTLERASIPYAQTASVPSFLRYTHVFDEFVDNNGKFIGDPEGLLSLYEATHVGFRDEKILQEAKRFTRHELSGMESQLQSPLKEKVKRALERPLHREVPIIHARLFISIYEKDDSMDEHLLKLAKYNFNFLQNLYKKELHELFRDRVAEAYLWGAGYHFEPQYTYVRKGFTLAIKILGIMDDTYDNYATLNEAQLFTEILDRWSMDEDDQLPDYMKTTLDFIMSAYEEYECYAQKNGKEFASPYFKETIQQLARAYNQELKWVMGEEMPSFQDYQKNSNITSCIYMLFASIIPGLKSLTQEAIDWIKSEPSFVKTVGMIGRYWDDTGSHKRENKGGKMLTVVDCYMKQYGVSEQETMSKFAEAIEDGWKDVNEGWVAMANFMPKEIAVQFLNYLRLCDATYDRNNGDGYTDPTLSKPTITSLFVQPLVISFQS

>OsaTPS02-scaffold943.g31068.t1

MAASNSNSNSRPSVTYHPNVWGDYFLSYASELTEISRAEREEHEKQKERVRNLLVQTRDDSSFKLQLIDSIQRLGVGYHFEKEIEKSLRYIYTTYYDTKDNDDLHIIALRFRLMRQQGFHVPCDVFKRFIDEEGNFKEWIKDDVEGILSLYEASHYGMHGEEILDKALEFCSSRLGQGMEDSLSVRTPISKTITRFGAKKFISGYQDISQLHHQPLLKFAISDFNMLQKVHQRELSQLTRWWKELDFGNKLPFARDRLVECYFWIVGVYFEEGYAVARRLLTKVIYLASVLDDIYDVYGTLEELTIFTAVVQRWDINGMDKLPPYMRIYYKALLDVYFEMEYEMGKIGKSYTVEYAKQEMKRLAEVYFEEAKWSFNKYKPRMEEYMNVALLSSGYMMMAVNALAVIPHHISQEEFHWVLSEPSLLRASLTITRLMDDIAGYGSEEKLSAVHYYMSENGVSETEALVELGKQVKNAWKDLNKELVEPRASSNPILRCVVNFTRVIVVLYADGDAYGNSKTKTKELIKSILVDPIII

>OsaTPS03-scaffold1616.g42691.t1

MASATVQSFPRPSSNFSPSLWGDIFTSKSNSINDNAFYEKEFEELKEEIGTMIVAKEVDMAEKLNFIDSVERLGISYHFVREIDDQIHTLYDAFQEIENSDDLHTVALYFRLLRQHGRNISSDIFKKFTEDGGEFKNFLKDNPEGILSLYEASHLRTHNEEILDKALTFTTAHLQTIAPNLKSPLKEKVEHALEQPLQRGVPRVEARLYISAYEELEWKNDSLLRLAKLDYNLLQMLHRQELSELSRWWKELDLISKLTYARDRMVECYFWTVGVYFEPQYSRARVMLAKTIAMISVIDDTYDSHGTIDELQIFTEAVDR

>OsaTPS04-scaffold1616.g42692.t1

MASATVQSFPRPSSNFSPSLWGDIFTSKSNSINDNAFYEKEFEELKEEIGTMIVAKEVDMAEKLNFIDSVERLGISYHFVREIDDQIHTLYDAFQEIENSDDLHTVALYFRLLRQHGRNISSDIFKKFTEDGGEFKNFLKDNPEGILSLYEASHLRTHNEEILDKALTFTTAHLQTIAPNLKSPLKEKVEHALEQPLQRGVPRVEARLYISAYEELEWKNDSLLRLAKLDYNLLQMLHRQELSELSRWWKELDLISKLTYARDRMVECYFWTVGVYFEPQYSRARVMLAKTIAMISVIDDTYDSHGTIDELQIFTEAVDSYNIEAKWFIEGYMPPFSDYMANGFITSTYFLLAASSFMGITSASVEAFDWLMKKPRIQVANVTICRVIDDVATYEVCVAFTLAHYNIEAKWFIEGYMPPFSDYMANGFITSTCFLLAASSFMGITSASVEAFDWLMKKPRIQVANVTICRVINDVATYEIEKERGQSATGIECYMKDNGVSEEEAMQEFKNIAENAWKDMNGEMVKEKSVSMEILKRIINLSRLIDVVYKNNQDGYTHPEKVLKPLITALLLHSFQS

>OsaTPS05-scaffold2018.g48302.t1

MAQICESAAPISTNVEDIRKSKTYHSSVWGNHFLSYTADITEISAAEKEQLEKQKEKVKNSLAQTPDKSMAKMELIDAIQRLGVGYHFVKEIQESLRRIHEEQISKEEDDDDDDDDDDVRVVALRFRLLRQGGYRAPCDVFKRFTDREGNFKESLKKDVEGMLSLYEASYYGVNGEEIMDKALQFSSSHLESLVQQMSTTTTMTTITIFRRLQEALDTPISKASIRLGATKFVSTYREYESHDEDLLNFAKLDFNILQKMHQEEANHITRWWENLGSGNKFGFARDRLVECYFWSLGVYFEPHYKTARMFLAKVLAMTTIIDDIYDVYGSLDELRRFTHAVESWDIEVAYELPSYMRICYEALLSTYEEMENEMAKQGQSYRLHYAKQAMTESVKTYMKESEWCYNKTIPTMDEYMKVALISGGYLMVATTSLVGIKADFITKNDFDWIINHPPILQASEVLTRLMDDLAGHGKEQKITAVGCYMNEHGCSEMEASRELSKQVKKAWKDLNDEWMEPRSASMEILACIVNLSRVLHIMYSTGEDGFTDSTTRTKQSIKSLLVDPMN

>OsaTPS06-scaffold2410.g52924.t1

MESQSSVAYVKNLEETRRSAKFHPSIWGDHFLSYNSEKTEITVAENEEHAKQKEMVRSLLARVPDDSTHKMELIDAIQRLGVEYHFEKEIEEYLKYIHVEQICKDRDVDAVSLCFRLLRQGGYNVSSDVFNKFIDNKGNFVESLKDDVKGLLNLYEAAHLGAHGEEILERAIEFCSSHLQTFLRQTDNMPLSKRVDEALRMPNRRSLTRLGARKFISMYEEDESHYPTLLNFAKLDFNRVQKMHQRELSDATSWDNNMAMEDWPPYIQTCYKSIVDTYAGIEEQMEKEGKSYRVEYAKQDLKKLVMAYFEEAKWLYRDYVPTAEEYMKVSLVSCGYMMMSTSSLVGMGDLVTKQDFDWITNEPLIVRASSVICRLMDDLVGDE

>OsaTPS07-scaffold2421.g53050.t1

MAPAQAEIQRPVANFSPSLWGEQFIKHHSASHQVEGKHSRIVEVLKNEVRSMIDAGNMVDTMSLIDTLERLGVSYHFENQIEQKLHQYFDLNTDYNNEDYDLYTVALHFRLFRQHGYRLSADVFGRWRDENGKFQEGLKNDAKGLLSLYEASYLRTRGEPLLDDALGFATATLKSIAPRLESPLREQIEHALIQSLHFGNPRIEARKFISIYEQYEDKNESLLRFAKLDYNQLQMLHKEELDVVSRWWKDLDLVAKLPYARDRVVECFFWAMGVYHEPQYSRARIMLTKTIAMTSIIDDTYDAYGTIEELDVFTEAIERWNIEEMKRLPDYVKPFYKALLELYDQFEEELAKEGRSYAAHYAIESLKELVRSYHLEAKWFIQGYLPPFEEYLKNALITCTYCYHTTTSLLGVESAMKEDFEWLSKKPKMLVASLLICRVIDDIATYEVEKDRGQIATGIESYMKENCATKEEAVAKFFEIATDAWKDINEECMRPSPYSRDVLMRILNLERIIDVTYKGNEDGYTQPEKVLKPHIIDLFVDPIEN

>OsaTPS08-scaffold2677.g55836.t1

MARGQAEVQRPVANFSPSLWGEQFIKHDPAAHQVEEKHSKTIQALKNEVRSMIDSGNMIDTMSLIDTLERLGVSYHFENQIEEKLQQYFDLNADYNNETYDLYTVALHFRLFRQHGHRLSTDVFGRWRDENGKFQEGLKNDAKGLLSLYEASYLRTRGEPLLDDALDFATATLKSIAPRLESPLREQVEHALIQSLHFGNPRIEARKIEARKFISIYEQYEDKNESLLRFAKLDYNQLQLLHKEELHVVSRWWKDLDLVAKLPYARDRVVECFFWAMGVYHEPQYSRARIMLTKTIAMTSIIDDTYDAYGTIEELDVFTEAIERWNIEELKRLPDYVKPFYKALLELYDQFEEELAKEGRSYATHYAIESLKELVRSYHVEAKWFIQGYLPPFEEYLKNALITCTYCYHTTTSLLGVESATKEDFEWLSKKPKMLVASLLICRVIDDIATYEVEKDRGQIATGIESYMRDNGATKEEAVAKFFEIAMDAWKDLNEECMRPSPYSRDVLMRILNLERIIDVTYKGNEDGYTQPEKVLKPHIIALFIDPIQI

>OsaTPS09-scaffold5167.g73736.t1

MAPAVAEIQRPVANFSPSLWGEQFIKQDSAAHQVGEEEHSETVEVLKNEVRGMIGPGNMVDTMSLIDTLERLGVSYHFETEIEDKLQQYFDLNTDYNDEAYDLYTVALHFRLFRQHGHRISADIFARWRDENGKFQEGLKNDAKGLLSLYEASYLRTRGETLLDEAVEFATATLKSTAPHLESPLREQVEHALIQPLHFGNPRIEARNFISIYEEFGDKNESLLRFAKLDYNQLQMLHKEELHVVSRWWKDLDLVAKLPYARDRVVECFFWAMGVYHEPQYSRARIMLTKTIAMTSIIDDTYDAYGTIEELDVFTEAIERWHIDEINRLPEYIKPFYKALLELYEQFEEELADEGRSYAAHYALKELVRSYHLEAKWFIQGYLPPFEEYLKNALITCTYCYHTTTSLLGVETAIKEDFEWLSKKPKMLVAGLLICRVIDDIATYEVEKDRGQIATGIESYMKENGATIEETIAKFFEIAMDAWKDINEECMRPSPYSRDVLMRILNLERIIDVTYKGNEDGYTQPEKVLKPHIIALFVDPIQI

>OsaTPS10-scaffold6100.g77427.t1

MDITTSSRPLANYHPNIWGDRFLVYTPESHTGGEEELVEKLKKEVKLELIDALNDDVKLLKLVDAIQRLGIEYHFEEEIDHALQNFSENFDHFSRKNSDLFTLALGFRLLRQHGYRVSCSIFEQFKDGEGGFKATEVLAVLEFFEATHLRVHDEDVLDEASIFARNFLESALPSLSNPLAEQVHHALHEYSNRRGLTRVEARHYIPIYGQFESHHQGLLKLAKLDFNILQSMHKRELSEVYRWWKDLDVPTNFPYARDRMVETYFWIMGVYFEPKYALARKILVKLQSLASIMDDTYDAYATFQELQLFTQAIQRWSYSCLDKLPNYMRVCYKALLEAFEEFEEDITKQGISNHLSYAIEAMKVIDRDYFVEIKWREEKYKPRSEEYMQVATASCAYTSLIIISFVGMGDCVRKEDFEFVLSQPNIVRAALAICRLTDDLVGHEFEQEREHIPSWVECYTDEHKISKSEAVSEFKNRIESSWKDINEAFLNPTQIPTSQLYRILNFTRVIEVIYSKGDWYTHVGPQMQNLITQLLIQPIP

>OsaTPS11-scaffold6894.g79659.t1

MDALSIVDESLRNVRPPITIHAPSIWAHTFSKSSFKDQEQVQKKYEEAIEELKKEVRGKLMAAATPLEQMILIDTLERLGLAYHFESDIEHKLQQIYDDDVRGHECDLFATALRFRLLRQHRHRVSCAHVRFHDENILEEAERFTRHELSCMESQLERPLKDKVKRALERPLHREVPLIYARLFVSIYEKDDSMDEHLLKLAKFNFNFLQNLYRKELCDLSRWWNKFDLKTKLPYIRDRLVEAYLWGVGYHFEPQYSYVRKGLVLGIKIIGIMDDTYDNYATINEAQIFTEILDRWSMDEIDRLPDYMKIVLHFVMSAYEEYERDAEKNGKEFASPYFKETIQQLGRAYNQELKWVMGKQMPPFKDYLKNSEITSCIYPMFASIIPGLKSLTQESIDWIKSEPNFAVKAGLIGRYRGDIASHKRENKGGEILTVMDCYMKQYGVSTQETISKFAEAVEDSWKEVNEGWVGTISIPKEITVQFLNYSRVCDAIYNRNNGDGYTDPSVVKPTITALFVDPIVI

>OsaTPS12-scaffold6915.g79717.t1

MSQIYALAAPISTKNTNVEDIRKSVTYHPSVWKSYFLSYTSDITEISADEKEQLQKQKKKVQNLLAQTPDESTVKIELIDAIQRLGVGYHFVKEIEESLRQIHETQIREDEDDVRVVALRFRLLRQQGYRASCDVFKRFTDHEGNFKESLKKDVEGMLSLYEASYYGVNGEEIMDKALQFSSSHLQSLVQQTSTTTTITIFRRLQEALDTPISKTLIRLGATKFIYTYQEDESHDKDLLDFAKLDFNILQKMHQEEANHVTRWWEDMGLVKKLGFPRDRLVECYFWSLGLYFEPQYKAARIFLAKVLAMATIVDDIYDVYGSLDELRRFTHAIRSWDITAADELPPYMRICYEALLGVYEEMGDQIGVPYAIQTMKEMVESYLEEAEWCYTKYVPTMEEYMKLALVSVGYLMVATNSLAGINENSITKNDFDWIANRPPILRASELLTRLMDDLAGYGKEEKISAVSCYMKEHGCSEEEASRELWKQVNKAWKDLNDGWMEPRVASTEILARVVNQSRISNILYSTGEDGFSDSTKRTKQFVKSLLVDPIN

>OsaTPS13-scaffold10009.g84172.t1

MATANIVSAVSCDVRHLRPPTVAYKPSMWGDVFSNFHFDNKLQEKNVEPMEELKEETRSMLMAATSTKLMILVDKLERLGLAYHFQTEIEDRLKQVFDSPEEEDDHDLFATALRFRLKQVFDSPEEEDDHDLFATALRFRLLRQHQYNVSCNVFNKFVDEDGKWKETLSSHTEGILSLYEAAHVRIHDENILDEAVAFTVHHLTHGSTDEFLLKLAKLNFNFLQNIYRQELAELTRWWDKFDLKTKLFYARDRIVECYLWGNALRYEPRYSDLRLAVAKNMQLCSIMDDTYDNYATLEEDDLFTDILERLRTRSRKTGKIICTCLLQGSTFIPSMKSVDEDAVKWLLSEPKIVISTAKMGRSMEDLGSHERENREGKLPTVVDCYMKDKGISKQEALSDFSKLVENGWKDITAEWVKGSAAPKELMEHLLNYGRIAEVTYKNNEDGYTDPEKYVGPQIASLYMEPIPL

>OsaTPS14-scaffold7925.g81842.t1

MSSLKIFFFSGTAKKSAVRFPLKNGDVSHRHTVAFKDSAMMVNAKSKGVMDVRPPSFIFKPSIWGDIFSRFAIDDQVQQKYVEEIEALKNEVRSMLMTATSTKLMVLIDTLERLGLAYHFEIEIKNELKQVYIDREDDNEDYDLFATALRFRLLRQHQHHVSCSDFNKFVDKDNKLNETLGNDVVGLLSLYEATHVRIHDEHILDEAVAFTTHHLSRMLPQLESPLKEKVQHALEYPLHKSLTVLHMRFHILSYEEDDSSDELLLRLAKLNFNYLQNVYRNELHQLLKWYNKFDLQLKLPYARHRMEECYIWGMAHHFKPQHSYVRMVVAKTYQMLSIMDDTYDNYATIEELERFNRALESPIKQILFQMTLIGLMGGATKAT

>OsaTPS15-scaffold3188.g60854.t1

MGVYHEPQYSRARIMLTKTIAMTSIIDDTYDAYGTIEELDVFTEAIERWNIEEMKKLPDYVKPFYKALLELYDQFEEELAKEGRSYAAHYAIESLKELVRSYHLEAKWFIQGYLPPFEEYLKNALITCTYCYHTTTSLLGVESATREDFEWLSKKPKMLVASLLICRVIDDIATYEVEKDRGQIATGIESYMRDNGATKEEAVAKFFEIATDAWKDLNEECMRPSPYSRDVLMRILNLERIIDVTYKGNEDGYTQPEKVLKPHIIALFIDPIQI

>OsaTPS16-scaffold2155.g49924.t1

MMTDVRPPMISFKPSMWGDTFSTFIFDEKVQQKYFEEIETLRKEVRSMLMAATSTKLMILINKLERLGLAYHFQTEIENKLNQFYVDEKDEVEDYDLFQTALRFRLLRQHQYHVSCNDFNKFFDEENKLKETLYSDVAGLLSLYEAAHVRIHDEHILDEALAFTSHHLSCMMPELEAHLKEQVQHALEYPLHRSLTVLHIRFRILSFEEDESRDELVLRLAKLNFNYLQNIYKNELVQVSEWYNKFDLKSKLPYARHRLVECYTWGVAYHSKPRYSYVRMALAKTYQMLTIMDDTYDNYATIEELEVFTRALERWDIYEIDRLPDYIKPVYKFIMSVSEEFGRDADKAEKSYAIPYYIEGVRTNFIYNTFLADLFFHML

>OsaTPS17-scaffold4162.g68216.t1

EINATRKDQLEKQKKKVKNLLAQTPDESTIKIELIDAIQRLGVGYHFANEIQESLQKFHETQITKDDDDVRVVALRFRLLRQEGCRAPCDVFKRFMDQEGNFKESLKKDVEGMLSLYEASYYGVDGEEIMDKALHFSSSHLESLVQQMSTTTTTTTTIFRRLQEALDTPISKTLVRLGAVKFISMYQEDESHNKDLLNFAKMDFDILQKMHQEEARQVTRWWEDLGLGNKLGFARDRLVECYFWSLGVSFEPQHKAARIFLTKVLAVATVIDDIYDVYGGLDEVRRFTRAIRSWNISAADDLPPYMKICYEALLSVYEEMGDQIGVPYAIKTMKEMIEAYMEEAEWCYTKYIPTMDEYMKVALVSAGYRMVATTFLAGINRITRQDLDWIMNRPPILQASEILTRLMDDLAGHGKEAKIATVGCYMKEYGCSEMEASRELWKQVNKAWKDLNDGWMEPRAASRKILACIINQSRISNILYSTGEDGFSDSTTGTKQFIKSLLVDSMN

>OsaTPS18-scaffold1965.g47609.t1

MDDSVRNVRPPVTSHAPSIWANTFSESSFDPQVSILNHINMLSRLLLFELKLQRCNKDMKKQLKTQVRGKLMAAPTPIKQMIFIDTLERLGLTYHFETEIEQKLQQIYNDDVSGDGRDLFTTALRFFFAPTTSAPRVLRWWNKFDLKTKLPYIRDRLVEAYLWGVGFHFEPQYSYVRKGLALGIKVIGIMDDTYDNYATLNEAQIFTEILDRWSVDEINRLPDYMKTVFHFIMSAHEKYERDAEKNAKEFASPFFKETGLQSRTKMGYGKKDASIPRLSKEFRDNKLHLRRENKGGEVLTAVDCYMQHYHVSEQETISKFAEANEDAWKDVNEGWATMISMPKEIVVLFLNYSRLCDATYNRNNEDGYSDPNVFKQSIKALFVDPIVL

>OsaTPS19-scaffold1614.g42655.t1

MNKKFINNRGMLSLYEASNLAFNKEDYLDKARDYARSHLKKMIADVDKKFVEEVIHALEIPFHRRMSRSEAKWNIEMYDGKQSVTNQMLHKLAVMDFNKVQSLYQHELQQVVRWWRGVGLAKELSFARDRVMESFFWSIGMVWEPEFSKCRVGLAKVVLLITVLDDIFDVYGSPQDLHNLTQAVQKWDVNAGNHLPHCMKLFFLALYNTINDLAYQTLKDHHQLIIHQLSKGWADLCKAFLREAKWRCNKEIPRFYEYLDHGSISSSGVILLMHAYFLITPTIRDEALESLNDKHRLLRSSATIFRLANDLSLYEEEIERGEVKAVSCYMHETGSSNEDARAHMQKLMDQNWKVLNEEITTSDNAVFGKSFVGTSCNLARVALCQYQYGDAHSKPSDESRNRIILGIAKDAGNPVRIDDRFLAGDYGHYPYYSHCNVIGRWKQISREVVDGNRRGGSRERNCLRMEQEFSFSNCQLSLAKSFFRSSISLCQASCRTTSIAHFPLPNNFLAKEVIKGATKGMGLSCRLGYVDLG

>OsaTPS20-scaffold5960.g76950.t1

MEIVSAPLPRRTANYKPNIWNYDYLESLTNKFHEEKMDREAEKLRDELRSLLRASKDPLQKLELIDLINKLAISHHFEEEIFESIANTSTMKNHDLYSSALYLRILRQHGHHVPQDVVVRVLDEEETKMMNWDEKTRNEVFEASHLEVEGECLLDRAKAIPPVNAEKCPSHFSATWFNAKKHVLAVNKSPLHRLAGLSFNMIQVQHQKELKEILRWWKDLGLLEVLTFTRDRVVESFLWAVGVAYEPQYGSLRKWLTKAISLVLIIDDVYDIYGSKNELEQFTVAVERRVLMQLSSVKGRFFKWDPSEIQKLPEAIGRCFRTLYDTANDIDDEVQKEKGWSSVLPQLKEVWLGFCKALMVEARWADQKGQSPSLQEYLDNGWTSSSGPVLSLHVLLGIGDDMPETMAAFTGNRQLIHHTSLIIRLCNDQGTSQAELERGDVPSSILCHMREANVSEKEGREHIRKLTWDSWKKINESFMRCPEKHKRTMRYIVNTARVANFIYQNGDGFGVQDRETRDQVLSCLIHPWKL

>OsaTPS21-scaffold793.g27838.t1

MEMISATTNSPRRSANYQPNIWNYDYLISLTDNKHEELEKLEREAETLKEQVCSLFVGSKDPLHKLELIDLFNKLGISHFFEEEVRKSLEEMQHAESLMMMEDDLYSAALYFRILRQHGYHVSQEAISRLLDDKENSWDEKTRVEILEASYLVAYDEDMLDKKFSGVDVSSATSVNSNCPSHRSVSWFNVSSHMHANSNPIILRLAGLSFKAMQLHHHKDLNHILRWWRKVEALEFVKFSRDRVVESFLSAVGVAHEPQHTSLRKWLTKAICMVVIIDDVYDIYGSIHELQQLTDAIERQVLNKLVVLITAEPSFA

>OsaTPS22-scaffold6103.g77439.t1

MGTERRSANYKPSIWRDRYLQSLNSCYGEERFLLQAERLKNEVKILLKKTKNPMDQIDVIDAVQRLGIFYHFTDHIDIMFKNIHALDDDWIHTHNLYATSLRFRLLRQHGYNISPEVFRKFMDEKTGSFKPAICCDPKGLLSLYEASYLSVEGESNVMEEARVFTTHHLKQVVNQEDIDPNLAKEIQHALEVPLHWRVLRLESRWFIDAYKRRRGSNPLLLELAKLDFNITQKMYQDELKELSRQKWYTKSRLAEKLSFARDRLVECFLWTVGFTFQPNFKYCRVMLVKFAVLITIIDDIYDVYGTLDEVQLFTDAVERWDIKALEELPEYMRPCFLALFNTVNEMAYDVLRDQGFNIIPHSTQRWAELCNKYLVEAQWYHSGYKPSLEEFLTNGWVSSTGPLLIMHAHFCMTNPLKDEALDDMHTHPEILKWTSLVSRLADDLGTSTEELKRGDNPKSIQCYMHDTGCSEEDSRHYIKELIASTWNKINKDILMNRRYSRDFITTSINFARISQCMYQYGDGHGVPDRESKALILSLVIEPILLS

>OsaTPS23-scaffold5960.g76947.t1

MESRRSANLGASIWDDHFIQSLTSSYTGEKHAEKAEKLKAEVRTMMEQTTDELKQLELIDNLQRLGICNHFQDLIQKILQKMYEEERYGDRQKNLHITALRFRLLRQDGYHVPPDVFSSFMDKTGDFQEHLSKDITGLVSLYEASYLSMEGETILDLAKDFSSHHLHKIVEETKEKRVADQIIHALELPLHWRVQRLEARWFIQYYECGSDANPSLVELAKLDYNMVQATYQEELKRLSRWYEETGLSEKMSFARHRLAECFLWSMGFVPERHFGYARENLTKIAEFITILDDIYDVYGTLEELQVLTEIMERWDINLLDRLPEYMRICFLAVFNATNELAYHILRDQGINVISNLRRLWAELSRSYYKEATWFHSGYIPATEEYLNTAYISISGPLIHFYSYITTTNPINKHELQSLEKHAYWPSMVLRLADDLGTSSEARQHVKRLIDAAWKKMNKGILIEKPFESFSSIAMNLGRIALCFYHDGDGFGLPNSKTKKKLVSLVVHPIHMP

>OsaTPS24-scaffold581.g22628.t1

MGKIPNPKQAINRAVVLGNYASSPACMCLKLRRSGMYKPTLWDFHRLQSINTVYTDEEYKTRAGNLVVQVKKILEEESDWLRQLKLIDDLQRLGVSYHFDNEIDRILNSIFLEKKFCKERDLYSRALAFRLLRQHRLKVSQDVFDCFKGDFDENVEALLELYEASFLLMEGEDTLEAARVFSTDVLQKKLDDHENGRRLIDDYICTLVRHSLELPLHWRVQRPNARWFMDAYVNRSDFNSTVLDLAKLDFNIVQAAHQQELKQVSRWWKESGLGEKLPFARDRVVENYLWNVGLIFQPEYGDARVMTAKLFVLITVIDDVFDVYGTLEETQLFNNAITRWDIEAIHQLPHYMQICYLALDGFINEMAYHVLKEHGLVWADLCTSYAKEAEWYHTGYTPTMEEYMSNAWISISAHLILTHVFFLITNPIEEEAAASLRNYHNIIRCSALILRLADDLGTSPHEMKRGDVPKAVECYMNETGGSMEEGRQHVKFMLREVWKEMNREIFAGESTPFALNFIRSAADLGRQAQFMYLRGDGHGIRNPHIEQSISTLLFQPIL

>OsaTPS25-scaffold1263.g37014.t1

MHAIVSRIPTHMKPANCRYRTFYGGAQRVGSFIAASGGRACSAKPFTQNPTTRRSGNYKPSHWDFNYIQKRRAAELIAQVKMLLQRQMEAVPLLELIDDLQRLGISYHFLDEINQVLSCIYLDNFYAEERDLYSTALAFRLLRDHGFQVPQGSTTPLVLQNVSELHACISAPCSGDADSLENSSLFIEAYGENPQINPTLLQLAKLDFNILQAIHQQELTLLSRWWKDTGLAKMLPFVRDRTVENYFWTLGRLFQPEYGYSRMMATKYEMERGDVPKSVELYMKEAGASREEAREHVEHMIWETWKKLNEERVRDCEFSKDFMTSAVDLGRMAQYMYQNGDGHGIQNPQIKNEFAL

>OsaTPS26-scaffold77.g5351.t1

MYSLVNICVPCAFHGGAPKLQRIAAAAAAATPSNSTPQARRSANYKPPFWDFNYIQSFTTQFTEERYSTQAAELILEVKMLLQQQMEVVPRLELIDDLQRQDVFERYKNEKGAFRPSLGNDTRGVLQLYEASFLLTHGEQTLELANEFATNILRKRLVDDNNLVISHALELPLHWRIQRPNARGFIEAYGRRPQMNPIVYDLAKLDFNIVQAMHQQELKLISRWWKQTRISEKLPFARDRVVECYLWTLALFPPEYEYANARIMATKANILITIIDDIFDVYATLEELQLFNTVIQRWDVGAMDHDQLPNYMQICFLTLNNFVNEMAYDVLKDKDILIIQHLRKSWSDLFKSFIQEAQWQAAGQAPTLKQYLSNGWISSSVPVMLSHTFFLITNPIHKEAVESLYKYHDLVQFPAMLLRLANDLATSQNEMERGDVAGCVECYMKETGGSRDEGGQHVRFMMWETWKSMNQERVGDCEFCKDFIRSAVDLGRMAQYMYQLGDGHGIQNPQIKDRISCLLFEPIR

>OsaTPS27-scaffold581.g22632.t1

MSGLIMQVASVKPRNYCKFQLMVSASKLQRGVRSLRPRCSSQLIHNQTQRRSGGYHPTLWDFNTIHSLNTQYKEESRLRRAGSLIEQVKMLLMDEEMDGVRRLELIHDLHCMGISYHFHKEISQILHSTYLEYNKTDQQRDLYSTSLGFRLLRQHDFHVPQEVFDCFKNETGVFKPSLGEDIKGVLQLYEASFLLTQGEDTLELAREFATNILQRQVNDHQIDNSLLSSIRAALKLPSHWRIQIPNARSFIDDYKWRPDMNQVVLELAKLDINIVQAQYQEDLKEATRWWKNTCLVEHLPFVRDRIVECYLWSIGILHPRHRRYERIMLARVIALISAIDDVFDVYGTLEELQLFTDAVRRWDDIESIKQLPKYMQVCYLALYNFVNEMAYDTLKEKGFNSAPYLRKAWGGLIESYKKEANWYHNGHKPSLEEYLENAWISAGACPALSHIYFWVADPVEEEVAQTMHKYHHLVRASSMLLRLADDMGTSLDEVERGDVPKSIQCYMNDKNASEEEARAHMRSLIAQTWKRINAEMVSENSPFSKEVIEAAANIGRMGQFMYQDEQDGFGMQHSKVHKLLRGLLFEHYA

>OsaTPS28-scaffold1797.g45336.t1

MQVLPITKHFHNSSAVRRRASSATPPRCSLQTERRRSGGYQPALWDFDSIQSLKTTNCEDERQLTKRADLIGQVRKMVVEGGVSDVARLELIDELHRLGISCHFEDEIIQILNSNHKYTNLETDLYSKSLRFRLLRQYGVPVSQEVFDCFKNDKGTNFKATLGHDIKGLLQLYEASFLQTQGEETLELAKEFATNLLHKKLEESDHEIDDDLLSSIRGALEFPTHWRVQMPNARSCIHAYKRRPDMNPTVLELAKVDINIAQAQFQQELQETSRWWESTRLAQQLPFARDRIVECYFWTTGVLRRREHRYERIMLTKINALVTTIDDVFDIYGTLEELHLFTTAMLRWGDIESMMQLPPYMQLCFLALHDFVIEMGYDILKEKGFNSIPYTQKTWVDLIESYLIEAKWYYNGYKPSKEEYITNAWISIGGVPILSHIFFRLTDSINKEDVDRMHEYHDAVRASCTILRLADDMGTSLDEVERGDVPKYIQCYMNEKKCGEDDARKHARSVIEETWKTMNTEIMSIDSPFSKHFVEAAANLGRMAQFIYQDGLDGFGMQHSKVNKLLRGLLFEPYA

>OsaTPS29-scaffold3344.g62122.t1

MSTISMHVAILNKPAPNFLHNLNNKSSNPQRVPSTRRRPSCSLQLDVETRRSGNYQPSAWDFNYVQSLSNHYYKEERHLERKGKLIVEVKMLLEQEMAAVQHLELIEDLQNLGLLYLFQDEIKIILNSIYNHHKCFHNNEQGEKENADLYFTSLGFRLLRQHGFKVSQGALLIKILDSNHFMILSIDIHFIIAEVFDCFKNEEGTDFKASLGDDTKGLLQLYEASFLVREGEDTLEMGRQFSTKILQKKVDLAELIEEENLLSWIRHSLELPLHWRIQRLEARWFLDAYATRPDMNPIIFELAKLEFNVFQGVQQEELKDLSSWWNNTGIAEKLPFARDRIVEGHYWAIGTLEPCQYKYQRSLIAKIIALATVVDDVYDVYGTLDELQLFTDAIRRWDIESINQLPNYMQLCYLAIYNFVSELAYDIFRDKGFNSLPYLHKSWLDLVEAYFVEAKWFHSGYTPTLQEYLNNSKITITCPAIVSQIYFTFAHSIDKSVIESVYKYHDILYLSGMLLRLPDDLGTAPLEMKRGDVAKAIQCYMKEHNAPEKEAREHVRFLMREAWKQMNTAAAADDCPFVDDFVVGAASLGRVANFVYVEGDGFGVQHPKIHQQMAEILFYPYH

>OsaTPS30-scaffold3344.g62123.t1

MSTISMHVAILNKPAPNFLHNLNNKSSNPQRVPSTRRRPSCSLQLDVETRRSGNYQPSAWDFNYVQSLSNHYYKEERHLERKGKLIVEVKMLLEQEMAAVQHLELIEDLQNLGLLYLFQDEIKIILNSIYNHHKCFHNNEQGEKENADLYFTSLGFRLLRQHGFKVSQGALLIKILDSNHFMILSIDIHFIIAEVFDCFKNEEGTDFKASLGDDTKGLLQLYEASFLVREGEDTLEMGRQFSTKILQKKVDLAELIEEENLLSWIRHSLELPLHWRIQRLEARWFLDAYATRPDMNPIIFELAKLEFNVFQGVQQEELKDLSSWWNNTGIAEKLPFARDRIVEGHYWAIGTLEPCQYKYQRSLIAKIIALATVVDDVYDVYGTLDELQLFTDAIRRWDIESINQLPNYMQLCYLAIYNFVSELAYDIFRDKGFNSLPYLHKSCTQNVLVGLIPINLVAKKLKFLWTNYWQWLDLVEAYFVEAKWFHSGYTPTLQEYLNNSKITITCPAIVSQIYFTFAHSIDKSVIESVYKYHDILYLSGMLLRLPDDLGTAPLEMKRGDVAKAIQCYMKEHNAPEKEAREHVRFLMREAWKQMNTAAAADDCPFVDDFVVGAASLGRVANFVYVEGDGFGVQHPKIHQQMAEILFYPYH

>OsaTPS31-scaffold3957.g66769.t1

MSITSSSMHAGIFFSYLHKLAPAPPHLVCCGGGLRRRPSSSLQLQPAQTRRRSGNYEPSAWDFSYLQSLNNHKEERCLRRQAELIEQDQIKHVLSIIYNQHSCFHNNEAATGEEASLYFTAIGFRLLREHGFEVSQEVFDHFKNEKGTDFRQDLVDDKDCCNFTRHLGEDTFELLDSVEAYFQEAKWYHSGYTPSLEQYLNNAKISISCPALLSQIYFTLASSIDKPEIESMYKYHHILHLSGMLLRLPDDLGTASHELGRGDVAKDMKERNVCEEEAKGHVRFLNREAWKQMNTARAADDCPFTHDFVVAAANLGRVANFMYVDGDGFGLQHSAIHQHMAELLFHPYAS

>OsaTPS32-scaffold4479.g70152.t1

MYSISMHFGILNKPTPNFLHKRPSMPPRVSSLSAASTTTRCRTSCSLQLDAKPTDNTRRSGNYQPSAWDFSYIQSLNNHHYKEERYLTRQAELIELVKMMLEDEMETVEQLELIDDLKNMGLSHFFNHQIKQILNTIYNEHKRFHSNEGVEEKDLYFVALGFRLLRQHGFTVSQEVFDCFKNDKGTDFEPNLGEDTKGVLQLYEASFLWREGEDTLELGREFATKILQKKVEEGGDDNLLSSIHHALAIPLHWRIQRLEARWFLDAYASRPHKNPIIFQLAKIEFNIVQATFQEELKDVSRWWNSLGFVEKLPFARDRIVESYFWAVGTFEPLQYEYQRKQVAKIIALVTVIDDIFDVYGTVDELQLFIDTIRRWDTESINQLPYYMQLCYLAVYNFASETAYTILRDKGFMCISYLHRSFLDLVEAYVEEAKWYHSGYTPTLEEYLKNASISVTCPAVISQIYFTLVNSIDKPVIESMYKYHEILVLSGLLLRLPDDIGTAWITMRLHVHE

>OsaTPS33-scaffold6266.g77960.t1

MFTINMQVAILAKPANYYLYNFGTNRWRVSSTSLATHLRASCSLQLDIKPADDEARRSGNYQPSAWDFNYLQSLNNHHYKEERHLERKGKLIAEVKMLLEQEMAAVQHLELIEDLQNLGLLYLFQDEIKIILNFIYNHHKCFHSNGGGAEENADLYFVALGFRLLRQDGFEVSQGVFDCFKNEEGSDFKPNLAKDTRGMLQLYEASFLVREGEDTLEMGRQFSTKILQKKVETELIDENLLSWMCHSLELPLHWRIQRLEARWFLDAYATRPDMNPIVFELAKLDFNIVQATQQEELKHVSRWWNNIGLAKKLPFVRDRVVEAYFWAVGFIEPHQYGYQRLVIAKMVALVTTIDDVYDVYGTLHELELFTDIIKRWDTESINQLPYYMQLCYLALYNFVSELAYDILKDRGFNSIPYLHKSWVDLVEGFLEEAKWYYSGHTPSLEEYLKNASITIAAPAAISQLYFTLANTIQKPVIENMYKYHDILHLSGLILRLADDLGTAPFEVERGDVAKAVQCYMKERNGSEKEAQQHVRFLIGEAWKEMNTAMGAEDCPFTSDLVAVAANIGRVAQFMYNGGDGYGVQHSKIHQHMAELLFQPYV

>OsaTPS34-scaffold8396.g82581.t1

MYNMSINMHVGLIPNKPAHDNYLHKNASRQRHVSSLAATRPSPSCSFQQDHQTRRVGNYQPSTWDFDYIESLNNQYKEEKYLSMQAELIEQVKMLLEEDMEAVQQLELIDDLDNLGLSYFFKDKIKHNLNIIYNHTKVFRMNEAEEEMDLYLAALGFRLLRKHGFNVSQGIFDCFKNEEGSDFKPRLAEDTKALLQLYEASFLLREGEDTLELAREFAAKYLQKKLDEGGDEIDDINLFRSIKHSMDLPLHWRIQRYEARWMLDAYARRPDMNPVIFELAKLDFNIIQATQEEELKYVARWWNSSCLAEKLPFVRDRIVECYFWALGLFEPHQNGYQRKMAAKIITFVTVIDDVYDVYGTLDELQLFTDTIRRFAWLSLVEAFFKEAKWYYSGYTPTLEEYLNNSKISISSPTIISQVYFTLQNSSTDKTDIESLYKYHHILYLSGMILRLADDLGTTQFEMKRGDVAKAMQCYMKETNASEEAAQEHVRFLIRKAWKEMNTAMAAAECPFSHDLVEAAANLGRAAQFIYLEGDGHGVQHSKIHQQMADLLFYPYV

>OsaTPS35-Scaffold3784.g65511.t1

MSLASTLPFRPSSASHGAAFCAPPPPRSSAGHSSLSLSHAHTLLHARTRENGCLPLFRPLTPTSSLQCTALSRPRTEDYIDVIQNGLPVIKWHEIVENDAEKDSLKETTSDKIRELANVIRSMLQSMGDGEISISPYDTAWAALVEAGGGGRRRPQFPSSLDWISKNQFPDGSWGDKTFSIYDRIINTLACVVALRSWNIHPDKTDKGILFIRKNMIRIDDENLEHMPIGFEVALPSLIEKARKFGIDIPNDTKGLREIYARREIKLKKIPREILHQVPTTLLHSLEGMSGLTWKKLLKLQSEDGSFLFSPSSTAFALQQTRDDNCLNYLTKHIHKFNGGVPNAYPVDLFEHLWAVDRLQRLGVSRYFKPEIEECIAYVHRYWTDKGICWARNSQVKDIDDTAMGFRLLRLHGYEVCADVFENFKSGGEFFCFQGQSTQAVTGMYNLYRASQLMFPGENILEDAAHFSANFLQLKRANNHLFDKWIITKDLPGEVGYALDVPWYASLPRVETRFYLEQYGGDDDVWIGKTLYRMPYVNNNKYLEMAKLDYNNCQALHQKEWRNIQKWYRSCNLGEFGLSERNLLQAYYIAAASVFEPERSQERLAWAKTQILMETIMLHFDFQQLSREQRRAFLDEFQHGSMLKYTNGGRYKTRSSLVGTLVRTLNQLSLDILLAHGRDIHHPLKNAWCklkKWLKSCGEGGDAELLVATLNLSGGGRRHTWPLESSNPKYEELLKATIGVCDKLRMFQRRKEHDGNGCFSIAGGITTTEIELEMKELVKLVLTENLHSEINQNFLIVAKSFYYAAYCNPGSINFHIAKVLFERVL

>OsaTPS36-Scaffold3016.g59254.t1

MSSVSTITSLFRSTATASGLSLSLSLSLSLSCECVFKAELQENMMQVSFQDMYNSVRGQATDTLEVVVSEKIRQLREKIEWMLQNMDDGEISVSPYDTAWVAMVEDINGSGHPQFPASLRWISDNQLADGSWGDRKFVVYDRILNTLACVVALTAWKKHPHKCNKGLTFIRNNIEKLGHENEELMPIGFEVVFPSLIEKAQKLGIEIPNIDSPCIQKIQAMRDYKLRKIPMELLHKKPTSLLHSLEGMQGLVWQKLLDFRSDGSFLCSPSSTAYALHHTKDELCLNYLLKAVKKFDGGVPNVYPVDMFEHLWCVDRLQRLGICRYFRIQIKESLDYVYKYWTDKGICWARNTNVQDIDDTAMGFRLLRMHGYDVSTDVFEQFEKAGEFCSFPGQSTHAITGMYNLYRTSQVMFHGEHILEDARNYSANFLHKRRLANELVDKWIITKDLPGEVGYALDVPFFASLPRLEARFFIEQMPYVNCDAYLELAKLDYNHCQTVHQLEWKSIQKWYKDCNIGEFGLSERSLLLTYYLAASTAFEPERKGERLAWVKTAVLVEAIMCQQLSQEQKREFVDEFENCSILKIENGGRHKTRIRLAEILISTVSQLSFEILVAQGRDIKQQLSNKWQKWLKTWEEGGDLGEAEAQLLLHTLHLSSGLDESSFSHQKYHELMEATSKVCHQLRLFKNRKVREQAIHFSSKHLGSSQVDDAQGCVSDLVIGTTFQIEANMKKLVKLAFTKSSRDLDSITKQSFFAIARSFYYTAYCDAGAINAHIYKVLFQKID

>OsaTPS37-Scaffold2041.g48588.t1

MYNRSQHQHIQIQLIFFPFSIQRKFNLEIMNSLYHLQQSPVSHRRILPSSSTAAKLHPPQYISISACMNNNKNLNYKLSHKKISKAPEIRVGIFVSWVLTVVIAVAMVDAPQVHDKDDATAKQGHDAVNDIEDPIEYIRTLLKTTGDGRISVSPYDTAWVAMIKDVKGSDAPEFPSSLDWIRRNQLSDGSWGDEKFFCVYDRLVNTIACVVALKSWNLHADKVERGVRYIKDNVDKLRDGNEEHMTCGFEVVFPALLQRAKNLGIQDLPYDAPVIQEIYHTREQKLKRIPMEMMHKVPTSLLFSLEGLENLDWDKLLKLQSADGSFLTSPSSTAFAFMQTKDKKCYQFIKNTIDTFNGGAPHTYPVDVFGRLWAIDRLQRLGISRFFQSEILECLDHIHKFWTDKGVFSGRESEFCDIDDTSMGVRLLRMHGYDVDPNVLRNFKKDEKFSCYGGQMIESPSPIYNLYRASQLRFPGEEILEDASKFAYEFLQEKLSNNQILDKWIKLGLEMPWYATLPRVEASYYIHYYAGSGDVWIGKTLYRMPEISNDTYHELAKTDFKRCQAQHQFEWIYMQEWYESCNIEAFGISRKELLVAYFLATASIFELEKTKERIAWAKSQIISKMIKSLFNNESTSSQEKLQFLSDFRNSNGLNNTNMVLATLTQFLEGFDRYTSHQLKNAWSVWLTKLQQGDGDGGPDAELLTNTLNVCAGHIAFREEILLHNEYKTLSNLTGKICQQLSQIHNEKEMESEGEITAKTSIKNRELEQDMQTLVKLVLEKSSGGINRDVKKTFLAVAKTYYYRAYHDAQTVDTHMFKVLFEPVG

>OsaTPS38-scaffold7905.g81814.t1

MDDLITCIKNLLNTIEDGRISVSAYSTSWVALIKNIDGHDIPQFPSSLDWIVQNQLPDGSWGDAHFFCIYDRLVNTIACVIALHTWKVHAHKIKKGVSFVKENIWKLEDACAAHMTSGFEVIFPTLLRRARDLGIPGLPCDDIPAIRKIYAARDHKMKNVSAYNTSWVALIKNIDGRDMPQFPSSLDWIVQNQLPDGSWGDAHFFCIYDRLINTIACVIALHTWKVHADKIEKGVSFVKENIWKLEDACDAHMTCGFEVIFPNLLKRARDLGIPGLPCDDIPAIRKIYAARDHKMKKIPREVMHQVTTTLLYSLEGLDDLDWSRLLKLQSADGSFLTSPSSTAFAFMQTNDHNCLKFITSVVQTFNGGAPDTYPVDMYARLWAVDRYWNANGIFSARDINFSDLDDTSMGFRLLRLQGFDVDPNVLTNFKEGDRFCCHIGEVTRSTSPTYALYRASQIQFPGEEILEEAYHFSRKFLQDWLASDQLLDKWILSKDFPNEIKVGLEVPWYASLPRVEAAYYMQHHYGASTDVWIAKTAYRMPDVNNDEYLELAKLDFKRCQDQHQIELSYMQGWYENHKVEEFGISRKDLVVAYFLAAATVFEPERAKERIVWAKTELICGMIKAFFVGESMCSLEEKRAFLQEFRSSITGSNIVMKSESRVINVLLEAIGELLGGFDESIVRQLENTWCTWLMRLDRGDWATAELLVSTLNICTRRVSQHDHIFLNQDYKTLSNLVNKICHHLSQIPNQKVEEMRMCGRSRAKDKMNEMREDMKALTKLVLEESSNLHKNTKQAFLAVAKTYYYRAYFDADTVDLHIFKVLFEPVV

>OsaTPS39-Scaffold3756.g65332.t1

MHILPTRPITFSFGFGYGSISSATRCRLSSKHCTCCWVAGYGSQEGKSGSRLENASPDSYLLSLLGVAQKERGISIIDPKRTWKNRRQDNNRISCKDVITNASRRGTPQVCAGNGGFEPQLFNEKSEIEECIEIIRKTLQSFDDGQISASAYDTAWVALIRDVMNKDLPQFPSSLDWISSNQLSDGSWGDHKIFVPSDRLLCTLACVVALRAWNAHPNESERGILFVKENISKLEDAVNGDGQNTTFGFEILFPAFLQRARKLGIEGLPYHHPTAQKICAARDRKLERISKEVKEEVPASLLLILEGLMENIKWENIMKMQSADGSFLGSPATSAFVFMETEDESCLRYLKNITEKFGGGAPSLYPVDILTRVLAVDRLQQLGISGFFHSEIQDCLTYIYRFWTGNGVFSERNSTFCDVDVTSMAFRLLRLHGYNVSSDVFTNFKKDDKFLVYGDNKSVDSPSTILNLYKASLVRFAGETILEQANEFCHNFLDNCLTSKHLHTEPKYELETPWFASLPRLVARFNIEHYNNDKYWMGKSLYRLSDTNEETYLKLAKLDYNRCQEQHQMEWKEMQQWYEDCNLKELGISKNEILEAHFLAVASIYEVERSGERVAWVKSQILSQILSTYYFIKESDQHDEGQFSAPFNTKADQCGKGFTNVQRIITILFEALTQIKKTARERGSGDITDDLLHEAWGGWLKKLGEEGTKGRQEAELIARIINICGGHILSKEILSHHEYKTLSQLNNRICNQLRNQKMISKGIENEMEVLVQLVLQESSNGISKAIKQTFLVVAKTFYYRAYFSPLQIENHVAMVLLEQVV

>OsaTPS40-scaffold543.g21653.t1

MSLPLSTRVLFVPKPSQFRSSSFSHASASLDIGLQRATSPPQSASVSTSFEETKDRIAKLFHKDELSVSTYDTAWVAMVPSPTSSEEPCFPDCLNWLLENQCHDGSWARPHHHPMLIKDVLSSTLACILALKKWGVGEEQINRGLHFIELNFASATEKSQITPVGFDIVFLAMLDYARDLSLNMRLDPTTLNDLICKRDLELKRCNQSNSTEREVYWAYIAEGMGKLQNWGSVIKYQRKNGSLFSCPSTTAAAFIALRNTDCLNYLRSALKKFGSAVPAVYPLDIYSQLCKVDNLERLGISRYFSNEFQSVLDETYRSWLQGDEEIFMDAATCALAFRTLRMNGYNVTSDPLTKILQECFPSSFHGNMTDINTTLEIYRASEHIIYEDERDLEKHNLRLKTFLEQELSSGGFVQSCQLGRNIDAEVNQALDYPFYAIMDRMAKRKNIELYNSDNTRILKTSYCSPNFGNKDFLHMSVEDFNRCQDIHREELKELERWVIENRLDELKFARSKSAYCYFSAAGTIFSPELSDARMSWAKNGVLTTVVDDFFDVGGSMEELKNLIHLVELWDVDVSTKCTSPNVQIIFSALKHTICEIGDKGFKLQGRTAMVARHLKQTNNEIRHVQQLTVLGWLDLLYSMMKENEWSRDKFVPTIDEYISNAYVSFALGPIVLPALYLVGPKLSEEMVNHSEYHNLFKLMSTCGRLLNDIRSYERELKDGKLNALSLYMMNNGGEVTMEAAVSEVRSWIERQRRELLRTVLGKTVVPEACKELFWHMCTVVHMFYSKDDGFTSQDLLGVVNAIIKEPLVINDS

>OsaTPS41-Scaffold702.g25723.t1

MQEHSNCHSLNIIYPLTPFSTTSHLSEIKYSTRRQPLLPPPPFLAAVETDMSLTINLRFIPFSGHRVQCRRRIFPVVESPKITSKSSVAVKCSLATPTDLMVKIEEKFKGNSLAAMNIDSSDIPANLCIIDTLQRLGVDRYFQSEIDAILDDTYRLWKLKRKDIFSGITTHAMAFRLLRVKGYDVSSEELAPYADQERVDLTRTDVSTVVELYRAAQERVDEEDITLEKLYVWTSAFLKQQMMNGAITDQKLHKQVEYYLKNYHGILDRMGVRRGLDLYDVGHYKALKAADRFSNRWNEDFLAFARQDFNISQAQHQKELQQLQRWYSDCKLDTLKYGRDVQGVANFLTSAMTGDPELSDVRLAFAKHIVLVTRIDDFFDHGGSREESYKILELLKEWKERPSGEYGSEEVEILFKAVYDTVNELAEMAFVEQGRSVKDLYIQLWVEIISIFKIELDTWSDDTALTLDEYLSSSWVSIGCRICILVSMQFIGLKLTDEMLRSEECLDMCRHVSMVDRLLNDVQTFEKERKENTGNSVSLLLKAHKDEGGISEEEAIEKIKDLAEYNRRKLMRIVYKKGTIFPRKCKDMFLKVCRIGCYLYKSGDEFTTPQQMMEDMKSLVYEPLTIHSPSQANNVEKMSSAL

>OsaTPS42-scaffold930.g30815.t1

MVNSIESLVSEVKKEMIWEKRDAFVSPSAYDTAWLAMIPHQHNDNRPMFECCVKWILQNQKEGGFWGETDEEDLPTIDALPATLACIAALKFWNLGHQNIESGLGFIRSKAEILLKINYQNLPRWFVLTFPAMIELAEAVGLHLVFPQGLITPIADIFITRHHILQAEELVENEEWRYFPPLLSYLETLPSTYEFDRQQTVNRYLSCDGSLFQSPSATAQAFMSTGNIHSLQYLQSLVQKFPKGVPAKYPVDGDLIKLSIVDHVQGLGLAWHFSEEIEQLLAQVYRNQKNEKSNPSGTNTVSLKLYKDALAFRLLRMHGHNVHPGSFCWFLHQPETVSYMEENREHFISAMYSVYRATDLSFPGENEMEEARNFSTKFLENPKTRHRDHNFLITKGLQNMMKYELDVPWSARLDRLYHRKWIEENKISPLWIGKASFYRLSCLDNKKLMQLAVDNFEFQQSIYIQELEELKGWSKKWRLSEMGFGREKTVYTYFAVASCSCLPHNSIMRLVIAKAAIIVTVADDFYDMEGSLTDLEILTNAVHKWDGEGLEGHSETIFEALDGFVNDIVAKCHPQQASVVLPKLQNLWRESFAAWMLERRWSITGHLASMDEYLETGMASIAAHTIALPATTFLSQTGPHGQYQTITILLMAITRLANDMQSYQKEAAEGKMNMVLLHRQKHQNSSIEESVGHVKQILELKRKEFMQHVFMGEDMPESCKKIHLYCMKVFEMFFNSGNLFDSETALLDDIKKSIYLPIPEQHRLLNVPSLNNNRPKVSARFHAVLKPPQATSLTIKSSVAQYSTKPHLPITFNMTAPFSKNMPFIGLPL

>OsaTPS43-scaffold558.g22102.t1

MEEMKQVLLQHSNLEEKNNRNDSLILVDGIQRLGLDHHFDEEIDMILGKLSNHNFDSVEHDLYDVSLHFRLLRNHGYHVSSDVFNNFKGNGGKFEAELRQDIQGLMELHEAAQLGFTDEDIIDEAENFSRVNLNKCLEDTKVDDYHKKVIKNTLRHPQHKNIARLTARNCMNGGLVKGCSNKRWGKTLTELAKMDASMGELMHQEELLQVSKWWESVGIAEGLSGARSQPVKWYIWSMAMLIDSPSLRVERIELAKVIAFVYVIDDIFDLYGTTDELSLFTQAINRWEYSASDTLPEYMRKSYKGLLDTTNGIAQKVEDNYGTNPIDSFKVAWISLCSAFMLEARWFRSSELPRAKEYLENGQVSTGAHVILVHLFFLLGHGRGWSSLHLKDTSTLLSSVATICRLSDDLGTAQVFSSSETNIKRP

>OsaTPS44-scaffold1569.g42016.t1

MEVYRSLDVTPLTTDETLFEKVEQVRDVLESVEQDSLESLELIDTIQRLGLCYYFHDEIQALLRHQISNDSDDLYQASLRFRLLRQQGFQVLADDFFRKFLSDDDGGGLKFQGELADETRGLMAMFEASHLNTGGEEILYGAAIFSSRHLNDMIMRCIDEEQVKMAKHCLVYSQHKSLARLTARIYLRFLDGKHPWENLLRDLAELEFVSTELLHKQEILQVARWWRGTRLGNELKSSRNQPLKWHMWSTATLIRSADWSKHRLLLTKSISLVYVVDDIFDLYGEIHELSLFTEAVIRWDASVTEKLPSYMKTCFNAIYDTTNEVSSFVSEEYGWNPIHFLIKEWGSLMEAFLIEGKWFRSVECAKSDEYLKNGVISSGVPMVLSHLFCLMGDPLTVQNQTLLNDPHGLTHSVAKLLRLLDDLGSAQDEQQEGYDGSYVECCMREGRVESLEGAREHVMGMVWETWENINKLGLCSTSPFSASFRHACLNAARMVPTMYSYDQNHRLPLLKHYVKSMFHDATLAKSILG

>OsaTPS45-scaffold774.g27420.t1

MSCARIARALPYSSAKTSIPWQSGHCPTAVRLPRFPACTATPTSPPLINRDNSLLKNTRQHVLFPWVEKEDSSKRREYLLEKTAQKLQRKDTEAVEKLKLIDNIQRLGIGYYFGDAINAILRSSFSTEEEEEDLFTAALRFRLLRHNGIQIGPEIFLKFKDERGKFDGLDTLGLLSLYEASHLGVEGEYILEHAMEFAEPRLRRSLLELAAPLHGEVAQALDVPRHLRMARLEARLFIEQYGKQSEHDGDLLELAILDYNHVQAQHQSELTEINRWWKQLGLVDKLSFGRDRPLECFLWTVGLLPEPNYSSIRIELAKTISILLVIDDIFDTYGEMDELILFTHAIQRWDLEAMEDLPEYMKICYMALYNTTNEICYKVLKDTGRMALPNLKSTWIDMIEGFMVEAKWFNGGSAPKLNEYMENGVSTAGAYMAFAHIFFLIGEGVTHQNSQLFTQKPYPKLFSTAGRILRLWDDLGTAKMDSKDIIYHLRSKGVLTLIKHSRGAGARRSGIKRAVIYERQVNGRGGEKSHFGRNKTLMEGSEWGTRLQQEYAIIHNPSRT

>OsaTPS46-scaffold774.g27428.t1

MSCGRMTIALPKCSAKASTPRRRRHCPTAVHHPHFPARAASSAPPTFNRDAYPITPHVEDSSRRREYLLEETARQLQRNDTETVEKLKLIDNIQRLGIGYYFEDAINAILRSPFSTEEEEDVFTAALRFRLLRHNGIQVSPEIFLKLKDERGKFDVWDTLGLLSLFEASNLGAKGEEILEEAMEFAELRLRRSLPGMAAPLHGEVAQALDVPRHLRMARLEARRFIEQYGKQSDHDGNLLELAILDYNQVQAQHQMELTEITRWWKQLGLVDKLSFGRDRPLECFLWTVGLLPEPKYSSIRIELAKAISILLVIDDIFDTYGETDDLILFTNAIRRWDLEAMEGLPEYMKICYMALYNTINETCYKVLKDNGWTALPYLKSTWIDMIEGLMVEAKWFNGGSAPKLEEYIENGVSTAGAYMAFAHIFFLIGEGITHQNSQLFTQKPYPKLFSAAGRILRLWDDLGTAK

>OsaTPS47-scaffold774.g27424.t1

MATEGEEILEEAMEFTEARLRRSVAEAAPPLRDEVAAALEAPRHLRMERLEARRFIEHYGKQSDHNRDLLELAILDYNHVQAQHQSELAEIIRWWKQLGLVEKLGFGRDRALECFLWTVGLLPHPKYSSCRIESAKAAAILYVIDDIFDTYGKLDQLILFTHAIRRWDLEAMEDLPEYMKICYMALYNTTNEICYRVLKDSGRVALPYLKSVWIETIEAYMVEVKWFNGGSAPKLEEYIENGVSTAGAYMAFAHLFFLIGEGLTHQNIMLFRQKPYPKLFSAAGRILRLWDDLGTSQEEEERGDAASCIQLFMKEYNFATVEEARNRVSEEISRLWKDLNRGLTSSNNGLPLAIVKVALNMARASQVVYKHHGHTYMSSVDNYVEALFFTPLFSS

>AB|O81086

MAGVSAVSKVSSLVCDLSSTSGLIRRTANPHPNVWGYDLVHSLKSPYIDSSYRERAEVLVSEIKAMLNPAITGDGESMITPSAYDTAWVARVPAIDGSARPQFPQTVDWILKNQLKDGSWGIQSHFLLSDRLLATLSCVLVLLKWNVGDLQVEQGIEFIKSNLELVKDETDQDSLVTDFEIIFPSLLREAQSLRLGLPYDLPYIHLLQTKRQERLAKLSREEIYAVPSPLLYSLEGIQDIVEWERIMEVQSQDGSFLSSPASTACVFMHTGDAKCLEFLNSVMIKFGNFVPCLYPVDLLERLLIVDNIVRLGIYRHFEKEIKEALDYVYRHWNERGIGWGRLNPIADLETTALGFRLLRLHRYNVSPAIFDNFKDANGKFICSTGQFNKDVASMLNLYRASQLAFPGENILDEAKSFATKYLREALEKSETSSAWNNKQNLSQEIKYALKTSWHASVPRVEAKRYCQVYRPDYARIAKCVYKLPYVNNEKFLELGKLDFNIIQSIHQEEMKNVTSWFRDSGLPLFTFARERPLEFYFLVAAGTYEPQYAKCRFLFTKVACLQTVLDDMYDTYGTLDELKLFTEAVRRWDLSFTENLPDYMKLCYQIYYDIVHEVAWEAEKEQGRELVSFFRKGWEDYLLGYYEEAEWLAAEYVPTLDEYIKNGITSIGQRILLLSGVLIMDGQLLSQEALEKVDYPGRRVLTELNSLISRLADDTKTYKAEKARGELASSIECYMKDHPECTEEEALDHIYSILEPAVKELTREFLKPDDVPFACKKMLFEETRVTMVIFKDGDGFGVSKLEVKDHIKECLIEPLPL

>AGO24474

MALVSISPLASKSCLRKSLISSIHEHKPPYRTIPNLGMRRRGKSVTPSMSISLATAAPDDGVQRRIGDYHSNIWDDDFIQSLSTPYGEPSYQERAERLIVEVKKIFNSMYLDDGRLMSSFNDLMQRLWIVDSVERLGIARHFKNEITSALDYVFRYWEENGIGCGRDSIVTDLNSTALGFRTLRLHGYTVSPEVLKAFQDQNGQFVCSPGQTEGEIRSVLNLYRASLIAFPGEKVMEEAEIFSTRYLKEALQKIPVSALSQEIKFVMEYGWHTNLPRLEARNYIDTLEKDTSAWLNKNAGKKLLELAKLEFNIFNSLQQKELQYLLRWWKESDLPKLTFARHRHVEFYTLASCIAIDPKHSAFRLGFAKMCHLVTVLDDIYDTFGTIDELELFTSAIKRWNSSEIEHLPEYMKCVYMVVFETVNELTREAEKTQGRNTLNYVRKAWEAYFDSYMEEAKWISNGYLPMFEEYHENGKVSSAYRVATLQPILTLNAWLPDYILKGIDFPSRFNDLASSFLRLRGDTRCYKADRDRGEEASCISCYMKDNPGSTEEDALNHINAMVNDIIKELNWELLRSNDNIPMLAKKHAFDITRALHHLYIYRDGFSVANKETKKLVMETLLESMLF

>AGO24475

MALVSTAPLASKSCLHKSLISSTHELKALSRTIPALGMSRRGKSITPSISMSSTTVVTDDGVRRRMGDFHSNLWDDDVIQSLPTAYEEKSYLERAEKLIGEVKNMFNSMSLEDGELMSPLNDLIQRLWIVDSLERLGIHRHFKDEIKSALDYVYSYWGENGIGCGRESVVTDLNSTALGLRTLRLHGYPVSSDVFKAFKGQNGQFSCSENIQTDEEIRGVLNLFRASLIAFPGEKIMDEAEIFSTKYLKEALQKIPVSSLSREIGDVLEYGWHTYLPRLEARNYIQVFGQDTENTKSYVKSKKLLELAKLEFNIFQSLQKRELESLVRWWKESGFPEMTFCRHRHVEYYTLASCIAFEPQHSGFRLGFAKTCHLITVLDDMYDTFGTVDELELFTATMKRWDPSSIDCLPEYMKGVYIAVYDTVNEMAREAEEAQGRDTLTYAREAWEAYIDSYMQEARWIATGYLPSFDEYYENGKVSCGHRISALQPILTMDIPFPDHILKEVDFPSKLNDLACAILRLRGDTRCYKADRARGEEASSISCYMKDNPGVSEEDALDHINAMISDVIKGLNWELLKPDINVPISAKKHAFDIARAFHYGYKYRDGYSVANVETKSLVTRTLLESVPL

>AGO64404

MAEISESSIPRRTGNHHGNVWDDDLIHSLNSPYGAPAYYELLQKLIQEIKHLLLTEMEMDDGDHDLIKRLQIVDTLECLGIDRHFEHEIQTAALDYVYRWWNEKGIGEGSRDSFSKDLNATALGFRALRLHRYNVSSGVLKNFKDENGKFFCNFTGEEGRGDKQVRSMLSLLRASEISFPGEKVMEEAKAFTREYLNQVLAGHGDVTDVDQSLLREVKYALEFPWHCSVPRWEARSFLEIYGHNHSWLKSNINQKMLKLAKLDFNILQCKHHKEIQFITRWWRDSGISQLNFYRKRHVEYYSWVVMCIFEPEFSESRIAFAKTAILCTVLDDLYDTHATLHEIKIMTEGVRRWDLSLTDDLPDYIKIAFQFFFNTVNELIVEIVKRQGRDMTTIVKDCWKRYIESYLQEAEWIATGHIPTFNEYIKNGMASSGMCILNLNPLLLLDKLLPDNILEQIHSPSKILDLLELTGRIADDLKDFEDEKERGEMASSLQCYMKENPESTVENALNHIKGILNRSLEEFNWEFMKQDSVPMCCKKFTFNIGRGLQFIYKYRDGLYISDKEVKDQIFKILVHQVPMEE

>AGO64405

MAQISESVSPSTDLKSTESSITSNRHGNMWEDDRIQSLNSPYGAPAYQERSEKLIEEIKLLFLSDMDDSCNDSDRDLIKRLEIVDTVECLGIDRHFQPEIKLALDYVYRCWNERGIGEGSRDSLKKDLNATALGFRALRLHRYNVSSGVLENFRDDNGQFFCGSTVEEEGAEAYNKHVRCMLSLSRASNILFPGEKVMEEAKAFTTNYLKKVLAGREATHVDESLLGEVKYALEFPWHCSVQRWEARSFIEIFGQIDSELKSNLSKKMLELAKLDFNILQCTHQKELQIISRWFADSSIASLNFYRKCYVEFYFWMAAAISEPEFSGSRVAFTKIAILMTMLDDLYDTHGTLDQLKIFTEGVRRWDVSLVEGLPDFMKIAFEFWLKTSNELIAEAVKAQGQDMAAYIRKNAWERYLEAYLQDAEWIATGHVPTFDEYLNNGTPNTGMCVLNLIPLLLMGEHLPIDILEQIFLPSRFHHLIELASRLVDDARDFQAEKDHGDLSCIECYLKDHPESTVEDALNHVNGLLGNCLLEMNWKFLKKQDSVPLSCKKYSFHVLARSIQFMYNQGDGFSISNKVIKDQVQKVLIVPVPI

>AGQ38710

MAMPSSSLSSQIPTAAHHLTANAQSIPHFSTTLNAGSSASKRRSLYLRWGKGSNKIIACVGEGGATSVPYQSAEKNDSLSSSTLVKREFPPGFWKDDLIDSLTSSHKVAASDEKRIETLISEIKNMFRCMGYGETNPSAYDTAWVARIPAVDGSDNPHFPETVEWILQNQLKDGSWGEGFYFLAYDRILATLACIITLTLWRTGETQVQKGIEFFRTQAGKMEDEADSHRPSGFEIVFPAMLKEAKILGLDLPYDLPFLKQIIEKREAKLKRIPTDVLYALPTTLLYSLEGLQEIVDWQKIMKLQSKDGSFLSSPASTAAVFMRTGNKKCLDFLNFVLKKFGNHVPCHYPLDLFERLWAVDTVERLGIDRHFKEEIKEALDYVYSHWDERGIGWARENPVPDIDDTAMGLRILRLHGYNVSSDVLKTFRDENGEFFCFLGQTQRGVTDMLNVNRCSHVSFPGETIMEEAKLCTERYLRNALENVDAFDKWAFKKNIRGEVEYALKYPWHKSMPRLEARSYIENYGPDDVWLGKTVYMMPYISNEKYLELAKLDFNKVQSIHQTELQDLRRWWKSSGFTDLNFTRERVTEIYFSPASFIFEPEFSKCREVYTKTSNFTVILDDLYDAHGSLDDLKLFTESVKRWDLSLVDQMPQQMKICFVGFYNTFNDIAKEGRERQGRDVLGYIQNVWKVQLEAYTKEAEWSEAKYVPSFNEYIENASVSIALGTVVLISALFTGEVLTDEVLSKIDRESRFLQLMGLTGRLVNDTKTYQAERGQGEVASAIQCYMKDHPKISEEEALQHVYSVMENALEELNREFVNNKIPDIYKRLVFETARIMQLFYMQGDGLTLSHDMEIKEHVKNCLFQPVA

>AGQ41594

MAQLSFNAALKMNALGNKAIHDPTNCRAKSERQMMWVCSRSGRTRVKMSRGSGGPGPVVMMSSSTGTSKVVSETSSTIVDDIPRLSANYHGDLWHHNVIQTLETPFRESSTYQERADELVVKIKDMFNALGDGDISPSAYDTAWVARLATISSDGSEKPRFPQALNWVFNNQLQDGSWGIESHFSLCDRLLNTTNSVIALSVWKTGHSQVQQGAEFIAENLRLLNEEDELSPDFQIIFPALLQKAKALGINLPYDLPFIKYLSTTREARLTDVSAAADNIPANMLNALEGLEEVIDWNKIMRFQSKDGSFLSSPASTACVLMNTGDEKCFTFLNNLLDKFGGCVPCMYSIDLLERLSLVDNIEHLGIGRHFKQEIKGALDYVYRHWSERGIGWGRDSLVPDLNTTALGLRTLRMHGYNVSSDVLNNFKDENGRFFSSAGQTHVELRSVVNLFRASDLAFPDERAMDDARKFAEPYLREALATKISTNTKLFKEIEYVVEYPWHMSIPRLEARSYIDSYDDNYVWQRKTLYRMPSLSNSKCLELAKLDFNIVQSLHQEELKLLTRWWKESGMADINFTRHRVAEVYFSSATFEPEYSATRIAFTKIGCLQVLFDDMADIFATLDELKSFTEGVKRWDTSLLHEIPECMQTCFKVWFKLMEEVNNDVVKVQGRDMLAHIRKPWELYFNCYVQEREWLEAGYIPTFEEYLKTYAISVGLGPCTLQPILLMGELVKDDVVEKVHYPSNMFELVSLSWRLTNDTKTYQAEKARGQQASGIACYMKDNPGATEEDAIKHICRVVDRALKEASFEYFKPSNDIPMGCKSFIFNLRLCVQIFYKFIDGYGIANEEIKDYIRKVYIDPIQV

>AGQ948Z0

MALLSITPLVSRSCLSSSHEIKALRRTIPTLGICRPGKSVAHSINMCLTSVASTDSVQRRVGNYHSNLWDDDFIQSLISTPYGAPDYRERADRLIGEVKDIMFNFKSLEDGGNDLLQRLLLVDDVERLGIDRHFKKEIKTALDYVNSYWNEKGIGCGRESVVTDLNSTALGLRTLRLHGYTVSSDVLNVFKDKNGQFSSTANIQIEGEIRGVLNLFRASLVAFPGEKVMDEAETFSTKYLREALQKIPASSILSLEIRDVLEYGWHTNLPRLEARNYMDVFGQHTKNKNAAEKLLELAKLEFNIFHSLQERELKHVSRWWKDSGSPEMTFCRHRHVEYYALASCIAFEPQHSGFRLGFTKMSHLITVLDDMYDVFGTVDELELFTATIKRWDPSAMECLPEYMKGVYMMVYHTVNEMARVAEKAQGRDTLNYARQAWEACFDSYMQEAKWIATGYLPTFEEYLENGKVSSAHRPCALQPILTLDIPFPDHILKEVDFPSKLNDLICIILRLRGDTRCYKADRARGEEASSISCYMKDNPGLTEEDALNHINFMIRDAIRELNWELLKPDNSVPITSKKHAFDISRVWHHGYRYRDGYSFANVETKSLVMRTVIEPVPL

>AGQ9M7D1

MALVSSAPKSCLHKSLIRSTHHELKPLRRTIPTLGMCRRGKSFTPSVSMSLTTAVSDDGLQRRIGDYHSNLWDDDFIQSLSTPYGEPSYRERAEKLIGEVKEMFNSMPSEDGESMSPLNDLIERLWMVDSVERLGIDRHFKKEIKSALDYVYSYWNEKGIGCGRDSVFPDVNSTASGFRTLRLHGYSVSSEVLKVFQDQNGQFAFSPSTKERDIRTVLNLYRASFIAFPGEKVMEEAEIFSSRYLKEAVQKIPVSSLSQEIDYTLEYGWHTNMPRLETRNYLDVFGHPTSPWLKKKRTQYLDSEKLLELAKLEFNIFHSLQQKELQYLSRWWIHSGLPELTFGRHRHVEYYTLSSCIATEPKHSAFRLGFAKTCHLITVLDDIYDTFGTMDEIELFNEAVRRWNPSEKERLPEYMKEIYMALYEALTDMAREAEKTQGRDTLNYARKAWEVYLDSYTQEAKWIASGYLPTFEEYLENAKVSSGHRAAALTPLLTLDVPLPDDVLKGIDFPSRFNDLASSFLRLRGDTRCYKADRDRGEEASSISCYMKDNPGLTEEDALNHINAMINDIIKELNWELLKPDSNIPMTARKHAYEITRAFHQLYKYRDGFSVATQETKSLVRRTVLEPVPL

>ObQ15GI4

MEENGMKSKILIFGGTGYIGNHMVKGSLKLGHPTYVFTRPNSSKTTLLDEFQSLGAIIVKGELDEHEKLVELMKKVDVVISALAFPQILDQFKILEAIKVAGNIKRFLPSDFGVEEDRINALPPFEALIERKRMIRRAIEEANIPYTYVSANCFASYFINYLLRPYDPKDEITVYGTGEAKFAMNYEQDIGLYTIKVATDPRALNRVVIYRPSTNIITQLELISRWEKKIGKKFKKIHVPEEEIVALTKELPEPENIPIAILHCLFIDGATMSYDFKENDVEASTLYPELKFTTIDELLDIFVHDPPPPASAAF

>ObQ2KNL6

MAKSPETEHPVKALGWAATDNSGTFSPFNFSRRATGERDVQFRVLYCGVCHSDLHMVKNEWGVTHYPIVPGHEIVGIVTEVGSKVEKVKIGDKVGVGVLVGSCRQCDQCSNDLENYCYKQILTYGAPYLDGTIARGGYSDIMVADEHFIIRWPENFPLDAGAPLLCAGITTYSPLKYFGLDKPGLRVGVNGLGGLGHVAVKFAKAFGTKVTVISTSLSKKEEAMQHLGVDEFVVSTDPQQMQAAVGTLDGIIDTVSAPHPIVPLLSLLKPHGKLIVVGLPDKPLQLPVFPLIQGRRTIAGSGIGGLKETQEMIDFAAKNNIVADVEVIPIDYINTAMDRLLKSDVKYRFVIDVEKSLKPQ

>ObQ5SBP0

MSTFVISNSMHVGISFSFLHKLPQTPPPQVVCCSGGLRLRPSCSLQLQPPPTTRRSGNYEPSAWDFNYLQSLNNYHHKEERYLRRQADLIEKVKMILKEEKMEALQQLELIDDLRNLGLSYCFDDQINHILTTIYNQHSCFHYHEAATSEEANLYFTALGFRLLREHGFKVSQEVFDRFKNEKGTDFRPDLVDDTQGLLQLYEASFLLREGEDTLEFARQFATKFLQKKVEEKMIEEENLLSWTLHSLELPLHWRIQRLEAKWFLDAYASRPDMNPIIFELAKLEFNIAQALQQEELKDLSRWWNDTGIAEKLPFARDRIVESHYWAIGTLEPYQYRYQRSLIAKIIALTTVVDDVYDVYGTLDELQLFTDAIRRWDIESINQLPSYMQLCYLAIYNFVSELAYDIFRDKGFNSLPYLHKSWLDLVEAYFQEAKWYHSGYTPSLEQYLNIAQISVASPAILSQIYFTMAGSIDKPVIESMYKYRHILNLSGILLRLPDDLGTASDELGRGDLAKAMQCYMKERNVSEEEARDHVRFLNREVSKQMNPARAADDCPFTDDFVVAAANLGRVADFMYVEGDGLGLQYPAIHQHMAELLFHPYA

>ObQ5SBP1

MWSTISISMNVAILKKPLNFLHNSNNKASNPRCVSSTRRRPSCPLQLDVEPRRSGNYQPSAWDFNYIQSLNNNHSKEERHLERKAKLIEEVKMLLEQEMAAVQQLELIEDLKNLGLSYLFQDEIKIILNSIYNHHKCFHNNHEQCIHVNSDLYFVALGFRLFRQHGFKVSQEVFDCFKNEEGSDFSANLADDTKGLLQLYEASYLVTEDEDTLEMARQFSTKILQKKVEEKMIEKENLLSWTLHSLELPLHWRIQRLEAKWFLDAYASRPDMNPIIFELAKLEFNIAQALQQEELKDLSRWWNDTGIAEKLPFARDRIVESHYWAIGTLEPYQYRYQRSLIAKIIALTTVVDDVYDVYGTLDELQLFTDAIRRWDIESINQLPSYMQLCYLAIYNFVSELAYDIFRDKGFNSLPYLHKSWLDLVEAYFVEAKWFHDGYTPTLEEYLNNSKITIICPAIVSEIYFAFANSIDKTEVESIYKYHDILYLSGMLARLPDDLGTSSFEMKRGDVAKAIQCYMKEHNASEEEAREHIRFLMREAWKHMNTAAAADDCPFESDLVVGAASLGRVANFVYVEGDGFGVQHSKIHQQMAELLFYPYQ

>ObQ5SBP2

MWSTISISMNVAILKKPLNFLHNSNNKASNPRCVSSTRRRPSCPLQLDVEPRRSGNYQPSAWDFNYIQSLNNNHSKEERHLQGKAKLIEEVKMLLEQEMAAVQQLEFIEDLKNLGLSYLFQDEIKIILNSIYNHHKCFHNNHQQRTDENADLYFVALGFRLFRQHGFKVSQEVFDCFKNEEGSDFIPNLAEDTKGLLQLYEASYLVRQDEDTLEMARQFSTKILQKKVEEKMIEENLLSWTCHSLELPLHWRVQRIEAKWFLDAYASKPDMNPIIFELAKLEFNIAQALQQGELKDLSRWWNDTGIAEKLPFARDRIVEAHYWAIGTLEPYQYRYQRSLIAKIIALTTVVDDVYDVYGTLDEPQLFTDAIRRWDIESINQLPHYLQLCYLAIYNFVSELAYDIFRDKGFNSLPYLHKSWLDLVEAYFLEAKWFHSGYTPTLEEYLNNSKMTITCPAIVSEIYFAFANSIDKTEVESVYKYHDILYLSGMLLRLPDDLGTTTFEMKRGDVAKAIQCYMKEHNASEEEAREHIRFLMREAWKQMNTAAAANNCPFVNDFVVGAASLGRVANFVYVEGDGFGVQHSKIHQQMAELLFYPYQ

>ObQ5SBP3

MSCARITVTLPYRSAKTSIQRGITHCPALLRPRFSACTPLASAVPLSSTPLINGDNSPLKNTHQHVEERSSKRREYLLEETARKLQRNDTESVEKLKLIDNIQRLGIGYYFEDAIDAVLRSPFSAEEEEDLFTAALRFRLLRHNGIQVTPEIFLKFKDERGEFDESDTLGLLSLYEASNLGVTGEEILEEAMEFAEPRLRRSLSELAAPLRSEVAQALDVPRHLRMARLEARRFIEQYGKQSDHDGDLLELAILDYNQVQAQHQSELTEITRWWKQLGLVEKLGFGRDRALECFMWTMGILPHPKYSSSRIESAKAAALLYVIDDIFDTYGKMDELILFTDAIRRWDLEAMEGLPEYMKICYMALYNTTNEICYRVLKDTGRIALPYLKSVWIETIEAYMVEVKWFSGGSAPKLEEYIENGASTVGAYMVLVHLFFLIGEGLTHQNVLFFKQKPYHKPFSAAGRIFRLWDDLGTSQEEEERGDMASSIRLFMKEYKLSTVEEARSCVLEEISRLWKDLNEGLISIKDALPLTIVKVALNIARTSQVVYKHEQHTYMLSVDNYVEALFFTPLLSS

>ObQ5SBP4

MESRRSANYQASIWDDNFIQSLASPYAGEKYAEKAEKLKTEVKTMIDQTRDELKQLELIDNLQRLGICHHFQDLTKKILQKIYGEERNGDHQHYKEKGLHFTALRFRILRQDGYHVPQDVFSSFMNKAGDFEESLSKDTKGLVSLYEASYLSMEGETILDMAKDFSSHHLHKMVEDATDKRVANQIIHSLEMPLHRRVQKLEAIWFIQFYECGSDANPTLVELAKLDFNMVQATYQEELKRLSRWYEETGLQEKLSFARHRLAEAFLWSMGIIPEGHFGYGRMHLMKIGAYITLLDDIYDVYGTLEELQVLTEIIERWDINLLDQLPEYMQIFFLYMFNSTNELAYEILRDQGINVISNLKGLWVELSQCYFKEATWFHNGYTPTTEEYLNVACISASGPVILFSGYFTTTNPINKHELQSLERHAHSLSMILRLADDLGTSSDEMKRGDVPKAIQCFMNDTGCCEEEARQHVKRLIDAEWKKMNKDILMEKPFKNFCPTAMNLGRISMSFYEHGDGYGGPHSDTKKKMVSLFVQPMNITI

>ObQ5SBP5

MDVSILRDVRPPVTSYAPNIWADTFSNISLDEEVQKKYAETIEALKQVVRGMLMAAATPIKQMIFIDTLERLGLAYHFETEIEHKLQKIYDDNVCGDDCDLFTTALRFRLLRQHRHHVSCDVFDKFLYEEGKFKGDAEGLLSLYEASHVRFHNEKILEEAERFTRQELSCWIKLQSPLKDKVKRALERPLHREVPILYARHFISIYEKDESMDEHLLKLAKFNFNFLQNLYKKELYDLSRWWNKFDLKTKLPYIRDRLAEAYLWGVGYHFEPQYSYVRKGVVLSIKIIGILDDTYDNYATVNEAQLFTEILDRWSMDEIDRLPDYMKIVLHFVMSAYEEYERDAKIVYGKKFASPYFKETIQQLARGYNQELKWVMEKQMPPFKDYLKNSEITSCIYIMFASIIPGLKSFTQEAIDWIKNEPNFAVKAGLIGRYWDDIGSHKRESKGGEMLTVMDCYMKQYSVSIQETISEFAKAVEDSWKEVNEGWVYTISMSKEITVQFLNYSRMCDASYNRNNGDGYTDPSFAKSNITALFVDPIII

>ObQ5SBP6

MTNMFASAAPISTNNTTVEDMRRSVTYHPSVWKDHFLDYASGITEVEMEQLQKQKERIKTLLAQTLDDFVLKIELIDAIQRLGVGYHFEKEINHSLRQIYDTFQISSKDNDIRVVALRFRLLRQHGYPVPSDVFKKFIDNQGRLDESVMNNVEGMLSLYEASNYGMEGEDILDKALEISTSHLEPLASRSRRINEALEMPISKTLVRLGARKFISIYEEDESRDEDLLKFAKLDFNILQKIHQEELTHIARWWKELDLGNKLPFARDRVVECYFWILGVYFEPQYNIARRFMTKVIAMTSIIDDIYDVHGTLEELQRFTDAIRSWDIRAIDELPPYMRLCYEALLGMYAEMENEMVKQNQSYRIEYARQEMIKLVTTYMEEAKWCYSKYIPNMDEYMKLALVSGAYMMLATTSLVGILGDPITKQDFDWITNEPPILRAASVICRLMDDVVGHGIEQKISSVDCYMKENGCSKMEAVGEFSKRVKKAWKNLNEEWVEPRAASMVILVRVVNLARVINLLYVGEDSYGNSSVKTKELIKGVLVHPIK

>ObQ5SBP7

MSANCVSAAPTSPKNSDVEEIRKSATYHSSVWGNHFLSYTSDVTEITAAEKEQLEKLKEKVKNLLAQTPDESTGKMELIDAIQRLGVGYHFTTEIQESLRQIHEGQIRNDDDDVRVVALRFRLLRQGGYRAPCDVFEKFMDDGGNFKESLKKDVEGMLSLYEASYYGIDGEEIMDKALEFSSSHLESMLHNISTKTNKSLLRRLQEALDTPISKAAIRLGATKFISTYREDESHNEDILNFAKLDFNILQKMHQEEANYLTRWWEDLDLASKLDFARDRMVESYFWSLGVYFQPQYRTSRIYLTKIISIVAVIDDIYDVYGSFDDLRSFTDVIQSWKISNADELPPYMRICFEALLGIYEDMGDRIGAPYAIDTMKELVDTYMQEAEWCYTEYVPTVDEYMKVALVTGGYLMVATTFLTGINNITKKDFDWIRNRPRLLQVAEVLTRLMDDIAGHGTEKKTTAVSCYMKEYECSEMEASRELSKQVKKAWKDLNDEWMEPRSSSAEIIGCIVNMSRVLHIMYSTGDDGFSDSSTRTTQAVKTLLVDHPMN

>ObQ6USK1

MSCARITVTLPYRSAKTSIQRGITHYPALIRPRFSACTPLASAMPLSSTPLINGDNSQRKNTRQHMEESSSKRREYLLEETTRKLQRNDTESVEKLKLIDNIQQLGIGYYFEDAINAVLRSPFSTGEEDLFTAALRFRLLRHNGIEISPEIFLKFKDERGKFDESDTLGLLSLYEASNLGVAGEEILEEAMEFAEARLRRSLSEPAAPLHGEVAQALDVPRHLRMARLEARRFIEQYGKQSDHDGDLLELAILDYNQVQAQHQSELTEIIRWWKELGLVDKLSFGRDRPLECFLWTVGLLPEPKYSSVRIELAKAISILLVIDDIFDTYGEMDDLILFTDAIRRWDLEAMEGLPEYMKICYMALYNTTNEVCYKVLRDTGRIVLLNLKSTWIDMIEGFMEEAKWFNGGSAPKLEEYIENGVSTAGAYMAFAHIFFLIGEGVTHQNSQLFTQKPYPKVFSAAGRILRLWDDLGTAKEEQERGDLASCVQLFMKEKSLTEEEARSRILEEIKGLWRDLNGELVYNKNLPLSIIKVALNMARASQVVYKHDQDTYFSSVDNYVDALFFTQ

>ObQ93WU2

MALQKVDISLSTEQLLQAQVHVWNHMYAFANSMSLKCAIQLGIPDILHKHGRPMTLSQLLQSIPINKEKTQCFQRLMRALVNSNFFIEENNSNNQEVCYWLTPASCLLLKEAPLTVTPLVQVVLDPTFTNPWHHMSEWFTHEKHATQFEAANGCTFWEKLANEPSKGRFFDEAMSCDSRLIAHVFTKDYKHVIEGIRTLVDVGGGNGTMAKAIVEAMPTIKCTVIDLPHVVAGLESTDNLNYIGGDMFQSIPSADAILLKSIIHDWDDVEGLKILKKCKDAVVMGGKVIIIDVVVGVNHDIDEVLEDQLHFDMAMMCYFNAKERTMSEWEKLIYDAGFKSYKLTPAFGVRSLIEAYP

>PFQ9AXM7

MYTGVIMHMAIPIKPAHYLHNSGRSYASQLCGFSSTSTRAAIARLPLCLRFRCSLQASDQRRSGNYSPSFWNADYILSLNNHYKEESRHMKRAGELIVQVKMVMGKETDPVVQLELIDDLHKLALSHHFEKEIKEILFNISIYDHKIMVERDLYSTALAFRLLRQYGFKVPQEVFDCFKNDNGEFKRSLSSDTKGLLQLYEASFLLTEGEMTLELAREFATIFLQEKLNDKTIDDDDDADTNLISCVRHSLDIPIHWRIQRPNASWWIDAYKRRSHMNPLVLELAKLDLNIFQAQFQQELKQDLGWWKNTCLAEKLPFTRDRLVECYFWCTGIIQPLQHENARVTLAKVNALITTLDDIYDVYGTLEELELFTEAIRRWDVSSIDHLPNYMQLCFLALNNFVDDTAYDVMKEKDINIIPYLRKSWLDLAETYLVEAKWFYSGHKPNMEEYLNNAWISISGPVMLCHVFFRVTDSITRETVESLFKYHDLIRYSSTILRLADDLGTSLEEVSRGDVPKSIQCYMNDNNASEEEARRHVRWLIAETWKKINEEVWSADSPFCKDFIACAADMGRMAQFMYHNGDGHGIQNPQIHQQMTDILFEQWL

>MLQ9SW76|

MALKVFSVATQMAIPSKLTRCLQPSHLKSSPKLLSSTNSSSRSRLRVYCSSSQLTTERRSGNYNPSRWDVEFIQSLHSDYEEDKHAIRASELVTLVKMELEKETDHIRQLELIDDLQRMGLSDHFQNEFKEILSSIYLDHHYYKNPFPKEERDLYSTSLAFRLLREHGFQVAQEVFDSFKNEEGEFKESLSDDTRGLLQLYEASFLLTEGETTLESAREFATKFLEERVNEGGVDGDLLTRIAYSLDIPLHWRIKRPNAPAWIEWYRKRPDMNPVVLELAILDLNIVQAQFQEELKESFRWWRNTGFVEKLPFARDRLVECYFWNTGIIEPRQHASARIMMGKVNALITVIDDIYDVYGTLEELEQFTDLIRRWDINSIDQLPDYMQLCFLALNNFVDDTSYDVMKEKGVNVIPYLRQSWVDLADKYMVEARWFYGGHKPSLEEYLENSWQSISGPCMLTHIFFRVTDSFTKETVDSLYKYHDLVRWSSFVLRLADDLGTSVEEVSRGDVPKSLQCYMSDYNASEAEARKHVKWLIAEVWKKMNAERVSKDSPFGKDFIGCAADLGRMAQLMYHNGDGHGTQHPIIHQQMTRTLFEPFA

>NTG3CCC0

MQVIITSSHRFFCHHLHQLKSPTSLSAQKAEFKKHGPRNWLFQTEGSLLYKPVRLNCATSDASYLGNVNEYLESDHSKNSEEKDIQVSRTIQMKGLTEEIKHMLNSMEDGRLNVLAYDTAWVSFIPNTTNNGNDQRPMFPSCLQWIIDNQLSDGSWGEEIVFCIYDRLLNTLVCVIALTLWNTCLHKRNKGVMFIKENLSKLETGEVENMTSGFELVFPTLLEKAQQLDIDIPYDAPVLKDIYARREVKLTRIPKDVIHTIPTTVLFSLEGLRDDLDWQRLLKLQMPDGSFLISPASTAFAFMETNDEKCLAYLQNVVEKSNGGARQYPFDLVTRLWAIDRLQRLGISYYFAEEFKELLNHVFRYWDEENGIFSGRNSNVSDVDDTCMAIRLLRLHGYDVSPDALNNFKDGDQFVCFRGEVDGSPTHMFNLYRCSQVLFPGEKILEEAKNFTYNFLQQCLANNRCLDKWVIAKDIPGEIWYALEFPWYASLPRVEARYYIEQYGGADDIWIGKTLYRMPDVNNNVYLQAAKLDYNRCQSQHRFEWLIMQEWFEKCNFQQFGISKKYLLVSYFLAAASIFEVEKSRERLAWAKSRIICKMITSYYNDEATTWTTRNSLLMEFKVSHDPTRKNGNETKEILVLKNLRQFLRQLSEETFEDLGKDIHHQLQNAWETWLVFLREEKNACQEETELLVRTINLSGGYMTHDEILFDADYENLSNLTNKVCGKLNELQNDKVTGGSKNTNIELDMQALVKLVFGNTSSNINQDIKQTFFAVVKTFYYSAHVSEEIMNFHISKVLFQQV

>NTG3CCC1

MVLGLRSKIIPLPDHKLGNIKLGSVTNAICHRPCRVRCSHSTASSMEEAKERIRETFGKIELSPSSYDTAWVAMVPSRYSMNQPCFPQCLDWILENQREDGSWGLNPSHPLLVKDSLSSTLASLLALRKWRIGDNQVQRGLGFIETHGWAVDNKDQISPLGFEIIFPCMINYAEKLNLDLPLDPNLVNMMLCERELTIERALKNEFEGNMANVEYFAEGLGELCHWKEMMLRQRHNGSLFDSPATTAAALIYHQYDEKCFGYLNSILKLHDNWVPTICPTKIHSNLFLVDALQNLGVDRYFKTEVKRVLDEIYRLWLEKNEEIFSDVAHCAMAFRLLRMNNYEVSSEELEGFVDQEHFFTTSSGKLMNHVAILELHRASQVAIHERKDHILDKISTWTRNFMEQKLLDKHIPDRSKKEMEFAMRKFYGTFDRVETRRYIESYKMDSFKILKAAYRSSGINNIDLLKFSEHDFNLCQTRHKEELQQMKRWFTDCKLEQVGLSQQYLYTSYFIIAAILFEPEYADARLAYAKYAIIITAVDDFFDCFICKEELQNIIELVERWEGYSTVGFRSERVRIFFLALYKMVEEIAAKAETKQGRCVKDHLINLWIDMLKCMLVELDLWKIKSTTPSIEEYLSVACVTIGVPCFVLTSLYLLGPKLSKDVIESSEVSALCNCTAAVARLINDIHSYKREQAESSTNMVSILITQSQGTISEEEAIRQIKEMMESKRRELLGMVLQNKESQLPQVCKDLFWTTINAAYSIHTHGDGYRFPEEFKNHINDVIYKPLNQYSP

>NTQ40577

MASAAVANYEEEIVRPVADFSPSLWGDQFLSFSIKNQVAEKYAKEIEALKEQTRNMLLATGMKLADTLNLIDTIERLGISYHFEKEIDDILDQIYNQNSNCNDLCTSALQFRLLRQHGFNISPEIFSKFQDENGKFKESLASDVLGLLNLYEASHVRTHADDILEDALAFSTIHLESAAPHLKSPLREQVTHALEQCLHKGVPRVETRFFISSIYDKEQSKNNVLLRFAKLDFNLLQMLHKQELAQVSRWWKDLDFVTTLPYARDRVVECYFWALGVYFEPQYSQARVMLVKTISMISIVDDTFDAYGTVKELEAYTDAIQRWDINEIDRLPDYMKISYKAILDLYKDYEKELSSAGRSHIVCHAIERMKEVVRNYNVESTWFIEGYTPPVSEYLSNALATTTYYYLATTSYLGMKSATEQDFEWLSKNPKILEASVIICRVIDDTATYEVEKSRGQIATGIECCMRDYGISTKEAMAKFQNMAETAWKDINEGLLRPTPVSTEFLTPILNLARIVEVTYIHNLDGYTHPEKVLKPHIINLLVDSIKI

**Partial sequences:**

>scaffold6100.g77428.t1

MKVIDRDYFVEIKWREEKYKPRSEEYMQVATASCAYTSLIIISFVGMGDCVRKEDFEFVLSQPNIVRAALAICRLTDDLVGHEFEQEREHIPSWVECYTDEHKISKSEAVSEFKNRIESSWKDINEAFLNPTQIPTSQLYRILNFTRVIEVIYSKGDWYTHVGPQMQNLITQLLIQPIP

>scaffold5960.g76948.t1

MLQWAGLSRSYYKEATWFHGGYIPTTEEYLNVSYISISGPTLLFYGYFTTTNPINKHELESLERLARSPSMVLRLADDLGTSSDEVERGDVSKSIQCFMNETGCCEEEARQHVKRLIDAEWKKMNRDILMEKPFKSFGSIAMNLGRIALCFYENGDGFGLPHSETKKKMVSLVVHPIHMP

>scaffold5960.g76951.t1

MGVTPSLEEYLDNGWASSSGQVLCLHVLAGVGDDMADTLGLADHEIIYHTSLIFRLCNDLATFKAEAERGDSPSSIACYMKEANATEKEAREHIKELVSESWKKINGAWMRCPKSQQKMMRHAVNIARVANFLYQNGDGFGVQDQETKNKVLSCLIHPSQLAPPPL

>scaffold543.g21655.t1

MMKENEWSRDKFVPTIDEYISNAYVSFALGPIVLPALYLVGPKLSEEMVNHSEYHNLFKLMSTCGRLLNDIRSYERELKDGKLNALSLYMMNNGGEVTMEAAVSEVRSWIERQRRELLRTVLGKTVVPEACKELFWHMCTVVHMFYSKDDGFTSQDLLGVVNAIIKEPLVINDS

>scaffold2410.g52914.t1

MKRLVRAYFDEAKWLYRDYVPTMEEYMKVSFMSCGFRMLSTSSLVGMGDLVTKQDFDWTKNEPLIVRSASVLCRLMDDLVGDKYEQKPTAVCCYMKQYGVSKDEACRELREQVKKTWKDMNQECLEPRPASMEILTRIVNLCRIMNLLYADYDCYTDPIKSKEWVKMVFVEPIAI

>scaffold2410.g52916.t1

MQMKKLVMAYFEEAKWLYGDCIPTVEEYMKVSLVSCGYMMLSTSSLVGMGDLVTKQDFDWIINEPLVVRASSVICRLMDDLVGDEETSSVHCYMKQYGVSKDEAREELRKQVKNAWKDMNAECQEPRAASMEILTRVVNLGRVINLLYADDDCYANPIKSKEWLKMVLLDPIVI

>scaffold3179.g60793.t1

MEEAEWCYNKIIPTMDEYMKLAIVSIGYTLVATTSLVGIIEDFITENDFYWISNHPPITQASETLSRILDDLTGHGEQKIIVIDCYMKEYGCLEMEACRELRKQVEKPWKDLNDERMKKSRVISTEVLACIVNLTRVAHMMYSTGQDEYSNSSAKIKEEIRSLLIDPIN

>scaffold10009.g84173.t1

MAMKRENREGKLPTVVDCYMKDKGISKQEALSDFSKLVENGWKDITAEWVKGSAAPKELMEHLLNYGRIAEVTYKNNEDGYTDPEKYVGPQIASLYMEPIPL

>scaffold2410.g52920.t1

MENLGRAYAVQYSKQEVKKLVRAYFEEAKWLYNDYVPTVEEYMKVSLVSCGYMALSTSCLIGTGDLVTKQDFDWISNKPLIVRVSTVICRLMDDLVGDKYEQKPMAVYCYMKQYGASKDEACRELREQVKKAWKDMNQECLEPRPASIEILTHIVNLARIMNLLYAEDDGYTDPVKSKEWVKMVFVDPIVI

>scaffold3507.g63415.t1

MAMEDWPPYIQTCYKSIVDTYAGIEEQMEKEGKSYRVEYAKQDLKKLVMAYFEEAKWLYRDYVPTAEEYMKVSLVSCGYMMISTSSLVGMGDLVTKQDFDWITNEPLIVRASSVICRLMDDLVGDEDQARDELRKQVKNAWKDMNQECLEPRPASMQILMRVVNLGRVINLLYADDDCYANPIKSKEWVKMVLLDPMDI

>scaffold1680.g43587.t1

MLIILALAFLQLPLASAFVARRNYSQNNGDGRGTTVLPFGKPTIFNRSKLITRWWKNTCLVEKLPFVRDRVVESYFWTIGILGPRQYRKGRLVVAKVIALATVMDDIYHVYGTLEELELFTEAIRRWDFSSINQLPYYMQLCYLALTNFVNDTAYDVLKEKGFNIIPYLQKSVIYTCTEMPWPTLIYTGIESLNPTLAN

>scaffold5057.g73206.t1

MEGGMRMANSIKAVVAKLPYARDRVVECFFWAMGVYQEPRYSRACVMLTKTIAMTSIMMLMVKKDRGQIATGIESYMRENGSTKEEAVAKFFEIATDAWKDINEECMRPSPYSRDILMRILNLERIIDIIYKGNANGYTQPEKVL

>scaffold6279.g77994.t1

MSLYEDYEREAEKQEKPFAAHYYKESVKQLCRAYSKEQTWIREQRMPPFEEYMANAVINTCINQSFIAFVPTIKCLDEEAVQWLLSEPNMLFVHCKDESHKGGFSQP

>scaffold5167.g73735.t1

MLVAGLLIYRVIDDMATYEVEKDRGQIATGIKSYTKENGVTIEEAIAKFFEIVVDAWKDINEECNEDGYTQPEKVLKPHIIALFVDPVQI

>scaffold6279.g77995.t1

MVKVVEQIRPKDKVVVRKRSSRYLWSNVLHYEPQFSDFRMAFAKNMKLCTILDDTYDNYATLDEDDLFTEILEKYISVQTSGSAGSGSKFEPKPPVHGPK

>scaffold9733.g83970.t1

MLTAVDCYMKQYGVSKQETMSKFAEFVEDKWKDINKEWATTTSPHNETKVQFLNYARMADACYNKNNGDAFTDPKIFSTQIASLFVDPIAI

>scaffold6894.g79660.t1

MDCYMKQYGVSTQETISKFAEAVEDSWKEVNEGWVGTISIPKEITVQFLNYSRMCDASYNRNNGDGYTDPSFAKPNITALFVDPIVI

>scaffold6894.g79658.t1

MCIVLKREEKGGETLTVMDCYMKQYGVSKQETISRFSDEIEDLWKEVNKEWVVTTSVPREIAVQFVNYSRLCDATYNKNNGDGYTDPKLSALSIRALFLDPIVIS

>scaffold6894.g79657.t1

MDEADQLPDYMKTTLQFIMSAYEEYERYAEKNGKQFASPYFKETIQQLGRAYNQELKWIMENQMPPFQDYLKNSEITSCIYILFASVMPGLKSLTQETIDWIKSEPSFAVKAGLIGRYWDDIGSHRVCLSTLFE

**Pseudogenes sequences:**

>scaffold6367.g78236.t1

MARNQPLKWYTWSMAVLKDDTRLSEQRVQLTKSIAFIYLIDDVFDLYGTLNELDIFNQAVHRWDYSAMDMLPEYMKTCYKSLLDTTNEIATSISENHGYNPIIILKQTWVSLCNAFLVEAKWLASKNLPRAEEYFDNAKVSSGVHVALVHLFFLLGLDESDLHDKCKIISCVAAILRLWDDLGSAEDENQNGRDGSYIECYMRDEPSLAFEEAREHVTEMIEREWKRLNKECLNQSSSSSQFEEAALNMARMVSIMYSYDHNHRLPLLK

>scaffold5960.g76949.t1

MESRRSANLGASIWDDHFIQSLTSSYTGEKHAEKAEKLKAEVRTMMEQTTDELKQLELIDNLQRLGICNHFQDLIQKILQKMYEEERYGDRQKNLHITALRFRLLRQDGYHVPPDVFSSFMDKTGDFQEHLSKDITGLVSLYEASYLSMEGETILDLAKDFSSHHLHKIVEETKEKRVADQIIHALELPLHWRVQRLEARWFIQYYECGSDANPSLVELAKLDYNMVQATYQEELKRLSRWYEETGLSEKMSFARHRLAECFLWSMGFVPERHFGYARENLTKIAEFITILDDIYDVYGTLEELQVLTEIMERWDINLLDRLPEYMRICFLAVFNATNELAYHILRDQGINVISNLRRLWAELSRSYYKEATWFHSGYIPATEEYLNTAYISISGPLIHFYSYITTTNPINKHELQSLEKHAYWPSMVLRLADDLGTSSVQNKQFAGSKIMVYESRLGNLEHKFGRDEERGCFEIDPVFHEQNWMLRVGSKAACEAFDRCSVEEDEQGHID

>scaffold581.g22644.t1

MSGLTMQVAKPNDDPEATSPRFGISTLFSCSTINEGICSPLPLDLDSSDNMVSPCPKRQVDECGDHQIDKTLLSSLRAALKSPSHWRIQIPNARSFIDDYKRRLDMNPAVLELAKLDINIVQSQYQEDLKEATRWWKNTRLVEHLPFVRDRIVECYSWSIGFLHPRHRRYERIMLARVIALITTIDDVFDVYGTLEELQLFTDVVRRMRLREATSQNQFSDNMNNKNASEGEARAHVRSLVAQTWKRVNEEMMSENSPFSKEFIEAAANIGRMGHYVVDLKDYLPKSVAFGFSASNTGYFFSSWPLFHTDDEDEEDSDDNESEDSWDILDSTIDNEFEEGSGPKRIPYSDLVLATNNFAKEEKLGEGGFGAVYTTPTLQ

>scaffold581.g22636.t1

MICRLVSVACARVAPYNSLITKPNDDSEATSSRFGISTTTISQDNVVDGMKKWTRGTCIRLINKGICTPLPLDSDSFHGMVSACPKNAKGVFKPGHADDIKGLLQLYEASFLLTQGEDTLELAREFAARILQRQVDECGDNQISSIRAFLKLPSHWRIQIPKARSFIDDCKRRPGMNPVMLELAKLDINIVQAQYQEDLKDESR

>scaffold543.g21654.t1

MSLPLSTRVLFVPKPSQFRSSSFSHASASLDIGLQRATSPPQSASVSTSFEETKDRIAKLFHKDELSVSTYDTAWVAMVPSPTSSEEPCFPDCLNWLLENQCHDGSWARPHHHPMLIKDVLSSTLACILALKKWGVGEEQINRGLHFIELNFASATEKSQITPVGFDIVFLAMLDYARDLSLNMRLDPTTLNDLICKRDLELKRCNQSNSTEREVYWAYIAEGMGKLQNWGSVIKYQRKNGSLFSCPSTTAAAFIALRNTDCLNYLRSALKKFGSAVPAVYPLDIYSQLCKVDNLERLGISRYFSNEFQSGDEEIFMDAATCALAFRTLRMNGYNVTSDPLTKILQECFPSSFHGNMTDINTTLEIYRASEHIIYEDERDLEKHNLRLKTFLEQELSSGGFVQSCQLGRNIDAEVNQALDYPFYAIMDRMAKRKNIELYNSDNTRILKTSYCSPNFGNKDFLHMSVEDFNRCQDIHREELKELERWVIENRLDELKFARSKSAYCYFSAAGTIFSPELSDARMSWAKNGVLTTVVDDFFDVGGSMEELKNLIHLVELWDVDVSTKCTSPNVQIIFSALKHTICEIGDKGFKLQGRSIMNHIISIVRSLTFHLHFLYIKNLSSFYADNSAAMVARHLKQTNNEIRHVQQLTVLGVSEILS

>scaffold5167.g73739.t1

MAPAVAEIQRPVANFSPSLWGEQFIKQDSAAHQVGEEEHSETVEVLKNEVRGMIGPGNMVDTMSLIDTLERLGVSYHFETEIEDKLQQYFDLNTDYNDEAYDLYTVALHFRLFRQHGHRISADIFARWRDENGKFQEGLKNDAKGLLSLYEASYLRTRGETLLDEAVEFATATLKSTAPHLESPLREQVEHALIQPLHFGNPRIEARNFISIYEEFGDKNESLLRFAKLDYNQLQMLHKEELHVVSRWWKDLDLVAKLPYARDRVVECFFWAMGVYHEPQYSRARIMLTKTIAMTSIIDDTYDAYGTIEELDVFTEAIERWHIDEINRLPEYIKPFYKALLELYEQFEEELADEGRSYAAHYALKELVRSYHLEAKWFIQGYLPPFEEYLKNALITCTYCYHTTTSLLGVETAIKEDFEWLSKKPKMLVAGLLICRVIDDIATYEVEKDRGQIATGIESYMKENGATIEETIAKFFEIAMDAWKDINEECMRPSPYSRDVLMRILNLERIIDVTYKGNEDGYTQPEKVLKPHIIALFVDPIQI

>scaffold5167.g73738.t1

MLHKEELHVVSRWWKDLDLVAKLPYARDRVVECFFWAMGVYHEPQYSRARIMLTKTIAMTSIIDDTYDAYGTIEELDFFSDAIERWNIEEMKKLPDYVKPFYKALLELYDQFEEELAKEGRSYAAYYAIESLKELVRSYHLEAKWFIQGYLPPFEEYLKNALITCTYCYHTTTSLLGVESAIKEDFEWLSKKPKMLVAGLLICRVIDDISTYEVEKDRGQIATGIESYMKENGATIDEAVAKFFEIAMDAWKDINEECMRPSPYSRDVLMRILNLERIIDVTYKGNEDGYTQPEKVLKPHIIALFVDPIQI

>scaffold5167.g73737.t1

MAPAVAEIQRPVANFSPSLWGEQFIKQDSAAHQVEEEEHSKTVEVLKNEVRGMIGTGNMVDTMSLIDTLERLGVSYHFETQIEEKLQQYFDLDTDYNDEAYDLYTVALHFRLFRQHGHRISADIFGRWRDENGKFQGLKNDARGLLSLYEASYLRTRGETLLDEAVEFATATLKSITPHLESPLREQVEHALIQPLHFGNPRIEARNFISIYEEFEDKSESLLRFAKLDYNQLQMLHKEELHVVSRWWKDLDLVAKLPYARDRVVECFFWAMGVYHEPQYSRARIMLTKTITMTSIIDDTYDAYGTIDELDAFTEAIERWNIEEIKRLPDYIKPFYRALLELYDQFEEELAKEGRSYHLEAKWFIQGYLPPFEEYLKNALITCTYCYHTTTSLLGVETAMKEDFEWLSKKPKMLVAGLLICRVIDDIATYEVEKDRGQIATGIESYMKENGATIEEAVAKFFEIAMDAWKDINEECMRPSPYSRDVLMRILNLERIIDVTYKGNEDGYTQPEKVLKPHIIALFVDPIQI

>scaffold4162.g68214.t1

MSDVSGPRQLLVKKAFSADHGPRIDASDAIPRSFLKYSWRERLQSTRFFFFFFFWSLGVYFEPHYKTARIFLAKALAMTTIVDDVYGSLGELRAFTTAIQSWDIRAAHELPPYMRICYEAFVAGIRGDSITENDFDRIVNRPSIIQASEVLARIMDDVAGHGKEQKIPAVDCYMKEHGCSGMEASRELSKQVKKGWKDLNDEWMQHLWRFLRALTGKIAEILNLRPERLQDSQFTTSILNDVEEDFLAHDLYALESFAGQSNAPEKLKQVFDILASVVCLKVAAHVARNYSGKVIYFDTGNSFSPKRVVQFLSLSSDSSTAEANKTVERVMSSIVCHSVFDIFTLLDMLHLLMNDLKSQIDCHVKMLIIDSLSSLIAPVLGGGGAHGHALMVTAGFLLKELAHEYNLSIVVTNHMVGGEAGVSKPALGESWKSIPHVRLLLSRDYSTNGRRITILKHPFLIRIKIARSMLSALVVTKEEWMYGTFLGARRWIAPQKGTFRIDVDANFNDETQFYGGGHSSSKSQMGNTGGSGKAYSKAKLYF

>scaffold3179.g60794.t1

MVASLWFGPHPYDTLHQSYFQKNISNPNQQSLGSDSIAAAITSDHRFQSVLAAALISDIGGSASMNKSSSLTSSQSACLTIVSPSFQFSSSNKNVFEKFMNGEGNFKESMKKDLEGILSLYEASYYGVNGEEIMGKALQFSSSHLESLIHQISIKTNRSLLRQLQEALDTPIIKTSIRLGATKFILTYQEDESHNEDLLNFAKLDLNILKKMHQEEANLITSWDISVADELPPYMKICYETLVGVYAGMEDELEKYGNSYRIQYAK

>scaffold2421.g53048.t1

MAPPQAEIQRPVANFSPSLWGEQFIKHDPASHQVEEKHSKTVQVLKNEVRGMIDAGNMVDTMSLIDTLERLGVSYHFETQIEQKLQQYFDLNTDYNNEAYDLYTVALHFRLFRQHGYRLSADTFRRWRDENGKFQEGLKNDAKGLLSLYEASYLRTRGETLLDDALDFTTATLKSIAPHLESPLREQVEHALIQPLHFGNPRIEARNFISIYEEYQDKNESLLRFAKLDYNQLQMLHKEELDVVSRWWKDLDLVAKLPYARDRVVECFFWAMGVYHEPQYSRARIMLTKTIAMTSIIDDTYDAYGTIEELDAFTKAIERWNIKEMKRLPDYVKPFYRALLQLYNQFEDELSKKGRSYAAHYAIESVEKDRGQIATGIESYMKDNGATKEEAVAKFFEIATDAWKDINEECMRPSPYSRDVLM

>scaffold1387.g39076.t1

MELPVKTGGSTVSVFRNRNRTKQKCPRFWFGTGSGGPVPLVPEINDAGKKQLEKQKKNMKNLLAQTPDESTAKMELIDAIQRLGIGYHFSKEIQESLQKIHETQIYKEDDDVCVVALRFRLLRQQGYRASCDVFEKFMDGEGNFKESMKKDVEGILSLYEASYYGKEQKITVIDCYIKEHGCSQMEASRELRKQVEKAWKDLNDERLKKSKVVSTEKGKRPVPHPIDHREEAGPRGPDRFENARKPLARFEDTLRSPVQGRPRDPAQWNPWRNGLHCPARTYAMAVERVLRMEVLRLLRKGQLVNHLRPRDQQVANRQDTRCLRHSPANQALLQQQSPPPPYIDRVINCISGGSEISWRVVLGLFPALTSHCFCSLPSHRLGYVLIRAVTFFYEVRAWSASIGLR

>scaffold1382.g38993.t1

MGKSFNSYQATIGMAPYEALYGRRCKLPLYWDEDCKILYERKVETKIRWAIRDSREDWDDCILFSLPPNFHVSQLRKYVRDPRHVICQEEITVTPELQYEEKPEAILDRCFISKWEINGMDQLPPYMRLYYKALSDVYFEMEYEMGKTGESHTVEHAKQEIKNLADVYFEEAKWSFNEYKPRMEEYTKVALLSSGYMMMTVNALAVIPPHFSGRVRLGFE

>scaffold11482.g84987.t1

MAMEDWPPYIQTCYKSIVDTYAGIEEQMEKEGKSYRVEYAKQELKKLVMAYFEEAKWLYRDYVPTAEEYMKVSLVSCGYMMISTSSLVGMGDLVTKQDFDWITNEPLIVRASSIICRLMDDLVGDEYEQKTASVQCYMKQYGMSKDQARDELRKQVKNAWKDMNQECLEPRPASMQILIRVVNLGRVINLLYADDDCYANPIKSKEWVKMVLLDPMDI

>scaffold845.g29034.t1

MNDVEGMLSLYEASNYGVKGEDILDKALEISSSHLKSLTSLSRRVNEALEMPISKTLARLGARKWWKKLDFANKLPFARDRVVECYFSILEVYYEPHYNIARRFLTKVIAMTSIMDDIYDVHGTLEELQYFTDAIQSWDVSAVDELPPYMTQCYEALLGVYTEMENEMIEEKITNVDCYMKENGCSKMEAADEFSKRVKEAWKDLNEECLEPRAASMVILVRVVNLACVINLLYVGEEAYGNSSDNTKELIKAVLVDPVE

>Sb01g015070

FSTLHEAALRFRLLRTHGLWVSPDELSKFRGDDGSFRTEIMNDHRCLLSLYNAAHLLTHGEVELEEAILFARQHLELGLLTSSLRAPLAGQVTRALKLPLPRTLKRLEALDYMSEYTQEQTYNPSILELAKLDFNLLQRLHLKELKAICQWWKDIYQEVELNYMRDRVVECFFWSYTVYYEQDHLRARAMLTKIFALLTVVDDTFDDHATLDESRKLAEALRRWDNSAVSMLPEYLRKFYLRLLQNFEDFEDELPPNERYRVAYTREAFQMTSESYLQESEWFHHSCKPKFQEQVNVSTVSIGPQIAATAMLMGMGDEATRDAFEWALRGDTAVMSFGWIARFLNDIASFNSGKSKKDVATSVECYMNEYNVTSEVAMTEIGYLIEDGWKTANRARFELLPAVQRIINLTVCMPFTY

>Sb01g021990

ARNSNVKDVDDTAMAFRLLRLHGYNVSPSVFKNFEKDGEFFCFVGQSTQAVTGMYNLNRASQIAFQGEDVLHRARIFSYEFLRQREAQGLRDKWIIAKDLAGEVQYTLDFPWYASLPRVEARTYLDQYGGKDDVWIGKTLYRWYIENCLDTFGVQPQDVLRAYFLAASCIYEPSRAAERLAWARTSMIANAISTHLDISADKKRLECFVHCLYEESDVSWLKINPNDAILERALRRLINLLTQEALPIHEGQRFIHSLLSLAVIEICAGRISEAISVINNKDSDWFIQLTCNTCDGLNHKVLLSQDAEKNEATINCIDKIELNMQELQLLRSDEKTTNKKTKQTLWDVLRSSYYHCPQHIIDRHVSRVI

>Sb01g032610

RNESFHEISLQFWLLRQDRYYVSCDVFQSFMDNQQNLNVSLQSDVRALLALYEAAHLGTPNEQFLIEAQRQTTSLLRSMVDHLEKPLADKVRHALQTPSFRRMKRLEARLYIPLYEEDKEDCDELILELAKLDFYLLQQIHREEVKEICEWYHGLDSPRKLFYARHRPAEAYFWALGVYYEPQYAKARKLLAKFIATITPYDDTFDNYGMWEELQPFADVMQRWDMKEAEGLNECYSDFARFMFGTMIEIENALPKDIGRRNVNFIRDIINEVCKGYVTEIGWRDSKYIPLLEEHLKVTLITCFYWAINCTAFVVFEENVTEEILKWMSKFPQIVKDSCIISRLMDDIVAHEFETERNNVATAVTCYMNEYKTTKEEASDVLWNSVEHAWKSMNHEYLSLPSSLLIRVINLARMMETMY

>Sb01g034700

GGGAADDGLHTVALRFRLLRQHGVWVPADVFDRFKDTTGGFSESLSSDPRGLLSLYNAAHMATPGEQGLDEAISFARRHLESLKLKGTLSSPLAEQVCRALDIPLARLPKRLETMHYVVEYEKEEGHDAVLLELARLDFDLVRFLHLRELKDLSLWWKDLYGNVKLNYARDRLVENYFWTCGVFHEEEYSRARMLFAKTFGLLSLMDDTYDVYATLEECHILNDAIQRWDENSASILPEYMRMFYINLVRNFQGFEDSLQPNEKYRVSYAKQAFKLSSKYYLDEAKWCSEKYAPSFKEHMEVSVMSSGFPTLAVVLLMGAGDMATREAFRWAIGVPAVVSASGEVARFRNDIASYKKKNKKDVASSVECYAKEHGTSGEEAAVAIAGMAEHAWRTINRSCMLLPAAQLVVNLTKTLEVIY

>Sb01g035460

NSTCLHDVALRFRLLRQHGFWISPDEFNRFKDKNGNFDVDITNDARGLLSLYNAAYLFTHGEAELEEAILFARQHLESMRNNLEYPLAQLVNRALHLPLPRTFRRVEALHYISEYKGTPTHNPSLLEFAQLDFDLLQRLHLKELKALSRWWKDLYNEGELTYSRDRVVECYLWSYTAYFEKEHTRARMILAKIIALIILTDDTYDVRATLEECRKFNEAIQRWEESAISLLPDYLKKFYLKLMNIFKEFEDELEPHEKYRVAFSRKAFQILSSNYLQEAEWFHGGYKPTFKDQVKISTVCSGAPFASVGLLVGMGDVATKEALEWAISCTDAVKAFAEVTRFMNDLASFKRGKNKNDVDSSVECYISEHGVTTEVAFAKINSLIEEAWKTINRARFELLPAVQRVANITASMPLMY

>Sb01g039090

DVDRVDDVRLMTLSFRLLRQNNYPVSPEVVRSLKDGTGNFKKTLQKDTEGLLSLYEASHLAFEGDHLLDEARVFSTEALRELRPSMHPHLRSSVDNALAVPLHWAAPRLQARWFINHYARDSDADLSLLHFAKLDFNNVQKLQQQELSRITRWWRNADLSESLPFARDRLMECFYFATGVAPEPSLEACREVVAKTFALIVLLDDIYDIYGTLDELVMFTDAIERWGTSASEQLPEYMKAIYLTIVSTSNEVAEHVLRQEGCDARFLLKKAWHDLCKAFLMEAKWHYSNYKPTLHEYLENGWISVSGPLMLIHAFPIIEKGVTPNSIQQLESYPKLVQMVSKIFRLCNDSATHSEELKRGDAPSSIAIYMSENRATEHDARKAMRDLTMETWKTVNQDAFRFPLPFANACVNMARISHCIY

>Sb04g001780

HSDDLFDATLAFRLLREAGHDVSADVLRRFTDDSGEFKLPLSMDIRGLLSLHDMSHLDIGGEVLLYKAKEFSSKHLASAIRYLEPSLAEYVRQSLDHPYHLSLMQYKARHHLTYLQSMPIRDTVVEKLAVAEFQLNKLLHQQEIQEVKRWWMDLGLVQEIPVVRDQVMKWYMWSMTAVQGCSFSRYRVEITKIIALVYVVDDIFDLVGTLEELSLFTEAVKVWNMAAADSLPRCMRSCYMALYTVTNEITDMVEKEHGLNHVNHLRKAWAVLFDGFMVEAKWLATEQVPTAEDYLRNGVVTSGVPLTFVHIFIMLGDQSIEALIDQMPSVISCPAKILRLWDDMGSAKDEAQEGLDGSYMDFYLMENRCGPSDVEAHMRNLIAREWEELNRECLTFSSNLTQTCLNAARMISVMY

>Sb04g001800

HSDDLFDATLAFRLLREAGHDVSANDVQRFTKDSCEFKLPLSKDIRGLLSLHDMSHLNIGGEALLYKAKEFSSKHLASAIRYLEPSLAEYVRQSLDHPYHLSLMQYKARHHLTYLQSLPIRDTAVEKLAVTEFQLNKLMHQKEIQEIKRWWMDLGLVQEIPIVRDQVLKWYMWSMTTLQGYSFSRYRVEITKIIALIYVVDDIFDLVGTLEELSHFTEAVKVWNTAAADSLPSYMRSCYKALYTITNEIADMAKQEHGLNPVNHLRKAWVVLFDAFMVEAKWLTMDQVPTAEDYLRNGVVTSGLPLTLVHIFIMLGDQSTETLIDHMPSVISCPAKILRLWDDMGSAKDEAQEGIDGSYRDFYLMENRCGPSDAEAHMRSLIAREWEELNRECLTFSSNFTHACLNITRMISVMY

>Sb04g001810

SNDLFDATLAFRLLREAGYDVSADVLWRFTDNSGEFKLPLSKDIRGLLSLHDMSHMNIGGEALLDKAKEFSSKHLASAIRYLEPSLAEYVRQSLDHPYHLSVMPYKARHHLTYLQSLPTRDTAVEKLAIAEFQLNKLLHQKEMQEIKRWWMDLGLAQEIPVVRDQVLKWYMWSMTALQGYSFSRYRVEMTKIISLVYVVDDIFDLVGTLEELSLFTEAVKMWNTAAADSLPSYMRSCYKALYTITNEIADMAEKEHGLNPANNLRKAWTVLFDGFMVEAKWLANHQVPTAEDYLRNGVVTSGVPLTFVHIFIMLGDQNSEALIDHMPSVISCPAKILRLWDDMGSAEDESQEGFDGSYKNFYLMENRCSPTDAEAHMRSLIAREWEELNRECLTFSSNFTQVCLNIARMVSVMY

>Sb05g006470

GGDDLFALALQFRLLRQHHYNVASEIFNNFMDENGDFKDALRSNVDGLLSLYEAAHLGKSDEDLLRKAIIFTKDCLSSLVNGGQLPKPVLQEVLHALDLPTQRRIKRLEAKLYISIYENGDESNQDIVELAKLNFHMLQQMHRDEVRTISLWWYNDLNPSSLGPYMRKRPVECYYWALGIFYEPQYAKARIVLTKLLTLLTMFDDIIDSYGTMEEVHLFNQAVQSWNEEAAKQIGDGYWYLIFHISKTLEEFVSKDGGSPMAIDCFKETLKAGSKAMVQELVWREEGQVPTVHEYLKQGAAVSILYWPIAVISFAGMFPSDDEIFTWAGSYPKIIESSTTLCRLMDDVAGHENEEERSKCVTAVECYVREHGVTVQEAKQALTCLVDEQWRCINQELYAVPIALLDPVLDLVRVMEEVY

>Sb05g019210

EILRSEELHIVALRFRLLRQHGFFVSTDVFDEFRDGTGNFNTCLTRDPKGLLSLYNAAYLAVPGEDVLDGVIAFTRTHLEAMKGNLTSPIADQICRALDIPLPRYMPQLETMHFITEYEQEDGHNATLLELARLDYCLTRSAQLKELRTFCLWWKDFYKNVNLTYSLDRGVEMYFWGFGVFPGEGNSRARIIFSKIVALISLMDDTFDTHATFEDCKNLDEAIQRWDESATSILPEYLRMYYTKMLSCFNEFEDILEPKEKYRVPYVQKAVMLQSKYYLEEAKWCNEKYMPTFKDQIELSSLSSTIPVLTLAALMAAGNEATKEALEWASVVPDMKGRKNKNDVASSLDCYMNEHGTTGTEAAAALSAMVEHAWRRINKAFMEIDRALLRAAVINQARTNEVVY

>Sb05g022320

ATDVLSLFAEESRFHDSVEGHMNDTKALLELYKASLVEYALNLPFYATLQPFKHKRNIECFGTEGIRIHKSAYLACDATENILALAIEDFHLSQSIYQQQLQYIERWVKEVRLDQLKFARDLPLSLFVFLATNVFPCELYDASIAWTQKCILTTVVDDFFEGGGSTKELRNFVTLIEKWDMHAGIECSEDIEILFRAVYDTNNQIAAIGAKLQNRSVIDHIVEIWVKYVRTLMIEAEWTTKGHVPTMEEYMSVAETSSALGPVVVPSLYLVGPKLSDDMIRDPEYKNLLRYLGIGIRLINDIGTYEKEMSEGYVNSVLLRAFDSSIEAAKREIHVLLANSQRELLKLVLPIPRPCKDIFWNTYKIGRQFY

>Sb06g002820

KDHTYDNIAFTALKFRLLRENGFPEGQLGYHNYGNCTTKTPRQEDVNTLLLLHEASYLAFGDEEILDVARTYSAKALKELMPSMLPHLREAVAHALEIPLHWRAPRLETRWFIDYYARDINMCPLLLQFAKLDFNQVQDEHQKDLAAVTWWWRNIGLGEKLPFARDRLMECFHYANGIVWDPKLGPCRQMLAKVSNLIVHLDDVYDVYGTMDELVLFTNAIARWDAIPNERLPEYMKALYSVIYHTSNEVAEHALKEHGCSMHYHLQKLWHDICMAFLLEAKWHHGNCRPSIQKYLENGWVSSSAPLLLSHAFSMLHSVINMNTISKMQTTHRLVQQVSLIFRLCNDSATHMDELQRGDAPSFIAINMAENGNEDDSRKVMQDLILKSWKVINEEAFQYSTPFNKACVNLARISHCVY

>Sb06g028210

RDEEIMLDAATCAMAFRILRMNGYDVSSDELYHVAEASMFHNSLGGYLNDTRTMLELHKASTVSTSEDEYILDTIGSWSSTLLREQLGSGGALRRTPLFREVEHALDCPFYTTLDRLDHRWNIENFNVTGHRMLETPYLSSRHTSRDILTLAVRDFSSSQFKYQQELKHLESWVKECKLDQLPFARQKLAYFYLSAAGTMFPPELSDARILWAKNGVLTTVVDDFFDVGGSKEELENLLLLVEMWDEHHKIEYSEQVEIVFSSIYNSVNQLGAKASLLQDRNVTKHLVQIWLDLLKSMMTEVEWRMSKYVPTEEEYMANASLTFALGPIVLPTLYFLGPKIPKSAIKDPEYNELFRLMSTCGRLLNDVETFEREYNEGKLNSVSLLVLHGSMSISDARRKLQKPIDTCRRDLLRLVLVIPRPCKELFWKMCKVCYFFY

>Sb06g028220

KDEEIMMDKATCAMAFRLLRMNGYDVSSDVLSHVAGPSTFHDSLQGYLNDTKSLLELYKASKVSLSENDLVLDGIGFWSGNLLKDKLCSSRVKKDLIFEMEYAVKFPFYATLERLEHKRNFDAWGPLMLTTKSSSFCIDQEFVALAVEDFSFSQYVYQDELRHLDSWVKENKLDQLQFARQKLTYCYLSAAATIFSSELSDARISWAENGVLTTVVDDFFDVGGSKEELENLIALVEKWHAHHTVGYSEQVKIVFSAIYTTVNHLGVIASAAQGRDVTNHLVEIWLDLLRSMMVETEWQRSQYVPTVEEYMTNAVVSFALGPIVLPALYFVGQEVLEHAVEEYDELFRLMSTCGRLLNDSQSFEREGNQGKLNSVSLLVRHSGMSIEAAKKALQKSIDVSRRDLLRLVLVVPRPCKELFWKMCKILHLFY

>Sb06g031270

LHDVALRFRLLRQQGFWVSPDEFNRFKDKHGNFDVGITNDARGLLSLYNAAHLFTHGEAELEEAILFARQHLESMRNNLEYPLAQQVNRALLVPLPRTVRRLEALHYISEYKESPAHDPSLLEFAQLDFDLLQRLHLKELKALSRYNQYLAIRIVLTFLGLNNVNTTHRYCSAAYYEKEHSRARMILAKIIALIILIDDTYDVRATLEECRKFNEAIQRWEESAISLLPDYLKTFYLKLMNIFKEFEDELETHEKYRVAFSRKAFQILSSNYLQEAEWFHGGYKPTFEDQVKISTVCSGAPFASVGLLVGMGDVATEEALEWAISCTDAVKAFAEVTRFMNDLASFKHGKNKNDVDSSVECYISEHGVTTEVAFAKINSLIEDAWKTINRARFLPVVQRVANITASMPLIY

>Sb07g003080

GASASSHDLYVTSLRFYLLRKHGYTVSSDVFAKFRDEQGNISSDDDMTTLMMLYDAAHMRTRGEDILDNIIVFNKSRLETVVKSENLEPDLAEEVTITLETTRFRRAERVEARRFISVYEKKATRDDTILEFAKLDYNIVQAVYCDELKQLSMWWKDLRSQVDMTFSRDRLVEMYFWMTVIVYEPDYSYSRIMLTKLVLYIALLDDIYDNYSTTDESNIFTTAFKRWDDKAVEEIPQHLRNFYKSVIRTADEIVAELKVQNNKNSEVVREVMFHVAESYHAEVKWRDEQYVPADVDEHLQISLGSIMAMQVVVLTFVSMGDVTTREIIDWAFTYPRMIRAVTAMARILNDIMSYEREQASDHMASTVQTCMKQYGVTVEEAIEKLKFICEEAWMDIVQGCLYPMAILHKVVSVGRSLDFIY

>Sb07g004470

DVKDLNLVSLRFYLLRKNGYDVSSDVFLNFKDKDGNFASDDIRSLLSLYNAAYLRTHGEEVLDEAIIFTRRHLEAALTSLESKLADEVSLSLQTPLFRRVRILETRNYIPIYEMEPSRNEAMLEFAKLNFNLLQILYCEELKTVTAWWKQLNIETDLSFIRDRIVEMHFWMAGACSEPKYSLSRVILTKMTAFITILDDIIDTHSTTEEGKLLAKAIDRCSQDANEVLPDYMKHFYMFLLKTFDSCEDELGPNKRYRLKILVRGYSQEIEWRDEHYVPETIDKHLEISRVTVGAFQLACSSFVGMGDIITKEVLDWLLTYPELLKCFTTFVRLSNDITSTEHSTTMHDACEKIKGLIEDSWKDMMQLYLEQSKVVAQTVVDFARTGDYMY

>Sb07g004480

DDKDLNLVSLRFYLLRKHGYYVSSDVFTSFKDKEGNFVADDTKCLLSLYNAAYLRTHGEKVLDEAIIFTRHQLEALLDSLESTLADEVSVTLQTPLFRRVRILETRNYIPIYEKEAARNEVILEFAKLNFNLLQLIYCEELKKVTLWWKQLNVETNLSFIRDRIVECHFWMTGACFEPQYSLARVISTKMTACITILDDIMDTYSTTEEAMLLAEAIYRWEENAAELLPEYMKDFYLYLLKTIDSCDNELGPNKSFRTFYLKEVLKVLVRGNSQEIKWRNENYVPETINEHLEHSGRSVGAFQVACSSFVGMGDNITKEILEWFLTYPELLKSFTTIARLSNDIASTKREQNVGHHVSTVQCYMLKHGTTMDDAYEKIKELIEDTWKDMMELYLEQPKLVTQTVVDFARTADYMY

>Sb07g004485

DDKDLNLVSLRFYLLRKHGYDVSSDVFKCFQDKEGNFVVKDTKSLLSLYNAAHLRIHGEEVLDEAIIFTRGKLESVLDSLETTLADEVTLALQTPLFRRVRILETRNYIPIYEKEVARNEVILEFAKLNFNLLQLLYCEELKMITLWWKQLNVETNLSFIRDRIVEMHFWMTGACSEKKYSLTRTITTKMTAYITILDDIMDTHSTTEEAMLLAEAIYRCEENAAELLPEYMKDFYLYLLKTFDSVKHELGPNRSFRVFYLKELLKILVRGYSQEIKWRDEHYVPETIDEHLEVSKATVGAFQVACSSFVGMGDIITKEILDWLLSYPKLLKSMTTFVRLSNDIASTKREQTGGHHASTVQCYMMQHGTTIHDACEKIKELTEDTWKDMMKLYLEQPKVIIQTVLDFARTAEFMY

>Sb07g005130

QDLDLPTTSHLFYLLRKHGYHISSDVFLKFRDDKGNIVTDDARCLLLMYEAAHLRVKGEEILDNILIFTKSQLQCIVDDLEPQLKEEVKYALETPLFRRLKRVQTRQYISIYEKNTAHNNMLLEFSKLDFNILLTLYCEELKDLTLWWTEFQTQANTSIYARDRMVEMHFWMMGVFFEPQYSYSRKMLTQLFMIVSILDDLYDNHCTTEEGNVFTAALERWDEEAVEQCPTYLRTLYVNILTTVKAIEEWLNLQNNKHAKLVKRLIIDMAKCYNAETEWRDKKYVPATVDEHLKISARSSGCMHLVSQGFISMGDVATSEAIKWASTYPKIIQAVCIIARLANDIMSYKREETSQNMVSTVKTCAKEYGTTVAQAIEKLRELIEEAWMDITEECLQQPKVLLERVANLARTMDFLY

>Sb07g020980

NSSSLHEVALRFRLLRQQGFWVSADEFEKFKNEDGSFISGITNDPKGLLSLYNAAHLLTHDEEILEDAILFSRQHLELIRSSLKSPLAEQVGRALEIPLPRTLKREETISFIPEYSIQDQTYSPVILELAKLDFNLLQHLHQKELKEISQWWKELSGEIGLDYVRDRIVECYFWSYTVHYEQENARARMILARLFLLTSLLDDTYDVHATLEEARELNKAIERWDDNDVSLLPEYLKEFFVKVISNFREFEDELESHEKYRNVYNIKGFQTLSKYYLQEAEWFHHGYTPSFKDQVNVSVITAGGQVLSIGLLVGMGEATKEAFEWATGDTDAIWACGQVSRFMDDMSAFKNGRNNMDVASSVECYMKERNVPSEVALATISSFVEDAWKTINQAKYPTLLPVVQRVTSLAKSMTLLF

>Sb07g025700

DNFHGSNDLHVVALRFGLLRQHGLWVSADVFDKFRDAMGSFSMDLATDSKGLLSLYNAAHMAVPGEAVLDDAVAFARRHLEAAKGKLIRSPMVEQVSRALNTPRPRWPRRLEAMHYITEYEQEDEHNAIILELARLDFSIVRSVYIEEIKNLSLWWRDLYNDVKLPYARNRIVETHLFSSGVFPEKEHSRARIIFTKTFAFLSLMDDTYDTHATLEECQKLTEAIQRHEYLRMFYIKLLRNYKEIEEDILEPWEKNRMADFKKSFKLVSKSYLKEAEWFSQNYTPSFKEHIDFSITSTGLPMLSHVALMGAGQLATKEAFDWALDMPDLVKGMAETGRFFNDISSYKPRNSLKDVVSSLECYMKEHDMTPNDATVAFETMVEHAWRRINKAYMELDHGILPAVVNMARTVQMFY

>Sb09g000990

SDYDLGTVALWFCLLRKHRYRVSSDVFVRFKDEKGGFLVDSPQDLLNLYNAAHMRTHGEVILEKAILFSQRRLETMIPYMEGSLLAEIKSALEIPLPRRVRIYELKYYISTYEKDATVHEKVWQLAKLNSNIMQLHHQHELGIITSWDSKGAHDLPECMKFALEKIFDSYETIENMLHQEEKYRMAYLRYFVKDLVRSYSKEVKMLQEGYIPKSVEEHLKVSVITTTCPFLSCASFVGMHDIATKDFFDWVSSVPKMVQELSVILRLVDDLGSYEREQLIPHVASTINSYMKEHNVSIEVARGQIQVLKEKSWKDFNSEWLAYPKQLLERIFNFTRTMEFIY

>SollyTPS10

MASSSSTNKSRPLANFHPTVWGYHFLSYTPQFTEITNQEKVEVNEYKERIRKMLVKAPEGSLQKLVLIDAMQRLGVAYHFDNEIETSIQNIFDASSKQNDNDNNLYVVSLRFRLVRQQGHYMSSDVFKQFINQDGKFKETLTNDVQGLLSLYEASHLRVRDEEILEEALTFTTTHLESIIVSNLSNNNNSLKVEVSEALTQPIRKTLPRVGARKYISIYENNVAHNHVLLKFAKLDFNVLQKLHQRELNELTRWWKDLDFANKIPYARDRLVECYFWILGVYFEPKYSRARKMMTKVLKITSVIDDTFDAYATYDELVAFTDAIQRWDASAIDSISPYMRPLYQALLDIYSEMEQVLSNEGKLDRVYYGKHEIKKIVRAYFKEAQWLNDANYIPKYEEHMEISLVTAGYMMGATNCLVGVEEFISKDTFEWLKNEPLIVRAASLISRAMDDIVGHEDEQKRGHVASIIECYMKEYGASKQEAYAKFKKEVTNVWKDINKEFFRPTEVPMFVLERALNFARVIDTLYQEVDGYTNSKGLLKDLVNSLLIESVKISIS

>SollyTPS12

MASSSANKCRPLANFHPTVWGYHFLSYTHEITNQEKVEVDEYKETIRKMLVEAPEGSEQKLVLIDAMQRLGVAYHFDNEIETSIQNIFDASSKQNDNDNNLYVVSLRFRLVRQQGHYMSSDVFKQFINQDGKFKETLTNDVQGLLSLYEASHLRVRDEEILEEALTFTTTHLESTVSNLSNNNSLKAEVTEAFSQPIRMTLPRVGARKYISIYENNDAHNHLLLKFAKLDFNMLQKLHQRELSDLTRWWKDLDFANKYPYARDRLVECYFWILGVYFEPKYSRARKMMTKVIQMASFFDDTFDAYATFDELEPFNNAIQRWDINAIDSVPPYLRHAYQALLDIYSEMEQALAKEFKSDRVYYAKYEMKKLVRAYFKEAQWLNNDNHIPKYEEHMENAMVSAGYMMGATTCLVGVEEFISKETFEWMINEPLIVRASSLIARAMDDIVGHEVEQQREHGASLIECYMKDYGVSKQEAYVKFQKEVTNGWMDINREFFCPDVEVPKFVLERVLNFTRVINTLYKEKDEYTNSKGKFKNMIISLLVESVEI

>SollyTPS14

MATNLTLETDKEIKNMNQLSMIDTTITRPLANYHSSVWKNYFLSYTPQLTEISSQEKLELEELKEKVRQMLVETSDKSTQKLVLIDTIQRLGVAYHFDNEIKISIQNIFDEFEQNKNEDDNDLYIVALRFRLVRGQRHYMSSDVFKKFTNDDGKFKETLTKDVQGLLNLYEATHLRVHGEQILEEALSFTVTHLKSMSPKLDSSLKAQVSEALIQPIYTNVPRVVAPKYIRIYENIESHDDLLLKFVKLDFHILQKMHQRELSELTRWWKDLDHSNKYPYARDKLVECYFWATGVYFGPQYKRARRMITKLIVIITITDDLYDAYATYDELVPYTNAVERCEISAMDSISPYMRPLYQVFLDYFDEMEEELTKDGKAHYVYYAKVEMNKLIKSYLKEAEWLKNDIIPKCEEYKRNATITVANQMILITCLIVAGEFISKETFEWMINESLIAPASSLINRLKDDIIGHEHEQQREHGASFVECYVKEYRASKQEAYVEARRQIANAWKDINTDYLHATQVPTFVLQPALNLSRLVDILQEDDFTDSQNFLKDTIKLLFVDSVNSTSCG

>SollyTPS16

MELCTQTVPADHEVEITRRVGSHHPTVWGDHFLAYANLSGASEEEEKQHEDLKEEVRKMLVMAPSNALEKLELINTIQCLGVAYHFEHEIESYMCTHYEEYWIDDLHAIALCFRLLRQQGYRVSCDAYKKFTDDQGNFKIELINDVHGMLSLYEAAQFRVHGEEILDEALNFTTTQLKLILPKLSNSPLAQQVANALKFPIKDGIVRVEARKYISFYQQNQNHNQLLLNFAKLDFNILQMLHKKELCDITRWWKELEIVKTLPYVRDRLAEVYFWSLGVYFEPQYSTARKILTKNISMISLIDDTYDIYGTLDELTLFTEAIERWNIDASQQLQLPSYMKIIYCGLLDVYDEIKKDLANENKSFLINYSIIEMKKMVMAYFQEAKWYYGKTIPKMEEYMKSGISTSAYVQVATTSWLGMGNVATKDSFDWIVNEPPILVASSIIARLLNDLLSHEEEQKRGDAPSGVECYMKEYGVTKEEAHIKIRNTIENSWKDLYEEYFKVNGTIIPRVLLMCIINLARVIEFIYKDEDAYTFPKNNLKDVIYRILIDPII

>SollyTPS17

MELCTQTVAADHEVIITRRSGSHHPTLWGDHFLAYADLRGANEGEEKQNEDLKEEVRKMLVMAPSKSLEKLELINTIQCLGLGYHFQSEIDESLSYMYTHYEEYSIGDLHAIALCFRLLRQQGYYVSCDAFKKFTNDQGNFKEELVKDVEGMLSLYEAAQFRVHGEQILDEALNFTIAQLKQILPKLSNSQLAQQITNALKYPIKDGIVRVETRKYISFYQQNQNHNEVLLNFAKLDFNILQTLHKKELSDMTRWWKKMELVNTLPYARDRLVECYFWCLGTYFEPQYSVARKMLTKISFYISIIDDTYDIYGKLDELTLFTQAIERWNIDASEQLPLYMKIIYRDLLDVYDEIEKELANENKSFLVNYSINEMKKVVRGYFQEAKWYYGKKVPTMEQYMKNGISTSAYILLTTTSWLAMGNVATKDAFDWVATEPPIVVASCYIIRLLNDLVSHEEEQKRGNAASAVECYMNEYSVTKEEAHIKIRDIIENYWKDLNEEYFKVDMIIIPRVLLMCIINLTRVAEFIYKDEDAYTFSKNNLKDVISDILVDPII

>SollyTPS18

MIIGYRINFRPLSHDKLRSHVMWQRQCSYNTASSMDGFEEAKERIRESFSKVELSPSSYDTAWVAMVPSKYSLNEPCFPQCLDWIIENQREDGSWGLNPTHPLLLKDSLSSTLACLLALTKWRVGDEQIKRGLGFIETQSWAIDNKDQISPLGFEIIFPSMIKSAEKLNLNLAMNKIDSTIKRALQNEFTRNIEYMGEGVGELCDWKEIIKLHQRQNGSLFDSPATTAAALIYHQHDQKCYEYINSILQQHKNWVPTMYPTKIHSLLCLVDTLQNLGVHRHFKSEIKKALEEIYRLWQQKNEEIFSNVTHCAMAFRLLRMSYYNVSSDELAEFVDEEHFFSTSGKFISDVAIIELHKASQLTINEKDDILDKINNWTGIFMQQKLLNNDFLDIKSKKEVELALRMFYVTYDRAENRRYIESYQENNFKMLKTAYRCGSMNNIDLLTFSMQEFELGLSQYQEEVEQLKRWYEDYRLEQVGLAQEYIYRTHLISVAVFFEHELSNARIMYAKYAMFLTLSDDLFEHLASKDELLNIIELVQRWDEHTNVGFHSEKVKLFFTALYDTIEEVATNAQIKQGRNVKHHIIELFVEGLNSMLVDRVEWGTRIPSIEEYLRVSLSTFGGKCMVLTSQYVVGIHLCNYQSDDEIQDLCYCSGIVMRLLNDLQSFKRERSDSRLVNMVKLVMKQRSGTICEEEEEEAIKHIKETIECNRRKLLRMVLQSKGKGSKVPQALKDLFWRTTKAVYFFYSDHDEFRSPNKVKHHINQVIYKPLHNR

>SollyTPS19

MIVGYRSTIITLSHPKLGNGKTISSNAIFQRSCRVRCSHSTTSSMNGFEDARDRIRESFGKLELSPSSYDTAWVAMVPSRHSLNEPCFPQCLDWIIENQREDGSWGLNPTHPLLLKDSLSSTLACLLALTKWRVGDEQIKRGLGFIETYGWAVDNKDQISPLGFEVIFSSMIKSAEKLDLNLPLNLHLVNLVKCKRDSTIKRNVEYMGEGVGELCDWKEMIKLHQRQNGSLFDSPATTAAALIYHQHDQKCYQYLNSIFQQHKNWVPTMYPTKVHSLLCLVDTLQNLGVHRHFKSEIKKALDEIYRLWQQKNEQIFSNVTHCAMAFRLLRMSYYDVSSDELAEFVDEEHFFATNGKYTSHVEILELHKASQLAIDHEKDDILDKINNWTRAFMEQKLLNNGFIDRMSKKEVELALRKFYTTSHLAENRRYIKSYEENNFKILKAAYRSPDINNKDLLAFSIHDYELCQAQHREELQQLKRWFEDYRLDQLGLGERYIHASYLFGVTIIPEPELSDARLMNAKYAMLLTIVDDHFESFASKDECLNIIELVERWDDYASVGYKSEKVKIFFSTFYKSIEELATIAEIKQGRSVKNHLINLWLEMMKLMLMEQVEWCSGKTIPSIEEYLYVTSITFCAKLIPLTTQYFLGIKISKDLLESDEICGLWNCSGRVMRILNDLQDSKKEQKEGSITLVTLLMKSMSEEEAVMKTKEILEMNRRELLKMVLVQKKGSQLPQLCKDIFWRSSKWAHFTYSQTDGYRIPEEMKNHIDEVFYKPLNH

>SollyTPS20

MIVGYRSTIITLSHPKLGNGKTISSNAIFQRSCRVRCSHSTTSSMNGFEDARDRIRESFGKLELSPSSYDTAWVAMVPSRHSLNEPCFPQCLDWIIENQREDGSWGLNPTHPLLLKDSLSSTLACLLALTKWRVGDEQIKRGLGFIETYGWAVDNKDQISPLGFEVIFSSMIKSAEKLDLNLPLNLHLVNLVKCKRDSTIKRNVEYMGEGVGELCDWKEMIKLHQRQNGSLFDSPATTAAALIYHQHDQKCYQYLNSIFQQHKNWVPTMYPTKVHSLLCLVDTLQNLGVHRHFKSEIKKALDEIYRLWQQKNEQIFSNVTHCAMAFRLLRMSYYDVSSDELAEFVDEEHFFATNGKYKSHVEILELHKASQLAIDHEKDDILDKINNWTRAFMEQKLLNNGFIDRMSKKEVELALRKFYTTSHLAENRRYIKSYEENNFKILKAAYRSPNINNKDLLAFSIHDFELCQAQHREELQQLKRWFEDYRLDQLGLAERYIHASYLFGVTVIPEPELSDARLMYAKYVMLLTIVDDHFESFASKDECFNIIELVERWDDYASVGYKSEKVKVFFSVFYKSIEELATIAEIKQGRSVKNHLINLWLELMKLMLMERVEWCSGKTIPSIEEYLYVTSITFCAKLIPLSTQYFLGIKISKDLLESDEICGLWNCSGRVMRILNDLQDSKREQKEVSINLVTLLMKSMSEEEAIMKIKEILEMNRRELLKMVLVQKKGSQLPQLCKDIFWRTSKWAHFTYSQTDGYRIAEEMKNHIDEVFYKPLNH

>SollyTPS21

MLIGCRSKIIIISHHKLGNGKTISSNAIFQRSCRARCSHSTTSSMNGFEDARDRIRESFGKLELSPSSYDTAWVAMVPSRHSLNEPCFPQCLDWIIENQREDGSWGLNPTHPLLLKDSLSSTLACLLALTKWRVGDEQIKRGLGFIETYGCAVDNKDQISPLGFEVIFSSMIKFAEKLNLNLPLNLHLVNLVNCKKDSTIKRNDEYMGEGVGELCDWKEIIKLHQRQNGSLFDSPATTAAALIYHQRDQKCYEYLNSILQQHKNWVPTMYPTMIHSLLCLVDTLQNLGVHRHFKSEIKKALDEIYRLWQQKNEEIFSNITHCAMAFRLLRMSNYDVSSDELAEFMDEEHFFTTSGKYTSHVEILELHKASQLAIDQEKDDILDKINNWTRTFMEQKLLNNGFIDRMSKKEVELALRKFYTTYDRAENRRYIKSYEENNFKILKAAYRSPNINNKDLLIFSIHDFDLCQTQHREELQQLKRWFQDCRLDQLGLSEQFISTTYLIGIAVVSEPEFSNARLMYAKYVMLLTIVDDLFDGFASKDELLNIIQLVERWDDYASVGYNSERVKVFFSVFYKSIEELATIAEIKQGRSVKNHLINLWLEVMKMMLIERIEWWTSKTIPSIEEYLYVTSITFGSRLIPLTTQYFLGIKISKDLLESDEIYGLCNCTGIVMRLLNDLQTYKREQGESSMNLVTILMTQSPRRTNICEEEAIMKIKEILEMNRRELLKMVLVQKKGSQLPQLCKDIFWRTSKMVYFTYSHGDEYRFPEEMKNHIDEVIYKPLNH

>SollyTPS24

MSATIIFPAASSSSSYLSVVKHQMIRDITIPSRRLGGGLSFTQHSSSTAACVVDATRGPDFALQCNETTKERIRKLFHKVEFSVSSYDTAWVAMVPSPHSAKVPCFPECLHWVLHNQLEDGSWGLPHHQPLLLKDVLSSTLACVLALKRWGIGEQLISNGLRFIELNFASATDEDQYSPIGFDVIFPGMLEYAQHLSLKLHLESGVFNELLHKRAIQLTRPYDSSSLELNAYLAYVSEGIGELQDWKMVMKYQRKNGSLFNSPSTTAASLIHLHDSGCLDYLRGALKKFGNAVPTIYPINIHASLCMVDDLKKLGICRHFSEEIQNVLDETYRCWLQGEDEIFTSAGTCSMAFRILRGYGYNVSSDPVAQFLEQEQYSGHLNDIHTMLDLYQALEMIIATDKPVSMKLNSSSLQSLIQRLSDEFYPPNGLTKQIREQVDDVLKFPSHANIKRVANRRNIKHYDVDNTRVLKTSYSSSNFGNKDFLTLAVEDFNLCQSIHRNELKQLERWLTQNRLDKLKFVRERSAYCYFSAAATIFQPELSDARMSWAKNGVLTTVIDDFFDVGGSMEELNNLILLFKKWDVDVSTDCCSERVGIIFSALHSTISEIGDKASKWQARSVTRHITDIWLNLLNAMLREAEWAKDMSVPSLDKYMANGYVSFALGPIFLPALYFVGPKLPDDVVQHPEYHSLFELVSTCGRLLNDIRSFERESKDGKLNAVTLSVTHGNGRISEEAAIEGLSHRVEMQRKELLKLVLQREGSVVPNACKDLFWEMSKVLHQFYIKDDGFSSMGMADTVNAIIHEPITLNYLGDSKLITDYN

>SollyTPS25

MACINMVSIASTMQTQKLHSTTEKDSQPERISTYKPNIWKYDHLLSLTNQYSEAKYKIEAEKLKEEVGCMFSNTTSPVAQLQLIDGIDKLGLSAYFEVDTKETLENIILYMKTSSTSKDLYATALCFRLLREHGYHASQDMLKDLFDGKGKLPLDMKTSLELFEGSHLSIDGENLLNDIRLFSTKNLKNLSLDVDRLTSNPLAWRVRWYDVRKHIITAQNCNDTNPMLLKLAKLNFNIIQATHQKDLKDVIRWWRNVSIIENLEFTRERIVESFFFAVGIASEGEHGSMRKWLAKVIQLILIIDDVYDIYGTLADVQQFTVAIEKWDPEEVQRLPKSIQICFGALHDTMEDISVEIQRQKGGPSVLPHLKQVWVNFCKALLVEATWYHKGHIPTLEDYLHNGWTSSSGPLLSLHVILGLTNENLHLCKNCQEIIYYTSLIIRLCNDQGTSTVELERGDVASSIICYMHQENVSEDVAREHIESIILNSWEKTNYHFNRLSTSHRKIMKHVINEARMAHVMYLSGDGFGVQDGETQDQVLINLFRPLGRNPGHHFGRLYRPNFIVMGLGPVIFEVPI

>SollyTPS27

MTSEQQSVFCNQIHSTTSFRKSNIDETLIQRRNANYKPNIWKYDILQSLKSQYSECKYKKEAQKLKEEFLWVVAEIENPLAKLELIDSINKMALSHLFDKEIMVFLQNMEKLKDSDNEMDLYSTALYFRIFRQYGYNVTQDVFLSYMDEMGEKINVDTNMDPKTMMQLFEASHLALKDENMLDEARIFCTNNLKNIIPMEMPLHWKVEWYNTREHISKQANEKEEGVSKLKLLQLAKLNFNMVQAEHQKDLVHILRWWRNLGLIENVSFSRDRIVESFLWSVGVAFEPQHSNFRNWLTKAITFIIVIDDVYDIYGTLQNLQLFTDAVVRWDPKVVEQLPSCMQICFWKLYDTTNDVALEIQQQKGCKFPVLTYLQKVWAEFCKALLVEAKWDSKGYTPTFSEYLENGWKSSGGTVLSLHVLLGLAQDFSQVDYFLENERDLIYYSSLIIRLGNDLGTSTAELERGDVSSSILCYMRKENVKEDVARKHIEEMVIETWKKMNRHCFENSSPLIKYIMNIARVTHFIYQNGDGFGVQDRETRQQILSSLVQSLPLN

>SollyTPS28

MSLLEGNVNHENGIFRPEANFSPSMWGNIFRDSSKDNQISEEVVEEIEALKEVVKHMIISTTSNAIEQKIHLIDTLERLGIYYHFEKEIEDQLSKMFDQNLIHEEDDLYKVALYFRLFRQHGYPISSDCFNQFKDTKGKFKKTLLIDVKGMLSLYEAAHVREHGDDILEEALIFATFHLERITPNSLDSTLEKQVGHALMQSLHRGIPRAEAHFNISIYEECGSSNEKLLRLAKLDYNLVQVLHKEELSELTKWWKDLDFASKLSYVRDRMVECFFWTVGVYFEPQYSRARVMLAKCIAMISVIDDTYDSYGTLDELIIFTEVVDRWDISEVDRLPNYMKPIYISLLYLFNEYEREINEQDRFNGVNYVKEAMKEIVRSYYIEAEWFIEGKIPSFEEYLNNALVTGTYYLLAPASLLGMESTSKRTFDWMMKKPKILVASAIIGRVIDDIATYKIEKEKGQLVTGIECYMQENNLSVEKASAQLSEIAESAWKDLNKECIKTTTSNIPNEILMRVVNLTRLIDVVYKNNQDGYSNPKNNVKSVIEALLVNPINM

>SollyTPS3

MSIFSTRYLVTPFSSFSPPKAFVSKACSLSTGQPLNYSPNISTNIISSSNGIINPIRRSGNYEPTMWNYEYIQSTHNHHVGEKYMKRFNELKAEMKKHLMMMLHEESQELEKLELIDNLQRLGVSYHFKDEIIQILRSIHDQSSSEATSANSLYYTALKFRILRQHGFYISQDILNDFKDEQGHFKQSLCKDTKGLLQLYEASFLSTKSETSTLLESANTFAMSHLKNYLNGGDEENNWMVKLVRHALEVPLHCMMLRVETRWYIDIYENIPNANPLLIELAKLDFNFVQAMHQQELRNLSRWWKKSMLAEKLPFARDRIVEAFQWITGMIFESQENEFCRIMLTKVTAMATVIDDIYDVYGTLDELEIFTHAIQRMEIKAMDELPHYMKLCYLALFNTSSEIAYQVLKEQGINIMPYLTKSWADLSKSYLQEARWYYSGYTPSLDEYMENAWISVGSLVMVVNAFFLVTNPITKEVLEYLFSNKYPDIIRWPATIIRLTDDLATSSNEMKRGDVPKSIQCYMKENGASEEEARKHINLMIKETWKMINTAQHDNSLFCEKFMGCAVNIARTGQTIYQHGDGHGIQNYKIQNRISKLFFEPITISMP

>SollyTPS31

MAPAAALMSKCQEEEEIVRPVADFSPSLWGDRFHSFSLDNQVAEKYVEEIETLKEQTRSMLMSGKTLAEKLNLIDIVERLGIAYHFEKQIDDMLNHIFNIDPNFEAHEYNDLCTLSLQFRILRQHGYYISPKIFSRFQDANGKFKESLCDDIRGILNLYEASHVRTHGEDTLEEALAFSTAHLESAAPHLKSPLSKQVTHALEQSLHKSIPRVETRYFISIYEEEELKNDVFLRFAKLDFNLLQMLHKQELSEVSRWWKDLDFVTTLPYARDRAVECYFWTMGVYAEPQYSQARVMLAKTIAMISIVDDTFDAYGIVKELEVYTDAIQRWDVSQIDRLPEYMKISYKALLDLYNDYETELSNDGRSDVVQYAKERMKEIVRNYFVEAKWFIEGYMPPVSEYLSNALATSTYYLLTTTSYLGMKSATKKDFEWLAKNPKILEANVTLCRVIDDIATYEVEKGRGQIATGIECYMRDYGVSTQVAMDKFQEMAETAWKDVNEGILRPTPVSAKILTRILNLARIIDVTYKHNQDGYTHPEKVLKPHIIALLVDSIEI

>SollyTPS32

MALLNNQDEIVRPVANFSPSLWGDRFHSFSLDNQVADKYAQQIETLKEQTRSLLSDAACGTTLAEKLNLIDIVERLGLAYHFEKQIEDMLDQIYKADPNFEAHDLNTLSLQFRILRQHGYNISQKIFSRFQDANGKFKESLSNDIKGLLNLYEASHVRTHGEDILEEALAFSTAHLESAAPHLKSPLSKQVTHALEQSLHKSIPRVETRYFISIYEEEEFKNDVLLRFAKLDYNLLQMLHKQELSEVSRWWKDLDFVTTLPYARDRAVECYFWTMGVYAEPQYSQARVMLAKTIAMISIVDDTFDAYGIVKELEVYTDAIQRWDISQMDRLPEYMKVSFKALLDLYEDYEKELSKDGRSDVVQYAKERMKEIVRNYFVEAKWFIEGYMPPVSEYLSNALATSTYYLLTTTSYLGVKSATKEDFEWLAKNPKILEANVTLCRVVDDIATYEVEKGRGQIATGIECYMRDYGVSTQVAMDKFQEMAEIAWKDVNEGILRPTPVSTEILTRILNLARIIDVTYKHNQDGYTHPEKVLKPHIIALLVDSIEI

>SollyTPS33

MASAAALMSNCQDIVRPVADFSPSLWGDRFHYFSLDNQVAEEYAQEIETLKEQTRSLLSDAACGTTLAEKLNLIDIVERLGLAYHFETQIEDMLDQIYKSDPNFEAHDLNTLSLQFRILRQHGYNISPKIFCRFQDANGKFKESLSNDIKGLLNLYEASHVRTHGEDILEEALAFSTAHLESAAPHLKSPLSKQVTHALEQSLHKSIPRVETRYFISIYEEEEQKNDVLLRFAKLDFNLLQMLHKQELSEVSRWWKDLDFVTTLPYARDRAVECYFWTMGVYAEPQYSQARVMLAKTIAMISIVDDTFDAYGIVEELEVYTDAIQRWDISQIDRLPDYMKISYKALLDLYDDYETELSKDGRSDVVHYAKERMKEIVRNYFVEAKWFIEGYMPPVSEYLSNALATSTYYLLTTTSYLGVKSATKEDFEWLAKNPKILEANVTLCRVIDDIATYEVEKGRGQIATGIECYMRDYGVSTQVAMEKFQEMAEIAWKDVNEGILRPTPVSTEILTRILNLARIIDVTYKHNQDGYTHPEKVLKPHIIALLVDSVEI

>SollyTPS35

MASAAALVSNYREEEIVRPVADFSPSLWGDRFHSFSLDNKIAGKYAQEIETLKEQSRVILSASSGTTLAQKLDLIDIVERLGLAYHFEKQIDDVLDQIYKADPNSEAQEYNDLQTSSIQFRLLRQHGYNISPKLFSRFQDAKGKFNESLSNDIKGLLNLYEASHVRTHGEDILEEALAFSTAHLESAAPHLKSPLSKQVTHALEQSLHKSIPRVETRYFISIYEEEEQKNDLLLRFAKLDFNLLQMLHKQELSEVSRWWKDLDFVTTLPYARDRAVECYFWTMGVYAEPQYSQARVMLAKTIAMISIVDDTFDAYGIVKELEVYTDAIQRWDISHIDRLPDYMKISYKALLDLYDDYETELSKDGRSDVVHYAKERMKEIVRNYFVEAKWFIEGYMPPVSEYLCNALATSTYYLLTTTSYLGVKSANKEDFEWLAKNPKILEANVTLCRVIDDIATYDVEKGRGQIATGIECYMRDYGVSTEEAMEKFEEMAEIAWKDVNEGILRPTPVSTEILTRILNLARIIDVTYKHNQDGYTHPEKVLKPHIIALLVDSIEI

>SollyTPS36

MSGAMATFSVFPHSLINFNIWRYTCEPKVHSLKRKLMSPLLAMDVNSSRHLANFHSNIWGYHFLSYTSQLTEITTQEKLEVDELKEKVMNMLMEIRDDNSTQKLVLIDAIQRLGVAYHFHNEIETSIQNIFDASKQNDNDNNLYVVSLRFRLVRQQGHYISSDVFKQFMERDGKFKKTLNNDVQALLSLYEAAQIRVRGEDILEEALTFTTTHLESMIPLLSDNPLKAQIIEALTHPIHKVIPRLGARKYIDIYENMESHNHLLLKFSKLDFNMLQKQHQRELSELTSWWKDLDLASKVPYARDKLVEGYTWTLGVYFEPQYSRARRMLVKVFKMLSICDDTYDAYATFDELVLFTNAIQRWDINAMDSLPPYMRPFYQAILDIFDELEEELTKEGKSDRVYYGKFEMKKLARAYFKEAQWLNAGYIPNCDEYIKNAIVSTTFMALGTTSLIGMEEFITKDIFEWITNEPSILRASSTICRLMDDISDHESDQQRGHVASVIECYTKEYGASKQEAYVKFRKEVKDAWKGINKALLRPIEVPIFVLQRILNLARTMDTFFQDEEDGYTNSNSKCKDIVTLLLVDSVTIGRS

>SollyTPS37

MANITKAFSPLPLYLCQIGSKRSSIKVSCRSSNRWNFQEDLLKKTSYLQTSYNRDGFNSTKFGLLVKDVKYALRTQINNNNNLVLVDTLQRMGIEHHFQQEIQSILQKEYEQNTCFLKYQNHHDISLCFRLLRQEGYHVSADVFKKLKNNDDGTFGLNLNQDVNGLIGLYEASQLGVEGEYILDEIAKFSGDHLNACLANSDEARIIKETLKYPYHKSLSRWKNKSFINNFKGINGWGKNTLKELANMDYFITKEIHQHELAQVFRWWKSLGLAEELKLLRDQPLKWYTWPMAMLTDPKMSQERIELAKCISFVYVIDDIFDVYGTIEELTLFTQAVHRWELSAMMDLPEYMRSLYKALYNTINSIGYNIYKIYGQNPTQNLQNTWAHLCSAFLIEAKWFACGMVPTTDEYLKNGLVSSGVYVALIHLFYILGLGVSSMHLQDISLMSTSIAKILRLWDDLGSAKDENQEGKDGSYVEYYMKENKDSSMELAREHVIKLIEDEWKQLNKEHFCLMSQSTRSFSKASLNSARMVSLMYSYDDKQSLPILQEYIKSMLDGNL

>SollyTPS38

MLQSCISTMDIRRSGNYKPSIWEDGYVQSRPNLYAEEKYCERAEKLKEEVRKMLQKRMTNSLEQLELVDILQRLGIYYHFEEEIDTVLKQIYVNYNKRDHHNEELYDTALEFRLLRQHGYHLPQEIFCSFMNEEGKFKTALVEDTKGLLSLYEASYLCMEDENIMENARDFATHYLMENVKKKMDEQVSHALEMPVHWRMERLEARWFIEIYHKKENMNPLLLELAKLDYNMVQATYLEELKQMSRWDKNMKLVKKMSFVRDRLVEGFFWAVGFTPNPQFGYCRKLSTKLSVLLTTIDDIYDVYGTLDELELFTDIVDRWDINAIEQLPEYMKISFLALFNSMNELAYDILKEQGFSIISHIRKQWANLCKAYLLEVKWYQRGYTPSLDEFLRNAWITNTGPVLIMHAYFCITNPIKEDELQRLNHYPAIIYSPSLILRLANDLATSPDEIKKGDYLKSIQCYMHDSKSCEENARNYIKKLIDETWKKMNRDILRDESLSKDFRRTSMNLARIAQCMYQHGDGFGIPDRETKDRILSLFFQPIPLT

>SollyTPS39

MEMTKVLISPSQYLSMHIISGNIIQNERSIQVSCKSSNKWAVQEDLLRATSTYNQDGFDSTKFGLLMKDVKYALRTQINNNNNLVLVDTLQRMGIEHHFQQEIQSILQKEYEQNTCFLKYQNHHDISLCFRLLRQEGYHVSADVFKKLKNNDDGTFGLNLNQDVNGLIGLYEASQLGVEGEYILDEIAKFSGDHLNACLVNSDEARIIKETLKYPYHKSLSRWKAKSFINNFKGINGWGKSTLQELANMDYSITKEIHQHELIQVSRWWSSLGLAEDLKLLRDQPLKWYTWPMTMLTDPKMSQQRIELAKCISFVYVVDDIFDVYGTIEELTLFTQAVNRWELCVMKDLPEYMRATYKALYDTINSIGYNIYKIYGQNPTQNLRNAWANLCNAFLKEAKWFASGELPTTDEYLKNGLVSSGVHVVLVHMFYLLGFGLNNQNSIYLEDSSAMASSVATILRLWDDLGSAKDENQEGNDGSYIECYMKGQKNASIELAREYVVKLIEDEWKQLNKKHFNLMNGSLGSYSKASLNLARMVPLMYNYDDKQSLHVLQEYINTMLYDV

>SollyTPS4

MKAILLNNIGVLSSRPPRATCLFSINGGKPSSLIVVSKASSPNPTTIRRSGNYKPTMWDFQFIQSVNNLYAGDKYMERFDEVKKEMKKNLMMMVEGLIEELDVKLELIDNLERLGVSYHFKNEIMQILKSVHQQITCRDNSLYSTALKFRLLRQHGFHISQDIFNDFKDMNGNVKQSICNDTKGLLELYEASFLSTECETTLKNFTEAHLKNYVYINHSCGDQYNNIMMELVVHALELPRHWMMPRLETRWYISIYERMPNANPLLLELAKLDFNIVQATHQQDLKSLSRWWKNMCLAEKLSFSRNRLVENLFWAVGTNFEPQHSYFRRLITKIIVFVGIIDDIYDVYGKLDELELFTLAVQRWDTKAMEDLPYYMQVCYLALINTTNDVAYEVLRKHNINVLPYLTKSWTDLCKSYLQEARWYYNGYKPSLEEYMDNGWISIAVPMVLAHALFLVTDPITKEALESLTNYPDIIRCSATIFRLNDDLGTSSDELKRGDVPKSIQCYMNEKGVSEEEAREHIRFLIKETWKFMNTAHHKEKSLFCETFVEIAKNIATTAHCMYLKGDSHGIQNTDVKNSISNILFHPIII

>SollyTPS40

MSISASFLRFSLTAHYQPSPSSSPPNQPFKFLKSNREHVEFNRILQCHAVSRRRTKDYKEVQSGSLPVIKWDDIAEEVDVETHTLEVYDPSSNEDHIDAIRSMLGSMGDGEISVSAYDTAWVAMVKDVKGTETPQFPSSLEWIANNQLADGSWGDNSIFLVYDRVINTLACVIALKSWNLHPDKILLGMSFMRENLSRIGDENAEHMPIGFEVAFPSLIEIAKKLGLDFPYDSPVLQDIYASRQLKLTRIPKDIMHKVPTTLLHSLEGMTDLDWQKLLQFQCTDGSFLFSPSSTAYALMQTQDHNCLNYLKNAVHKFNGGVPNVYPVDLFEHIWTVDRLQRLGISRYFELEIKECIDYVSRYWTNKGICWARNSPVQDIDDTAMAFRLLRLHGYAVSADVFKHFESKGEFFCFVGQSNQAVTGMYNLYRASHVMFSGEKILENAKIFTSNYLREKRAQNQLLDKWIITKDLPGEVGYALDVPWYASLPRLETRFFLEHYGGEDDVWIGKTLYRMPLVNNSLYLELAKSDYNNCQALHQFEWRRIRKWYYECGLGEFGLSEKRLLVTYYLGSASIFEAQRSTERMAWVKTAALMDCVRSCFGSPQVSAAAFLCEFAHYSSTALNSRYNTEDRLVGVILGTLNHLSLSALLTHGRDIHHYLRHAWENWLLTVGEGEGEGEGGAELIIRTLNLCSVHWISEEILLSHPTYQKLLEITNRVSHRLRLYKGHSEKQVGMLTFSEEIEGDMQQLAELVLSHSDASELDANIKDTFLTVAKSFYYSAYCDDRTINFHIAKVLFERVV

>SollyTPS41

MRNLREEIKNMLSSMGDGRSSVSPYDTAWVSFIEDTNTNINGTSKRPLFPSCLQWIIDNQLDDGSWGEELVFCIYDRLLNTLACVVALTLWNTCLHKRNKGVMFIKENLRKLEGGEVVNMTSGFEFVFPSLLDKAQQLHIDNIPYDAPVFRDIYARREVKFTRFPKDLIHTIPTIVLFSLEGLRDLDWQRLLKLQMEDGSFLTSPSSTAIVFMNTNDDKCFTFLQNAVQKFNGGVPCSYPADIQARLWAIDRLQRLGISYYFEEEIKDLLEYVFRYWDKENGFFSARNSNICEVDTTCMAIRLLRLHGFDVSPDVLHKFKDGDEFFCLRGESNKSATVMFNLYRCSQALFPGEIICEEAKNFTYNFLHQYLANNQSKDKWVIAKDIPGEIRYALEFQWYASLPRVESRLYIDQYGGADEIWISKTLYRMPDVSNNVYLEAAKLDYNRCQSQHRFEWLIMQEWFEKGNFQKFGISKKEVLVSFFLAASSIFEVEKSRQRLAWAKSCILCKMITSYINQEATTWNSFLMEFKNYRDMSIKKSNETKEIIVLNNLCQFLHQLTKETYQDLGKDIHHQLHNVWEEWLEENNTTCQEAAVLLVQTINLSSGHMTHDEILSKYTNKVCHMLNEFQNDQICNSSKARDIELHMQALVKLVFSNTSSNNINQGIEDTYFKVVKTFYYTAHVSEETINNHISKVLFQKA

>SollyTPS5

MVSILSNIGMMVVTFKRPSLFTSLRRRSANNIIITKHSHPISTTRRSGNYKPTMWDFQFIQSLHNPYEGDKYMKRLNKLKKEVKKMMMTVEGSHDEELEKLELIDNLERLGVSYHFKDEIMQIMRSINININIAPPDSLYTTALKFRLLRQHGFHISQDILNDFKDENGNLKQSICKDTKDILNSSKDEHDNLKQSTCNNTKGLLKLYEASFLSIENESFLRNTTKSTLAHLMRYVDQNRCGEEDNMIVELVVHALELPRHWMVPRLETRWYISIYERMSNANPLLLELAKLDFNIVQATHQQDLRILSRWWKNTGLAEKLPFSRDILVENMFWAVGALFEPQHSYFRRLITKVIVFISIIDDIYDVYGTLDELELFTLAIQRWDTKAMEQLPDYMKVCYLALINIINEVAYEVLKNHDINVLPYLTKSWADLCKSYLQEAKWYHNGYKPNLEEYMDNARISIGVPMVLVHSLFLVTNQITKEALDSLTNYPDIIRWSATIFRLNDDLGTSSDELKRGDVSKSIQCYMNEKGASEEEAIEHIEFLIQETWEAMNTAQSKNSPLSETFIEVAKNITKASHFMYLHSDVKSSISKILFEPIIISNVAFALK

>SollyTPS7

MVSIFSNAGMMMVTFNRPSFTCFSSLHHYSISARGAINNISTPISATRRSGNYKPTMWDFQFIQSLHNPYEGDKYMKRLNELKKEVKKMMMTVEGSHDEELEKLELIDNLERLGVSYHFKDEIMQILRSINININIAPPDSLYTTSLKFRLLRQHGFHISQDVLKDFKDENGNLKQSICKDTKGMLELYEASFLSTETENTLKSATRFTMSHLKNYVDNHSCGNQDDDIIVELVVHALELPRHWMMPKLETEWYIRIYGRMPNANPLLLELAKLDFNIVQAAHQQDLKILSRWWKSMSLAEKLSFSRDRLVEDFFWSVGLAFEPQHSLCRRMLAKNVAFIIVIDDIYDVYGSLDELEIFTHAVERWDIKAMEQLPDYMKICYLSLFNTTNEMAYHILKQQGINVLPYLTKQWTDLCKSYLQEAKWYHNGHKPRLEEYMDNAWISIATPLVLLHAFIFLTNPITQEALESLNNYPDIIRRCAIINRFVDDLGTSSDELKRGDVPKSIQCYMNDTGASEEEAREHINLLIKEMWEVMNKDQISKQVLFSEEFIKIVFNFSRTSHCVYQHGDGHGIQNSHITNRISKLLFEPLII

>SollyTPS8

MYKLEMTMSISKSNLISKLEVPKSCISNVPIRRSGNYQPSIWDYNHIQSLKNHYSDEKFMRRRNELKMEVKIMLSDRNMKQLEQLEIIDNLQRLGLSYHFEDEIYSILNNLSDKGSKRDHLYAKALEFRLLRQHGFNIVSQETFGGFYDNTTGFGEIHHNEDTKGMLYLYEASFLAIEGEKELELARNLTEEHLREYLADQNKNDVDQNLVELVHHALELPLHWRMLRLETKWFINYYKKRQDKMIPFLLELATLDFNIVQAAHIEDLKYVARWWKETCLAENLPFARDRLVENFFWTIGVNFLPQYGYFRRIATKVNALVTTIDDVYDVFGTLDELQIFTHAIERWSIDELDRLPDNMKMCYYALDNFINQLADDAFEEQGIFISPYLRNSWRDLCKSYLREAKWYHSQYIPSMEEYMDNAWISISAPVILVHAYFLVANPVNKEALHYLENNYHDIIRCSALILRLANDLGTSSDELKRGDVPKSIQCYMNETQASEEEARQYIRLLISQTWKKLNEAHWLAADPFPKIFVTCAMNLARMAQCMYQHGDGHGGNNSTTKNHIMALLFESVPLGHKHSSAEKEDHSMVNYREKFMI

>SollyTPS9

MAASSADKCRPLANFHPSVWGYHFLSYTHEITNQEKVEVDEYKETIRKMLVETCDNSTQKLVLIDAMQRLGVAYHFDNEIETSIQNIFDASSKQNDNDNNLYVVSLRFRLVRQQGHYMSSDVFKQFTNQDGKFKETLTNDVQGLLSLYEASHLRVRNEEILEEALTFTTTHLESIVSNLSNNNNSLKVEVGEALTQPIRMTLPRMGARKYISIYENNDAHHHLLLKFAKLDFNMLQKFHQRELSDLTRWWKDLDFANKYPYARDRLVECYFWILGVYFEPKYSRARKMMTKVLNLTSIIDDTFDAYATFDELVTFNDAIQRWDANAIDSIQPYMRPAYQALLDIYSEMEQVLSKEGKLDRVYYAKNEMKKLVRAYFKETQWLNDCDHIPKYEEQVENAIVSAGYMMISTTCLVGIEEFISHETFEWLMNESVIVRASALIARAMNDIVGHEDEQERGHVASLIECYMKDYGASKQETYIKFLKEVTNAWKDINKQFFRPTEVPMFVLERVLNLTRVADTLYKEKDTYTNAKGKLKNMINSILIESVKI

>Vv01000402001

MAYHTLKEQGHNILPYLTKAWADLCKVFLVEAKWAHKEYIPTFEEYLENGWRSASGVAILIHAYFLMSKNITKEALECLENDHELLRWPSTILRLCNDLATSKAELERGESANSISCYMHQTGVSEESAREHMKILTGESWKKMNKVPDPFSKPFMEIFNLARISECTY

>Vv01000410001

MECFLWTVGIFPDPRHSSCRIELTKAIAILLVIDDIYDSYGSLDELALFTDAVKRWDLGAMDQLPEYMKICYMALYNTTNDIAYRILKEHRWSVIEDLKRTWMDIFGAFLAEAHCFKGGHVPSLEEYLNNAVTTGGTYMALVHAFFLMGQGVTRENMAMLKPYPNIFSCSGKILRLWDDLGTAREEQERGDNASSIECYKRERRLDDEACRKHIRQMIQSLWVELNGELVALPLSIIKAAFNLSRTAQVIY

>Vv01000414001

MECFLWTVGIFPDPRHSSCRIELTKAIAILLVIDDIYDSYGSLDELALFTDAVKRWDLGAMDQLPEYMKICYMALYNTTNDIAYRILKEHGWSVIEDLKRTWMDIFGAFLAEAYCFKGGHVPSLEEYLTNAVTTGGTYMALVHAFFLMGQGVTRENMAMLKPYPNIFSCSGKILRLWDDLGTAREEQERGDNASSIECYKRERMLEDEACRKHIRQMIQSLWVELNGELVALPLSIIKAAFNLSRTAQVIY

>Vv01001153001

TRNTRVSDVDDTSMGFRLLRLHGYDVSPDVFKQFEKGDEFVCFPGQSSQAITGLFNLFRASQFLLPGEKILENARKFCSKFLREKQTCNLEDKWIIAKDLAGEVGYALDIPWYASLPRVETRFYVEQYGGGDDIWIGKTLYRMYHVNNDIYSELAKLDFNNCQELHQLEWDRIQEWWTHSNLQEFGLSRETLLLAYFLAAASIFEPERSVERVAWAKAAVLVEAVASYFNKETCIKQRRAFLLKFGRYRKGSSNLSKTGEELAGLLLTTLNQLSLDAQELHGSDIHQLLHRTWEMWLTKNLAEEDGCQGEAEVLVDVINLCSGRSITEELLNHPLYKHLLHLTNGVSHQLSPFYQHKAHIGSYNPEKESDVSSKVEPDMQELAQLVLDIDPVIKRTFLMVAKSFYY

>Vv1007468001

TVSDCHNNLYELALGFRLLRQEGYYVSADVFNNLKDTEGKLQEKLSQDIKGLMGLYEASQLCIKGEDTLEEIGNFSSQLLNAWNTHNDHSQARIVRNTLGHPHHKSLAKFMAKSFLSDFQGTDGWVNVFRELAKMDFNVVKSIHQKEMLQVSK

>Vv01001155001

AKNSEVHDIDDTAMGFRLLRLHGHDVSADVFKYFEKGGEFFCFAGQSSQAVSGMFNLYRASQVLFPGETILENAKKFSSKFLREKQACDLLDKWIIMKDLPGEIGYALDVPWYASLPRVETRIYIEQYGGKDDVWIGKTLYRMPYVNNNDYLELAKLDFNNCQALHQLEWDSIQQWYTQCHLGEFGVSRKASLLAYFLAAACIFEPERSIERLAWAKTAILVLAVRSYFEKFGYSPSGGDYMKKINGRGSNMTAVFLKSGEELVGLLLGTLNQLSLDTLVAHGRDIRHILRQTWEMWLMKHLDEGDGYRGEAELLVRTINLCAGRSLSEELLAHPQYRRLSQLTNRICNDLGLFLHKGHNGTYNPENGSPMSHRVESDMQELTKIVLGINPEIKRTFEMVGKSFYAAY

>Vv01005217001

TVSDCHNNLYELALGFRLLRQEGYYVSADVFNNLKDTEGKLQEKLSEDIKGLMGLYEASQLCIKGEDILEEIGNFSSQLLNAWNTHNDHSQARIVRNTLGHPHHKSLARFMAKSFLSDFQGTDGWVNVFRELAKMDFNVVKSIHQKEMLQVSKWWKDLGLAKELKFARNQPLKWYMWPLAVLPDPSLSEQRVELTKPISLVYIIDDIFDVHGTLDELTLFTEAVNRWEYAAVEQLPDYMKICFNALNGITNEISSKVYNDHGWNPMESLRKAVCQITFTILD

>Vv01005218001

IHGDCDEDLYEVALRFRLLRQEGYTVPADVLNNFKNKEGKFKQNLREDIRGLMGLYSFSAKYRRRYT

>Vv01005221001

IHGDCDEDLYEVALRFRLLRQEGYTVPADVLNNFKNKEGKFKQNLREDIRGLMGLYEASQLSIGEDILEEAGNFSSLLLNACLQHLDRHQAAVVKNTLEHPHHKSWARFMTKNFLTDFQGTNGWINALQELAKIDFNMVKSVHQKEMLQISKWWKDLGLTEELKFARDQPLKWYMWPMAIIPDPRLSEQRIELTKPISLIYIIDDIFDVGGTLDELTLFTEAVNRWDLSAFKELPEYMKMCFKTLDDITNEISTKVHKEHKWNPVGSLRKAWASLCNAFLVEAKWFASGQRLRST

>Vv01005268001

IHGDCDEDLYEVALRFRLLRQEGYTLPADVLNNFKNKEGKFKQNLREDIRGLMGLYEASQLSIGEDILEEAGNFSSLLLNACLQHLDRHQAAVVKNTLEHPHHKSLARFMTKNFLTDFQGTNGWINALQELANIDFNMVKSVHQKEMLQISKWWKDLGLTEELKFARDQPLKWYMWPMAIIPDPRLSEQRIELTKPISLIYIIDDIFDVGGTLDELTLFTEAVNRWDLSAFKELPEYMKMCFKTLDDITNEISTKVHKVHKNPVGSLRKAWASLCNAFLVEAKWFASGHVPKAEEYLKNGAVSSGVHVVLVHLFFLLGHGITKENVDLVDDFPGIISSTATILRLWDDLGSAKDENQDGHDGSYVECYLKEHGSSVENARQIVAHMISDMWKRLNKECLPFSTSFTKGSLNIARMVPLMY

>Vv01005271001

THGESIQDLYEVALRFRLLRQEGYHVPADVFNNFKNKEGKFKQNLSKDIKGLLGLYAASQLSIEGEDILEEAQRFSSTLLNAGLEHLDHHEATVVGHTLEHPHHKSLPRFMAKSFLKDFQGPNGWLTVLQELAKADFNMVQSIHQQELLQISKWDIAAFETLPNYMKICFKTLDDITNEISNKVYKEHGWDPVDSLRKTWMGLCNAFLVEAKWFASGHVPKAHEYLKNGVISSGVHVVLVHLFFLLGHGITRGNVDLVDDFPSTISSTAAILRLWDDLGSAKDENQDGHDGSYIGCYIKEHGSSMENARQNVTYMISDLWKRLNKECLPFSTSFTEGSLNIARMVPLMY

>Vv01005272001

THGESIQDLYEVALRFRLLRQEGYHVPADVFNNFKNKEGKFKQNLSKDIKGLLGLYEASQLSIEGEDILEEAQRFSSTLLNAGLEHLDHHEAVVGHTLEHPHHKSLPRFMAKSFLKDFQGPNGWLTVLQELAKADFNMVQSIHQQELLQISKWWQDLGLAEELKFARDQPLKWHMWPMAVLPDPSLWDIAAFETLPNYMKICFKTLDEITNEISNKVYKEHGWNPVDSLRKTWMSLCNAFLVEAKWFASGHVPKAHEYLKNGVISSGVHVVLVHLFFLLGHGITRGNVDLVDDFPSIISSTAAILRLWDDLGSAKDENQDGHDGSYIECYIKEHGSSMENARQNVTYMISDLRKRLNKECLPFSTSFTKGSLNIARMVPLMY

>Vv01006399001

NSPGIKANLYAAALCFRLLRQHGYGVSQDMFSGFMEEVGMFSKSTCTNVKGMIELFEASHLALEGENILDEAKAFSSGYLKEIISNLDNNLAKQVVHSLERPLHWRVQWFDIRWYIDFYEEEGVNLDLLKLAKLNFNMVQAVHQKDLKHISRWWRNLGLIENLSFTRDRLVESFLCGVGFSSEPQHGSFRLCITKVIIFIQVLDDVYDIYGSLDELEQFTNAVDRWDSKEIQQLPESMKLCFQVLQDTTNAVANEIQKEKGWDNVLPMLQQAWANFCKSLFVEAKWYNKGYTPTLQEYLSNAWISSSVSLLSVHAIFFCVPEAKEEMVDFLEKNQELVYSSSLILRLCNDLGTLEAELERGDAASSVLCYMKEVNVSEEISRTHIRGMLVKTWKNMNGHCIPLLQPIVNIITNIARVAQWIY

>Vv01006465001

IHGDCDEDLYEVALRFRLFRQEGYTVPADVLNNFKNKEGKF

>Vv01006466001

TVSDCHNNLYELALGFRLLRQEGYYVSADVFNNLKDTEGKIQEKLSEDIKGLMGLYEASQLCIKGEDILEEIGNFSSQLLNAWNTHNDHSQARIVRNTLGHPHHKSLAKFMAKSFLSDFQGTDGWVNVFRELAKMDFNVDENQDGHDGSYLECYMKEKGTSIENARCHVMHTISETWKSLNNECLPFSTSFTKACLNVARMVPLMY

>Vv01006467001

TVSDCHNNLYELALGFRLLRQEGYHVSADVFNNLKDTEGKLQEKLSEDIKGLMGLYEASQLCIKGEDILEEIGNFSSQLLNAWDTHNDHSQARIVRNTLGHPHHKSLARFMAKSFLSDFQGTDGWKNVFRELAKMDFNVVKSIHQKEMLQVSKWWKDLGLTEELKFARDQPLKCYMWPMAIIPDPRLSEQRIELTKPISLIYIIDDIFDVGGTLDELTLFTEAVNRWDLSAFKELPEYMKMCFKTLDDITNEISTKVHKEHKWNPVGSLRKAWASLCNAFLVEAKWFASGHVPKDEEYLKNGAVSSGVHVVLVHLFFLLGHGITKDNVDLVDDFPGIISSTATILRLWDDLGSAKVNNHMQVVN

>vv01006589001

IHGDCDEDLYEVALRFRLLRQEGYTVPADVLNNFKNKEGKFKQNLREDIRGLMGLYEASQLSIGEDILEEAGNFSSLLLNACLQHLDHHQAAVVKNTLEHPHHKSLARFMTKNFLTDFQGTNGWINALQELAKIDFNMVKSVHQKEMLQISKWWKDLGLTEELKFARDQPLKWYMWPMAIIPDPRLSEQRIELTKPISLIYIIDDIFDVGGTLDELTLFTEAVNRWDLSAFKELPEYMKMCFKTLDDITNEISTKVHKEHKWNPVGSLRKAWASLCNAFLVEAKWFASGHVPKAEEYLKNGAVSSGVHVVLVHLFFLLGHGITKENVDLVDDFPGIISSTATILRLWDDLGSAKVNNHMQVVN

>Vv01006591001

IHGDCDEDLYEVALRFRLLRQEGYTVPADVLNNFKNKEGKFKQNLREDIRGLMGLYEASQLSIGEDILEEAGNFSSLLLNACLQHLDRHQAAVVKNTLEHPHHKSLARFMTKNFLTDFQGTNGWINAFQELAKIDFNTVKSVHQKEMLQISKWWKDLGLTEELKFARDQPLKWYMWPMAIIPDPRLSEQRIELTKPISLIYIIDDIFDVGGTLDELTLFTEAVNRWDLSAFKELPEYMKMCFKTLDDITNEISTKVHKEHKWNPVGSLRKAWASLCNAFLVEAKWFASGHVPKAEEYLKNGAVSSGVHVVLVHLFFLLGHGITKENVDLVDDFPGIISSTATILRLWDDLGSGKDENQDGHDGSYVECYLKEHGSSVENAR

>Vv01006642001

NSPGIKANLYAAALCFRLLRQHGYGVSQDMFSGFMEEVGMFSKSTCTNVKGMIELFEASHLALEGENILDEAKAFSSGYLKEIISNLDNNLAKQVVHSLERPLHWRVQCFDIRWYIDFYEEEGVMNLDLLKLAKLNFNMVQAVHQKDLKHISSNAWISSSVSLLSVHAIFFCVPEAKEEMVDFLEKNQELVYSSSLILRLCNDLGTLEN

>Vv01013515001

MAYDLLKEQGSHIIAYLRKAWADLCKSYLLEAKWYHARYTPTLQEYLSNAWISISAPTILVHAFFFVTNPITEDALECVEQYCNIIRWSSIILRLSDDLGTSSDELKRGDVPKSIQCYMHETGASEEDAREHIKCLIGETWKKMNEDRVPFSQTFIGIAINLARMAQCMY

>Vv01013517001

MLRLEARWFIDAYERSQDMNPILLEFAKLDYNMVQAKHQEDLKYASRWWRSTRLGEKLSFARDRLMENFLWTVGEIFEPQFGYCRRMLTKVNAMITTIDDVYDVYGTLEELELFTDAVDRWDINAMDQLPEYMKICFLALYNSTNEMAYDLLKEQGSHIIAYLRKAWADLCKSYLLEAKWYHARYTPTLKEYLSNAWISISAPVILVHAFFFVTNPITEDALECLEQYCNIIRWSSIILRLSDDLGTSSDELKRGDVPKSIQCYMHETGACEEDAREHIKCLIGETWKKMNEDRVPFSQTFIGIAINLARMSQCMY

>Vv01013518001

MNPILLELAKLDYNMVQATYQEDLKHASMWWRSTRLPEKSSFSRDRLVENFLWAVGFIFEPQFGYCRRMLTKLISLITTIDDVYDVYGTLDELELFTDAVDRLVVSVYSLSF

>Vv01014174001

LVDDNEDLTNASLRFRLLRQEGYGVPSDVFSKFKDKEGNFKESLIGDLPGMLALYEATHLMVHGEDILEEALAFTTAHLQSVATDPNNPLSKQVIRALKLSIHNGVTSVGARHYISIYQEDGSHNESLLKLAKLDFNLLQSLHRKELSEITRWWKGLDFATKLPFARDRLVEIYFSALGVCFEPQYSLSLRFLTKVAIMITMVDDIYDAYGTIEELTLLTEAIERWDASSIDQLPDYMKCFYRALLDLYEEMEQEMAKEGKLYRVHYAKELMKKQIQSYFVEAKWSNQGYIPTFDEYMSNGVVSGCCSLLIATSFVGMGDIVTKESFQWVLSRPTMIGASQIICRLMDDMASHEFEQKRVHVASSVECYMKQYGVSKQEAYDELNKQVVKAWKDINQECLPVLMPIITRVLNIARMMNILY

>Vv01014175001

LVDDNDDFTNASLRFRLLRQEGYPIPSDVFNKFKDKEGNFKEYLIGDSLGMLALYEATHLMVHGEDILEEALAFTTTHLQSMATDPNNPLAKQVIRALKRPIRKGLTRVEATHYISIYQQDGSHNKSLLKLAKLDFNLLQSLHRKELSEISRWWKGLDVATKLPFARDRLVESYFWTLGVYFEPQYFPARRFLTKMTAMLTIMDDIYDAYGTIEELELLTEAVERWDASCIDQLPDYMKWFYRALLDVYEEMEEEMAKEGKLYRVHYAKEAMKRQIQAYFIEAKWLNQRYVPTFDEYISNTLVSCGYTLLIATSFVGMGDVVTEEAFQWVLSHAKMIRASETISRLMDDLVSHEFEQTRMHAPSSIECYMKQYEVTKQEACDELNKQIVRAWKDINQECLQVPMPLVTRVLNFSRVIDILY

>Vv01014323001

LVDDDNDDLASAALRFRLLRQEGYHIPSDVFKKFKDEEGNFKESLVGDLPGMLALYEATHLMVHGEDILDEAQAFTTAHLQSMAPDSDNPLKKQVIHALERPIRKSLIRVEARHYISIYQEYDSHNKSLLKLAKLDFNLLQSLHRKELSEITKWWKGLDFATKLPFARDRIVEGYFWVLGVYFEPQYFLARRILLKVFAMTSIIDDIYDTHGTFEELKLFAEAIERWDASSIDQLPDYMKLCYQALLDVFEEIEEEMTKQEKPYRVHYAKQAMKKLVQAYFVEAKWLKQEYIPTIEEYMTNALVSCGYFQLATISFVGMGDMVTKKAFDWVFSDPKMVRASAIICRLMDDIVDHEFEQKRGYVVSGVQCYMKQYGVTEQEACEEFINEIMNAWKDMNEECLQVPMPLLTCVVNFARVIDLLY

>Vv01014324001

LVDDDNDDLASAALRIRLLRQEGYHIPSDVFKKFKDEEGNFKESLVGDLPGMLALYEATHLMRPIRKSLIRVEARHYISIYQEYDSHNKSLLKLAKLDFNLLQSLHRKELSEITKWWKGLDFATKLPFARDRIVEGYFWVLGVYFEPQYFLARRILLKVFAMTSIIDDIYDTHGTFEELKLFAEAIERWDAGSIDQLPDYMKLCYQALLDVFEEIEEEMTKQEKPYRVHYAKQAMKKQVQAYFVEAKWLKQEYIPTIEEYMTNALVSCGYFQLATISFVGMGDMVTKKAFDWVFSDPKMVRASAIICRLMDDIVDHEFEQKRGYVVSRVQCYMKQYGVTEQEACEEFNNEIMNAWKDMNEECLQVPMPLLTCVVNFARVIDLLY

>Vv01014325001

LVDDNDHLTTVSLLFRLLRQEGYHIPSDVFKKFMDEGGNFKESLVGDLPGMLALYEAAHLMVHGEDILDEALGFTTAHLQSMAIDSDNPLTKQVIRALKRPIRKGLPRVEARHYITIYQEDDSHNESLLKLAKLDYNMLQSLHRKELSEITKWWKGLDFATKLPFARDRIVEGYFWILGVYFEPQYYLARRILMKVFGVLSIVDDIYDAYGTFEELKLFTEAIERWDASSIDQLPDYMKVCYQALLDVYEEMEEEMTKQGKLYRVHYAQAALKRQVQAYLLEAKWLKQEYIPRMDEYMSNALVSSACSMLTTTSFVGMGDIVTKEAFDWVFSDPKMIRASNVICRLMDDIVSHEFEQKRGHVASAVECYMKQYGVSKEEAYDEFKKQVESAWKDNNEEFLAVPVPLLTRVLNFSRMMDVLY

>Vv01014557001

LDDDDNDDLTNASLRFRLLRQEGYHIPPDVFNKFKDDEGNFKESLTGDLPGLLALYEATHLMVHGEDILEEALAFSTAHLQSMATDSTHPLAAQVTRALKRPIRKCLTRVEARHYISVYQEDGPHNKTLLKLSKLDFNLLQSLHRKELSEVTRWWKGLNFAKKMPFARDRLVEGYFWILGVYFEPQYSLARRILIKVLAMISIIDDIYDAYGTFEELKLFTEAIERWDANSVNQLPDYMKPCYQALLDVYEEMEEEMAKEGNLYRVQYAKAAIERQIQAYFVEAKWLNQEYTPTLDEYMSNALISSGYSMLMTTSFVGMGDIATKEAFDWVFNDPKMVRASSVICRLMDDIVSHEFEQKRGHVASAVECYMKQHGVCKQEAYDELNKQVVNAWKDMNKECLQVPMPLLTRVLNFSRVIDILY

>Vv01014558001

LDDDDNDDLTNASLRFRLLRQEGYHIPPDVFNKFKDDEGNFKESLTGDLPGLLALYEATHLMAHGEAILEEALAFSTAHLQSMATDSTHPLPAQVTRALKRPIRKCLTRVEARHYISVYQEDGPHNKTLLKLSKLDFNLLQSLHRKELSEVTRWWKGLNFAKKMPFARDRLVEGYFWILGVYFEPQYSLARRILIKVLAMISIIDDIYDAYGTFEELKLFTEAIERWDASSVDQLPDYMKPCYQALLDVYEEMEEEIAKEGNLYRVQYAKAAIQRQIQAYFVEAKWLNQEYTPTLDEYMSNALVSSGYSMLITTSFVGMGDIATKEAFDWVFSDPKMVRASSVICRLMDDIVSHEFEQKRGHVASAVECYMKQRGVCKQEAYDELNKQVVNGWKDMNEECLQVPMPLLTRVLNFSRVIDILY

>Vv01014566001

EDVYTASLRFRLLRQQGYHVSCDLFNNFKDNEGNFKESLSSDVRGMLSLYEATHLRVHGEDILDEALAFTTTHLQSAAKYSLNPLAEQVVHALKQPIRKGLPRLEARHYFSIYQADDSHHKALLKLAKLDFNLLQKLHQKELSDISAWWKDLDFAHKLPFARDRVVECYFWILGVYFEPQFFLARRILTKVITMTSTIDDIYDVYGTLEELELFTEAVERWDISVIDQLPEYMRVCYRALLDVYSEIEEEMAKEGRSYRFYYAKEAMKKQVRAYYEEAQWLQAQQIPTMEEYMPVASATSGYPMLATTSFIAMGDVVTKETFDWVFSEPKIVRASATVSRLMDDMVSHKFEQKRGHVASAVECYMKQHGASEQETRDEFKKQVRDAWKDINQECLAVPMTVLMRILNLARVMDCIY

>Vv01014569001

VSAEEDVYTASLRFRLLRQQGYHVSCDLFNNFKDNEGNFKESLSSDVRGMLSLYEATHFRVHGEDILDEALAFTTTHLQSATKHSSNPLAEQVVHALKQPIRKGLPRLEARHYFSVYQADDSHNKALLKLAKLDFNLLQKLHQKELSDISAWWKDLDFAHKLPFARDRVVECYFWILGVYFEPQFFFARRILTKVIAMTSIIDDIYDVYGTLEELELFTEAVERWDISAIDQLPEYMRVCYQALLYVYSEIEEEMAKEGRSYRLYYAKEAMKNQVRAYYEEAKWLQVQQIPTMEEYMPVALVTSAYSMLATTSFVGMGDAVTKESFDWIFSKPKIVRASAIVCRLMDDMVSHKFEQKRGHVASAVECYMKQHGASEQETHNEFHKQVRDAWKDINEECLAVPMPILMRVLNLARVIDVIY

>Vv01030646001

TTDDLSTTALRLRLLRQHGYPVSSEVFDQFRSKDGRFMDGISQDIAGLLSLYEASHLGLEGEDDLEEARRFSTRHLKSLVGNLESDLADQVQQSLEVPLHWRMPRLEARNFIDIYQRRNTKNSALLELAKLDYNLVQSSYQKELKELTRWWTDLGFKEKLSFSRDRLVENYLWSMGIAPEPHFSKCRIGLTKFICILTAIDDMYDIYGSPDELRRFTDAVNRWDTGALVDLPDYMKICYLAMYNFANEMAYDALRDHDLYILPYLKSQAEIARGDVAKSIQCYMIEERISEEQARDQVEKLIGYSWKKLNEASISLPKSMINSSLNMARSAQCIF

>Vv01030647001

TTYDLFTTALRFRLLRQHGYSISSDVFDKFRSKDGRFMDGISQDIAGLLSLYEASHLGVEGEDDLEEARRFSTMHLKGLVGNLEGDLADQVQQSLEVPLHWRMPRLEARNFIDIYQRRNTKNSALLELAKLDYNLVQSVYQKELKELTRWWTDLGFKENLSFSRDRLMENYLWSMGFTPEPHFSKCRIGLTKFICIFSAVDDMYDIYGSPDELRRFTDAVNRYEIYNIIIIQAANLLYVLFCVTLG

>Vv01033458001

TTDDLSTTALRFRLLRQHGYPVSSEVFDQFRSKDGRFMDGISQDIAGPLSLYEASHLGVEGEDDLEEARRFSTIHLKSLVGNLESNLADQVQQSLEVPLHWRMPRLEARNFIDIYQRRNTKNSALLELAKLDYNLVQSSYQTELKELTR

>Vv01036308001

DADDTEDDLYNIALQFRLLRQQGYNISCGIFNKFKDEKGSFKEDLISNVQGMLGLYEAAHLRVHGEDTLEEALAFTTTHLKATVESLGYPLAEQVAHALKHPIRKGLERLEARWYISLYQDEASHDKTLLKLAKLDFNLVQSLHKEELSNLARWWKELDFATKLPFARDRFVEGYFWTLGVYFEPQYSCARRILTKLFAMASIIDDIYDAYGTLEELQPFTEAIERWDIKSIDHLPEYMKLFYVTLLDLYKEIDQELEKYGNQYRVYYAKELKSQVRAYFAEAKWSHEGYIPTIEEYMLVALVTSGSCILATWSFIGMGEIMTKEAFDWVISDPKIITASTVIFRLMDDITTHKFEQKRGHVASGIECYMKQYGVSEEQVYSEFHKQVENAWLGINQECLAVPMPLLTRVVNLSRVMDVIY

>Vv01036312001

DADDTNDDLYNIALRFRLLRQQGYNISCGIFNKFKDEKGSFKEDLISNIQGMLGLYEAAHLRIHGENILEEALAFSTTHLKAMVESLGYPLAEQVAHALKRPIRKGLERLEARWYISIYQDEAFHDKTLLKLAKLDFNLVQSLHKEELSNLARWWKKLDFATKLPFARDRLVEGYFWIVGVYFEPQYLWAIRILTKIIVMTTVIDDIYDAYGTLEEIKHFTEAIERWDINSIDHLPEYMKLFFVALLDVYKEIEEEMEKERYQYRVHYAIEAMKNQVRAYFAEAKWFHEQHIPTMEEYMSVALSCSGYSLLATSSFIGMGEIASKEAFDWVIRDPKIIRASTVIARFMDDMKSHTREFHNQIVNVWMDINQECLAVPMPLLTRVLNLSRVMDVIY

>Vv01036313001

MASIIDDIYDAYGTLEELQPFTEAIERWDINSIDHLPEYMKLFYVTLLDLYKEIDQELEKDGNQYRVYYAKEVLKSQVRAYFAEAKWSHEGYIPTIEEYMLVALVTAGSCILATWSFIGMGEIMTKEAFDWVISDPKIITASTVIFRLMDDITTHKFEQKRGHVASGIECYMKQYGVSEEQVYSEFHKQVENAWLDINQECLAVPMPLLTRVVNLSRVMDVIY

>Vv01036315001

CIDDINDDLYDVALRFRLLRQQGFNISCDIFNRYTDEKGRFKESLINDACGLLGLYEAAHLRVGEEDILDEALAFTTTHLKSMVEHLEYPLAAQVTHALYRPLRKGLERLEARPFMSIYEDEASHSKALLKLAKLDFNQLQSLYKKELSNILGWWKDLDFSSKLPFVRDRLVEGYFWIATACFEPQYSYARRIQTKLHALITTTDDIFDAYGTLEELELFTEAIGRWDIDSTHQLPEYMKPCYQAVFDAYKEIEEMENTERSHSVHKAKDAMKNLVQAYLVEAKWFHGKYIPTIEEYMRVALVSIGAPVLTFISFIGMGEIATKEVFDWLQQNPKIVRASSKVIRLMDDMATHKFEQERGHIASSIECYMKQHGVSEQQAYGEFHKQLENAWKDINEECLAVPMLLLSRLLNFARAADVMY

>Vv01036317001

DADDTDDNLYNIALQFRLLRQQGYNISCGIFNKFKDEKGSFKEDLISNIQGMLGLYEAAHLRVHGEDILEEALAFTTTHLKATVESLGYPLAEQVAHALKHPIRKGLERLEARWYISLYQDEASHDKTLLKLAKLDFNLVQSLHKEELSNLARWWKELDFATKLPFARDRFVEGYFWILGVYFEPQYSRARRILTKLFAMASIIDDIYDAYGTLEELQPFTEAIERWDISSIDHLPEYMKLFYVTLLDLYKEIDQELEKDGNQYRVYYAKEVLKSQVRSYFAEAKWSHEGYIPTIEEYMLVALVTAGSCILATWSFIGMGEIMTKEAFDWVISDPKIITASTVIFRLMDDITTHKFEQKRGQVASGIECYMKQYGVSEEQVYSEFHKQVENAWLDINQECLAVPMPLLTRVVNLSRVMDVIY

>Vv01036320001

KQLKQQGFNISCDIFNRYTDEKGRFKESLINDACGLLGLYEAAHLRVWEEDILDEALAFTTTHLKSMVELLEYPLAAQVTHALYRPIRKGLERLEARPYMSIYQDEASHSKALLKLAKLDFNLLQSLYKKQLSNISRWWKDLDFSSKLPFGRDRVVECYFWIATACFEPQYSYARRIQTKILALITIIDDMFDAYGTLEELELFTEAIGRWDVNSIYQSYCVHYAKEAMKRSVRAYFNEAKWLHEDYVPTIEEYLSVAQVTSEVTLFTVICFVGMGMATKEVFEWVWNDPKIVGASSKIMRLMDDMASHKFEQERGHSASSVECYMKQHGVSEQHAYQELNKQVENAWKDVNQGCLAIPMPLLTRVLNFARTGDFMY

>Vv01036322001

NGDDMEGDIYNVALQFRLLRQAGFNISCGLNEFKDEKGNFKKALVSDVRGMLGLYEAAHLRVHGEDILAKALAFTTTHLKAMVESLGYHLAEQVAHALNRPIRKGLERLEARWYISVYQDEAFHDKTLLELAKLDFNLVQSLHKEELSNLARWWKELDFATKLPFARDRLVECYFWMLGVYFEPQYLRARRILTKVIAMISILDDIHDAYGTPEELKLFIEAIERWDINSIDQLPEYMKLCYAALLDVYKEIEEEMEKEGNQYRVHYAKEVMKNQVRAYFAEAKWLHEEHVPTFEEYMRVALVSSGYCILATTSFVGMGIATKEAFDWVTSNPKIMSSSNFIARLMDDIKSHKFEQKRGHVASAVECYMKQYGVSEEQVYKEFQKQIENAWLDINQECLAVSMPLLARILNLTRAADVVY

>Vv01036330001

DCNDMDGDLYNIALGFRLLRQQGYSISCGIFNKFMDERGRFKEALISDVRGMLCLYEAAHLRVHGEDIIAKALAFTTTHLKAMVESLGYHLAEQVAHALNRPIRKGLERLEARWYISVYQDEAFHDKTLLELAKLDFNLVQSLHKEELSKSCKVVERIRLCYKVTFCTRQIG

>Vv01036343001

CIDDINDDLYDVALRFRLRQQGFNISCDIFNKFTDERGRFKEALISDVRGMLGLYEAAHLRVHGEDILAKALAFTTTHLKAMVESLGYHLAEQVAHALNRPIRKGLERLEARWYISVYQDEAFHDKTLLELGKLDFNLVQSLHKEELSNLARWWKELDFATKLPFARDRLVEGYFWMHGVYFEPQYLRGRRILTKVIAMTSILDDIHDAYGTPEELKLFIEAIERWDINSINQLPEYMKLCYVALLDVYKEIEEEMEKEGNQYRVHYAKEVMKNQVRAYFAEAKWLHEEHVPAFEEYMRVALASSGYCLLATTSFVGMGEIATKEAFDWVTSDPKIMSSSNFITRLMDDIKSHKFEQKRGHVASAVECYMKQYGVSEEQVYSEFRKQIENAWLDINQECLAVSMPLLARLLNLTRTMDVIY

>Vv01036344001

CIDDINDDLYDVVLRFRLLRQQGFNISCDIFNRYTDEKGRFKESLINDAYGLLGLYEAAHLRVWEEDILDEALAFTTTHLKSMVEHLEYPLAAQVTHALYRPLRKGLERLEARPFMSIYQDEASHSKALLKLAKLDFNQLQSLYKKELSNILGWWKDLDFSSKLPFVRDRLVEGYFWIAIACFEPQYSYARRIQTKLHALMTTTDDIFDAYGTLEELEFFTEAIGRWDIDSTHQLPEYMKPCYQAVLDAYKEIEDMENTERSHSVHQAKDAMKNLVQAYLVEAKWFHGKYIPTIEEYMRVALVSIGAPVLTFISFIGMGEIATKEVFDWLQQNPKIVRASSKVIRLMDDMATHKFEQERGHIASSIECYMKQHGVSEQQAYEEFHKQLENAWKDINEECLAVPMLLLSRLLNFARAADVMY

>Vv01036348001

NGDDMEGDIYNVALQFRLLRQAGFNISCGLNEFKDEKGNFKKALISDVRGMLGLYEAAHLRVRGEDILDEALAFTTTHLRSMVEHLEYPFAEQVVHALKQPIRRGLERLEARWYISIYQDETSHDRTLLKLAKLDFNLVQSLHKEELSNISRWWKKLDFATKLPFARDRLVECYFWILGFYFEPQYVWARRILTKTIALTSTMDDIYDAYGTFEELKLFTAAIERWDINSIDHLPEYMKHFYVALLDVYKEIEEEMEKEGNQYRVQYAIEAMKNQARAYFHEAKWLHEGRIPTVEEYMSVAQVSSGDSMLTITSFIGMGIVTKEAFDWVITNPKIVTASSVISRLMDDITSHKFEQKRGHVASGVECYMKQYGASEEEVYDKFQKQVEDACKDINEEFLAVPMPLLMRVLNLSRVMYVIY

>Vv01036351001

KWDINSIDQFPEYLKPCYQAVLDVYKEIEEMENTERSYCVHHTKDAIKSLFQANLVQAKWFRGKYIPTIEEYMGIAMVTVGVPPLTIMSFIGMRETATKEVFDWVQQNPKIVRAASTVMRLMDDMASHKFEQERGHNASSIECYMKQHGVSEQQAYDELHKQIENAWKDINEESLAVPMLLLSRLLNFARSGDVMY

>Vv01036360001

NGDDMEGGIYNVALQFRLLRQAGFNISCGLFNEFKDEKGNFKKALVSDVRGMLGLYEAAHLRVHGEDILDEALALTTTHLRSMVEHLEYPFAEQVAHALKQPIRKGLERLEARWYISIYQDETSHDRTLLKLAKLDFNLVQSLHKEELSNITRWWKQLNFATKLPFARDRLVEGYFWILGVFFEPQYAWARRIVTKTIAMTSTMDDIYDAYGTFEELELFTEAIERWDINSIDHLPEYMKHFYVALLDVYKEIEEEMEKEGNQYRVQYAIEAMKKQVRAYFHEAKWLHEGRIPTMEEYMSIARVSSGYPMLTATSFIGMGKIVTKEAFDWVISDPKIVTASAVIARLMDDITSHKFEQKRGHVASGVECYMKQYGASEEEVYDKFQKQVVDAWKDTNEEFLAVPMPLLMRVLNLSRVMYVIY

>Vv01036361001

GIADINDDLYDVALRFRLLRQQGFNISCDIFKRYKDEKGRFKKSLINDPYGLLGLYEAAHLRVWEEDILDEALPFTTTHLKSIVEHLEYPLAAQVTHALERPIRKGLERLEARPFMSIYQDEASHSKALLKFAKLDFNLLQSLYKKELSNISRWWKDLDFSSKLPFARDRLVEGYFWIATCCFEPQYSYARRIQTKLHALITTMDDMFDAYGTFEELELFTEAIGRWDINSVHQLPEYMKPCYQAVLDVYKEIEEMENTERSYCVHHTKDAIKSLVQAYLVEAKWLHGKYIPTIEEYMGIAMVTVGVPVLTIMSFIGMRETATKEVFDWLLQNPKIVRATYIIIRLMDDMASHKFEQEREHIASSIECYMKQHGVSEQQAYDEFHKQTENAWKDINEECLTVPMLLLSRLLNFARSGDVMY

>Vv01036366001

DLNDIDGDLYNVALGFRLLRQQGYSISCGIKKFTDERGRFKEVLITNVRGLLGLYEAAHLRVHGEDILAEALTFTTTHLKAMVESLGYPLAEQVVHALNRPIRKGLERIEARWYISVYQDEAFHDKTLLELAKLDFNLVQSLHREELSNLARWWKELDFATKLPFARDRLVEGYFWILGVYFEPQYLRARRILTKVIAMTSILDDIYDAYGNPEELKLFTEAIERWDINSIDQLPEYMKLCYAALLDVYKEIEEEMEKEGNQYRVHYAKEVMKNQVRAYFAEAKWLHEEHVPTIEEYLRVALVSSGYCMLATTSLVGMGEIATKEAFDWVTSDPKIMSSSNFIARLMDDISSHKFEQKREHVASAIECYMKQYGVSEEQAYSEFRKQIENAWMDINQECLAVPMPLLARVLNLTRAADVIY

>Vv01036367001

GYNISCNVFNKFKDKNGSFRESLIGDVQGMLGLYEAAHLRVQEEDILDEALAFTTTHLKSLVKHLDHPLAVQVTQALHRPIRKGLERLEARPYISIYQDEASHSKALLKLAKLDFNLLQSLYKKELSHITRWWKDLDFSSRLPFRQLRGMRWDNTGIDKLPEYMKPCYRAVLDAYKEIEEKENEERSYCVHYAKEAMKNSVRAYFNEAKWLHGEYVPTVEEYMGVALVSCDVPMFTIISFVGMGIATKEAFDWVLNGPKIVRACSTIIRLMDDMASHKFEQERGHIASSVECYMKQYSVSNQHAYHELNKQVEKAWKDINQEFLAIPMPLLTRVLNFARTG

>Vv01036370001

MTSILDDIYDAYGNPEELKLFTEAIERWDSNSIDHLPEYMKLSYMALLDVYKEIEEEMEKEGNQYRVHYAKEVMKNQVRAYFAEAKWLHEEHRPTIEEYMRVAVVSSGYYLIATTSLVGMGERATKEAFDWVTNDPKIMSSSSLIARLMDDIRSHKFEQEREHVASAIECYMNQYGSEEQAYNNFQKQIENAWMDINQECLDVPMPILALILNLARAADVFY

>Vv01036372001

MTSILDDIYDAYGNPEELKLFTEAIERWDINSIDQLPEYMKLCYAALLDVYKEIEEEMEKEGNQYRVHYAKEVMKNQVRAYFAEAKWLHEEHVPTIEEYLHVALVSSGYCMLATTSLVGMGEIATKEAFDWVTSDPKIMSSSNFIARLMDDISSHKFEQKREHVASAIECYMKQYGCL

>Vv01036374001

YSDDKYDDLYNVSLRFRLLRQQGYNISCDVFNKFKDKNGSFRESLIGDVQGMLGLYEAAHLRVQEEDILDEALAFTTTHLKSLVKHLDHPLAVQVTQALHRPIRKGLERLEARPYIFIYQDEASHSKALLKLAKLDFNLLQSLYKKELSHITRWWEDLDFSSSLPFVRDRVVETYLWIVVACFEPQYSYARRIQTKLLVLITVIDDIYDAYGTLEELELFTEAIERWDNNGIEKLPEYMKPCYLAVLDAYKEIEEKENEERSYCVHYAKEAMKNSVRAYFNEAKWLHGEYVPTVEEYMGVALVSCDVPMFTIISFVGMGIATKEAFDWVLNGPKIVRACSTIIRLMDDMASHKFEQERGHIASSVECYMKQYSVSEQLAYHELNKQVEKAWKDINQEFLAIPMPLLTRVLNFARTGDFMY

>Vv01036376001

HSDDKYDDLCNVSLRFRLLRQQGYNISCDIFERFKDENGSFKECLNNDVEGMLGLYEAAHLRVQEEDILDEALAFTTAHLESLVEDLDYPLAAQATQALYRPIRKGLERLEARPHISIYQDEASHSKALLELAKLDFNLLQSLYKKELSYITRWWKDLDFSSKLPFVRDRVVETYLWIVAECFEPQYSYARRIQTKLLVLITVIDDVYDAYGTLEELELFTEAIERWDNNSIDSLPEYMKPCYQAVLDVYKEIEEKENEERPYCVHYAKKAVRSVRAYFNEGKWLHAEYVPTMEEYMGVALVSSDVPMFTIISFVGMGMATKEAFDWVLNGPKIVRACSTIVRLMDKYYFQNFEQERGHSASSVECYMKQNSVSEQLAYRELNKQVEKAWKDINQEFLAIPMHLLTRVLNFARTGEIFY

>Vv01036724001

REEEIFADRATCAIAFRILRLNGYDISSVPLAQFAEDQYFKFGQDFKDLGAALELFRASEMIIHPDEVVLEKQNSWSSHFLRQGLSNSSIHADRLNKYIAQEVEDALRFPYYANLDRIANRRSIEHYNVDDTRILKTAYRSSHVCNKDFLKLAVEDFNFCQSIHQNELKQLERWIIENRLDKLKFARQKLAYCYFSAAATIFSPEQSDARLSWAKNSVLTTVVDDFFDIGGSEEELLNLIQLVEKWDIDVAVDCSEQVEIVFSALHSTISEIGVKASAWQARNVTSHIIDIWLKLLRSMLQEAQWVSNKSAPTMDEYMTNAYVSFALGPIVLPALYFVGPKLSEEVVEGPECHKLYKLMSTCGRLLNDIHSFKRESKEGKANALALHMIHGGTTEEQAIREMKGLVKSQRRELQRLVLTVPRICKDLFWKMSKVLHTFY

>Vv1002718001

TVSDCHNNLYELALGFRLLRQEGYYVSADVFNNLKDTEGKLQEKLSEDIKGLMGLYEASQLCIKGEDILEEIGNFSSQLLNAWNTHNDHSQARIVRNTLGHPHHKSLARFMAKSFLSDFQGTDGWVNVFRELAKMDFNVVKSIHQKEMLQVSK

>Vv1007468001

TVSDCHNNLYELALGFRLLRQEGYYVSADVFNNLKDTEGKLQEKLSQDIKGLMGLYEASQLCIKGEDTLEEIGNFSSQLLNAWNTHNDHSQARIVRNTLGHPHHKSLAKFMAKSFLSDFQGTDGWVNVFRELAKMDFNVVKSIHQKEMLQVSK

>AT1G31950

DIIVGEDDLETISIMFEVFKLYGHKMSCDAFDRFRGNDGRFKESLVRDFRGMLQLFEVAHLGTPCEVIMDEALSFTRNHLESLTSGNASTASPHLLKHIQNSLYIPRYCNIEVLVAREYISYYEQEEGHDEILLKFAKLNFNFCQFHYVQELKTLTKWWRDLDLASKLPYIRDRLVESHLVALGPYFEPHYSLGRIIVAKINMIMVVVDDTYDAYATLPQVKALTECLQSIEVSDLPDYLRIVLGSLFDVMGEIEREMRPLGRLYRVKQVVEKIKIITKAYQEIAKWARTGHVSTFDEYMKVGVLTAGMADYAAYCFIGMEDINEKEAFEWLNSNPLIIKHLTAMFRLANDVGTYETEINRGEVANGLNCYMKQYGVTKEEASRELRKMYVYRKKVVVEEFMHVPRQVLLRCLNIARIFDVFY

>AT1G33750

MLFTDEDDLETTAIMFEVFRLYGHKISCDVFDRFKGVDAKFKEHLVSDVRGMLQLYEAAHLATPFETILDEALSFTRYHLESLAGQQATAPHISRHILNALYKPRFLKMEIIAAREYIHFYQKEGHDETLLKFAKLNFNFCQLHYVRELKTLTKWWKDIDLPYKLPYIRDRLLETFIGVMAVYLEPHYSLGRIIATKVSQVIVVMDDTCDAYGTFSEVRSLIDSLERWDPGAIDKLPSCLRIVIQSIVETMEDIEREMKPRGRSSSVQDTVEEIKIMGRAYAEISKWARAGHVPTFDDYIELGLDSSGIRCFAMYSFISMEDCEENQTNAWFKSKPKMLRALSVIFRLTNDIAGFEEEMRRGEVVNGVNCYVKQHNVTKELAVREIKKMIRDNYKIMMEEFLSVSRPILVRCFNIVRLVNLYY

>AT1G48800

EMMDGENDLYTVSIIFWVFRTYGHNISSDIFNRFKGHNGKFKECLATDAKGILSLYEAAHMGTTTDYILDEALSFTLSYLESLAANGTCKPNLVRRIRNALGLLQNKNVEILVAKEYIRFYEQEEDCDKTILEFSMLNLKFLQLHYLQELKLLTKWYKEQDFESKLPPYYRDRIVELHLATLAYINPKYSRVRIILTMIYTIQIILDDTCDRYASLREVESLAATIERWDHHAMEGLPDYLKSVAKFIFHTFQEFEREVSSESGGSYSLKATIEDCKRMMRSNLQLAKWAVTGHLPSFDEYLDVAGVEIAVYFTVAGILLGMENINKKEAYEWLIFRDKLVRAMSTKARLVNDLFGYKDDMRRGYVTNSINCYKKQYGVTEEEAFRKLHQMVADGDKMMNEEFLNVPHQVLKAVLDTLRAINICY

>AT1G48820

EMMASEDDLRFKGDNGKFKECLAKDAKGILSLYEAAHMGTTTDYILDEALSFTLTYMESLAASGTCKINLSRRIRKALDQPQHKNMEIIVAMKYIQFYEEEEDCDKTLLKFAKLNFKFLQLHYLQELKILSKWYKDQDFKSKLPPYFRDRLVECHFASLTCFEPKYARARIFLSKIFTVQIFIDDTCDRYASLGEVESLADTIERWDPHAMDGLPDYLKSVVKFVFNTFQEFERKCKRSLRINLQVAKWVKAGHLPSFDEYLDVAGLELAISFTFAGILMGMEVCKPEAYEWLKSRDKLVRGVITKVRLLNDIFGYEDDMRRGYVTNSINCYKKQYGVTEEEAIRKLHQIVADGEKMMNEEFLNVPYQVPKVILDTLRAANVSY

>AT1G61120

PMSYLADQLHKDSLAFRMLRMHGRDVSPRSFCWFLNQETRNHLERNIDSFLLVILSVYRATDLMFPGEHDLQEAREYTRNLLEKRRSIKEMIMHELSTPWIARLKHLDHRMWIEDKNSNVLSMEKASFLRLHSSYSDKLTHLAARNFEFQQAKYCRELEELTMWVKKWGLSDIGFGREKTTYCYFATVTSLPYEYAIKFGKLAAKTAILITIADDFFDEKGSFNDLEGLTKAVLRWEGELKSYGNIIFRALDDIVRETANTCRTHHKTDIIVHLRNIWGETFESWLREAEWSKKGHTSSMDEYIRNGMISIAAHTIALSISCLMEPCFPHNKLKPGNYDSITTLLMIIPRLLNDLQSYQKEQEQGKMNSVLLHMKNHGLEIEDSIAHIEKIIDSKRKEFLEHVLDLPKPCKEIHMSCCKVFEMFF

>AT1G61680

QFNGDLHEIALRFRLLRQEGHYVQEIIKNILDKKGGFKDVVKNDVKGLTELFEASELRVEGEETLDGAREFTYSRLNELCSGRESHQKEIMKSLAQPRHKTVRGLTSKRMIKIAGQEDPEWLQSLLRVAEIDSIRLKSLTQGEMSQTFKWWTELGLEKDVEKARSQPLKWHTWSMKILQDPTLTEQRLDLTKPISLVYVIDDIFDVYGELEELTIFTRVVERWDHKGLKTLPKYMRVCFEALDMITTEISMKIYKSHGWNPTYALRQSWASLCKAFLVEAKWFNSGYLPTTEEYMKNGVVSSGVHLVMLHAYILLGEELTKEKVELIESNPGIVSSAATILRLWDDLGSAKDENQDGTDGSYVECYLNEYGSTVDEARTHVAQKISRAWKRLNRECLPFSRSFSKACLNIARTVPLMY

>AT1G66020

EMMEDEDDLCTVSIIFWAFRRYGHYISSDVFRRFKGSNGNFKESLTGYAKGMLSLYEAAHLGTTKDYILQEALSFTSSHLESLAACGTCPPHLSVHIQNVLSVPQHWNMEILVPVEYIPFYEQEKDHDEILLKFAKLSFKLLQLQYIQDLKIVTKWYKELEFASKLPPYFRDNIVVNYFYVLAVIYTPQHSYERIMLTQYFTCLAILDDTFDRYASLPEAISLANSLERWAPNDAMDQPDYLKIVLNFILKTFEVFQKELEPEGRSYTVKATIEEFKTVTKGNFDLAKWAHAVHVPSFEEYMEVGEEEISVCSTLAGIFMCMEKATKEDYEWLKSRPKFIQTLCARCRLKNDITGFEDDMSRGYVTNAVNCYMKQYGVTKQEAFGELNKIIVEADKILNEEFLGVRHCVLKATFDLARMIFITY

>AT1G70080

IIDCKEDDLYTVSIIFRVFRLYGHYITPDIFNRFKGDDGNFKKCLNDDVRGMLSFYEASHFGTTTEDILEEAMSFTQKHLELFLVGEKAKHYPHITKLIQAALYIPQNFNLEILVAREYIDFYELETDHNEMLLKLAKLNFRFLQLQYIQDLKTLTTWWKELDLVSKIPVYFRERLAEPYFWATGIYYEPQYSAARIMLAKSIILVDIVDNTFDVYGTIDEVKSLVQAIERWDSDAVDVLPDYLKVVFRTTFDLFKELEEYVSSEARSFTMQYAYEQLRILMKGYLQEAEWSNRGHLPSHEEYIEVGVASTAGEVLLAMTFIPMGDAAGVGVYEWLRSRPKLTHALFVKSRLRDDIATYKEEMKRGDVCNGINCYTKQHKVSEEEACIEFEKKTNHMSKVMNEEFLFIPLHILRPVLNYGRLADVCY

>AT1G79460

GDEEICLDLATCALAFRLLLAHGYDVSYDPLKPFAEESGFSDTLEGYVKNTFSVLELFKAAQSYPHESALKKQCCWTKQYLEMELSSWVKTSVRDKYLKEVEDALAFPSYASLERSDHRRKILNGSAVENTRVTKTSYRLHNICTSDILKLAVDDFNFCQSIHREEMERLDRWIVENRLQELKFARQKLAYCYFSGAATLFSPELSDARISWAKGGVLTTVVDDFFDVGGSKEELENLIHLVEKWDLNGVPESSEHVEIIFSVLRDTILETGDKAFTYQGRNVTHHIVKIWLDLLKSMLREAEWSSDKSTPSLEDYMENAYISFALGPIVLPATYLIGPPLPEKTVDSHQYNQLYKLVSTMGRLLNDIQGFKRESAEGKLNAVSLHMHERNRSKEVIIESMKGLAERKREELHKLVLVVPRECKEAFLKMSKVLNLFY

>AT2G23230

TTMAGENDLSTVSVMFWVSGHMDINMFRRFKGEDGKFEECHTKDVKGLLSLYEAAQLGTSTEDILDEAMSFSSSHLECLAGGTCPPHISRLIQNELYMPQHHNAEILFASEYISFYKQEDVHNKVLLEFAKLNFKFLQLHWIHELKILTKWWNDQDLLSKLPPYFRDRMVECHLYAVIYYFEPQYSFGRIMLAKLLVLLTVVDDTCDRYGSVPEVAKLLDCVERWDPELGESLPDYLKTVFKFTLDVFEDCERAGKSEEGLSFNVDGALAERTHLNFAEWAAAEKVPTVEEYLEVGGVAVTMYATIALGLLGLGKAREHGYEWLKSRPKLVHDLATKGRLMNDMGGFKDDIGRGFLANVVNYYMKEYGTTEEETYKEFHKIVRDLEKSVNSEFLGVPREILSRALNCGKMIDVTY

>AT2G24210

TNIQESDLHATALEFRLFRQHGFNVSEDVFDVFMENCGKFDRDDIYGLISLYEASYLSTKLDKNLQIFIRPFATQQLRDFVDTHSNEDFGSCDMVEIVVQALDMPYYWQMRRLSTRWYIDVYGKRQNYKNLVVVEFAKIDFNIVQAIHQEELKNVSSWWMETGLGKQLYFARDRIVENYFWTIGQIQEPQYGYVRQTMTKINALLTTIDDIYDIYGTLEELQLFTVAFENWDINRLDELPEYMRLCFLVIYNEVNSIACEILRTKNINVIPFLKKSWTDVSKAYLVEAKWYKSGHKPNLEEYMQNARISISSPTIFVHFYCVFSDQLSIQVLETLSQHQQNVVRCSSSVFRLANDLVTSPDELARGDVCKSIQCYMSETGASEDKARSHVRQMINDLWDEMNYEKMILHHDFMETVINLARMSQCMY

>AT3G14490

DIIAKENDLETISTMFEVFRLRGYYMPCYAFNRFKGEDGRFKESLAEDIRGMLQLYEAAHLGTPSEDIMDEALSFTRYRLESLTSNHTATASPHLSKHIQNALYRARYHNLEILVAREYISFYEQEEDHDETLLKFAKLNFNYCQLHYIQELKDLTKWWKELDLASKLPYIRDRIVEVYFGALALYFEPRYSLGRIIVTKITMIVTVFNDTCDAYGTLPEVTSLVDSFQRWDLGDIEKLPSYVKIVFRGVFETLEEIEQEMRPQGRSRIVQVAVDEIKKLGKAYLAISKWARASHVPTFEEYMEFGMQTSMDHFAAYSFIAMEDCDENQTCEWYKSRPKMMEALNGVFRIKNDINTFEQEMSRGEVAKGLNCYMKQHGVSKEEAIGEMNKIYSNYYKIIMEEYLAVPRPILVRCLNVSRPIHHFY

>AT3G14520

DIIVGEDDLETISIMFEVFRLYGHKMSCDAFDRFRGEDGRFKESLAKDVRGMLQLFEVAHLGTPSEDIMDEASSFAQNHLDSWIGGNVSGATPHLLKHIQNSLYIPRYCNIEVLVAREYISYYEQEEGHNKILLKFAKLNFNFCQFHYIQELKTLTKWWKDLDLASKLPYIRDRLVESHLGGLGPYFEPHYSLGRIIVAKIIMTMVVVDDTYDAHATVPEVAVLTECLQRLNIGADDKLPDYLRTVLESVFEVMGEIEQEMRPKGRSYGVKQVLERFKNVAKADKQLTEWARTGDVPSFDEYMKVGLVTAGMDGYAGYCFIGMEDVSEKEAFEWLSSNPLIIQALNVMFRLANDVGTYETEINRGEVANGLNCYMKQYGVTKEEASQELRKIYSNNKKVVMEEFMHVPRQVLLRCLNFARLFDVMY

>AT3G14540

GIIVEEDDLETISIMFEVFRLYGHKMSCDAFDRFRGGDGRFKESLAKDVRGMLQLFEVAHLGTLSEDIMDEALRFTRNHLESLTSGNVSSASPHILKHIQNSLYIPRYCNIEVLVAREYISYYEQEEGYNEILLKFAKLNFNFCQCHYIQEIKTLTKWWKDLDLASKLPYIRDRSVESHLGGLGPYFEPQYSLGRIIVAKTIMIIVVADDTYDAHATIPEATVLTEYFQRLNIGADDKLSGYLRIVLESVFEVMGEIEQEMSPKGRSYSVKQVLERFKIIAKAYKQLTEWARKGHVPTFDEYMKVGLVTAGMGDYAGYCFIGMEDINEKEAFEWLNSNPLLIDALNVLFRIANDVGTYETEINRGEVANGLNCYMKQYGVTKEEASRELRKMYIYNKKVVVEEFMRVPRQVLLRCLNFARLFDVIY

>AT3G25810

WKCDKEEDLHATALEFRLLRQHGFGVSEDIFDVIIDKIESNTFKSDNITSIITLYEASYLSTKSDTKLHKVIRPFATEQIRNFVDDESETYNIMLRMAIHALEIPYHWRMRRLETRWYIDAYEKKHDMNLFLAEFAKIDFNIVQTAHQEDVKYVSCWWKETGLGSQLHFVRDRIVENYFWTVGMIYEPQFGYIRRIVAIVAALITVIDDIYDIYGTPEELELFTAMVQNWDINRLDELPEYMKLCFLTLFNEINAMGCDVLKCKNIDVIPYFKKSWADLCKAYLVEAKWYKGGYKPSVEEYMQNAWISISAPTMLIHFYCAFSGQISVQILESLVQQQQDVVRCSATVLRLANDLATSPDELARGDVLKSVQCYMHETGVSEEEARTHVQQMISHTWDEMNYETALLSRRFVETAMNLARMSQCMY

>AT3G25820

WKCDKEEDLHATSLEFRLLRQHGFDVSENIFDVIIDQIESNTFKTNNINGIISLYEASYLSTKSDTKLHKVIRPFATEQIRKFVDDEDTKNIEVKAYHALEMPYHWRMRRLDTRWYIDAYEKKHDMNLVLIEFAKIDFNIVQAAHQEDLKYVSRWWKDTCLTNQLPFVRDRIVENYFWTVGLIYEPQFGYIRRIMTIVNALVTTIDDIYDIYGTLEELELFTSMVENWDVNRLGELPEYMRLCFLILYNEINGIGCDILKYKKIDVIPYLKKSWADLCRTYLVEAKWYKRGYKPSLEEYMQNAWISISAPTILIHFYCVFSDQISVQNLETLSQHRQHIVRCSATVLRLANDLGTSPTELARGDVLKSVQCYMHETGASEERARDHVHQMISDMWDDMNSETKSRSRGFKEAAMNLARMSQCMY

>AT3G25830

WKCDKEEDLHATSLEFRLLRQHGFDVSENIFDVIIDQIESNTFKTNNINGIISLYEASYLSTKSDTKLHKVIRPFATEQIRKFVDDEDTKNIEVKAYHALEMPYHWRMRRLDTRWYIDAYEKKHDMNLVLIEFAKIDFNIVQAAHQEDLKYVSRWWKDTCLTNQLPFVRDRIVENYFWTVGLIYEPQFGYIRRIMTIVNALVTTIDDIYDIYGTLEELELFTSMVENWDVNRLGELPEYMRLCFLILYNEINGIGCDILKYKKIDVIPYLKKSWADLCRTYLVEAKWYKRGYKPSLEEYMQNAWISISAPTILIHFYCVFSDQISVQNLETLSQHRQHIVRCSATVLRLANDLGTSPTELARGDVLKSVQCYMHETGASEERARDHVHQMISDMWDDMNSETKSRSRGFKEAAMNLARMSQCMY

>AT3G29110

EIIAGEDDLYTISTIFWVFRTYGYNMSSDVFRRFKEENGKFKESLIEDARGMLSLYEAAHLGTTTDYILDEALDFASNNLVSLAEDGMCPSHLSTHIRNALSISQHWNMEIIVAVQYIRFYEQEVGHDEMLLKFAKLNFNLVQRLYLQEVKILTKWYKDQDIHSKLPPYYRPVVTEMHFFSTATFFEPQFSHARILQTKLFMAELLVDDTCDRYATFSEVESLINSLQRWAPDAMDTHPDYLKVVFKFILNAFEECEKELRPQGRSYSLEQTKEEYKRFAKSNLDLAKLAQAGNVPSFEEYMEVGKDEIGAFVIVAGSLMGMDNIDAVEAYDFLKSRSKFSQSSAEIVRYLNDLAGFEDDMRRGCVSTGLNCYMNQYGVTETEVFREFRKMVMNTCKIMNEEFLDVPLRVLKTNFSCVRSGFVGY

>AT3G29190

EMMANEEDLYTVSIIFWVFRRYGHYISSDFFRRFKGNDGNFKKSLIGDAKGMLSFYEAANMATTKDYILDEALSFTSSHLESLAANGACPPHMSRRIRNALNASQHWNMEMLVAVEYISFYEKEKDHNEMLLKFSKLNFKFLQLQYLQELKVLTKWYKEVDFVSKLPPYFRDRIVENHFFIQTLFVESQHSRARIMMAKYFILLVIQDDTLDRYASLPEAESLVNSLNRWAPDHAMDQPDYLKFVFKFILDTFEEFEKELRPEGGSFGVCATIEEFKSLVKANLEAEKWALADNMPSFEEYIEVTGVGITAMTTLMGAMMCMGKIVPKEDYKWLKSRPKIIQALAIKGRLMNDMKGYKEDMSRGYAANAVTCYMKQYRVTEQEALKEFEKMVAVANKTVNEEFLGVSRLVLKLAMGVGLMISITY

>AT3G29410

CLISDEDDLETIAIMFEVFRLYGHKMPCDVFERFKSEDGKFKESLVGDVRGLLQLYEAAHLGAPSEDIMDEALSFARYHLEPLAGTETSSNLFKHVENVLYRARYHSIEILVARQYISFYDQEEDQDETLLRFSKLNFNFCQMHYVKELKIVTRWWKELGIASKLPYSIRERNVETYLGGLGVLFEPRYSLARIFLAKLTLIMTVVDDTCDAYATLPEVQSLHDAFHRWDLRAMEELPRYMRIIYQSVFETVEDIDREMIARGKHGRLQLTIDEIKSLMIWYLGIAKWARSDQVPSFEDYMEIGTPSSALDDFASYGFIAMDDCDQKQLKEWFYSKPKIFHALNALFRIRNDIVTFEQEMSRGEVANGVNCYMKQHGVTKEAAVEELRKMERESYKIMIEEFMAMPRQILVRPVNIARVMDLFY

>AT3G32030

MIIAEEDDLETISIMFEVFRLYQHKMSCDSFVRFKGEDGRLKESLVGDVRGMLQLYQAAHLGTPSDQYIMEEAKSFTRNHLESLVESTTIPPHFSSHIRDALYIDRYHNMEILVARKYISFYEQEEGHDLTLLKFGKLSFNYCRLHYIQELKTLTKWWKDQDIPSNLPCVRDRIVETYFPTLGLYFEPRFSLGRIIIAKMTIIVVALNDVCDSYATYPEAKSLIDSLQRWDIEAIDELPNYSRIVLRLILETIGEIEREMKPRGRSASVQHTIDETKSLGRAYLALSKWASEGYMPTFDEYMEVGEVTGGMDDFALYSFIAMEDCDEKPLYEWFDSKPKILQALSVLYRINNDIVTYEREMSKGEVVNGVNSYMNQHGVTKEEAVEELRKMARDNYKIVMEELLDVPRPVLVRCLNLARLFDVFC

>AT4G02780

ARCSHVQDIDDTAMAFRLLRQHGYQVSADVFKNFEKEGEFFCFVGQSNQAVTGMFNLYRASQLAFPREEILKNAKEFSYNYLLEKRERELIDKWIIMKDLPGEIGFALEIPWYASLPRVETRFYIDQYGGENDVWIGKTLYRMPYVNNNGYLELAKQDYNNCQAQHQLEWDIFQKWYEENRLSEWGVRRSELLECYYLAAATIFESERSHERMVWAKSSVLVKAISSSFSSDSSFDQFHEYIANARRSDHNDRNDRPVQASRLAGVLIGTLNQMSFDLFMSHGRDVNNLLYLSWGDWMEKWKLYGDEGEGELMVKMIILMKNNDLTNFFTHTHFVRLAEIINRICLPRQYKARRNDEKEKTIKSMEKEMGKMVELALSESDTFRDVFLDVAKAFYYF

>AT4G13280

DITGDEKDLSTISIMFRVFRTYGHNLPSSVFKRFTGDDGKFQQSLTEDAKGILSLYEAAHLGTTTDYILDEALKFTSSHLKSLLAGGTCRPHILRLIRNTLYLPQRWNMEAVIAREYISFYEQEEDHDKMLLRLAKLNFKLLQLHYIKELKSFIKWWMELGLTSKWPSQFRERIVEAWLAGLMMYFEPQFSGGRVIAAKFNYLLTILDDACDHYFSIHELTRLVACVERWSPDGIDTLEDISRSVFKLMLDVFDDIGKGVRSEGSSYHLKEMLEELNTLVRANLDLVKWARGIQTAGKEAYEWVRSRPRLIKSLAAKGRLMDDITDFDSDMSNGFAANAINYYMKQFVTKEEAILECQRMIVDINKTINEELLSVPGRVLKQALNFGRLLELLY

>AT4G13300

DITGDENDLSTISIMFRVFRTYGHNLPSSVFKRFTGDDGKFERSLTEDAKGILSLYEAAHLGTTTDYILDEALEFTSSHLKSLLVGGMCRPHILRLIRNTLYLPQRWNMEAVIAREYISFYEQEEDHDKMLLRLAKLNFKLLQLHYIKELKTFIKWWMELGLTSKWPSQFRERIVEAWLAGLMMYFEPQFSGGRVIAAKFNYLLTILDDACDHYFSIPELTRLVDCVERWNHDGIHTLEDISRIIFKLALDVFDDIGRGVRSKGCSYYLKEMLEELKILVRANLDLVKWARGNQLPSFEEHVEVGGIALTTYATLMYSFVGMGEAVGKEAYEWVRSRPRLIKSLAAKGRLMDDITDFESDMSNGFAANAINYYMKQFVTKEEAILECQKMVVDINKIVNEELLTVPRRVLKQALNFGRLLEVLY

>AT4G15870

EMIAGEDDLYTISIMFWVFRTYGYNMSTDVFKRFKGENEKFMESITSDVKGMVSLYEAAHLRTTREDILEEALSFTTRNLESLARAGASSPHILMRIRNALCMPQHYNAEMIFAREYISFYEQEEDHNKMLLRFAKINFKFLQLNWIQELKTLTKWWKQQDLASKLPPYFRDRLIECYLFAIMIYFEPQFSLGRVSLAKINTVFTLVDDTCDRYGNVSEVAALVQCVERWDPDCMDSLPDYMKTVFKFAWNTFEECENAGIMEEGLSYDVQGALEEWEQGDVVPTFDEYLEIGGVEVTMYVSIACSFLGLGSSREQAYKWLKSRPKFVEAQAKRARLMNDIAGFEGDMSRGFDVNAIMYYMKQYKVTEEETFTRLQKMARDLDTTVNEEILSVPRQILKRAIDFGKMIEFTY

>AT4G16730

EIERETQDLHATSLEFILLRQHGFDVSQDAFDVFISETGEFRKTLHSDIKGLLSLYEASYFSMDSEFKLKETRIYANKRLSEFVAESKTICREDETYILEMVKRALETPYHWSIRRLEARWYINVYEKKHEMNPLLLEFAAIDFNMLQANHQEELKLISSWWNSTGLMKQLDFVRDRITESYFWTIGIFYEPEFKYCRKILTKIFMLIVIMDDIYDIYGTLEELELFTNVVEKWDVNHVERLPNYMRMCFLFLYNEINQIGYDVLRDKGLNVIPYLKQVWTDLFKTFLTESKWYKTGHKPSFEEYMQNGVISSSVPTILLHLFSVLSDHISDQTLTDDSKNHSVVRSCATILRLANDLATSTEEMARGDSPKSVQCYMYETRASEEEARRHMQSMISDSWDIINSDLKSLPRGFLAAAANLNRVVQCIY

>AT4G16740

IDRNRWGDLYATALEFRLLRQHGFSIAQDVFDGNIGVDLDDKDIKGILSLYEASYLSTRIDTKLKESIYYTTKRLRKFVEVNKNETKSYTLRRMVIHALEMPYHRRVGRLEARWYIEVYGERHDMNPILLELAKLDFNFVQAIHQDELKSLSSWWSKTGLTKHLDFVRDRITEGYFSSVGVMYEPEFAYHRQMLTKVFMLITTIDDIYDIYGTLEELQLFTTIVEKWDVNRLEELPNYMKLCFLCLVNEINQIGYFVLRDKGFNVIPYLKESWADMCTTFLKEAKWYKSGYKPNFEEYMQNGWISSSVPTILLHLFCLLSDQTLDILGSYNHSVVRSSATILRLANDLATSSEELARGDTMKSVQCHMHETGASEAESRAYIQGIIGVAWDDLNMESCRLHQGFLEAAANLGRVAQCVY

>AT4G20200

KMMDGEDDLYTVSIIFWVFRRHGYHISYGVFQRFKGSNGNFKESLTRDAKGMLSLYEAANLGTTKDFILEEALSFTSSHLESLAASGTCPPHLSVRIRNALGLSQHWNMEMLVPVEFIPFYEQEIEHDEMLLKFAKLSFKLGQLQYLQELKTLTKWYKELDFATNLPPYFRDRIVEHHFLVQAVFFSPQLSRERIMMIQYFTGLALLDDTFDRYASLHEAESLANSLERWAPDQAMDQPDYLRFVLNFILDTFEEFKRELGPEERSYSVNATIEEFKAAAKANIDLEKWAQADHIPSFEEYMEVGEVEVTVYASLAGIFMSMGMATKEAFEWLKSRPKLVQYLSIKGRLMNDLMGYEDDMSRGYVTNAVNCYMKQYGVTKEEAFRELYKIVVAANKTLNEEFLGVPHFLLKATIDLARMMTVAY

>AT4G20210

EMMAGEDNLYTISIIFLVLRTYGHHMSSDIFQKFKGNDGNFKGCISGDAKGLLALYEAAQLRTTTEYIMEEALSFTSSNLELLAADGRCPPHLSKHIRNALGLSQHKQMEVLVAVEYISFYEQEKDHDKILLKFAKLNFKLMQLHYLEELKVVTKWYKEHDFASNLPPYFKYVIVENHFFAITMYFEPKFSQKRIMLAKYFTVLVLLDDTCDRYASLSEAESLTNSLERWAPDDAMDQPHYLKFVFKFIMGCFEEFERELASEGRSYSVKATLEEFKTIVKANFDFAKLAHTGHVPSFKEYMEVGEVEVGVCATLAGNLMCIGHIGDEGVYEWLKSRPKFLKAASTYGRLMNDIAGFEDDMKREYVITGVNTYMKQYGLTKMEAIRELQNLVEYNHTIMNEEFLDLPRQIRKQVINVARSLNVSY

>AT4G20230

KIMAGEEDLYTVSIIFWVFRRYGHYISSDVFQRFKGSNGSFKESLIGDAKGMLSLYEAAHLATTKDYILDEALIFTSSHLETLVATGTCPPHLLARIRNALSICQHWNFEVLVPLDFIPFYEQEKDHDEMLLKFAKLSFKYLKLIYLQDLKILTKWYKKLDFPSKFPPYFKDRCVENYFFVLPVFFEPQLSSARMLLTKGFILLGIQDDTFDRYASISEAESLGNSLKRWAPDHSMDQPEYLKSVLKVILDTFQEFEKELSPEGRSYSVKYTIEEFQASSKANVELAKWAQVSHVPSFEKYMEVGQMEITACVTVAYILMSMGKTGTKEAFEWLKSRPKLVQSLCTKGRLMNDIAGFEDDMSRGYVVNAVNCYMKQYGVTEKEAFKELRKMVVNTHKTLNEEFLCVSHYVLRETMDFARMIIVTY

>AT5G23960

MVRQEGCDLYTVGIIFQVFRQFGFKLSADVFEKFKDENGKFKGHLVTDAYGMLSLYEAAQWGTHGEDIIDEALAFSRSHLEEISSRSSPHLAIRIKNALKHPYHKGISRIETRQYISYYEEEESCDPTLLEFAKIDFNLLQILHREELACVTRWHHEMEFKSKVTYTRHRITEAYLWSLGTYFEPQYSQARVITTMALILFTALDDMYDAYGTMEELELFTDAMDEWLPVVPDEIPDSMKFIYNVTVEFYDKLDEELEKEGRSGCGFHLKKSLQKTANGYMQEAKWLKKDYIATFDEYKENAILSSGYYALIAMTFVRMTDVAKLDAFEWLSSHPKIRVASEIISRFTDDISSYEFEHKREHVATGIDCYMQQFGVSKERAVEVMGNIVSDAWKDLNQELMVFPFPLLMRVLNLSRVIDVFY

>AT5G44630

DLIADENKLHTISTIFRVFRTYGYYMSSDVFKIFKGDDGKFKESLIEDVKGMLSFYEAVHFGTTTDHILDEALSFTLNHLESLATGRRASPPHISKLIQNALHIPQHRNIQALVAREYISFYEHEEDHDETLLKLAKLNFKFLQLHYFQELKTITMWWTKLDHTSNLPPNFRERTVETWFAALMMYFEPQFSLGRIMSAKLYLVITFLDDACDTYGSISEVESLADCLERWDPDYMENLQGHMKTAFKFVMYLFKEYEEILRSQGRSFVLEKMIEEFKIIARKNLELVKWARGGHVPSFDEYIESGGAEIGTYATIACSIMGLGEIGKKEAFEWLISRPKLVRILGAKTRLMDDIADFEEDMEKGYTANALNYYMNEHGVTKEEASRELEKMNGDMNKIVNEECLTMPRRILMQSVNYARSLDVLY

>AT5G48110

DMNLGEEDMYSISVIFRVFRLYRHKLSSDVFNRFKEENGDFKKCLLDDKKSLTKQWASRGNTWNYFVGGSNEEHLSGHIKNVLYLSQQENAEVVMSREYIQFYEQETHHDETLLKFAKINFKFMQLHYVQELQTIVKWWKELDLESKIPNYYRVRAVECLYWAMAVYMEPQYSVARIILSKSLVLWTIIDDLYDAYCTLPEAIAFTENMERWETDAIDMPDHMKVLLRSLIDLMEDFKGEVRSEGRLYSVEYGIDEWKRLFRADLTISKWARTGYIPNYDEYMEVGIVTGGVDVTVAFAFIGMGEAGKEAFDWIRSRPKFIQTIDLKSRLRDDVATYKDEMARGEIATGINCYMKQYKVTEEEAFLEFHRRIKHTSKLVNEEYFTVPLKLVRIAFNVGRVIDTNY

>EglobTPS002

MSARFSVIPSSSLPQETGCVEGRRSANFHPSIWGDYFLKYASDSACGKLGIAYHFELEIDKELEQIHRGYFEFHCDDNDNDLDTVALLFQLLRQRGYHVSCEIFNKFKDGDGNFGKSLIADVQGLLSLFEACHMRYHGDDNLEDALDLSVCCRWWKGLDVQKKFPFARDRLWWLGEYYEPEHEAAREILTKLISVTSIIDDIYDVYGTWEELELFTRWDVNAKDGLPEYMQECYKIVLDLYDEIGYEFSQKGRSYRLFYAKEVVSHLMILAWSFLGMGDIVTKDVFDWLLFNDPKKASSIIGRLLNDIAGHQFEQERGHVASAVECFMKQYRVTEEEAKEELRKQVTDAWKDINEELRGPTVVPMPILVRILNLTQALHMMYK

>EglobTPS003

EERIERLKGEVGKMLTSAMYKPAEKLNLIDQIQRLGISYHFELEIDKELEQIRKGYFEYHYDDNDNDLDTVALLFRLLRQRGYRVSCEIFNKFKDGDGNFGKSLIADVQGLLSLFEACHLRYHGDDNLEDALAFTTTHLESIDKRKASLHLEKKVSHALNQPIHKGMSRLEARHYIPLYQEEPSHNEVLLSLAKLDFNLVQEQHRKELGNLTRRWWKGLDVQRKFPFARDRLVEMYVWWLGEYYEPEHEAAREILTKLSSVGSIIDDIYDVYGTWEELELFTEAIERYRWDVDAKDGLPEYMQECYKIVLDLYDEIGYEFSQKGRSYRLFYAKEVVSHMKNQARAYLVEAKCFHQNHVPTMEEYMSIALPSSGIVSILAWSFLGMGDIVTKDVFDWLLFNDPKMVKASSIIGRLLNDIAGHQFEKERAHVASAVECFMKQYRVTEEEAKEELRKQVTNAWKDINEELRRPTVVPMPILVRILNLTQALHMMYTGETDHYTNAGTKMKEVVTSLLVDPLPM

>EglobTPS004

VEEQIEELKGEVRKMLTDVVDKPSQMLHLIDQIQRLGIDYHFEHDIDEHLEEIHKHYSRLDHGDFKGDDFHMVALIFRLLRQQGYDVSHDRTEVFNKFKDSEGNFRASLTSDVCGLLSLYEACHLRCHGDTILEEALPFAITHLESINESKVSTSFAKQVSHALKQPLRKGLPRLEARHYISLYQEDPSHDEVLLTLAKLDFNLLQEQHQKELGKITRFINWWKDIDVPRNFPFARDRIVELFFWVSGVYFEPEFVEARDILTKVIALTSILDDIYDVYGTLEELVLITEAIQKYVIRWDVDAIDVLPEYLQAYYKELLHLYEEIGNEVAAKGRSYRLVYAKETVMKRQARAFFQEAKWFQTNYTPTMEEYMPLQLKTTGYGMLATTSLVGMGDVVTKHAFEWSLSDCKIVKAAETICRLMDDISSHEQKRGHLVSSVELLMKEHGFSEQEAEKELRKRVNDAWKDTNEEFLRPTAVPMPILTRVLNLSRAMDVLYSDGDNYTHSGTKLKGYVTSLFVSPLPM

>EglobTPS005

VEEQIEELKGEVRKMLTNAVDKSSQMLHLIDQIQRLGIDYHFDRDIDEHLEQIHKHYSRLDHGDFRGDDLHMVALIFRLLRQQGYDVSSGVFNEFKDSEGNFRASLINDVPGLLNLYEACHLRCHGDAILEEALPFATTYLESINESKVSTSFAKQVSHALKQPLRKGLPRLEARHYIPLYQEEPSHDEVLLTLAKLYFNLLQEQHQKELDKITWWKDIDVPRNFTLAREKKVIALTSILDDIYDVYGTLEELVLITEAIQRWDVDAMDVLPEYMQVYYKELLHLYEEIGNEVAAKGRSYRLVYAKETVSIIAFFQEAKWFQTNYTPMMEEYMPLQLTTTGYGMLATTSLVGMGDVVTKHAFEWSLSDCKIVKAAETICRLMDDIFEQKRGHLISSVELLMKEHGISEQEAEKELRKRVNDAWKDTNEEFLRPTA

>EglobTPS006

LKGELRKMLVGAMDKPSQKLNLIDQIQRLGIAYHFEIEIYQQLEQIHKSYFELHDGDKDNDLHTIALLFRLLRQQGYAIEELELFTEAIERWDVEVKDGLPEYMQVCYKIVLDLYDEIDYEVTRKGRSNYLFYAKEAVSHMKNQVRAYFTEAKWFHQNHIPMMEEYMPIALSTIAIELLLVMLLLLGMGDTVTKDVFDWLLYSNPKIVNAMKIVYRLMDDIAGHKFEQERGHGPSSMECFMKQYGVTEEEAKEELHKQVANAWKDINEGLCCSTNVPRQLLVRILNFTRVVHVVYKDEIDLYTHAGTKLKEHVTNLYVNPLPM

>EglobTPS007

SPARETSRVVERRLANFHPSIWGDYFLKYASDIEGLKGELRKMLAGAMDKPSQKLNLIDQIQRLGIAYHFEIEIYQQIHKSYFELHDGDNDNDLHTNALLFRLLRQQGYAISCGMEIFNKFKDINGNFSESLIVDVQGLLSLFEACHMRFHGDDVLNDALAFAMTHLESIDKGKASPNLKKQVRHALKQPIHKGIPRLEARRYISLYQEEPLHNEVLLSLTKLDFNLLQEQHQKELGNLTWKDLDVERKFPFARDRLVEMYLWMSGGYFEPEHKATREILTKVFSIVTIIDDIYDVYGTLEELELFTEAIERWDVEVKDGLPDYMQACYKIVLDFYDEIGYEVTRKGRSDYLFYAKEAVSHMKNQVRAYFTEAKWFHQNHIPTMEESIALPTTAIELLLVMLLLGMGGNVTKDVFDWLLYSDPKMVNAVRMTFEQERGHVGFSKVCSNNIVLQRKRLRRNSINKWPMHGRTLMKGCAATNVPRQLLVRILNFTRVVHVVYKDEIDLYTHAGTKLKEHVTNLYVNPLPM

>EglobTPS008

LKGELRKMLIGAMDKPSQKLNLIDQIQRLGIAYHFEIEIYQQLEQIHKSYFELHDGDNDNDLHTNALLFRLLRQQGYAISCHNTFGTSSVASPNLKKQVRHALKQPIHKGIPRLEARRYISLYQEEPLHNEVLLSLTKLDFNLLQEQHQKELGNLTRRWWKDLDVERKFPFARDRLVEMYLWMSGGYFEPEHKATREILTKVFSIVTIIDDIYDVYGTLEELELFTEAIERRWDVEVKDGLPEYMQACYKIVLDLYDEIGYEVTRKGRSMKNQVRAYFTEAKWFHQNHIPTMEEYMPIALPTTAIELLLVMLLLGMGDTVTKDVFDWLLYSNPKMVNAVKVVCRLMDDIAGHKVFEQERGHGPSSVECFMKQYGVTEEEAKEELHNQVANAWKDINEGLCCSTNV

>EglobTPS009

IEKLKGEVRKMLTDATDKPSQKLNLIDQIQRLGIADHFEIEFHNSNKDGHLHTTALLFRLLQQQGYTISCLVDLYVEHEFFIVSHDNIFSHVLFGHSEIFNKFKDSNGNFNESLIADVQGLLSLFEACHTRFHGDDVLNDALAFTMTHLKSIDEGKASPNLKKQVSHALNQPIHKGIPRLEARQYIPLYQEKPSHNEVLLALAKLDFNLLQEQHQKELGNLTRRWWKNLDIERKFPFARDRLVEMYLWMSIVYFESDYEAAREILTKVASMVSIIDDIYDVNGTLEELGLFTEAIERYNWDINAKEGLPEYMQACYKTVLDLYDEIGYEVTSKGQSYRLFYAKEAVSHLIMKNLVRAYFAEAKWFHLNHVPTMEEYMPIALTSAAVELLLVTSLLGMEDFVTKDAFDWLLYGNSKMVKAVKLVGRLMDDIAGHKFEQERGHGPSSVECFMKQYKVTEEEAKVELRKQVADAWKDINEGLCCPAIVPRPLLVRILNFVRAMHVMYKDEIDIYTHAGTKLKEYVTSLYVNPLPM

>EglobTPS010

SSPTKSDDRIEKLKGEVRKMLTDATDKPSQKLNLIDQIQRVGIAYHFEIEINQQLEQIHDSYFNFHNSNKDGHLHTTALLFRLLRQQGYTISCEIFNKFKDSNGNFSESLIADVQGLLSLFEACHTRFHGDDVLNDALAFTMTHLKSIDEGKASSNLKKQVSHALNQPIHKGIPRLEARHYIPLYQEEPSHNEVLLALAKLDFNLLQEQHQKELGNLWKNLDVERKFPFARDRLVEMYLWMSMVYFEPDYEAAREILTKVASMVTIMDDIYDVHGTLEELGLFTEAIERYWDINAKEGLPEYMQAFYKAVLDLYDEIGCEVTRKGRSYQLFYAKEAVSHLMKNQVRAYFAEAKWFHQNYVPTMEEYMPIALATAAIELLLVTSLLGMEDFVTKDAFDWLLYGNSKMVKAVKLVGRLMDDIAGHKFEQERGHGPSSVECFMKQYEVTEEEAKEELRKQVADAWKDINEGLRCPTIVPRPLLVRILNFARAMHVVYKDEIDIYTHAKTKLEEHVTSLYVNPLPM

>EglobTPS011

IEKLKGEVRKMLTDAMDKPSQKLNLIDQIQRVGIAYHFKIEINQQLEQIHESCFNFHNGDKDSHLHTTALLFRLLRQEGYTISCEIFNKFKDSNGNFNESLIADVQGLLSLFEACHTRFHGDDVLNDALAFTMTHLKSIDEGKASPNLKKQVSHALNQPIHKGIPRLEARQYIPLYQEKPSHNEVLLALAKLDFNLLQEQHQKELGNLTRRWWKNLDIERKFPFARDRLVEMYLWMSIVYFESDYEAAREILTKVASMVSIIDDIYDVNGTLEELGLFTEAIERYNSHSWDINAKEGLPEYMQACYKTVLDFYDEIGYEVTSKGQSYRLFYAKEAVSHLMKNLVRAYFAEAKWFHLNHVPTMEEYMPIALTSAAVELLLVTSLLGMEDFVTKDAFDWLLYGDSKMVKAVKLVGRLMDDIAGHKFEQERSHGPSSVECFMKQYKVTEKEAKVELRKQVANAWKDINEGLCCTAIVPRPLLVRILNFVRAMHVMYKDEIDIYTHAGTKFKEYVTSLYVNPLPM

>EglobTPS012

VEEQVEELKGEVRKMVTNAVDKPSRMLHLIDQIQRLGIDYHFEQEIDAQLERIHKSYSQLDHGDFKGDDLHMVALMFRLLRQQGFNISEVFNNFKDNEGNFKKSLITDVRGLLSLYEACHLRCHGDAILEEALPFAITHLESIDERKVGTSLAKQVSHALKQSKGLPRLEARHYIIFYQEEPSHDKVLLTMAKLDFNLLQEQHQKELGAITWWKNIDVARKFPFARVYFQPEFAVARNILTRVTGLISILDDIYDAYGTLEELVPYTEAIEKYVIRWDVDAMDGLPEYMQAYYKEILNLRSYRLTYAKEAMKKQAKWYFHEAKWFHTGYTPTLEEYIPLALLTTGYEALSITSLVGMGDVVTRDAFEWLLGDCKILSASQIICRFMDDISSHKFEQKRGHVASSSEQEAEEELQKRVVDAWKDINTALMLVLTAILNLSRVMDLLYSNGGDHYTHSKTELKEHITSLFVSP

>EglobTPS013

LKGEVRKMVTNAVDKPSQMLHLIDQIQRLGIDYHFEQEIDAQLERIHKSYSQLDHGDDDLHMVALMFRLLRQQTEVFNNFEDNEGNFKKSLIIDVQGLLSLYEACHLRCHGDAILEEALPFAITHLESIDERKVGTSLAKQVSHALKQPLRKGLPRLEARHYIIFYQEEPSHVAKLDFNLLQEQHQKELGKITWWKNIDVARKFPFARDRIAEMFFWMVGVYFQRNILTRVTGLISILDDIYDAYGTLEELVPYTEAIEKYVIRILLDYQSTCKLIIRRFSISDEIGNDLATKGRSYRLTYAKEKAKWFHTGYTPTLGEYIPLALLTAFEWLLGDCKILSASQIICRFMDDISSHKFEQKRGHVASSVELFMKENHVSEQEAEWRSLHPLQDDHYTHSKTELKEHITSLFVSPLPI

>EglobTPS014

VEEQVEELKGEVRKMVTNAMDKPSRMLHLIDQIQRLGIDYHFEREIDEQLEVFNNFKDNEGNFKKSLITDVRGLLSLYEACHLRCHGDAILEEALPFAVTIDEMKVSTSLAKQVSHALKQPLRKGLPRLEARHYLYQEKPSHDEVLLTLAKLDFNLLQEQHQKELGKITWWKNIDVARKFPFARDRIAELFFWMVGTYFQPEFAMARNILTRVTGLISILDDIYDAYGTLEELVPYTEAIEKYVIRWDVDAMDGLPEYMQAHYKELLNLYDEIGNDLATKGRSYRLAYAKEAMKKQAKGYFHEAKWLHSDYTPTLEEYMPLALLTTGYEALSITALVGMGDVVTRDAFEWLLGDCKILSASQIICRFMDDISSHKFEQKRGHVASSVELLMKENRISEQEAEEELQKRVVDAWKDINEEFLRPTVGPRSVLTLILNLSRVIDVLYTNGDHYTHSKTKLKEHITSLFVSPLPI

>EglobTPS015

VEEQVEELKGEVRKMVTNAVDKPSRMLHLIDQIQRLGIDYHFEREIDEQLEQLDHGDFRGDDLHMGALMRQQGFEVFNNFKDNEGNFKKSLITDVHGLLSLYEACHLRCHGDAILEEALPFAVTHLESIDEMKVSTSLAKQVSHALKQPLRKGLPRLEKVLLTLAKLDFNLLQEQHQKELGGITWWKNIDVARKFPFARDRIAELFFWMVGTYFQPEFAMARNILTRVTGLISILDDIYDAYGTLEELVPYTEAIEKYVIRWDVDAMDGLPKYMQAHYKELLNLYDEIGNDLATKGRSYRLAYAKEAMKKQAKGYFHEAKWLHSDYTPTLEEYMPLALLTTGYEALSITALVGMGDVVTRDAFEWLKILSASQIICRFMDDISSHKFEQKRGHVASSVELLMKENRISDRPTVGPRSVLTLILNLSRVIDVLYTNGDHYTHSKTKLKEHITSLFVSPLLI

>EglobTPS017

EEQVEELKGEVRKMVTNAMDKPSRMLHLIDQIQRLGIDYHFEREIDEQLERIHKSYSQLDHGDFKGDDLHMVALMFPTLFGRTEVFNNFKDNEGNFKKSLIIDVQGLLSLYEACHLRCHGDAILEEALLAKQVSHALKQPLRKGLPRLEARHYIKLYQEKPSHDEVLLTLAKLDFNLLQEQHQKELGKITRSTNRWWKNIDVARKFPFARDRIAELFFWMVGTYFQPEFAMARNILTKVAGLISILDDIYDAYGILEELLVHEQVKGYFHEAKWLHSDYTPTLEEYMPLALLTTGYEALSITALVGMGDVVTRDAFEWLLGDCKILSASFEQKRGHVASSVELLMKEKQEAEEELQKRVVDAWKDINEEFLRPTVGPRSVLTLILNLSRVIDVLYTNGDHYTHSKTKLKEHITSL

>EglobTPS018

ADYHPSIWGDYFLVYEEQIEGLKGEVRKISNPTLGNIQRLGIFYHFKREIDEQLEQIHKSYSQLVHGDFKGDDLHMIALIFRLLRQQGYNVSSGMFVFNKFKNSERNFNVRGLLSLYEACHLRCHGDSILEEALPFAITHLESINESKVSTSLAKQVKRALRQPLRKGLPRLEARYYVPLYQEEPSHDQVLLALAKLDFNLLQEQHQKELGNITRRWWKDIDVATKFPFARDRIVELFFWISGAYFEPEFAVARDILTKVTALISILDDMYDVYGTLEELVILTEAIERWDVDAMDGLPDYMQAWYKVLLDVYDAVGNEVATKERSYRLTYVKEAVFEQKRGHVASSVELFMKEHNVSEQETEKELRKRVVDAWKDINEAFLRPTAVPMPILMRILNLSQVIHVLYSDGDNYTHSGTLLKDHVTSLFISPLPV

>EglobTPS019

NKGSSRVVERRWADYHPSIWGDYFLAYASVEEQIEGLQGEVRKMLTDVVNKPSQVLHLIDQIQRLGIFYHFKREIDEQLEQIHKSYSRLVYRDFKGDHLHMIALIFRLLRQQGYNVSSGMFEVFNKFKDGEGNFRELLITDVQGLLSLYEACHLRCHGDSILEEALLFAITHLESLNESKVSTSLAKQVKHALRQPLHKGLPRLEARHYVPLYQEEPSHDQVLLALAKLDFNLLQEQHQKELGNITRWWKDIDVARKFPFARDRIVELFFWISGAYFEPEFAVARDILTKVIALTSILDDMYDVYGTLEELVILTEAIERWDVDAMDGLPEYMQAWYKVLLDVYDAVGNEVATKERSYRLTYAKEAMKKQARVYFHEAKWFHTNYTPTLEEYMPLALLTTGYEMLAITSLVGMGDVVTNMLLNFEQKRGHVASSVELFMKEHDVSEQETEKELRKRVVDAWKDINEAFLRPTAVPMPILMRILNLSRVIHVLYSDGDNYTHSGALLKDHVTSLFISPLPV

>EglobTPS020

ADYHPSIWGDYFLVVEEQIEGLKGEVRKMLTDAVNKPSQVLHLIDQIERLGIFYHFKREIDEQLEQIHKSYSQLVHGDFKGDDLHMIALIFRLLRQQGYNVSSGMFIADHRCTWTSKPISINESKVSTSLAKQVKRALRQPLRKGLPRLEARDYVPLYQEEPSHDQVLLALAKLDFNLLQEQHQKELGNITWWKDIGIARKFPFARDRIVELFFWISGVYFEPEFAEARNILTKVIALTSILDDSTGEELVSLTKAIEKRWDVDAMDGLPKYMQAWYKLLLNVYDAIGNEVAMKERSYRMKKQARVSFHEAKWFHTNYTPTLEEYMPLALLTTGYEMLAITSLVGMGDVVTKHAFEWLLGDCKILKASQIICRLMDDIASHFEQKRGHVASSVELFMKEHNVSEQETEKELRKRVVDAWKDINEVPVPILMRTLNLSRVIHVLYSDGDNYTHSGTSLKDHVTSLFISPLPVS

>EglobTPS022

KGTSRAVEPRWADFHPSGWGDYFLVYASPTNSMVSGMVEEQIEGLKGEVRKMVTAVVTAVVDKPSQMLLLIDQIQHLGIFYHFEREIDEQIEQIHKSYSRLVHRVLKGDNLHMIALTFQLLQGYHVSSGMFSTEAFNKFNDGEGNFESLITDAHGLLSLYEAWHLRCHGDAIPEEALPFAITHLQWWKDIGIARKFSFARERIVELFFPEFAVARDILIKVTALLSILDNIYNVWYGTLEELVIFTEAIERWNVDAIDGLPGYMQAWSKVLLDVFDAIGNEMIIKERSYRLIYAKESVRSIVMKKQAKVYLYKTKWFYSNTPTLEEYKPLALSTSSYELLLITSLMGMGNVVTKHVFEWLLVNYKILKEGARGICSGVVHTQRFGAGNRKGVVDAWKDINEAFLHPTVVRLSTLMQVVDISQVIHVVYSYG

>EglobTPS024

VERRSADYHPSIWGDYFLVYASPTNSMVSSFSYGHLKGEVRKMLTDAVNKPSQVLPLIDQIERLGIFYHFKREIDEQLEQIHKSYSQLVHGDFKGDDLHMIALIFRLLRQRGYNVSSGMFVFNKFKNSEGNFRESLITDVRGLLSLYEACHLRCHGDSILEEALPFAITHLESINESKVSTSLAKQVKRALRQPLHKGLPRLEARYYVPLYQEEPSHDQVLLALAKLDFNLLQEQHQKELGNITRRWWKDIDVATKFPFARDRIVELFFWISGAYFEPEFAVARDIFTKVIALTSILDDMYDIYGTLEELVILTEAIERWDVDAMDGLPEYMQAWYKLLLNVYDAIGNEVATKGRSYRLTYAKEAVMKKQARVYFHEAKWFHTNYTPTLEEYMPLALLTTGYEMLAITSLVGMGDVVTKHAFEWLLGDCKILKASQIICRLMDDIASHQFEQKRGHVASSVELFMKEHNVSEQEAEKELRKRVVDAWKDINEAFLRPTVVPLPILMRTLNLSRVIHVLYSDGDNYTHSGTSLKDHVTSLFISPLPV

>EglobTPS025

PLQKLHLIDQIQRLGIEFHFEREVDEQLEQIHKSYSRLDHEDFKVDDLHTVALIFRLLRQHGYNISEVFDKFKDSKGNFRESLISDVHGLLSLYEACHLTTTHLESINESKVSTSLAKQVSHALKXPLRKGLPRLEASHYIPLYQEEPSHDEVLLTLAKLDFNLLQEQHQKELGKITKFPFARDRIVELFFWTTGIYFEPEFAAREILTKVISLTSIMDDIYDVYGTPEELALLNEAIQRWDFDAMDGLPEYMQAYFKEFLQLYEYIGNQLAAKGKSYRLIYAKEVVSIIDKLYMMKKLVGAYFQEAKWFHTNYIPTLEEYMPLQLITTGYGMLSTTSLVGMGDVVTEHVLQWSVSDCKFEQKRGHVVSAVEVLMKYRGISEQEAAEELHKGVIDAWKDTNEEFLRPTAVPMSVLTRMLNFSRVIDVLYSDGDNYTHSKTKLKDYVTSLFINP

>EglobTPS027

DQHAEQEIQKLDDEVKRMLCADADKPSLKLDMIDQIQRLGIAHRFASDIDHVLKQLSETCFACNNGDRDIDDLYTAALLFRLLRQQGYRVSDIFNKFKDPSGKFSEKHASDVRGLLSLYEASHLSVHGEDVLDQALSFSLTHLESVKEQLSPPLATQVRHALKQTIRKGVPRLEARQYISMYEAEPLHNEVLLSLAKLDFNRLQKQHQKELFDITRWWMGLDFKRKLPFARDRLVEGYFWIVGVHFEPELAVARRMMTKVIAVTSVLDDIYDVYGTYEELELFTQAIQRRWDIDCIHELPEYMQVFYKALINIYVEIEEILACTGKSYCLCYAVEAVRSMKRQARSYFAEAKWLHQQHKPTMDEYMSIALVSSGYPLLAVTSFVGMPDIVTKDDLDWLFNDPKILKASTIICRLMDDLATHKFEQSRGHVDSAVQCYMKQYGVTEQEAENNLRKQVNDSWKDINEECLRPTAVAMPLLVGILNLSRVMDVLYKDGGDHYTNPHIALKDYIHSVLIDPV

>EglobTPS028

PDLQVSAIPRSSPNIGTDHVIERRSAGYHPSIWGDYFLKYAEEQIEELKGKVRKMLASVVDKPSQMLHLIDQIQRLGFDYHFEHEVDEQLEQIHKSYSQLHLEDFKVDDLHIVALIFRLLRQQGYNVSVGCTEIFNKFKDSEGNFRESLVTDARGLLSLYEACHLRCHGDSILDEALPFATTHLESIDESKVSTSLAKQVSHALEQPLRKGLPRLEARRYIPLYQEEPSHDEVLLALAKLDFDLLQEQHQKELGEITRFRWWKEIDVPRKFPFARDRIVELFFWISGIYFEPEFAMARNILTRVISLTSILDDIYDVYGTLEELALLTEAIQKYGIRTLSQTSEFDQFCIESRWDVDAMDGLPVYMQAYYKELLHLYEYIGNELATKERSYRLVYAKEVVSIIGTLYMKKLARAYFQEAKWFHTNYIPTLEEYMSLQLITTGYGMLATTSLVGMGDVVTKHALEWSVGDCKIVKAAQTISRLMDDIASHQFEQKRGHVVSAVELLMKYHGVSEQEAGEELQKGVIDSWKDINEEFLRPTAVPMPILTRMLNFSRVMDVLYSDGDNYTHSETKLKDYVMLLFVSPL

>EglobTPS029

EHIERLKGEVRKMLTGAMDKPSQKLNLIDEIQRLGFAYHFEHEIDEQLEQIHRSYFEFHYGDNDDNLHTVAVLFRLLRQQGYNVSCEIFNRFKDSEGNFNKSIIADVQGMLSLFEACHLSYHGDDILNDALAFTISHLESIEKKKVSPNLVKQVSHALHQPIQKGLPRLEARRYIQFYQEEPLHNEVLLSLAKLDFNSLQEQHQKELGNLTRRWWKDINIEREFPFARDRLGELYVWMLGIYFEPKYEIARGIVTKMMVILSILDDIYDVYGTLEELELFTEAIERYRWDVDAKEGLPKCMQVFYKTLLDFYDEISNELARKGRSYRLFYAKEVVSMKIQVRAYLAEAKWFHHSHVPTMEEYMPIALISIGTQLTFVTAFLGMGDIVTKDAFDWLLSSDPRIVKASQVIGRLMNDIAGHKFEQERGHVASSVECFMKQYSVTEEEAKKELCKQVANAWKDINEELRRPTAVPMVLLMRIINLARATHAVYEDETDH

>EglobTPS031

FHACFLKHCVIYLQKFKFLGRVEGQIEELKGEVKKMLIDAVDKPLPKLHLIDQIQRLGIEYHFEREVDEQLEQIHKSYSRLDHEDFKVDDLHTVALIFRLLRQHGYNISRCLISDVPGLLSLYEACHLRCHGDSILDEALPFATTHLESINESKVSTSLAKQVSHALKQPLRKGLPRLEASCYIPLYQEEHSHDEVLLTLAKLDFNLLQEQHQKELGKITWWKNIDVPRKFPFARDRIVGVFFWTTGIYFEPEFAMAREILTKVISLTSIMDDIYDVYGTPEELALLNEAIQKYRWDFDAMDGLPVYQYMQAYFKEFLQLYEYIGNQLAAKGKSYRLIYAKEVVMKKLVGAYFQEAKWFHTNYIPTLEEYMPLQLITTGYGMLSTTSLIGMGDVVTEHVLKWSVGDCKSVKATQTICRLMDDVSSHEFEQKRGHVVSAVELLMKYRGISEQEAAEELHKGVIDAWKDTNEEFLRPTAAPMSVLTRMLNFSRVIDVLYSDGDNYTHSNTKLKDYVTSLFINPLPM

>EglobTPS032

IEELKGEVKKMLTDIMDKPLQKLHLIDQIQRLGIEYHFEREIDEQLEQIHKSYSRLDHEDFKVDDLHIVALIFRLMRQHGYNVSSEVFDKFKDSGGNFRESLISNVLGLLSLYEACHLRCHGDSILDEALPFATTHLESINESKVSTNLAKQVSHALKQPLRKGLPRLEARHYIPLYQEEPSHDEVLLTLAKLDFNLLQEQHQKELGKITWWKNIDVPRKFLFARDRLVELFFWTTGVYFEPEFAMARDILTKVISLTSIIDDVYDVYGTLEELALFNEAVQRWDVDAMDGLPEYMQALFKEFLQLYEYIGNELATKGRSYYLVYAKEVVSIRGKIYMKKLVSAYFQEAKWFHTNYIPTLEEYMSLQLITSGYEMLATTSLMGMGNVVTEHALKWSISDCKIGKAAQTIGRLMDDIVSHEFEQKRGHVVSAVELLIKYRGVSEQEAVEELQKRVIDAWKDTNEEFLRPTAVPMPILTRVLNLSRVVDVLYSDGDNYTHSETKLKDYVTSLFVNPLPM

>EglobTPS033

SQVSATPCAPPNKGTGHVIERRSAGYHPSVWGDYFLKYDSPSDKLRKKLHLIDQIQRLGMEYHFEREIDEQLEQIHKSYSRLDHEDFKVDDLHIVALIFRLLRQHGYNISVFDKFKDSKGNFRESLISDVRGLLSLYEACHLRCHGDSILDEALPFATTHLESINESKVSASLAKQVSHALKQPLRKGLPRLEASCYIPLYQEEPSHDEVLLTLAKLDFNLLQEQHQKELGKITRFFARDRIVELFFWTTGIYFEPEFAMAREILTKVISLTSIMDDIYDVYGTLEELALLNDAIQTYRWDFDAMDGLPEYMQAYFKEFLQLYEYIGNQLAAKGRSYRLIYAKEVVSIIDKLYMKKLVGAYFQEAKWFHTNYIPTLEEYMPLQLITTGYGMLSTTSLIGMGDVVTEHVLKWSVGDCKSVKATQTICRLMDDVSSHEFEQKRGHVVSAVELLMKYRGISEQEAAEELHKGVIDAWKDTNEEFLRPTAVPMSVLTRMLNFSRVIDGLYSDGDNYTHSKTKLKDYVTSLFLNPL

>EglobTPS036

VEEQIEKLKGNVRKVLAGVMDKPSQMLHLIDQIQRLGIDYHFEHEVDEQLEQINKSYSQLHLEDFKVDDLHMAALIFQLLRQQGYNVSVGCTEIFTKFEDSEGNFRESLVTDARGLLSLYEACHLRCHGDSILDEALLFATTHLESIDERKMSTSLLKQVSHALEQPLHKGLPRLEARHYISLYQVEPSHDEVLLTLAKLDFNLLQEQHLKELGKITWWKEIDVPRKFPFAREMIVELFFWISRIYFEPEFVMAKNILTKVISLTSILNDIYDVYSTLEELALLTEAIQKYGIRWDVDAMDGLPVYMQAYYKEFLQLYEYIGNELATKERSYYLVYAKEVVMKKLARAYFQEAKWFHTNYVPTLEEYMSLQLITIGYGMLATTSLVRMGDVVTEHALEWSIGDCKIVKVAQTIYRLMDDIASHQFEQKRGHVVSAVELLMKYHGVLEQEAREELQKGVIDAWKDINEEFLCLTAVPMPILTRMLNFSRVMDVYSNGDNYTHSETKLKYYVTLLFVSPLPM

>EglobTPS038

EHIERLKEVRKMLMGAMDKPSQKLNLIDQIQRLGFAYHFEHEINERLEQIYKSYFEFHYGDNDDNLHTVAVLFLLQQQGYNVIFNRFKDSEGNFNKSSIAEVQGMLSLFEACHLSYHGDDILNDALAFIISHLESIEKKKVSPNLVKQVSHALHQPIQKGLPRLEARRYIQFYQEEPSHNEVLLSLAKLDFNSLQEQHQKELGNLTRRWWKDIDIEREFPFARDRLGELYVWMLGIYFEPVYKIARGIVTKMMVILSILDDIYHVYGTLEELELFTEAIERYDRWDVDAKEGLPKCMHVFYKTLLDFYDEIGNELARKGRSYMKIQVRAYLVEAKWFHHSQPMMEEYMPIALISICSQLTFVTAFLGMGDIVTKDAFDWLLSSDPRIVKEVVGQLMNDIAGHKFEQERGHVASSVECFMKQYSVTNGRSVGEIFLKYFEAVKTFPSV

>EglobTPS039

SGVPFPSPAEETSPVAERRSAIFHPTIWTDYFLKYASDSTSTVEEQIKRLKGEVRKMLTGAMDKPSQKLNLIDRIQRLGLAYHFEHEIDEQLEQIHRSYFEFHCEDNNNNLHTIALLFRLLRQQGYNVSCGMCSNFLDEIFNRFKDNEGNFSKSIIADVQGLLSLYEACHLSYHGEDILNDVLTFTITHLESIDKRKSEPNLEKQVSHALHQPIQKGLPRLEARRYIQFYQEEPSHNEVLLSLAKLDFNSLQEQHRKELGNLAWWKDIDIEREFPFARDRLGELYIWNLGVHFEPEYEISRGILTKMMAILTILDDIYDVYGTIEELELFTEAIERYNRWDVDAKEGLPECMQVIYKILLNFYDEIGYELTRKGRSYRLFYAKEAVSHMKIQVRAYLAEAKWFHHSHVPTMEEYMPIALTTIGIQMALVASFLGMGDTVTKDVFDWLLFSDPKIVKALRVIGRLMNDIAGHKFEQERGHVASSVECFMKQYKVTEEEAKKELRKQVADAWKDINEELCRPTAIPRVLLMRIINLAGAIHAVYEDETDNFVNAGTNFKEFVTCLLVNP

>EglobTPS040

EIFEKFKDSDGNFRESLTADILGILSLYEACHLRVHGEDVLDEALSFTVTHLESIDKNQVSPTLAKQLSHALKQPIYKGLPRLEARQYIPIYQEEPSHNEVLLSLAKLDFNLMQEQHQKELGHIARSLNLANNLQVVEGIRCCKNFPFARDRLVECYFWILGVYFEPEFILARKFMTKVIAMTSIIDDIYDVYGTLEELKLFTEAMERWSIDAIDGLPKYMQVCYKALLDVYDDTEKAIAENGTSYGLYHAKEAVRWSIDAIDGLPKYMQVCYKALLDVYDDTEKAIAENGTSYGLYHAKEAVMVKASTTICRLMDDIVSHQMVKASTTICRLMDDIVSHQGHVASAVECFVHQHGVTEQEAKDELWRRVVEAWKDVNEECLAPTAIPSRLLTLILNLTRVIDVLYTDEDNYTNAGTKLKNYVASLLIYPLPM

>EglobTPS041

PQISAMPSPSPALETSHVAERRSGNFHPSIWGDYFLKYASDSERIERLKGEVKKMLTSAMDKLSQKLNLIDQIQRLGLAYHFEIEIEKELEQIHRSYFEHHCGDNDEDLHTTALLFRLLRQQGYDVSCGMEIFNKFKDNEGHFSKSLIADVRGLLSLFEACHVGFHSDDILNDALAFTVTHLESIDKEKVSRNLEKEVSHALSQPIHKGLSRLEARHYIQLYQEEPLHNEVLLSLARLDFNLLQKQHQKELGNITRLTRWWKDLDGERKFPFARDRLVELYFWMSGVYFEPKYEATREILTKMIVIVSIFDDMYDMYATLEEVEVFTEAIERYRWDVNAKDGLPKYMQVCYETLLDLYDEFGDKFTRKGQSYSLFYAKPIASTSIGCELLLGTSFLGMGDIVTKNDFDWLLYSDSKMVKASKVVARLMDDIAGHKEQERGHSPSSVECFMKQYRVTEEEAKEELRKQVVNAWKDMNEELRRSSAVPKLLRTRILNFAQVFDVVYNDEKDHYSHAGTKFKEHVTSLYVDPLPM

>EglobTPS042

VEEQIEELKGVVRKMLAGVVDKPSQMLHLIDQIQRLGIDYHFEHEVDEQLEQIHKSYSQLHLEDFKVDDLHMVALIFRLLRQQGYNVSEIFNKFKDNEGNFWESLVTDARGLLSLYEACHLRCHGDSILDEALPFATTHLESIDESKVSTSLAKQVNHALEQPLPKGLPRLEARRYIPLYQEEPSHDEVLLTLAKLDFNLLQEQHQKELGEITRFWKEIDVPRKFPFARDRIVELFFWTSGIYFEPEFAMARNILTKVISLTSILDDIYDVYGTLEELALLTEAIQKYRWDVDAINGLPVYMQAYYKELLQLYEYIGNELATKERSYRLVYAMKKLARAYFQEAKWFHTNYVPALEEYMSLQLITTGYGMLATTSLVGMGDVVTEHALEWSIGDCKIVKAAQTICRLMDDIVSHQFEQKRGHVVSAVELLMKYHGVSEQEAGEELQKGVIDAWKDINEEFLRPTTVPMPILTRMLNFSRVMDVLYSDGDNYTHSETKLKDYVTLLFISPL

>EglobTPS043

MSLPVSTIPSPSPAHETGQVAERRSGNFHPSIWGDYFLKYERIEKLKGEVRKMLTSAMDKPSQKLNLIDQIQRLGLAYHFEIEIDEQLEQIHRSYFEFHCGDNDSNLHTTALLFRLLRQHGYNIPCGMSFQFEIFNKFKDNEGNFSKSLIADVQGLLSLFEACHLGFHGDVILNDALAFTITLLESIDKGKVTGNLEKQVTHALNQPIHKGLPRVEARHYIQLYQEEPSHNEVLLSLAKLDFNLLQEQHQKELGNITRFMERESFRLPEIGLCFEPEYEATREILTKVMVIVSIFDDVYDIYATLEELELFTKAIERWAVDAKDGLPEYMQVCYKTLLDLYDEIGYEVTRKGQSYCLFYAKEVVMKNHMRTYLAQAKWFQQNYVPTMEEPIASLSIGCELLLGTSFLGMGDVVTKSYFDWLLLSDNKMVKASGVISRLMNDIAGHKVYKSYTIEQHSNGIYELRSKRLRTLSVNKKPLLVRILNFARVFHVVYNDEVDHYSHAGAKFKEFVTSLLVDPLPM

>EglobTPS044

KERIEKLKGEVRKMLTSAMDKPSQKLNLIDQIQRSGLAYHFEIEIDELEQIHRSYFEFHCGDNDNNLHTTALLFRLLQHGYNIPCGMSFQFEIFNKFKDNEGNFSKSLITDVQGLLSLFEACHLGFHGDVILNDALAFTITLLESIDKGKVSGNLEKQVTHALNQPIHKGLPRVEARHYIQLYQEEPSHNEVLLSLAKLDFNLLQEQHQKELGNITRFNFMIARWWKDLDGERKFPFARNRLVELYFWMSGVYFEPEYEATREILTKVMVIVSIFDDVYDIYATLEELELFTKAIERWAVDAKDGLPEYMQVCMKNHMRTYLAQAKWFQQNYVPTMEEYLPIASLSIGCELLLGTSFLGMGDVVTKSYFDWLLLSDNKMVKASGVISRLMNDIAGHKFEQERGHTASSVECFMKQYRVTEQEAKDALRKQCPQATPCANSQVPKPLLVRILNFARVFHVVYNDEVDHYSHAGAKFKEFVTSLLVDPLPM

>EglobTPS045

DQQAEQEIQKLKDEVKRMLCAHADKPSLKLDMIDQIQRLGIAYHFASEIDNVLKKLSQTYFVSNNGNYDNDDLYTVSLLFRLLRQQGCRISCDIFNKFKDTSGKFTEKHASDVRGLLSLYEASHLSVHGEDVLDQALSFSLKHLESIDKEQLSPPLAAQVQHALKQTIRRGVPRLEARRYISMYEAEPLHNKVLLSLAKLDFNHLQKQHQEELFDLAWWMGLDFKSKLPFARDRLVEGYFWILGVHFEPELAPVRRMMTKVIAMTSVLDDIYDVYGTYEELELFTRAVQRSDRWDIDCINELPEYMQVFYKALIDVYVEIGEKLACTGRSYGLDYAKEAVRMKRQARSYFAEAKWLHQQHKPTMDEYMSVALVSSGYPLLAITSFVGMQDIVTKDDLDWLFNDPKILKASTVICRLMDDLATHKFEQGREHADSAVQCYMKQYNVTEQEAENDLRKQVDDAWKDLNEESLCPTAVAKPLLMGILNLTRVMDVLYKDGGDHYTNPHIMLKDYIRSVLMDPV

>EglobTPS046

IFNKFKDTSGKFGEKHASDIRGLLSLYEASHLSVHGEDVLDQALSFSLKHLESIDKEQLSPPLAAQVQHALKQTIHRGVPRLEARQYISMYEAEPLHNKVLLSLAKLDFNHLQKQHQKELFDLAWWMSLDFKNKLPFARDRLVEGYFWILGVHFEPELALVRRMMTKVIAMTSVLDDIYDVYGTYEELELFTRAVQRSDWYRTVLSIYIRMRWDIDCINELPEYMQVFYKALIDVYVEIGEKLASAGRSYGLDYAKEAVRMKRQDRSYFAEAKWLHRQHKPTMDEYMSVALVSSGYHLLAITSFVGMQDIVTKDDLDWLFNDPKILKASTVICRLMDDLATHKFEQGREHADSAVQCYMKQYNVTEREAENDLRKQVDDAWKDLNEECLCPTAVAKPLLMGILNLTRVMDVLYKDGGDHYTNPHIMLKDYIRSVLMDPV

>EglobTPS047

DQQAEQEIQKLKDEVKWMLCAHADKPSLKPDMIDQIQRLGIAYHFASEIDNVLKKLSETYFVSNNGDHDNDDLYTVALLFRLLRQQGYRISCDIFNKFKDTSGKFGEKHASDIRGLLSLYEASHLSVHGEDVLDQALSFSLKHLESIDKEQLSPPLAAQVQHALKQTIHRGVPRLEARQYISMLHEAEPLHNKVLLSLAKLDFNHLQKQHQKELFDLAWWMGLDFKNKLPFARDRLVEGYFWILGVHFEPELALVRRMMTKVIAMTSVLDDIYDVYGTYEELELFTRAVQRSDRWDIDCINELPEYMQVFYKALIDVYVEIGEKLASAGRSYGLDYAKEAVRMKRQDRSYFAEAKWLHRQHKPTMDEYMSVALVSSGYHLLAITSFVGMQDIVTKDDLDWLFNDPKILKASTVICRLMDDLATHKFEQGREHADSAVQCYMKQYNVTEREAENDLRKQVDDAWKDLNEECLCPTAVAKPLLMGILNLTRVMDVLYKDGGDHYTNPHIMLKDYIRSVLMDPV

>EglobTPS048

VSATPCAPPNKGTGHVIERRSAGYHPSVWGDYFLKYQIEELKGEVKKMLIDVVEKPLQKLHLIDQIQRLGIEYHFEREVDEQLEQIHKSYSRLDHEDFKVDDLHTVALIFRLLRQHGYNISEVFDKFKDSKGNFRESLTSDVHGLLSLYEACHLRCHGDSILDKALPFATTHLESSNKSKVSTSLAKQVSHALKQPLRKGLPRLEASRYIPLYQEEPSHDEVLLTLAKLDFNLLQEQHQKELGKITRFSNRWWKNIDVPRKFPFARDRIVELFFWTTGIYFEPEFAMARELLTKVISLTSIMDDIYDVYGTVEELALLNAAIQKYGIRWDVDAMDGLPEYMQTYFKEFLQLYEYIGNQLAAKGRLYRLIYAKEVVSIIDKLYMKKLVRAYFQEAKWFHTNYIPTLEEYMPLQLITTDYGMLATTSLVGMGDVVTEHVLKWSVSDCKSVKATQTICRLMDDVSSHEFEQKRGHVVSAVELLMKYHGVSEQEAGEELLKGVIDAWKDTNEEFLRPTAVPMSVLTRMLNFSRVIDVLYSDGDNYTHSKTKLKDYVTLLFVNPLP

>EglobTPS049

SHGIAEERIERLKGEVGKMLTSAMYKPAEKLNLIDQIQRLGIAYHFELEIDKELEQIHRGYFEFHCDDNDNDLDTVALLFQLLRQRGYHVFHAPFGHTEIFNKFKDGDGNFGKSLIADVQGLLSLFEACHLRYHGDNLEDALAFTTTHLESIDKRKASLHLVKKVSHALNQPIHKGMSRLEARRYIPLYQEEPSHNEVLLSLAKLDFNLVQEQHRKELGNLTRRWWKGLDVQKKFPFARDRLVEMYVWWLGEYYEPEHEAAREILTKLISVTSIIDDIYDVYGTWEELELFTEAIERYNRWDVNAKDGLPEYMQECYKIVLDLYDEIGYEFDFCGTFPKYQVSRMKNQARAYLVEAKCFHQNHVPTMEEYMSIALPSAGIVSILAWSFLGMGDIVTKDVFDWLLFNDPKMVKAFTEEEAKEELRKQVTNAWKDINEELRGPT

>EglobTPS050

EQERGHSPSSVECFMKQYRVTEEEAKEELRKQVVNAWKDMNEELRRSSAVPKLLRTRILNFAQVFDVVYNDEKDHYSHAGTKFKEHVTSLYVERIERLKGEVKKMLTSAMDKLLQKLNLIDQIQRLGLAYHFEIEIDKELEQIHRSYFEFHSGDNDDDLHMTALLFRLLRQQGYDVSCGMIFNKFKDNEGHFSKSLIADVRGLLSLFEACHVGFHGDDILNDALAFTVTHLESIDKGKVSRNLEKEVSHALSQPIHKGLSRLEARHYIQLYQEEPLHNEVLLSLARLDFNLLQKQHQKELGNITRRWWRDLDGERKFPFARDRLVELYFWMSGVYFEPKYEATREILTKMIVIVSIFDDMYDVYATLEEIEVFTEAIERYSWHSRWDVDAKDGLPKYMQVCYETLLDLYDEFGNKFTRKGQSYCLFYAKEVVSMKNHLKAYFAEAKWFHQNHMPTMEEYMPIASTSIGCELLLGTSFLGMGDIVTKNDFDWLLYSDSKMVKASKVVARLMDDIAGHKFEQERGHSPSSVECFMKQYRVTEEEAKEELRKQVVNAWKDMNEELRRSSAVPKLLRTRILNFAQVFDVVYNDEKDHYSHAGTKFKEHVTSLYVD

>EglobTPS051

EEQIEGLKGEVRKMLTDAVNKPSQVLHLIDQIERLGIFYHFKREIDEQLEQIHKSYSQLVHGDFKGDDLHMIALIFRLLRQHNVSSGMFVFNKFKNSEGNFRESLITDVRGLLSLYEACHLRCSVFESLLQEKKKEFKLYESVKQKYFRFHSYLFNHRWWKDIDVATKFPFARDRIVELFFWISGAYFEPEFVEARDILTKVIALTSILDDMYDVYGTLEELVILTEAIERWDVDAMDGLPEYMQAWYKVLLDVYDVGNEVATKERSYHLTYAKEAVMKKQARVYFHEAKWFHTNYTPTLEEYMPLALLTTGYEMLGMGDVVTKHAFEWLLGDKILKASQIICRLMDDIFSHQFEQKRGHVASSVELFMKEHDVSEQETEKELRKRVVDAWKDINEAFLRPTAVPVPILFSIILNLSQVIHVLYSDGDNYTHSGTLLKDPVSHL

>EglobTPS058

DQHAEQEIQKLDDEVKRMLCADADKPSLKLDMIDQIQRLGIAHRFASDIDHVLKQLSETCFVCNNGDRDIDDLYTAALLFRLLRQQGCRVSNIFNKFKDPSGKFSEKHASDVRGLLSLYEASHLSVHGEDVLDQALSFSLTHLRLEARRYISMYEAEPLHNEVLLSLAKLDFNRLQKQHQKELDITRRWWMGLDFKRKLPFARDRLVEGYFWILGVHFEPELAVARRMMTKVIAVTSVLDDIYDVYGTYEELELFTQAIQRRWDIDCIHELPEYMQVFYKALINIYVEIEEILACTGKSYCLCYAVEAVRSMKRQARYYFAEAKWLHQQHKPTMDEYMSIALVSSGYPLLAVTSFVGMPDIVTKEDLDWLFNDPKILKASTIICRLMDDLATHKFEQSRGHVDSAVQCYMKLYGVTEQEAENNLRKQVNDSWKDINEECLRPTAVAMPARVMDVLYKDGGDHYTNPHIALKDYIHSVLIDPV

>EglobTPS059

DALTFTITHLESIDKRKVSPNLEKQVSHALNHPIRKGLPRLEARHYIQFYQEEPSHNEVLLSLAKLDFNSLQGQHQKELGNLTRFRWWKDLDIKREFPFTRDRLAELYVWMLGVHFEPDYEIARGIVTKMMVIISILDDIYDVYGTLEELEIFTEAIERYRWDVDAIEGLPECMQVIYKIIFELYDEIGYELTRKGRSYRLFYAKEAVSHMKIQVRAYLVEAKWFHKSHIPTMEEYMPIASTTIGNQMAFVASFLGMRDIVTKDTFDLLLSSNHKIVKASKVIGRLMNDIAGHKFEQERGHVASSVECFMKQYKVTEEEAKKEIRKLVADAWKDINEELRHPTAVPMVVLMRIVNLAGAIHAVYEDETDHYVNAGTNFKEFVTCLLVNP

>EglobTPS060

ERIERLEGEVKKMWIGAMDKPSQNLIDHIQRLEFAYHFEHEIDEQLEQNHRSYFEFHYDDNNGNLHTIANRFKDSKGNFSKCITDVQGMLSLFEACHLSYHGNDILNGALAFTITHLESIKKKKVTPNLKKQVSHALHQPIQKGLPRLEARLYIQFYQVEPLHNEVLLSLAKLDFNSLQEQRQKELGNLTRRWWKDIDMEMEFPFARDRLGEVHVWMLGIDFEPVYEITRGMVTKMMVILYILDDIYDVYGILEELELFTKAIERRWDVDAKEVLPKCMQVFYKTLLDFYDELGNELTRKERSYRIMKIQVSMYLAEAKWCHQNHIPTMEEYIPVGLISNGSQLVFVTAFLVMGDIVTKDTFDWLLSGDPKIVKASQVIGWLMNDIAGHKFEDRGHVASLMEGFTEYRVMEEVAKKELCKKSGQCWTDIKEELPRLIAVPMVLLMRIINLARAMHVVYEDETNHYVNAGT

>EglobTPS062

MALPVLCPTFLPSTICHNQPSLLSFRHLRFAQFVTCASKIEDQEIVRRSANWQPSVWDYDFVQSLSVDYTEDKHMEQVQRLKEEVRGLFDREINQVAKLEFIDVVQRLGLGYHFEMEIKNALSSIYNNTEDAQISDNLYATSLRFRLLRQHGYNVPQGACQDVFQRFMSKMGTFNELLHEDVKGLLGLYEASFHGLEGETILDEGWNFASKHLNDLNLDKVPTNIASHVSHALDMPIHWRPNRLEARWFMDMYGKQQDMIPSLLRLAKIDFNLVQSIHRKEVSNLARYRWWVELGANKMTFSRDRLVENYFWSCIFVFEPQYTAFRELSTRIGCMVSLIDDIYDIYGTPEELELLTDFDYFSRWDITNIDKLPPTIRDGFMVLYNTTNELGYWTMRERGINPIPYLRKLVISTCWADECKAYMKEVYWYNKGIKPTLKEYMDVGVDSIGGLILLLDSYFLTTDKLTEEGLDYVSKIPGVMHSSAKILRFNDDLSTSSHELARGDNSKALECYMNETGASEEAAREHIKHLVRETWKKMNKEVFEDYPFSGFGPFLSACLNLARASHCFYDYGDGHGLPGHQTKDHVVSTIFESVPLD

>EglobTPS063

MALPALFTSFVPSSISHNQPSLLSFRHPRVTCAVTIENPEIVRRSANWKPTVWDYEFLQSLRVDYTEDKYAEQVQRLKEEIRGLFNREMNQVAKLEFIDTVQRLGLGYHFEMETKNALSSIYDNTGYAQLLNDLHAVSLGFRLLRQHGYKIPQGIDVFQQFMNKTGTFNESLNKDVRGLLSLYEASFHGLEGETILDEARNFASKHLKDLNLDKVPTMLASYVRHALDMPIHWRPNRLEARWFMDMYEKQQDMIPSLLRLAKLDFNLVQSVHKKEVSNMARYRWWVELGANKMTFFRDRLVEHYFWCCAMVFEPQYTAYREMTTKLTCMVTLIDDVYDVYGTQEELELLTDFLVRWDITEIDKLPPIIRDSYMALYNTTNEIGYWTMRELGINTIPYMQKVWADECKAYIKEVHWYNKGIKPTLKEYMDNAVDSIGGLIMLLGSYFLTTDQSLITCRKFRVSCIALPRSFDSTMISVPHRYELARGDNFKALECYMNETGASEEATREHVKKMVHETWKRMNKDVFEDYPYSGFGPFLGACLNLARASQCFYQYGDGHSLPDNETKDHLVRALFDPVPLD

>EglobTPS064

MVLPALFTSFVPSPISHNQPSLLSFRHPRCSSSSFSSGAKSVTCAMTIENPEIARRSANWKPNVWDYEFLSLIVDYTEDKYAEQVQRLKEEIRGLFNREMNQVAKLEFIDAVQRLGLGYHFETETKKNALSSIYDNAGYAQLLNDLHAVSLGFRLLRQHGYKISQGIDVFQQFMNKTGTFNESLNKDVRGLLSLYEASFHGLESETILDEVRNFASKHLKDLNLDKVPAMLASYVRHALDMPIHWRPNRLEARWFMDMYEKQQDMIPSLLRLAKLDFNLVQSVHKKEVSNMARWWVELAANKMTFFRDRLVEHYFWTCAMVFEPQYTAYREMTTKLTCMVTLIDGVYDVYGTQEELELLTDFLVDILSRWGIIEINKLPPTIRDSMALYNTINEIGYWTMRELGINTIPYMQKVDECKEYIKEVHWYNKGIKPTLKEYMDNAVDSIEGLIMLLGSYFLTTDKLTEEGLGYVSKIPSVMHCSAKILRLNNDLSTSSSHELARGDNFKALECYMKETGASEEATHEYVRQMVHETWKRMYPYSGFGPFLGACLNLARASQCFYQYGDGHGLPDNETKDHLVRALFDPVPLD

>EglobTPS065

MALPVLCPSILPSTIFHNQPSLLSFRHLHSSFSCSANWEPSVWDYGVVQSLSVDYEDKYMEQVQRLKEEVRGLFDREINQVAKLEFIDVVQRLGLGYHFETEIKNALSSIYNNTEDAQVSDNLYVASLRFLLRQHGYNIPQGTCQDVFQRFMSKMGTFNESLHEDVKGLLGLYEASFHGLEGETILDEGWKFASKHLKDLNLNEVPTNIASNVSHALDMPIHWRPNRLEARWFMDMYGKQQDTIHSLLQLAKIDFNLVQSIHRKEVSNLARYRWWVELGANKMTFFRDRLVENYFWSCIFVFEPQYTAFRELNTRIGCLDTTLIDDVYDIYGTPEELELLTDFILRWADECKAYMKEVYWYNKGIKPTLKEYMDVAVDSIGGLILMLDSYFLTTDEVTEEGLDYEIPGVMHSSARILRFNDDLSTSSHELARGDNSKALECYMNETGASEEAAREHIKHLVRETWKKMNKEVFEDYPFSGFKPFLGSCMNLARASHCFYDYGDGHGLPGHQTKDHLVSTIFESVPLD

>EglobTPS066

MALPALFTSFVPYSISHNQPSLLSFRHPRCSSSSFSSGAKSVTCAVTIENPEIVRRSANWKPNVWDYEFLQSLRVDYTEDKYAEQVPRLKEEIGGLFNREMNQVAKLEFIDAVQRLGLGYHFETEIKNALSSIYDNAGYAQLLNDLHAVSLGFRLLRQHGYKISQGIDVFQQFMNKTGTFNKSLNKDTRGFLGLYEASFHGLEGETILDEARNFASKHLKDLNLDKVPAMLASYVSHALDMPIHWRPNRLEARWFMDMYEKQQDMILSLLRLAKLDFNLVQSVHKKEVSNMARYRWWVELGANKMTFFRDRLVEHYFWTCAMVFEPQYTAYREMTTKLTCMVTLIDDVYDVYGTQEELELLTDFLVRDILSRWDITEIDKLPPTIRDSYMALYNTTNEIGYWTMRELGINTIPYMQKVWADECKAYIKEVHWYNKGIKPTLKEYMDNAVDSIGGLIMLLGSYFLTTDKLTEDGLDYVSKIPSVMHCSAKILRLNNDLSTSSYELARGDNFKALECYMNETGASEEAAREHVRQMVHETWKRMNKDVFEDYPYSGFGPFLGACLNLARASQCFYQYGDGHGLPDNETKDHLVRALFDPVPLD

>EglobTPS068

MALPALFTSLVPSSISHNQPSLLSFRHPRCSSSSFSSGAKLVTCAVTIENPKIVRRLANWKPNVWDYEFLQSFGVDYEDKYAEQVQRLKEEIRGLFNREMNQVAKLEFIDAVQRLGLGYCFETEIKNALSSIYDNTGYAQLLNDLHVVSLGFRLLRQHGYKISQGIDVFQQFMNKTGTFNESLNKDVRGLLGLYEASFHGLEGETILDEARNFASKHLKDLNLDKVDVFQQFMNKTGTFNESLNKDVRGLLGLYEASFHGLEGETILDEARNFASKHLKDLNLDKVVSNMARYRWWVELGTNKMTFFRDRLVEHYFWCCAMVVEPQYTAYREMTTKLTCMVTLIDDVYDVYGTQEELELLTNFLVRDITEIDKLPPIIRDSYMALYNMTNEIGYWTMRELGINTIPYLQKVNKGIKPTLKEYMDNAVDSIGGLIMLLGSYFLTTDKLTEEGLDYVSKIPSVMHCSAKILRLNNDLSTSYELAGDNFKALECYMNETGASKEAMREHVRQMVHKTWKRMNKDVFEDYPPSGFGPFLGACLNLAQASQCFYQYEDGHSLPDNETKDHLVRALFDPVPLD

>EglobTPS069

ASSGAQFVTCASKIEDQEIARRSANWEPSVWDYGVVQSLSVDYTEDKYMEQVQRLKEEVRGLFDKLEFIDVVQRLGLGYHFETEIKNALSSIYNNTEDAQVSSNLYAASLRFRLLRQHGYNIPQDVFQRFMSKMGTFNESLHEDVKGLLGLYEASFHGLEGETILDEGWKFASKHLKDLNLNEVPTNIASNVSHALDMPIHWRPNRLEARWFMDMYGKQQDMIPSLLRLAKIDFNLVQSIHRKEVSNLARYRWWVELGANKMTFFRDRLVENYFWSCIFVFEPQYTAFRELNTRIGCLVTLIDDVYDIYGTPEELELLTDFILRWDITNIDKLPPTIRDSFMVLYNTTNEVGYWTMRERGINPIPYLRKVWADECKAYMKEVYWYNKGIKPTLKEYMDVAVDSIGGLILMLDSYFLTTDEVTEEGLDYVSKIPGVMHSSARILRFNDDLSTSSHELARGDNSKALECYMNETGASEEAAREHIKHLVRETWKKMNKEVFEDYPFSGFKPFLGSCMNMARASHCFYDYGDGHGLPGHQTKDHLVSTIFESVPLD

>EglobTPS071

MALPALSTSFLPSSIHHNQPSLLFFRHLCSSSSASTSSTSFGAQFVTCTLKIEAQEIGRRSANWQPNVFDYDFLQSLNVDYTEDKYSEEAQRLKKEVKGLFNKDMNLVAKLEFIDLVQRLGLGYQFEMEIKNALSSIYNNAEDAQLLDDLYAISLRFRLLRQHGFNILQDVFQRFMSKSGTFNESLNEDVKGLLGLYEASFHVLEGETILDEAWTFASKHLKDLNLNKIPTNLATHVDHALEMPIHWRPNRLEARWFIDMCEKQQDMIPSLLRLAKLDFNSVQSIYRKEVSTLARYRWWVELGANKMTFCRDRLMENYFWSMIMVFEPQHTAFREMNGKIASMVTLIDDVYDVYGTPEELELLTDFIVRFWDITDIDRLPPIIRDSFMALYNTTNEIGYWTMRERGINAIPHLQKWAEECKAYLKEVHWCSKGIKPTLKEYMDVATYSTGGLVMLLASYFLTTDKLTEEGLNYVSEIPSIMHSSCEMLRLINDFSTSSYELARGDNLKALECYMNETGASEEAAREHIMHKVREGWKLMNRAMFEDYPIPGLRPFLGACLNQARVSHTFYRYGDGFGRPDNDTKDYLASAIYKPVPLD

>EglobTPS073

MALPALFTSFVPSSISHNQPSLLSFRHPRCXSSSFSSGAKSCAVTIENPEIVRRSANWKPNVWDYEFLQSLRVDYTEDKYAEQVQRLKEEIKGLFNREMNQVAKLEFIDVVQRLGLGYHFEKEIKNALSSIYDNTGYAQLLNDLYAISLGFRLLRQHGYNIRQGIDVFQQFMNKTGTFNESLNKDVKGLLGLYEASFHGLEGETMLDEARNFASKHLKDLNLDKVPTMLASYVSHTLDIPIHWRPNKLEARWFMDMYEKHQDMIPSLLRLAKLDFNLVQSVHKKEVSNMARYRWWVELGANKMTFFRDRLVEHYFWNCTMVFEPQYTAYREIVYDVYGTLEELELLTDFLVRWDITEIDKLPPTIRDSYMALYNTTNEIGYWTMRELGINTIPYMRKVWADECKAYIKEAHWYNKGMKPTLKEYMDNAVDSIGGSIMLLGSYFLTTDKLTEEGLDYVSKIPSVMHCSAKILRLNNDLSTSSYELARGDNFKALECYMNETGASEEAAREHVKQMVHETWKRMNKDVFEDYPARAF

>EglobTPS075

FTSFVPSSIRHNQPSLLSFRHPRCSFSNPEIVRRSANWKPNVWDYEFLQSLRVDYTEDKYAGQVQRLKEEIRGLFNREMNQVAKLEFIDAVQRLGLGYHFETEIKNALSSIYDNTRYAQLLNDLHAVSLGFRLLRQHGYKIPQDVFQQFMNKTGTFNKSLNKDVRGLLGLYEASFHGLEGETILDEARSFASKHLKDLNLDKVPAMLASYVSHALDIPLHWRPNRLEARWFMDMYEKQQDMIPSLLRLAKLDFNLVQSVHKKEVSNMARYRWWVELGANKMTFFRDRLVEHYFWNCTMVFEPQYTAYREMTTKLACMVTLMHSMKPTLKEYMDNAVDSIGGLIMLLGSYFLTTDQLTGEGKALECYLTEIGASEEAAREHVKQMVHKTWKRMNKDVFEDYPYSGLGPFLDACLNFARASQCFYQYGDGHGLPDKETKDHLVRALFDPVPLD

>EglobTPS076

SIHHNQPSLLFFRHLCSSSSSGAQFLTCTLKIEAQEIGRRSANWQPSVWDYDFVQSLGVDYDKYSEEAQRLKKEVKGLFDKDMNLVAKLEFIDVVQRLGLGYQFETEIKNALSSIYNNTEVAQLSDDLDAVSLRFRLLRQHGFNVSQDVFQRFMSKSGTFNESLNEHVKGLLGLYEASFHVLEGETILDEAWTFASKHLKDLNLDEIPTNLVSHVDHALEMPIHWRPNRLEARWFIDMCEKQQDMIPSLLQLAKLDFNLVQSIYRKEVSSLARYTINLAARWWVDLGANKMTFCRDRLVENYFWSSIMVFEPQHTAFREMNGKIASMVTLIDDVYDVYGTLEELELLTDFIVRFWDITDIDRLPPTIRDSFMALYNTTNEIGYWTMRERGINPIPHLQKVVISTCWANECKAYLKEVQWCSKGIKPTLEEYMDVATYSAGGLVMLLASYFLTTDELTEEGLNYVSKIPSIMHCSSKMLRLINDFSTSSYELARGDNLKALECYMNETGTSEEAAREHIMHMVREAWKWMNRAVFEDYPIPGLRPFLGACLNLARVSHTFYRYGDGFGLPNNDTKDYLVSAIYKHVPLD

>EglobTPS077

EERYMERVENMKEEVKDFICSEMPQVEKLEHIDAVQRLGLGYHFEVEIKKALQTIINGKTNRSGAFDDDLPATALLFRLLRQNGFNVEQGIFERFMTEDVSNFKESLREDVQGLLSLYEASFCGFKGEAIIDEAKIFSSTCLENLKGDHIWAKKIDRALDMPVHWRPNRLEARWFMDMYEEDQCDRSNPILLDLAKLDFNIVQSVYRDEVSKLARRWWVNLGLNKMDFCRDRLMEHYLWNALMVYEPQFGAFREMSTKITCMITLMDDVYDVFGSWEELQLLTKFIWDISQIDKLPLTIRTCFLAMYNTTNEVGYWTMKEQGFNIIPYLHKLWVNQGKTWLEEAKWYHEGHKPTLKEYLNASVTSIGGHLVLLCSYFTTSDKLSKEILEYLCNIPNVMYCSSLILRLTNDLSTSSDELVRGDNFKSLHCYMNETGASEEATRQHIKSLVQDAWKQMNEDAFCYNPCPGPFRGACLNLARASQLFYQYGDGHGIPDRETKDN

>EglobTPS078

RPSVLLFKFPRPTPSYSIADVATTAGIWCISCIDPNTNEQSPVARRSANYMPSVWDYDILKSPSADFAEERCTEPVQRMKEEVKDTLERENHLLAKLELIDAIQRLGLQYHFENDIKRALQVIRDDSNDACFSNDLHSTALRFRLLRQHGYDLSQGIDAFQRFINKTGTFEESLKKDVKGLLGLYEASFHGLEGENILDEAQDFASKHLKNLNLNEIPTCLAKQVLHALDMPIRWRPNRLEARWFMDMYGKQQDMIPSLLRLGKLDFNLVQTIHRKEVSNLARYRWWVELGANKMTFSRDRLVENYFWSCLMVFEPQYTAYREMTTKIGCMVTLIDDVYDVYGTLEELVLLTDFIVRFWDITDIDNLPPTIRDSFMALYNTTNEIGHWTMREQGINPIPYMRKWADECRAYIKEVHWYNEGIKPTLKEYMSNAVDSIGGLIMLLHSYFLTTDNLTKEGLDYVSKIPRIMHCSAKILRLNDDLGTSSYELARGDNFKALECYMNETGASTEAAQQHIKHLVRETWKTMNKDACEDYPFPGFKPFLGACLNLARASQCFYQYGDGHGLPGHETRDHIVSTLFKPVPLD

>EglobTPS081

MALPALLTNFLPSSIRPNQHSFLLVSRPCSSFSSPSAISSGARFAKCSLTIEDQDTARRSANWKPSVWDYGSVQSLNTDFEDKYTEQVQRLKEEVKGLFHREINQVAKLEFIDVVQRLGLGYHFETDIKNALSSIYNNTEDAQLSDDLYAVSLRFRLLRQHGYNLQQDVFQRFMNKMGTFNESLKEDVRGLLSLYEASFHGLEGETIVDEAWNFASKHLKDLNLDDVPANLASNVSHALDMPIHWRPNRLEARWFMDTYEKQQDKIPCLLRFAKVDFNIVQSIHKKEVSNMARYRWWVELGANKMTFFRDRLVEHYFWCCAMVFEPQYTEFREMTTKLTCMVTLIDDVYDVYGTLEELEVLTDFIVRFWDITDVDKLPLKIRACFLALYNTTNEIGYWMMRERGINPIPHMRKVVISTCWADECKAYIKEARWYNKGIKPTLDEYVDNGVTSIGGLIMLLGSYFLTTDKPTEEGLDYVSNIPSVMHCSAKILRLNNDYELARGDNFKALECHMNETGASEEATREHIKHLVRKTWKRMNRDVFEDYPYSGFGPFLGACLNLARASQCFYQYGDGHGLPDHETKAHIVSSLFDPVPLD

>EglobTPS082

MALPAVSTSFPPSSIHHNQPSLLFFRNLRSSSSVATSSNSSGAQFVTCASKIEVQEIGRRPANWQPSVWDYDFVQSLSVDYTEDKYSEEVQRLKKEVKGLFDKEMNQVAKLEFIDMVQRLGLGYQFKMEIKNALSSIYTEDAQFSDDLEAVSLRFRLLKQHGYNIPQDVFQRFMSKTDTFNESLNEDVKGLLGLYEASFHGLEGETILDEAWTFASKHLKDLNLDEIPTNLVSHVSHALDMPIHWRPNRWFINMYEKQQDMIPSLLRLAKLDFNLVQSIYRMEVSHLARYRWWVELGANKMTFCRDRLVESYFWSNTMVFEPQHTAVREMNGKIASMVVLIDDVYDIYGTPEELELLTDFIVRFWDITNIDSLPPTIRDSFMALYNTTNEIGYWIMRERGINPIPYLQKVWANECKAFLKEVHWCSNGIKPTLKEYMDVATYSAGGLVLLLASYFLTTDKLTKEGLDYVSKIPSIMHCSSKMLRLINDFSTSSYEVARGDNLNALECYMNETGASEEAAREHITHMVREAWKWMNRAVFEDYPIPGIGPFLGACLNLARVCHTFYRYGDGFGHPSNDTKDYLVSAIYEPVPLD

>EglobTPS083

SNKYDSILQKEGIKERMEGLVEEVKPMLSEAVDSLAKLELIDCMTKLGLSNLFENEMKEALETVASIHNGVFTMEEHLYASALRFRLLRQHGHIVSQSEFNRSNGEDVKTMIELLEASHLALEGENILHEAKAFSTGILRERVSSLDGRLFKRTVHALELPLHWRVQWFDIKWQISLYEQREDKQSNLLELAKLNFNTVQATHQRDLREISRRWWRDLGLMEHVDFTRDRLVESFLCALGLSQETRLSSLRKSLTKVVILILVIDDVYDLYGSLEELECFTSAIWDSEQIQQLPECMKVCFRALNDVIHEIAYDIGKDEDWHRVLPHLAKAWADFCKALLTEAKWDNMGYTPSTIEEYLSNAWTSSSGPLIMSHASFFVGHMNLEDVADLLERNKDLIYNVSMIIRLCNDLGTSAERDRGDAPSSVVCYMQEANVPEDVARKHIKELINQAWKSINAHCFGNVETPFVRTFIDVTVNASRVAHMLYQFGDGFGVQDGDIRRQILSAVIHPIALN

>EglobTPS084

MEYGTQQAQFKPCQANLPEVVGTMNQRRTANYKPNIWNYDFLQSLSSKHDSNKYDSILQKEGIKERIEGLVEEVKRILSEVVDSLAKLELIDSMTKLGLSNLFENEMKEALETVASINNDIFNMEDHLYANALWFRLLRQHGHIISQSEVELLEASHLALEGENILHEAKTFSTGILRKRVSSLDGRLFKRAVHALELPMHWRVQWFDIKWQISLYEQREDKQSNLLELAKLNFNTVQATHQRDLREISRRWWRDLGLMEHVDFTRDRLVESFLCALGLSQEPRLSSHGSARKSLTKVVILILVIDDVYDLYGSLEELECFTSAISRRDSEQIQQLPECMKVCFRALNDVIHEIAYDIGKDEDWHRVLPHLAKAWEDFCKALLTEAKWDNMGYTPSLEEYLSNAWTSSSGPLIMSHASFFVGHMNWEDVADLLERNKDLIYNVSMIIRLCNDLGTSAERDRGDAPSSVVCYMREANVPEDIARKHIKELINQEWKSINAYCFSNADTPFVRTFIDVTANAARVAHMLYQFGDGFGVQDGDIRRQILSAVIHPLALN

>EglobTPS086

EVVGTMHQRRSANYKPNIWNYDFLQSLSSKHEGIKERMEGLVEEVKPMLSKSVDSLAKLELIDSMTKLGLSNLFENEMKEALERVASNNNGVFTMEEHLYASALRFRLLRQHGHIVSQSEFLFNRSNCEDVEAMIELLEASHLASEGENILHEAKAFSTGILRERVPSLDGRLFKCTVHALEIPLHWRVQWFDIKWQISLYEQREDKQSNLLELAKLNFNTVQATHQRDLREISRRWWRDLGLMEHVEFTRDRLVESFLCALGLSQETRLSSLRKSLTKVVILILVIDDVYDLYGSLEELECFTSAIWDSEQIQQLPECMKVCFRALNDVIHEIAYDIGKDEDWHLVLPHLAKAWADFCKALLTEAKWDNMGYTPSLEEYLSNAWTSSSGPLIMSHASFFVGHMNLEDVADLLERNKDLIYNVSMIIRLCNDLGTAERDRGDAPSSVVCYMREANVPEDVARKHIKELINQAWKSINAHCFGNVETPFVRTFIDVTVNASRVAHMLYQFGDGFGVQDGDIRRQILSAVIHPVALN

>EglobTPS087

SNKYDSILQKEGIKERMEGLMEEVKRMLSEVVDSLAKLELIDRMTKLGLSNLFENEMKEALETVASINNGAFTMEEHLYANALRFRLLRQHGHIISQSEFNRSYCEDVEAMIELLEASHLALEGENILHEAKTFSTGILHDRVSSLDGRLFKRAVHALELPSDWRVQFDIKWQISLYEQREDKQSNLLELAKLNFNTVQATHQRDRWWRDLGLMEHVEFTRDRLVESFLCALGLSQEPRLSSLRKSLTKVVILILVIDDVYDLYGSLEELECFTSAITRLSTRDSEQIQQLPECMKVCFRALNDVIHEIAYDIGKDEDWHRVLPHLAKWADFCKALLTEAKWDNMGYTPSLEEYLSNAWTSSSGPLIMSHASFFVGHMNLEDVADLLERNKDLIYNVSMIIRLCNDLGTSTAERDRGDAPSSVVCYMREANVPEDIARKHIKELINQEWKSINAYCFSNAETPFVRTFIDVTVNAARVAHMLYQFGDGFGVQDGDIRRQILSAVIHPLALN

>EglobTPS088

MEYATEQAQFQPCQANLPEVVGTMHQRRSANYKPNIWNYDFLQSLSSKHDSNKYDSILQKQGIKERMEGLVEEVKPMLSEEVNSLAKLELIDRMRKLGLYNLFNDEMKEALEPVAFNRSYCEDVEAMIELLEASHLALEGENILHEAKAFSTGILRERVCPRFGASVALEGSVVRHQMANKLGEDKQSNLLELAKLNFNARESVHALELPLHWRVQWFDIKWQISLGRTSKAICLSLPNISTTHQRDLREISRRWWRDLGLMEHVDFTRDRLVESFEPRLSSLRKSLTKVVILILVIDDVYDLYGSLEELECFTSAITRLSTWADFCKALLTEAKWDNMGYTPSLEEYLSNAWTSSSGPLIMSHASFFVGHMNLEDVADLLERNKDLIYNVSMIIRLCNDLGTSTAERDRGDAPSSVVCYMREANVPEDIARKHIKELINQEWKSINAYCFSNAETPFVRTFIDVTANAARVAHMLYQFGDGFGVQDGDIRRQILSSVIHPLALN

>EglobTPS089

CLAHSPQVGSKTHQRRSANYKPSIWKYEFLQSLNDKYDELRSFKEGSNGLNGSNCQDVEAIVELLEASHLALEDENILNEAKTFSTGILHERVPGLDGLPFKRAAHYLELPMHRRVQWFDIKWQIDLYEQQEDKQSNLLELAKLNFNTVQATHQRDLIEISRWWRDLGLLEHVDFTRDRLVESFLCALGLSQEPRFSSLRKSLTKVIILILVIDDVYDLYGSLKELECFTSAITWDSEQIQQLPDCMKICFQVLHDVTYENAYDIGKDEDWHRVLPNLTKAWADFCKALLTEAKWDNIGHIPSLEEYLSNAWTSSSGPLILSHAYYFVGHMKLEDVEELLERNKYLIYNVSMIIRLCNDLGTAEKDRGDAPSSVVCYMREANVSEDVARKHIKELINQAWKSINAHCFGNAELPFLQPFIDVAMNAARVAHMLYQFGDGLGIQDGDIRRQILSTVIQPLALD

>EglobTPS090

MHQRRSANYRPNLWKYDFLQSLNNSYDLVRFEQVERLVEEVKPTLSEAVNSLLKLELIDKMKKLGLSNLFGNEIKEVLQTVASTNNGVFNMEDHLYASALQFRLLRQHGHVVSQGKFDVEAMIELLEASYLAMEGENILEEAKAFSTGILQERVSGLDGQLLKRAVHALELPMHWRVQWFDIKWQIDLYEQQEDKQSSLLQLAKLNFNIVQATHQRDLRDISRRWWRDLGLIEHVDFARDRLVESFFCALGLSQEPQFSSFRKSLTKVIILILVIDDLYDLYGSLEELECFTDAIWDLEQIQQLPECIKVCFQALRDVTYEIAHEIGKDEDWHQVVPHLMKAVSWADFCKALLNEAKWDKMGYTPSLEEYLSNAWTSSSGPLILSHAYYFMGQMKLEDAADMLKRNKDLIYNVSIIIRLCNDLGTSTAERERGDAPSSVVCYMREANVS

>EglobTPS091

QRRSANYKPNIWKYDFLRSLNNNFVHALELPMHWRVQWFDIKWQIDLYEQQEDKQSNLLELAKLNFNTVQATHQRDLIEISRRWWRDLGLIEPVQLTVYDLYGSLEKLECFTSAVTRLALSPPELFLEISKEEDWHRVLPCLTKAYLSNAWTSSSGPXLLSHAYFFVGAEIERGDAPSSMVCYMREANVSEDVARKHIKGLIDQAWKNINAHCFVNAETPFLRPYIDVTVNAARAAHMIYQSGDGFGVQDGTIGQQMLSAVIEPLSLD

>EglobTPS092

MALPALSTSFLPSSIHHNQPSLLFFRHLRSSSPAATSSTAFGAQFVTRASRIEVQEIGRHSANWQPSVWDYDYLQSLSVNYTEDKCSEEVQRLKKEVKVLLDGEMNQVAKLKLIDAVQRLGLGYQFEMEIRNALSSIYNNTEDAQLSENLDVVSLRFRLLRQHGYNIPQDVFQRFMSKTGTFNESLNEDVKGLLGLYEASFHGLKGETIIDEAWTFASKHLKDLNLNEIPTNLASHVSHALDMPIHWRLNRLEARWFIDMYKKQQDMIPSLLRLAKLDFNLVQSVYRKEVSNLARYTINLRWWVELGANKMTFCRDRMVESYFWSNSMVFEPQHTAFREMNGKIASMVVLIDDVYDIYGTLEELELLTDFIVRFRWDITDIDRLPPIIRDSFMAMYNMTNEIGYWTMRERGINPIPYLRKVWADQCKAYLKEVHWRSKGIKPTLKEYIDVATNSSGGLVLLLPSYFLTTDKLTEEGLDYVSKIPSIIRCSCKMTRLINDFSTSSHELARGDNLKALECYMNETGASEEAAREHIMHMVREAWKWMNRAVFEDYQIPGLRPFLGACLNMARICHTFYGCGDGFGQPSNDTKDSLASAIYEPVPLD

>EglobTPS093

MALQALSTSFLPSSFHHNQSPLLFFRHLRSSSSAATSSTTSSAQFVTCASKIEVQEIGRHSANWQPSVWDYDFLQSLTVNYTEDKCSEEVQRLKKEVKGLFDGEMNQVAKLKFIDVVQRLGLGYRFEMEIKNALSSIYNNTEDAQLSDNLDVVSLRFRLLRQHGYNTPQDVFQRFMSKTGTFNESLNEDVKGLLGLYEASFHGLEGETILDEAWTFASKHLKDLNLNEIPTNLASHVSHALDMPIHWRLNRLEARWFIDMYKKQQDMIPSLLRLAKLDFNLVQSVYTKEVSNLARYTINLAARWWVELGANKMTFCRDRIVESYFWSNSMVFEPQHTAYREMNGKLASMVVLIDDVYDIYGTPEELELLTDFIVRFRWDITDIDRLPPIVRDSFMAMYNTTNEIGYWTMRERGINPIPYLRKVWAEECKAYLKEVHWRSKGIKPTLKEYIDVATNSSGGVVLMLPSYFLTTDKLTEEGLDYVSKIPSIMRCSSKMLRLINDLSTSSHEVARGDNLKALECYMNETGASEEAAREHIMHMVREAWKWMNRAMFEDYRIPGLGPFLGACVNTARICHTFYGCGDGFGQPSNITKDSLASAIYDPVPLD

>EglobTPS095

MALPALSTSFFPSSIHHNQPSLLFFRHLRSSSSAATSSTASGAQFVTCASKIEVQEIGRHSANWQPSVWDYDFLQSLGVNYTEDKCSEEVQRLKKEVKGLFDREMNQVAKLKFIDVVQRLGLGYQFETEIKNALSSIYNNTEDAQLSDNLDVVSLQFRLLRQHGYNIPQDVFQRFMSKTGTFNESLNEDVMGLLGLYEASFHGLEGETIIDEAWTFASKHLKDLNLNEIPTNLASHVSHALDMPIHWRLNRLEARWFIDMYKKQEDMIPSLLRLAKLDFNLVQSVYRKEISNLARYRWWVELGANKMTFCRDRIVESYFWSNSMVFEPQHTAYREMNGKLASMVVLIDDVYDIYGTPEELELLTDFIVRFRWDITDIDRLPPIIRDSFMAMYNTTNEIGYWTMRERGINPIPYLRKVWAEECKAYLKEVHWRSKGIKPTLKEYVDVATNSSGGVVLMLPSYFLTTDKLTEEGLDYVSKIPSVMRCSSKMLRLINDLSTSSHEVARGDNLKALECYMNETDASEEAAREHIMHMVREAWKWMNRAVFEDYRIPGLGPFLGACVNTARICHTFYGCGDGFGQPSNITKDSLASAIYEPVPLD

>EglobTPS097

PVPCSASTRAALSQGGWRSPRYQPTLWSYDYLRSLPTSFLEQEVRSAMKDESAELSTILALVDDIQRLGLVFLFEEDVKRALRRYHSPDGGYKNRDQKTLHGTALFFRILRQNGFEVSDVFRIFMDERGTFMESLGRDVEGLLSLYEASHLAFEEEGILLEAKEFAVKHLKRLNDIDNGKDLEYFRVNRGSVPALHQRMPLLEARQSIEAYSPQRDVERRLLELAVYNFNMVQSILQRDLQEMSRSVWERGRWWNDVSLANELSFARDRLMECFFWTVGMAYEPQFSNLRKGLTKVTALVTTIDDVYDVYRSMDELELFTDAVWDVNALSNLPSCMKLCFLALYNAVHEMAYDVLKQNGENIIPCLTKAVSWSDMFKAFLQEAKWKHDKVTPTFEEYMNNGWISVSGLVILIHAFFLSTPDVRKEEIELIETHGHDLLKSPAIIFRLCNDLGTSSVRIAELERGETANSILCYMQDTGVSENVAREHIKELIDVEWKNMNRYQVDDSMFGKSFVRLAFNLARIAHYTYQDGDAHGDPDDRAKYRIHSLLIDPISL

>EglobTPS098

RVQMLEQEVRSAMKDENAELSTILALVDDIQRLGLIFLFEEDVKRALRRYHSLDGGHKNRDQKTLHGTALYFRILRQNGFEVQTDVFWIFMDEQGTFMESLGRDVEGLLSLYEASHLAFEDEDILHEAKAFAIEHLKRLNNIDVSKDLEYFQVNWGLALPLHQRMPLLEARRSIEAYRTRRDADRRLLELAVYNFNMVQSILQRDLQEMSRANETALVTTIDDVYDVYGSLDELELFTDAVHRFRWDVDAVSSLPGCMKLCFLALYNAVHEMAYDVLKQNGENIIPCLTKAWSDMLKAFLQEAKWKHNKVTPTFEEYMNNGWISVSGLVILIHAFFLSTPHIRKEELELIETYGHDLLKSPSIIFRLCNDELERGETANSILCYMQDTGVCENVAREHIKELIDSTFTLPRIAHHTYQDGDAHGAPNDRSKYRIHSLLIDPISL

>EglobTPS099

MALHLFSLPSVFSEKISRQVPRSTSTKAAFPQGGRRSANYQPSVWTHNDLPSLVTDEDRQSCRVVKVELQREKAQMVEEVRGALHDENAELITIFALVDDIQRLGLGRHFEEDISRALHRCLSPDVVYEGLQKSLHGTALSFRILRQHGFEVSQDVFKIFMDESGSFLKNLGQGMLSLYEASRLAFEDEDILREAETFTIEHLKNHNRDINKDLQGEVNHELEWPLHRRMSLLEARRFIEAYSRRRYTSHRILKFSATNFNTLQSTLQGDLQEVLWWDNVGLANELNFARDRLVECFFAAVAVADEHPLSNCRKGLTKANILNVIIDDVYDIYGTLDELGCSQVHRSSTNAVEDLPGYMKLCFLALYNCVNELAYDTLKETRENVIPYLTKAVHDWYDACEAFLQEAKWSHNKITPRVEEYLNNGWISVSGHVMLIHAYFLSSPSMRKEELESLEHYHDLLRLPSMIFRLTNDLATSSAELERGETTNSIWCYMQEMGVSELEARKYVIKMIDTTWKKLNKYLVNDSTFNQSFVRMAFNLARMAHCMYHDGDAVGAPDDLSMNRVHSLIIDPVSLEP

>EglobTPS100

RVQMLEQEVRSAMKDENAELSTILALVDDIQRLGLIFLFEEDVKRALRRYHSLDGGHKNRDQKTLHGTALYFRILRQNGFEVSKEQTDVFWIFMDEQGTFMESLGRDVEGLLSLYEASHLAFEDEDILHEAKAFAIEHLKRLNNIDVSKDLEYFQVNWGLALPLHQLIRWWNDVSLANKLSFARDRLMECFFWTVGMAYEPQFSNLRRGLTKVTALVTTIDDVYDVYGSLDELELFTDAVHRFRWDVDAVSSLPGCMKLCFLALYNAVHEMAYDVLKQNGENIIPCLTKAWSDMLKAFLQEAKWKHNKVTPTFEEYMNNGWISVSGLVILIHAFFPPHIRKEELELIETYGHDLLKSPSIIFRLCNDAELERGETANSILCYMQDTGVCENVAREHIKELIDTAWKKMNRYQVNNSLFGKSFVRLAFNLARIAHHTYQDGDAHGAPNDRSKYRIHSLLIDPISL

>EglobTPS101

LATPLLKLSSLTANGRVHCSVSTQVSDTQGGRRWANYQPTVWTYNYLSLVADGGRQSHCAVKVELQREKAQMLEEEVRGALNDEKAEPMTIFALVDDFQRLGLGQHFEEDISRALRRCLSNDAVNKSRQKSLHGTALSFRILRQHGFEVSQDVLKIFMDESGSFMKTLGGDVQGMLSLHEASHLAFEEEDILHGARSFAIEHLRNLNRNVNKDLQDQVKHELELPLHRRMPLLEARQSIEAYSRHGYTNHRILEFAVLNFNTSQSILQRDLQEMWWNNVGLANELNFARDRLVECFFAAVAVADEHPLSNCRKGLTKVNILNVIIDDVYDIYGTLDELELFTDAVRRRWDINAVEDLPGYMKLCFLALYNSVNELAYDTLKETRENVIPYLTKAVHDWYDSCEAFLQEAKWSHNKITPRVEEYFGWISVSGHVMLIHAYFLSSPSMRKEELESLEHYHDLLRLPSMIFRLTNDLATSSAELERGETTNSIWCCMQEMGVSELEARKYVIKLIDTTWKKLNKYLVNDSTFNQSFVRMAFNLARMAHCVYHDGDAVGAPDDLSRNHVHSLITDPVSLEPC

>EglobTPS102

MALRLLSAPYLPKLPSPTANGRVHCLASTQVSDTQGRRRSANYQPTVWTHNYLQSLDSDESRQSRHAVKQREKAQMLEEVRGALNDEKAEPMTIFALVDDFQRLGLGQHFEEDISRALRRCLSNDAVNKSRQKSLQGTALSFRIFRQHGFEVSQFKIFMDKSGSFMKTLGGDVQGMLSLHEASHLAFEEEDSLQEARSFAIEHLRNLNCNVDKDLKDQVKHELELPLHCRMPMLEAQSIEAYRRCGYTNHRIPEFAVTNFNTSQSILQRDLQEMEKVNFVRDRLMECFFWAVGVADEPTLANCRKRLTKVTSLITIIDDVYDVYGTLDELELFTDAVRRWDINAVDDLPGYMKLCFLALFNSVNEIAYDTLKETGKIVIPYLAKWYDLCKSFLQEAKWSYNKTNPRFEEYLNNGWISSSGHVILIHAYFLSSPSMRREEPESLEHYHDILRLPSMILRLTNDLGTSSELERGETTNSIMCYMQEMGVSESEARDYVMKLIDTSWKQMNKYLVNGSTFDQSFVRMAYNLARTTHFMYQDGDAHGAPDNRSRNRMHSLIIEPISL

>EglobTPS103

LLKLSSLTANGRVHCSVSTQVSDTQGGRRWANYQPTVWTYNYLQSLVADGGRQSRCTVKVELQREKAQMVEEVRGALHDENAELITIFALVDDIQRLGLGRHFEEDISRAVCLSPDVVYEGLQKSLHGTALSFRILRQHGFEVSDVFKIFMDESGSFMKTLGNDVQGMLSLYEASRLAFEDEDILREAETFTIEHLKNHNRDINKDLQGEVNHELEWPLHRRMSLLEARRFIEAYSRRRYTSHRILKFSATNFNTLQSTLQGDLQEVLWWDNVGLANELNFARDRLVECFFAAVAVADEHPLSNCRKGLTKANILNVIIDDVYDIYGTLDELYSQTLFAGPRWDINAVEDLPGYMKLCFLALYNCVNELAYDTLKETRENVIPYLTKAVHDWYDACEAFLQEAKWSHNKITPRVEEYLNNGWISVSGHVMLIHAYFLSSPSMRKEELESLEHYHDLLRLPSMIFRLTNDLATSSAELERGETTNSIWCYMQEMGVSELEARKYVIKMIDTTWKKLNKYLVNDSTFNQSFVRMAFNLARMAHCMYHDGDAVGAPDDLSMNRVHSLIIDPVSLEP

>EglobTPS104

VELQREKAQMLEEVRGALNDEKAEPMTIFALVDDFQRLGLGQHFKEDISRALRRCLSNDAVNKSRQKSLHGTALSFRILRQHGFEVSQDDFKIFMDKSGNFMKTLGGDVQGLLSLHEASHLAFEEEDILQEARSFAIEHLRNLNCKVDKDLQDQVKHELELPLHCRMPMLEAQRSIEAYRGCGYTNHRIPKFAATNFNTLQSILQRDLQEMWWNDVSLARNLNFVRDRLTECFFWAAGVADEPTLTNCRKRLTKVTSLITIMDDVYDVYGTLDELELFTDAVRRWDINAVDDLPGYMKLCFLALFNSVNEIAYDTLKETGKIVIPYLAKSYVNLVAPRFEEYLNNGWISSSGHVILIHAYFLSGPSMGREELESLEHYHDILRLPSMIFRLTNDLVTLSELERGETTNSITCYMQEMGVSESEARDYVMKLIDTSWKQMNKYLVNGSTFDQSFVRMAYNLARTTHFMYQDGDAHGSPDNLSRNRMHSLIIEPISLEPC

>EglobTPS105

YQPTVWTHNYLQSLEADESHQSRRAVKVELQREKAQMLEEVRGALNDEKAEPMTIFALVDDIQRLGLGQHFEEDISRALRRCLSNDAVNKSRQKSLHGTALSFRILRQHGFEVSQGIDVFKIFTDESGSFMKTLGGDVQGMLSLHEASHLAFEEEDILQEARSFAIEHLRNLNCNVDKDLQDQVKHELELPLHCRMPLLEARWSIEAYRRCRYPDHRIPEFAAMNFNTLQSILQRDLQEMWWNDVSLARNLNFVRDRLMECFFWAAGVADEPTLANCRKRLTKVTSLITIIDDVYDVYGTLDELELFTDAVRRWDINAVDDLPGYMKLCFLVLFNSVNEIAYDTLKETGKIVIPYLAKWYDLCKSFLQEAKWSYNKTNPRFEEYLNNGWISSSGHVILIHAYFLSSPSMRREELESLEHYHDILRLPSMILRLTNDLVTSSELERGETTNSIMCYMQEMGVSESEARDYVMKLIDTSWKQMNKYLVNGSTFDQSFVRMANNLARTTHFMYQDGDAHGAPDNRSRNRMHSLIIEPISL

>EglobTPS106

MALRLLFTPHLPVLSSRRANGRVRCSASTQISDPQEGRRSANYQPSVWTYNYLQSIVAGEGRQSRREVKVEQQKEKVQILEEEVRGALNDEKAETFTIFATVDDIQRLGLGDHFEEDISNALRRCVSKGAVFMSLQKSLHGTALGFRLLRQHGYEVSQGIDVFKIFLDESGSFVKTLGGDVQGVLSLYEASHLAFEEEHILHKARSFAIKHLENLNSDVDKDLQDQVKHELELPLHRRMPLLEARRSIEAYSRREYTNPQILELALTDFNVSQSTLQRDLQEMLGWWNNTGLAKRLSFARDRLIECFFWAVGIAHEPSLSICRKAVTKAFALILVLDDVYDVFGTLEELELFTEAVRRRWDLNAVEDLPVYMKLCYLALYNSVNEMAYETLKEKGENVIPYLAKAWYDLCKAFLQEAKWSNSRIIPGVEEYLNNGWVSSSGSVMLIHAYFLASPSIRKEELESLEHYHDLLRLPSLIFRLTNDAELERGETTNSIRCFMQEKGISELEARECVKEEIDTAWKKMNKYMVDRSTFNQSFVRMTYNLARMAHCVYQDGDAIGSPDDLSWNRVHSLIIKPISPAA

>EglobTPS107

MALPALFTTFLPSLTGYNQPSLFFSRLPRSSSSSSSSSTASGPQFMTRALKIEGQEIVRRSAEDQYTKQVQRLKKEVKGLFEREMNQVDKLEFFDVVQRLGLGYHFETEIKSALSLIYNNTEPSVWDYGLVQSLGVDYSDAQLSNDLYAASLRFRLLRQHGYNVPQDVFQRFMNMTGTFNESLSKDAKGLLGLYEASFHGLDGETILDEAWNFASKHLKDLNLDKVPSNLASNVSHALDMPIHWRPNRLEARWFMDMYEKQQDMIPSLLRLAKIDFNLVQSIHRKEVGNLARYRWWVELGANKMTFFRDRLVESYFWTCIMAFEPQYTAFREMCTKIGCMVTLIDDVYDIYGTPEELELLTDFIIRFRWDITDIDKLPPTIRNSFMVLYNTTNEVGYQTMRDQGINPIPYLRKVWADECKAYMKEVHWYNSGIKPALKEYMDVAVDSIGGLILLLHSYFLTTDKLTKEGLDHVSKIPHELARGDNSKALECYMNESGASEEVAREHIRHLVRNIWKKMNKDVFEDYPFSGFGPFLGACLNLARASHCFYEYGDGHSLPGHQNKDHLVSTIFESVPLD

>EglobTPS108

MALPVLFPSFLPSSISHNQPSLLSFRHPRCSSSSFSSGAKSVTCAATIENPEIVRRSANWKPNVWDYEFLQSLRVDYTEDKYAEQVQRLKEEIKGLFNREMNQVAKLEFIDVVQRLGLGYHFEKEIKNALSSIYDNTGYAQLLNDLYAISLGFRLLRQHGYNIRQGIDVFQQFMNKTGTFNESLNKDVKGLLGLYEASFHGLEGETMLDEARNFASKHLKDLNLDKVPTMLASYVSHTLDIPIHWRPNRLEARWFMDMYEKQQDMIPSLLRLAKLDFNLVQSVHKKEVSNMARYRWWVELGANKMTFFRDRLVEHYFWNCTMVFEPQYTAYREMTTKLACMVTLIDDVYDVYGTLEELELLTDFLVRLVLYDRWDITEIDKLPPTIRDSYMALYNTTNEIGYWTMRELGINTIPYMRKVWADECKAYIKEAHWYNKGIKPTLKEYMDNAVDSIGGSIMLLGSYFLTTDKLTEEGLDYVSKIPSVMHCSTKILRLNNDLSTSSYELARGDNFKALECYMNETGASEEAAREHVKQMVHETWKRMNKDVFEDYPYSGLGPFLDACMNFARASQCFYQYGDGHGLPDNETKDHLVRALFDPVPLD

>EglobTPS113

PHDLSFAKVAHPSSGITKRVEATKSMLGSMTDGEISISAYDTAWVALVEDVSGSGSPQFPEALRWIVDNQLPDGSWGDDLIFSPHDRIINTLACAQQVRNKKKRIPKDIMHQVPTTLLHSLEGMEGLNWEKLLKLQSADGSFLFSPSSTAFALMQTKDANCLDYLSRAVQRFNGGVPNVYPVDLFEHLWAVDRLQRLGVSRYFKDEIKECMSYVHRYCIRYWSEKGICWARNSRISDIDDTAMGFRLLRLHGHEVSAVSSDVFEQFKRGDVFSTFMGQSTEAVTGMFNLYRASELIFPGEKILEDAKSHAVKFLRRKREANELLDKWIRTKDLPGEVRVETRFYIEQYGGENDVWIGKTLYRFYLELAKLDYNNCQALHLSEWDNFQRRWYYESKLVDFGTSGKTLLYSYFTAAASIYEPERARERLAWAKTSVLVDAIASYCSRSSIPSKGGQGLIAALLGTLDQLSLEVLVARGKDIGHALRIAEKWLVGYEEEGDKYKGVVELLVQMIALGSGNSSFSDGRSSHPQYRHLCNLTNTICRHLAHRQTQKACENEDLPIQMAMQELVQLVLQDSAHRLDRDVKDAFFSVTRSFYYTAHCDPATIKSHIAKVLYERV

>EglobTPS114

SSEIGERVKAIKLMFWSMSDGKISTSAYDTAWVALVEDVGGSGNPQFPEALQWIANNQHPDGSWGDDLLFCPHDRILNTLACVVALKYWKIHPDKCDKGVGMSFFKDNISKLAEEKPEHMPIGFEVAFPSLIETAWKLEIEICDDSPVLQEIYANRNTKLNKRIPSDLLHQVPTSLLHSLEGMKELKWEKLLKLKNADGSFLFSPSATAFAFLQTRDLNCLNYLSRTVQRFHGGVPNVYPVDLFEHLWAVDRLQRLGVSRYFKEEIKECMSYVYRYCIRYWSDKGISWARNSNVADIDDTSMGFKLLRLHGYEVSAGTCQLLLRFFSFSCHSVSLEVFENFKNGNEFFSFMGQLIEPVTGMLNLYKASELIFPGEKILEDANRHAAKFLRRKQEANELFDKWIITKDLPGEVVGYALDVPWYANLPRVESRFYIDQCGGQNDVWIGKTLYRRMEYVDNDVYLELAKLDYNNCQALHLSEWDNFQRWYCESKLVDFGISGKTLLYSYFTAAASIYEPERAGERLAWAKTRVLVDAIASYLEGEGATREQRGAFVRAFIPDERGEGLIAALLGTVDQLSLEVLEARGKDISHPLRWEKWLVGYGEEGDKYKGVVELLVQMIALGACNSLLSDDLSSHPECHRLCNLTSTICRQLAHHQTQKVRKNENDQIEMAMQELAQLVLQNSTRGLNRDVKDAFFAVTRSFYYTAHCDTGTINSHIAKVLFER

>EglobTPS115

MSNKVELSISWYDTAWVAMVPFPGSPKSHRFPRSLSWLLNNKATPIRFNILLSGMIEQAGCLNLNLPLRSANLDSVSYERNFAVLRGLSEGSRIYLSHVSEGMGSLSDWEMIKKHQRKNGSLFNLPSTSAAALTHLQNVGCLRYLKLVVEKFGDASAPTIYPLGIYARLCMTENLERMGINCHFRKEIIDTLDDTYATSAMAFRILRSHGYDVSSRGEEEIFQHIATSAIAFRILCSHGYNVSSDNLFSCAIDQCGEIILDKINSWTGDFLKEGLLAGEMHADRLKDDMHEVDFPLDIQVDDALRFPLEESLTRAANRINIQLHNTNSMGILKCELQNFRNKDFLNLAMQDFNMCQAIILFSYARSYNFCNKDFLNLAVIDFNNCAIREEFKYLKRWVKEKKLDLKLARQKLAYCYFSAAETFLARQLLDTHISWAKNSVVVTVADDLFDVSGSAEGNEVLIRLLRKTISETGDKAVTWHGRNLTVHVAQIWLETLESMLTEAERARKKTVPTMDEDIANAHVSFGLGPTVLPALYLVGPKLSEKQVESPEYHNLFRLMSTSRRLLNDIQSYERESKQGKLHAVTLQMLDGSGTSEREAIERISSIIISMRRELLKLVLQEKDSIIPRACKDLFWKMSAVMHLFYMNADGFASDEKTSAVKALLDQPITLNEL

>EglobTPS116

EGSKERISDMFNKVELSLSSYDTAWVAMVPSPCSPQSPLFPRSVSWLMNNQLCDGSWGLPDHHPLLIKDALLSTLACVLALKQWGVGERQINKGLEYIASNSASVIDDRQHTPIGFNILLSGMIEQADCLNLNLPLRPADVDSVSYKRNLEVKRGRYLSYVAEGMGSSADWEMIMKYQRKNGSLFNSPSTTAAALTHLQNASCLHYLESVLEKFGDASVPTIYPLEIYARLCMIENLERLGIDRYFRKEVIHVLDDTYRTNRCWLQGEEEIFLDIATTAMAFRMLRSHGYDVSSEDQFCNTLEGYVKDAGSVLELYRASQLIINDDEIILDKINSWTYDFLRKGLHTGKMHANRLESYICGEVQVDDALKFPLHANLERVANRRNIELYNIDSTRVLKCGLRYSSCNFCNKDFLNLAVNDFNNCQAIYQEELKYLERRWVKEKRLDKLKFARQKLAYCYFSAAASFSPPQLSEARISWAKNGVLTTVVDDFFDVGGSAEELENFIWLVKRRWNVNMSADCCSEQVQIIFSALHSTISEIGDKAVTWQGRNVTGHVAQIWLELLESMLTEAKWTRKKVVPTMDEYMANAYVSFALGPIVLPALYLVGPKLSEEQVESLEYRKLFKLMSTCGRLLNDMQGFKQRESREGKLNAVTLQMLDRSSTSEREATERISSIIISKRRELLKLVLQERDSIVPRACKDLFWKMSTVLHFFYMDDDGFTSDEKTSAVKALLDQPITLNEL

>EglobTPS117

SQKSSIQSLINMIKRDLLSVIGSHSFLSPSPYDTAWLAMIPDPGLKFIHKNAELLLSRYTHGKFSRWIAIVLPGMVELARASSLEVVFPESVDRALADLFINRRQILREELVDKNQYCPLLSYLEALPSTYKISHETILKHLDSDGSLFQSPSATSRAYLSTGNEACLAYLQSLASNCASSGVPSLYPVDEDLTKLSMVHQLVRLGLTEYFDRENDEILAKIYRHINISFFRNYKHEKQITKSIHSIAAELYKDCLEFWLLRMHGYRVSPCMSCELYKFLNWFLFCWFLDHEEVRDHIWNTCNLMLPDEYELEKARIFSKKFLEKIASRETRDSSIISSSHCRMIEHELGLPWMARLDHLEHRTWMEEKDACVLWMGKFPYNRYLCLKRDSLLFFGFIRPSFVHNQDIVQLALQNYVLRQSVYRMELDVVKRYRWSETTGLRKMGFGREKTLYSYFAVAASISLPCNSDVRVLVAKSAIMITVADDFFDMEGSLEDLEKLTNAVQRYRWDGEGLTAHAKTIFEALDDLVTDFRMKCFKQSGKDIKKNLQEIWGETFHSWLMEAKWSKSGGAPPTQEYLDVGMTSIAAHILVLPSSCLASPTTPLHQLWSNAYQPITKLLMVITRLLNDIQSYLILSPCFYDFLTIQKEEKQGKLNFVLLYLKENPEASIEDSINFVQLLLEQLKKEFLLHVLEELCNLPEPSRRLHLGCLKVFHMFFNSSNRYDSETGMLHDIQKA

>EglobTPS119

IQIFMEFQKPSIQSLVQMIKREILPNMSSHSFLSSSPYNTAWLAMIPNPYQHDLPMFKGCLNWVLQNQNEEGFWGDYDHDEDEVSNKVECLASTLICMIMLKKWHVGLPSIEKGIREKLVDKKQYFPLLSYLEALPPTYKVSHETILKHLDSDGSLFQSPSATASAYLATGNACLAYLQSLALNCASNGGHINISFCRNYKHEKPIVKSIHSIAAELYKDCLGFWLLRMHGYKVSPCTSCKLYESLNWFLCWFLDREEVRDHIEKHYEYFSSVLLNIYRASNLMLPNEHELEKARTFSKKFLEKIASRGTRDSSIISSSHCRMVSGNIQIKHELGLPWMARLDHLEHRMWMEEKDACVLWMGKFSCHRFIRSSLVHNQDILQLALQNFEQRQSIYRMELDVLKRWSETTGLSKMGFGREKTVYSYFAVAASVSVPCNSDVRVLVAKGAVILTVADDFFDMEGSLEDLEKLTEAVQRYRWDGEGLSAHAKTIFEALDDLKCFKQLGKDIKKNLQNILVRKWGETFHSWLIEAKWSRSGDVRPTQEYLDVAMTSVGAHVLVLPPSCLASPTTSLHQLWSNPYQPITKLLMVISRLLNDIQTYDFLTFQKEEKQGKLNFVLLYLKENPGASIEDSINFVQLLLDQLKKEFLQHVLEEPCSVPELSRLLHLACLKVFNMFFNSSNRYDSDTDMLHDIQKANCALLFLKENPEASIEDPIKFVQLLLNQMKKEFLQHVLGDVCNLPEPGRRLHLGSLKVFHMFFNS

>EglobTPS120

NIVSSSFLSPSPYNTAWLAMIPNPHRHDCPMLCLNWVLHNQNEEGFWGDYDYEEHEMSDGGECLASTLVCMTVLKKWHVGSTLIEKGLVALATRPRHYFLPNHPKDLIIPVSTLAFCREKLVDRNQYYPLLSYLEALPPTYKIDGDGSLFQSPSATASAYLSTGHKTCLAYLQSLASNCSFCWFLDREEVRDHIEKHYEYFSSVLLNVYRASNLMLSDEQQLERVRTFSRKLLEKILSGETRDECIISSSHIEHELGLPWMARLDHLEHRMWLEEKDACVLWMGKLSVHNQDILQLALQDFVLRQSTYRMELDDEFRWSETTGLSKMEFCREKTTYSYFAVAASISLPCNSDIREVVAKSAIIVTVADNFFDMEGSLEDLEKLTDAVQRYRWDGEGLSGHAETIFKALVDLVTDFRVKCFKQSGKDIKKNLQDIWGETFHSWLMEAKWSRSGGAPPMQEYLDVGMTSIATHILVLPSSLHASPTTPLHQLWSSPYQPITKLLMVITRLLNDIQSYQQKEEKQGKLNFVLLYLKENPEASIEDSINFVQHLLDQLKKELLQHLKVFHMFFNSSNRYDSDMDMFHDIQKALVVPPRVPK

>EglobTPS121

MESQKSSFQSLVSTIKRDILSVMGSHSFLSPSPYDTAWLAMIPDPHRHERPMFEGCLNWVLQNQNEEGFWGHHDRPMFEGCLNWVLQNQNEEGFWGHHDYDRHEMPGGVECLASTLVCMTVLKKWHAGSPLIEKGGLKFIHKNAELLLSRYTHGKFSRWIAIVLPGMVELARASSLEVVFPESVDRALADLFINRRQSPFCREELVDKNQYCPLLSYLEALPSTYKISHETILKHLDSMVLYFNHPGSLFQSPSATSRAYLSTGNEACLAYLQSLASNCEAVPSLYPVDEDLTKLSLVHQLVRLGLTEYFDRENDEILAKIYRFCWFLDHEEVRDHIEKHYEYFSSVLLYIYRASNLMLPDEYELEKARIFSKKFLEKIASIEHELGLPWMARLDHLEQRRWSETTGLRKMGFGREKTLYSYFAVAASISLPCNSDVRVLVAKSAIMITVADDFFDMEGSLEDLEKLTNAVQRYRWDGEGLTAHAKTIFEALDDLVTDFRMKCFKQSGKDIKKNLQEIWGETFHSWLMEAKWSKSGGAPPTQEYLDVGMTSIAAHILVLPSSCLASPTTPLHQLWSNAYQPITKLLMVITRLLNDIQSYELILSPCFYDFLTIQKEEKQGKLNFVLLYLKENPEASIEDSINFVQLLLEQLKKEFLLHVLVELCNLPEPSRRLHLGCLKVFHMFFNSSNRYDSETGMLHDIQKA

>EglobTPS123

SSSPYNTAWLAMIPDPHQHDLPMFKGCLNWVLQNQNEEGFWGDYDHDEDEVSNKVECLASTLICMTMLKKWHVGSPLIEKGGLKFIHENMELLLPRNKHGKFPRWIAIVFPGMVDLARASGLEVVFPESVERIIADLFSNRQRILKRREKLVDMKQYFPLLSYLEVSHETILKHLDSDGSLFQSPSATASAYLATGNEACLAYLQTLALNCASNGGHINISFCRNYKHEKPIVKSIHSIAAELYKDCLGFWLLRMHGYKVSPCTISIVFCCWFLDREEVRDHIEKHYEYFSSVLLNIIEPAILCFRTNMNSRKQELSQRNFLRKSYQDILQLALQNFEQRQSIYRMELDVLKRRWSETTGLSKMGFGREKTVYSYFAVAASVSVPCNSDVRVLVAKGAVILTVADDFFDMEGSLEDLEKLTEAVQRYRKWGETFHSWLIEAKWSRSGDVRPTQEYLDVAMTSVGAHVLVLPPSCNDIKTYEVKFLWPSSYNWPLLPKWRSFIVALSPCFYDFLTFQKEEKQGKLNFVLLYLKENPGASIEDSINFVQLLLDQLKKEFLQHILEEPCSVPE

>EglobTPS125

REKLVDRNQYYPLLSYLEALPPTYKISHETILKHQDNDGSLFQSPSATASAYLSTGNETFLACLQSLVSFCWFLYREEVRDHIKKHYEYFSNVLLNVYRASNLMLPDEHKLEKARTFSKKFLEKIASGGTRGESILSLPWMARLDHLEHKMRLEEKDAFILWMGKLSCHRRSSLVHNQDILQLALHNFVLRQSIYRMELDVVKGYLCIWSSLINESRWSETTGLSKMGFGREKTTYSYFAVATSVSLPCNSDIREVVAKSAIIITVADDFFDMEGSLEDLEKLTNAVQRYPITKLLMVITRLLNDIQSYQQKEEKQGKLNFVLLYLKENPKASIEDSINFVQYLLEQLKKEFLKHVLEEPCSLPKPSKRLHLGCLKVFHMFFNSSNRYDSDMDMFHDIQKALVVPPLIPKLKSPMPLPEQLGPKPRVSVTKSLSGQFSLERFPRKSFVGCQMPP

>EglobTPS126

MEAQISLIQSLVNTIKREILLPNMVSSSFLTPSPYNTAWLAMIPNPHRHDCPMFKGCLNWVLHNHNEEGFWGDYDYEEHEMSDGGECLASTLVCMTVLKKWREKLVDRNQYYPLLSYLEALPPTYKISNETISVDGDGSLFQPPSATASAYLSTGHKTCLAYLQSLFCWFLDREEVRDHIEKHYEYFSSVLLNVYRASNLMLSDEQQLERARTFSRKLLEKILLRETRDECIISSSHRRMVIEHELGLPWMARLDHLEHRMWLEEKDACVLWMGKLSWHRNQDILQLALQDFVLRQSTYRMELDVVKRWSETTGLSKMEFCREKTTYSYFAVATSISLPCNSDIREVVAKSAIIVTVADDFFDMEGSLEDLEKLTNAVQRYWGETFHSWLMEAKWSRSGGAPPMQEYLDVGMTSIATHILVLPSSCLASPTTPLHQLWSSPYQPITKLLMVITRLLNDIQSYQQKEEKQGKLNFVLLYLKENPEASIEDSINFVQHLLDQLKKELLQHVLEEPCSLPKPSRRLHLGCLKVFHMFFNSSNRYDSDMDMFHDIQKALVVP

>EglobTPS128

MEFQKSSIQSLVNMIKRDILSIMGSHSFLSPSPYDTARLAMIPDPRRHDHPMFEGCLNWVLHNQNEEGFWGYHDYDTHEIPDGVECLASTLVCRIVLKKWHAGSPLIEKGTACFTSNGLKFIHKNGELLLSRYKHRKFSRWIAIVLPGMVELARASSLEVVFPESIERALANLFINRRQILERQEELVDKNQYCPLLSYLEALPPTYKISHETILKHLDSDGSLFQSPSATSSAYLSTGNEACLAYLQSLASNCVPSLYPVDEDLTKLSMVHQLVRLGLTEYFDRENDEILGQIYRFRNYKHEKQITKSIHSIAAELYKDCLEFWLLRMHGYFCWFLDHEEVRDHIKKHYEYFSSVLFYIYRASNLMLPDEHKLEKARIFSKKFIEKIASRETRDSSIISSSHCRMVIEHELGLPWMARLDHLEHRTWMEEKDACLLWMGKFPYNRYCLKRNSLILSNQDIVQLALQNYVLRQSVYRMELDVVKRYRWSETTGLRKMGFGREKTLYSYFAVAASISLPCNSDVRVLVAKSAIIITVADDFFDMEGSLEDLEKLTNAVQRYRWDGEGLTAHAKTIFEALDDLVTDFRMKCFKQSGKDIEKNLQEIVSWGETFHSWLMEAKWSKSGAAPLMQEYLDVGMASIAAHILVLPSSCLASPTAPLHQLWPNAYLPITKLLMVITRLLNDIQSYKTLQKEEKQGKLNFVLLYLKENPLLEQLKKEFLQHVLEELCNLPEPSRRLHLGCLKVFHMFFNSSNRYDSETGMLHDIH

>EglobTPS129

HINISFFRNYKHEKQITKSIHSIAAELYKDCLEFWLLRMHGYRVSPCMSCELYKFLNWFLCWFLDHEEVRDHIGIHYEYFSSVLLYIYRASNLMLPDEYELEKARIFSKKFLEKIVSIEHELGLPWMARLDHLEHRTWMEEKDACVLWMGKFPYLFFGFIRPSFVHNQDIVQLALNYLLRQSVYRMELDVVKRYRWSETTGLRKMGFGREKTLYSYFAVAASISLPCNSDVRVLVAKSAIMITVADDFFDMEGSIEDLEKLTNAVQRYRWDGEGLTAHAKTIFEALEDLVTDFRMKCFKQSGKDIKKNLWGETFHSWLMEAKWSKSGGAPPTQEYLDVGMTSIAAHILVLPSSCLTSPTSPLHQLWSNAYQPITKLLMIITQLLNDIQSYENPEASIEDSINFVQLLLEQLKKEFLQHVLEEHCNLPEPSRRLHLGCLKVFHMFFNSSNCYDSKTGMLHDI

>EglobTPS131

RLAIDYHFEDEIEAILQRHLLISTSRSHSRSIDADNLHEAALRFRLLRQGGYPVPSGSFNDGTQNEKPQDNDILGLTSLFEASQLGIEGEDALDQVGESTRLRLHSSLADLDHVQARFVRNSLGNPFHKSLARFTANDFLRNFVGHSCSWTKNLGELAHLDMNIVRSVHQREILQVSKYTKPISFIYIIDDIFDVYGTMDELSSFMDVVNRYIIRWECTEKDNIPDYMRMCFHALDDITNEFSLAVYKNHGWNPLHSLRKESVDHLENIPEIVSSTASILRLWDDLGSAEQDEFQDGRDGSYVECYKREFQGSSEEAARDHVKKMISEAWKSLNKACLYPQPFTNSFSKASLNTARMVPLMYNYDDSHSLPLLEHHMKSLLFK

>EglobTPS132

KHDQKIKSLIDFLNKVVNEPVESLIIVDMIQRLGVKSLFREQIKARQYTHFSSLNHGKDDVYEIALRFRLLRQEGYRVPAGLFDVFEYFNEKGKGFVMKLEGNIKGMMELYEASQMSTEGEDILDEAECFSSKCLNALLTCDLDNEQARMIESTLQYPYRKSFARLLAPQSFVNDIANLWMEDLLEVANRKRRIDQYVHQKEIHQINKRWWKELGLGEKMEFARDQPLKWYMWSMAILTDPSLSELRVELSKPISLVYIIDDIFDVHGTVDELILFTELIKRYRWDNACAEQLPEYMKICFKVLNDIANDFGRIIFENHGWNPTRFLKQWANLCNAFLVEFQWNASGKLPKADDYLKNAIITSGVPLVLTHLMGQNIANQSMDSKKEEVQLPNIIYLIAEILRLWDDLGCAQSMKDENQNGYDGSYVDCYLGENEGSSYQSAREHVMKLISKLWKLLNKECLSPCPSSAPFLEACVNAAKMVSLMYNYEDKHGLGLLQDHMKSL

>EglobTPS133

DALQRLAIDYHFEDEIEAILQRHLLISTSRSHSRPIDADNLHEAALRFRLLRQGGYPVPSGVFQRFLHDGTQNEKPQDNDILGLTSLFEASQLGIEGEDALDQVGESTCLRLHSSLADLDHVQARFALGNPFHKSLARFTANDFLRNFVGHSCSWTKNLGELAHLDMNIVRSVHQREILHVSKWWKELGMAKELKCARDQLTETGLSQERVLVTKPISFIYIIDDIFDVYGTIEDLTAFTDRWECTEKDNIPDYMRMCFHALDDITNEFSLAVYKNHGWNPLCSLRKWASLLNAFLVEARWLASGHSPTTQDYLDNAIVSSGVHVLLVHLFFILGERIAPESVDHLENIPEIVSSTASILRLWDDLGSAEQDEFQDGRDGSYVECYKREFQGSSEEAARDHVKKMISEAWKSLNKACLYPQPFTKSFSKASLNTARMVPLMYNYDDSHSLPLLEHHMKSLLFK

>EglobTPS134

DNSGTKHEEKIKSIIGFLNNVGGEPVESLIIVDMIQRLGIKPLFQEQIKAILRWQYTHFTSLNHGKDNVYEIALRFRLHRRYAFYADVFEYFKDKGKGFIMKVEGNVKGMMELYEASQMSIEGEDILDEAKCFSSKCLNELLTCDLDPEQVRMIESTLRYPYRKSFARSLAPLSFVNNMPGVNSWIEDLLEVANRERRIVQSMHQKENHQINWWKESGLGEEMKFARDQPLKWYLWSIAILTDPLSELRVELVKPISLVYIIDDIFDVYGKVDELILFTGVIKRYTYRWDDACAEQLPEYMKKCFKVLSDITNDFGNIIFEKHGWNPTRFLKQWANLCNAFLVEFQWNASGTLPKADDYLKNGIITSGVPLVLAHLFFQMGQNIANQSMDSKKEEVPLPNTIFLVAEILRLWDDLGCAQSSKNKNQNGYDGSYVECYLRENQGSSYQSAREHVMELISKSWKLLNKECLSPCPFSAPFLEACVNAAKMVSLMYNYEDKNGLGLLQDHMKSL

>EglobTPS136

DDSRIKHDEKTKSLIDFLNKVVNEPVESLIIVDMIQRLGVKSLFREQLKIKAILRWQYTHFSSLNHGKDDVYEIALRFRLLRQEGYHVPAGLFADVFEYFNDKGKGFVMKLEGNIKGMMELYEASQMSTEGEDILDEAGCFSSKCLNALLTCDLDHEQARMIESTLQYPYRKSFARLLAPQSFVNDMPGANSWMEDLLEVANRKRRIDQYVHQEEIHQINKYWKELGLGEKMEFARDQPLKWYMWSMAILTDPSLSELRVELIKPISLVYIIDDIFDVHGTVDELILFTRWDNACAEQLPEYMKICFKVLNDIANDFGKIIFEKHGWNPWANLCNAFLVEFQWNASGKLPKADDYLKNAIITSGVPLVLTHLFFLMGQNIANQSMDSKKEEVQLPNIIFLVAEILRLWDDLGCAQSLKDENQNGYDGSYVECYLRENEGSSYQSAREHVLNKECLSPCPFSAPFLEACVNAAKMVSLMYYEDKHGLGLLQDHMKSL

>EglobTPS137

ISTVQDDSRIKHEEKVKSITGFLNNVVSEPVESLIIVDMIMRLGIKSLFQEQIKAILQWQYTHFTSLNHGKDDVYEIALRFRLLRQEGYYVPAGLYAFYADVFEYFKDKGKGFIMKLEGNAKGMMELYEASQMSIEGVDILDEAECFSSKCLNELLTCDLDPEQVRMIESTLRYPYRKSFARSLAPLSFVNDMPVANSWMEDLLEVANRERRIIQSLHRKEIHQIRWWKELGLGEEMEFVRDQPLKWYMWSMAILTDPSLSELRVELIKPISLVYIIDDIFDVYGKADELILFTEVIERYTVRWDNACAEQLPQYMKKCFKVLSDITNDLGNIIFEKHGRNPMGFLKQWANLCNAFLVEFQWNASGKMPKADDYLKNGIITSGVPLVLAHLFFLMGQNIANQSMDSKKEEVPLPNTIFLVAEILRLWDDLGCAQSLKDENQNGYDGSYVECYLRENQGSSYQSARKHVMELISKSWKLLNKECLSPCPFSAPFLEACVNAAKMVSLMYNYEDKHGLGLLQDHMKSL

>EglobTPS138

IKHEEKIKSIIGFLNNVGGEPVESLIIVRLGIKPLFQEQIKAILRWQYTHFTSLNHGKDNVYEIALRFRLLRQEGYYAFYADVFEYFKDKGKGFIMKVEGNVKGMMELYEASQMSIEGEDILDEAECFSSKCLNELLTCDLDPEQVRMIESTLRYPYRKSFARSLAPLSFVNDMPGLNSWIEDLLEVANRERRIVQSVHQKENHQINWWKESGLGEEMKFARDQPLKWYLWSIAILTDPSLSELRVELVKPISLVYIIDDIFDVYGKVDELILFTGVIKRRWDDACAEQLPEYMKKCFKVLSDITNDFGNIIFEKHGWNPTRFLKQWANLCNAFLVEFQWNASGTLPKADDYLKNGIITSGVPLVLAHLFFLMGQNIANQSMDSKKEEVPLPNTIFLVAEILRLWDDLGCAQSSKDENQNGYDGSYVECYLRENQGSSYQSAREHVMELISKSWKLLNKECLSPCPFSAPFLEACVNAAKMVSLMYNYEDKHGL

>EglobTPS139

DDSRIKHDEKTKIDFLNKVVNEPVESLIIVDMIQRLGVKSLFREQIKAILRWQYTHFSSLNHGKDDVYEIALRFRLLRQEGYHVPAGLFADVFEYFNDKGKGFVMKLEGNIKGMMELYEASQMSMEGEDILDEAGCFSSNCLNALLTCDLDHEQARMIESTLQYPYRKSFARLLAPQSFVNDMPGANSWMEDSLEVANRKRRIDQYVHQEEIHQINKRWWKELGLGEKMEFARDQPLKWYMWQFSMVHVAILTDPSLSELRVELIKPISLVYIIDDIFDVHGTVDELILFTEVIKRYTIMKICFKVLNDIANDFGKIIFEKHGWNPTRFLKQWANLCNAFLVEFQWNASGKLPKADDYLKNAIITSGVPLVLTHLFFLMGQNIANQSMDSKKEEVQLPNIIFLVAEILRLWDDLGCAQSLKDENQNGYDGSYVECYLRENEGSSYQSAREHVMKLISKLWKLLNKECLSPCPFSAPFLEACVNAAKMVSLMYNYEDKHGLGLLQDHMKSL

>EglobTPS140

DDSRIKHDEKTKSLIDFLNKVVNEPVESLIIVDMIQRLGVKSLFREQIKAILRWQYTHFSSLNHGKDDVYEIALRFRLLRQEGYHVPAGLFADVFEYFNDKGKGFVMKLEGNIKGMMELYEASQMSMEGEDILDEAGCFSSNCLNALLTCDLDHEQARMIESTLQYPYRKSFARLLAPQSFVNDMPGANLWMEDLLEVANRKRRIDQYVHQEEIHQINKWKELGLGEKMEFARDQPLKWYMWSMAILTDPSLSELRVELIKPISLVYIIDDIFDVHGTVDELILFTEVIKRYTIRWDNACAEQLPEYMKICFKVLNDIANDFGKIIFEKHGWNPTRFLKQWANLCNAFLVEFQWNASGKLPKADDYLKNAIITSGVPLVLTHLFFLMGQNIANQSMDSKKEEVQLPNIIFLVAEILRLWDDLGCARSLKDENQNGYDGSYVECYLRENEGSSYQSAREHVMKLISKWWKLLNKECLSPCPFSAPFLEACVNAAKMVSLMYNYEDKHGLGLLQHHMKSL

>EglobTPS141

FQEDIRGRHERRLEEQVRGDSLESLVMVDALQRLAVDYHFEDEIEAILQRHLLISTSQSHSRTIDADNLHEAALRFRLLRQGGYPVPSDVFQRFLHKMNDGTQNEKPQDNDILGFTSLFEASQLGIEGEDALDQVGESTRQRLHSSLADLDRFVRNSLGNPFHKSLARFTANDFLRNFVGHSYSWTKNLGELAHLDMNIVRSVHQREILQVSKWWKELGMAKELKCARDQPMKWYMWPMAILTETGLSQERVLVTKPISFIYIIDDIFDVYGTIEDLTAFTDVVNRYIIWSYRWECTEKDNIPDYMRMCFHALDDITNEFSLAVYKNHGWNPLCSLRKWASLLNAFLVEARWLASGHSPTTQDYLDNAIVSSGVHVLLVHLFFILGERIAPESVDHLENIPEIVSSTASILRLWDDLGSAKQDEFQDGRDGSYVECYKREFQGSSEEAARDHVKKMISEAWKRLNKACLYPQPFTNSFSKASLNTARMVPLMYNYDDSHSLPLLEHHMKSLLFK

>EgranTPS001

MSARFSVIPSSSLPQETGCVEGRRSANFHPSIWGDYFLKYASDSNSLEERIDRLKGEVRKMLTSAMYKPAEKLNLIDQIQRLGIAHFELEIDKELEQIRRGYFEFHCDDNNNDLDTIALLFRLLRQRGSHVSCIFNKFKDGDGNFGKSLIPDVQGLLSLFEACHLRYHGDDNLEDALAFTTTHLESVDKRKASLDLKKKVSHALNQPIHKGMSRLEMRCYIPLYQEESSHNEVLLSLAKLDFNLVQEQHRKELGNLTRWWKGLDVQRKFPFARDRLVELYVWWLGEYYEPEHEAAREILTKLSSVSSIIDDIYDVYGTWEELELFTETIQRWDVDAKDGLPEYMQECYKIVLDLYDEIGYEFSRKGHSYRLFYAKEVMKNQVRAYFVEAKCFHQNHVLTMEEYMSIALPSSGFVSILAWSFLGMGDIVTKDVFDWLLFNDPKMVKASSIICRLLNDIAGHFEQERGHVASAVECFMKQYRVTEEEAKEELRKQVTDAWKDINEELRRPTVVPMPILVRILNLTQALHMMYNGEIDNYTHAGTKMKEHVTSLLVNPLPM

>EgranTPS002

MSARFSVIPSSSLPQETGCVEGRRSANFHPSIWGDYFLKYASDSNSMEERIERLKGEVGKMLTSAMYKPAEKLNLIDQIQRLGITYHFELEIDKELEQIRKGYFEYHCDDNDNDLDTVALLFRLLRQRGYRVSCEIFNKFKDGDGNFGKSLIADVQGLLSLFEACHLRYHGDDNLEDALAFTTTHLESVDKRKASLHLEKKVSHALNQPIHKGMSRLEARHYIPLYQEEPSHNEVLLSLAKLDFNLVQEQHRKELGNLTRWWKGLDVQRKFPFARDRLVEMYVWWLGEYYEPEHEAAREILTKLSSVGSIIDDIYDVYGTWEELELFTEAIERWDVDAKDGLPEYMQECYKIVLDLYDEIGYEFSQKGRSYRLFYAKEVMKNQARAYLVEAKCFHQNHVPTMEEYMSIALPSSGIVSILAWSFLGMGDIVTKDVFDWLLFNDPKMVKASSIIGRLLNDIAGHFEKERGHVASAVECFMKQYRVTEEEAKEELRKQVTNAWKDINEELCRPTVVPMPILVRILNLTQALHMMYTGETDHYTNAGTKMKEVVTSLLVDPLPM

>EgranTPS003

MSFQISAVPSSSPRLGTGHVIQRRSAGYHPSIWGDYFVKYASPSTSMEEQIEELKGEVRKMLTNVVDKSSQMLHLIDQIQRLGIYYHFEHEIDEHLEEIHKHYSRLDHGNFKGDDLRMVALIFRLLRQQGYDVSSEVFNKFKDSEGNFRASLTSDVCGLLSLYEACHLRCHGDTILEEALPFAITHLESINESKVSTSLAKQVSHALKQPLRKGLPRLEARHYIPLYQEEPSHDEVLLTLAKLDFNLLQEQHQKELGKITRWWKDIDVPRNFPFARDRIVELFFWVSGVYFEPEFVEARDILTKVIALTSILDDVYDVYGTLEELVLITEAIQKWDVDAMDVLLEYMQVYYKELLHLYEEIGNEVAAKGRSYRLVYAKETMKRQARAFFQEAKWFQTNYTPTMEEYMPLQLKTTGYGMLATTSLVGMGNVVTKHAFEWSLSDCKIVKAAETICRLMDDISSHHFEQKRGHLVSSVELLMKEHGFSEQEAEKKLRKRVNDAWKDTNEEFLRPTAVPTPILTRVLNLSRAMDVLYSDGDNYTHSGTKLKGYVTSLFVSPLPM

>EgranTPS004

MSFQISAIPSSSPRLGTGHVIQRRSAGYHPSIWGDYFVKYASPSTSMVCGFSEEQIEELKGEVRKMLTNVVDKSSQMLHLIDQIQRLGIDYHFEHDIDEHLEEIHKHYSRLDHGDFKGDDLHMVALIFRLLRQQGYDVSSEVFNEFKDSEGNFRASLTSDVCGLLSLYEACHLRCHGDTILEEALPFAITHLESINESKVSTSLAKQVSHALKQPLRKGLPRLEARHYIPLYQEEPSHDEVLLTLAKLDFNLLQEQHQKELGKITRWWKDIDVPRNFPFARDRIVELFFWVSGVYFEPQFVEARDILIKVIALTSILDDVYDVYGTLEELVLITEAIQKWDVDAMDVLPEYMQVYYKELLHLYEEIGNEVAAKGRSYRLVYAKETMKRQARAFFQEAKWFQTNYTPTMEEYMPLQLKTTGYGMLATTSLVGMGDVVTKHAFEWSLSDCKIVKAAETICRLMDDISSHHFEQKRGHLISSVELLMKEHGFSEQEAEKELQKRVNDAWKDTNEEFLRPTAVPTPLTRVLNLSRATVMRYYTHSGTKLKGYVTSLFVSPPM

>EgranTPS005

DDGIEGLKGELRKMLVGAMDKPSQKLNLIDQIQRLGIAYHFEIEIYQQLEQIHKSYYELHDGDNDNDLHTIALLFRLLRQQGYAISSKVFSIVTIDDIYDVYGTLEDTFTEAIERWDVEVKDGLPEYMQVCYKIVLDLYDEIGYEVTRKGRSNYLLYAKEAMKNQVRAYFTEAKWFHQNHIPMMEEYMPIALSTIAIELLLVMLLLLGMGDTVTKDVFDWLLYSKPKIVNAMKIVCRLMDDIAGHFEQERGHGPSSMECFMKQYGVTEEEAKEELHKQVANAWKDINEGLCCSTNVPRQLLVRILNFTRVVHVVYKDEIDLYTHAGTKLKEHVTNLYVNPLPM

>EgranTPS006

MSLQILAIPSSSPAQETSRVVERRLANFHPSIWGDYFLKYASDSNSMSSAIADDRIEGLKGELRKMLAGAMDKPSQKLNLIDQIQRLGIAYHFEIEIYQQLEQIHKSYYELHDGDNDNDLHTIALLFRLLRQQGYAISCEIFNKFKDINGNFSESLIVDVQGLLSLFEACHMRFHGDDVLNDALAFAMTHLEAIDKGKASPNLKRQVRHALKQPIHKGIPRLEARHYISLYQEEPLHNEVLLSLTKLDFNLLQEQHQKELGNLTRWWKDLDVERKFPFARDRLVEMYLWMSGVYFEPEYEATRELLTKVFSIVTIIDDIYDVYGTLEELELFTEAIERWDAEVKDGLPEYMQACYKIVLDFYDEIGYEVTRKGRSDYLFYAKEAMKNQVRAYFTEAKWFHQNHIPTMEEYKSIALPTTAIELLLVMLLLGMGDTVTKDVFDWLLYSDPKMVNALKVVCRLMDDIAGHKFEQERGHGPSSMECFMKQYRVTEEEAKEELHKQVANAWKDINEGLCCSTNVPRQLLVRILNFTRVVHVVYKDEIDLYTHAGTKLKEHVTNLYINPLPM

>EgranTPS007

MSLQILAIPSSSPAHETSRVAERRLANFHPSIWGDYFLKYASDSNSVSSPAIAEDRIEGLKGELRKMLIGAMDKPSQKLNLIDQIQRLGIAYHFEIEIYQQLEQIHKSYFELHDGDNDNDLHTIALLFRLLRQQGYAISCVRHALKQPIHKGIPRLEARRYISLYQEEPLHNEVLLSFTKLDFNLLQEQHQKELGNLTRWWKDLDVERKFPFARDRLVEMYLWMSGVYFELEYEATREILTKVFSIVTIIDDIYDVYGTLEELELFTEAIERWDVEAKDGLPEYMQACYKIVLDLYDEIGYEVTRKGRSDCLFYAKEAMKNQVRAYFTEAKWFHQNHIPTMEEYMPIALPTTAVELLLVMLLLGMGDTVTKDVFDWLLYSNPKMVNAVKVVCRLMDDIAGHKFEQERGHGPSSVECFMKQYGVTEEEAKEELHNQVANAWKDINEGLCCSTNVSRQLLVRILNFTRVVHVVYRDEIDLYTHAGTKLKEHVTNLYVNPLPM

>EgranTPS008

DSSHVPERRSANFHPSIWRDYFLKYASDSNSMDDRIEKLKGEVRKMLIDAIDKPSQKLNLIDQIQHVGIADHFEIEINQQLEQIHESYFNFHSGDKDSDLHTTALLFRLLRQQGYTISCEIFNKFKDNNGNFNESLIADVQGLLSLFEACHTRFHGDDVLNDALAFTMTHLKSIDEGKASPNLKKQVSHALNQPIHKGIPRLEARQYIPLYQEKPSHNEVLLALAKLDFNLLQEQHQKELGNLTRWWKNLDIERKFPFARDRLVEMYLWMSIVYFESDYEAAREILTKVASMVSIIDDIYDVNGTLEELGLFTEAIERWDINAKEGLPEYMQACYKTVLDLYDEIGYEVTSKGQSYQLFYAKEAMKNLVRAYFAEAKWFHLNHVPTMEEYMPIALTSAAVELLLVTSLLGMEDFVTKDAFDWLLYGNSKMVKAVKLVGRLMDDIAGHFEQERGHGPSSVECFMKQYKVTEEEAKVELRKQVADAWKDINEGLCCPAIVPRPLLVRILNFVRAMHVMYKDEIDIYTHAGTKLKEYVTSLYVNPLPM

>EgranTPS009

MSLPIMAISSSSPAQETSHVPERRSANFPPSTWRDHFLKYASDSNSDDRIEKLKGEVRKMLTDAIDKPSQKLNLIDQIQRVGIAYHFEIEINQQLEQIHDSYFNFHNSNKDSHLHTIALLFRLLRQQGYTISCEIFNKFKDSNGNFSESLIADVQGLLSLFEACHTRFHGDDILNDALAFTMTHLKSIDEGKASPNLKKQVSHALNQPIHKGIPRLEARHYIPLYQEEPSHNEVLLALAKLDFNLLQEQHQKELGNLTRWWKNLDVERKFPFARDRLVEMYLWMSIIYFESDYEAARKILTKVASMVSIIDDIYDVHGTLEELRLFTEAIERWDIKAKEGLPEYMQACYKTVLDLYDEIGYEVTRKGRSYRLFYAKEAMKNQVRAYFAEAKWFHQNYVPTMEEYMPIALATAAIELLLVTLLLGMEDFVTKDAFDWLLYGNSKMVKAVKLVGRLMDDIAGHFEQERGHGPSSVECFMKQYEVTEEEAKEELRKQVADAWKDINEGLRCPTIVPRPLLVRILNFARAMHVVYKDEIDIYTHAGTKLEEHVTSLYVNPLPM

>EgranTPS010

MSLPIMAISSSSPAQDSSHVPERRSANFHPSIWRDYFLKYASDSNSMDDRIEKLKGEVRKMLTDAMDKPSQKLNLIDQIQRVGIAYHFKIEINQQLEQIHESCFNFHNGDKDSHLHTTALLFRLLRQEGYTISCEIFNKFKDSNGNFNESLIADVQGLLSLFEACHTRFHGDDVLNDALAFTMTHLKSIDEGKASPNLKKQVSHALNQPIHKGIPRLEARQYIPLYQEKPSHNEVLLALAKLDFNLLQEQHQKELGNLTRWWKNLDIERKFPFARDRLVEMYLWMSIVYFESDYEAAREILTKVASMVSIIDDIYDVNGTLEELGLFTEAIERWDINAKEGLPEYMQACYKTVLDFYDEIGYEVTSKGQSYRLFYAKEAMKNLVRAYFAEAKWFHLNHVPTMEEYMPIALTSAAVELLLVTSLLGMEDFVTKDAFDWLLYGDSKMVKAVKLVGRLMDDIAGHFEQERGHGPSSVECFMKQYKVTEKEAKVELRKQVANAWKDINEGLCCTAIVPRPLLVRILNFVRAMHVMYKDEIDIYTHAGTKFKDYVTSLYVNPLPM

>EgranTPS011

MSVQVPAIPSSSPSKGRSGGVERPLAEFHASVWGDHFIKYASPSYSTKFKFLGRVEEQVEELKGEVRKMVINAVDKPSQMLHLIDQIQRLGIDYHFEQEIDAQLERIHKSYSQLDHGDFKGDDLHMVALMFRLLRQQGFNISSEVFNNFKDNEGNFKKSLITDVRGLLSLYEACHLRCHGDAILEEALPFAITHLESIDERKVSTSLAKQVSHALKQPLCKGLPRFEARHYIIFYQEEPSHDEVLLTMAKLDFNLLQEQHQKELGAITRWWKNIDVARKFPFARDKIAEMFFWMVGVYFQPEFAVARNILTRVTALISILDDIYDAYGTLEELVPYTEAIEKWDVDAMDGLPEYMQAHYKEILNLYDEIGSDLATKGRSYRLTYAKEAMKKQAKWYFHEAKWFHTGYTPTLEEYIPLALLTTGYEALSITSLVGMGDVVTRDAFEWLLGDCKILRASQIICRFMDDISSHKFEQKRGHVASSVELFMKENHASEQEAEEELQKRVVDAWKDINEEFLRPITAPMPVLTAILNLSRVMDLLYSNGGDHYTHSKTELKEHITSLFVSPLPI

>EgranTPS012

MSVQILTIPSSSPSKGRSGGVVRWPNFMRAYGAITSSNTLPPATQWLEEQVEELKGEVRKMVINAVDKPSQMLHLIDQIQRLGIDYHFEQEIDAQLERIHKSYSQLDHGDFKGDDLHMVALMFRLLRQQGFNISSEVFNNFKDNEGNFKKSLITDVRGLLSLYEACHLRCHGDAILEEALPFAITHLESIDERKVSTSLAKQVSHALKQPLRKGLPRLEARHYIIFYQEEPSHDEVLLTMAKLDFNLLQEQHQKELGVITRWWKNIDVARKFPFARDRIAEMFFWMVGVYFQPEFAMARNILTRVTALISILDDIYDAYGTLEELVPYTEAIEKWDADAMDGLPEYMQAHYKEILNLYDEIGNDLATKGRSYRLTYAKEAMKKQAKWYFHKAKWFHTGYTPTLEEYIPLALLTTGYEALSITSLVGMGDVVTRYAFEWLLGDCKILRASQIICRFMDDISSHKFEQKRGHVASSVELFMKENHASEQEAEEELQKRVVDAWKDINEEFVRPTTAPMPVLTAIINLSRVMDLLYNNRGDHYTHSKTELKEHITSLFVSPLPI

>EgranTPS013

MSFQVSANPSSSPSKGRSGGIERPLAEYHESVWGDHFIKYASPSYSTKFKFLGRVEEQVEELKGEVRKMVTNAMYKPSQMLHLIDQIQRLGIDYHFEREIDEQLEGIHKSYSQLDHGDFKGDDLHMVALMFRLLRQQGFNISSEVFNNFKDNEGNFKKSLITDVRGLLSLYEACHLRCHGDAILEEALPFAITHLESIDEMKVSTSLAKQVSHALKHPLRKGLPRLEAMHYIILYQEKPSHDEVLLTLAKLDFNLLQEQHQKELGGITRWWKNIDVARKFPFARDRIAELFFWMVGAYFQPEFAMARNILTRVTGLISILDDIYDAYGTLEELVPYTEAIEKWDVDAMDGLPEYMQAHYKELLNLYDEIGNDLATKGRSYRLAYAKEAMKKQAKGYFHEAKWFHSGYTPTLEEYMPLALLTTGYEALSITALVGMGDVVTRDAFEWLLSDCKILRASQIICRFMDDISSHKFEQKRGHVASSVELLMKENRISEQEAEEELQKRVVDAWKDINEEFLHPTAGPISVLTLILNLSRVIDVLYTKGDHYTHSKTKLKEHITSLFVSPLPI

>EgranTPS014

MSFQVSANPSSSPSKGRSGGVERPLAEYHESVWGDHFIKYASPSYSTEEQVEELKGEVRKMVTNAVDKPSQMLHLIDQIQRLGIDYHFEREIDEQLEGIQKSYSQLDHGDFKGDDLHMVALMFQLMRQQGFNISSEVFNNFKDNEGNFKKSLITDVPGLLSLYEACHLRCHGDAILEEALPFAITHLESIDEMKVSTSLAKQVSHALKHPLRKGLPRLEAMHYIILYQEKPSHDEVLLTLAKLDFNLLQEQHQKELGGITRWWKNIDVARKFPLARDRIAELFFWMVGAYFQPEFAMARNILTRVTGLISILDDIYDAYGTLEELGPYTEAIEKWDVDAMDGLPYMQAHYKELLNLYDEIGNDLATKGRSYRLAYAKEAMKKQAKGYFHEAKWFHSGYTPTLEEYMPLALLTTGYEALSITALVGMGDVVTRDAFEWLLSDCKILRASQIICRFMDDISSHKFEQKRGHVASSVELLMKENRISEQEVEEELQKRVVDASNRRPNVGSHLDSQSLTGDRCVIHQGRSLHPLQDAQRAYHITLCSPADL

>EgranTPS015

MSVQVSAIPSSSPSKGRSGGVERRLAEYHESVWGDHFIKYASPSYSTEEQVEELKGEVRKMVTNAMDKPSQMLHLIDQIQRLGIDYHFEREIDEQLERIHKSYSQLDHGDFKGDDLHMVALMFLLRQQGFNISSEVFNNFKDNEGNFKKSLIIDVRGLLSLYEACHLRCHGDAILEEALPFAITHLESIDERKVSTSLAKQVSHTLKQPLRKGLPRLEARHYIILYQEKPSHDEVLLTLAKLDFNLLQEQHQKELGGITRWWKNIDVARKFPFARDRIVELFFWMVGAYFQPEFAMARNILTRVTGLISILDDIYDAYGTLEELVTYTEAIEKWNVDAMDGLPEYMHEIGNDLATKGRSYRLAYAKEAVSIVGTFYMHPMKKQAKGYFHEAKWFHSSYTPTLEEYMPLALLTTGYEALSITALVGMGDVITRDAFEWLLSDCKILRASQFICRLWMTLVLTRFEQKRGHVASSVELLMKENCISEQDAEEELQKRVVDAWKDINEEFLRPTVGPMPVLTLILNLSRVIDVLYTNGDHYTHSKTKLKEHIASLFVNPLPI

>EgranTPS016

MSVPVSAIPSSSPNKESSRVVERRRADYHPSIWGDYFLVYASPTNSMEFKYVGRVEEQIEGLKGEVRKMLTNVVNKPSQVLQLIDQIQRLGIFYHFKREIDEQLEQIHKSYSQLVHGDFKGDDLHMIALIFRLLRQQGYNVSSVVFNKFKNSERNFRESLITDVRGLLSLYEACHLRCHGDSILEEALPFAITHLESINESKVSTSLAKQVKHALRQPLRKGLPRLEARYYVPLYQEEPSHDQVLLALAKLDFNLLQEQHQKELGNITRWWKDIDVATKFPFARDRIVELFFWISGAYFEPEFAVARDILTKVTALTSILDDMYDVYGTLEELVILTEAIEKWDVDAMDGLPDYMQAWYKVLLDVYDAVGNEVATKERSYRLTYVKEAMKKQARVYFHEAKWFHTNYTPTLEEYMPLALLTSGYEMLAITSLVGMGDVVTKHAFEWLLGDCKILKASQIICRLMDDIASHQFEQKRGHIASSVELFMKEHNVSEQETEKELRKQVVDAWKDINEAFLRPTAVPMPILMRILNISRVIHVLYSDGDNYTHSGTLLKDHVTSLFISPLPVSHLSRGAQEGTRQDYGGR

>EgranTPS017

MAAPVSAIPSSSPNKGSSRVVERRWADYHPSIWGDYFLAYASPTNSVELKYVGRVEEQIEGLKGEVRKMLTDVVNKPSQVLHLIDQIQRLGIFYHFKREIDEQLEQIHKSYSRLVYRDFKGDHLHMIALIFRLLRQQGYNVSSEVFNKFKDGEGNFRESLITDVQGLLSLYEACHLRCHGDSILEEALLFAITHLESLNESKVSTSLAKQVKHALRQPLHKGLPRLEARRYVPLYQEEPSHDQVLLALAKLDFNLLQEQHQKELGNITRWWKDIDVARKFPFARDRIVELFFWISGAYFEPKFAVARDILTKVIALTSILDDMYDVYGTLEELVILTEAIEKWDVDAMDGLPEYMQAWYKVLLDVYDAVGNEVATKERSYRLTYAKEAMKKQARVYFHEAKWFHTNYTPTLEEYMPLALLTTGYEMLAITSLVGMGDVVTKHAFEWLLGDCKILKASQIICRLMDDIASHQFEQKRGHVASSVELFMKEHDVSEQETEKELHKRVVDAWKDINEAFLRPTAVPMPILMRILNLSRVIHVLYSDGDNYTHSGALLKDHVTSLFISPLPVSHLSRGAQEGTRQDYGGPCPQIDGMHFFRPAANEDAMRKKQEKGKQAMERL

>EgranTPS018

MSVPVSAIPSSSPNKESSRVVERRRADYHPSIWGDYFLVYASPTNSMEFKYVGRVEEQIEGLKGEVRKMLTDAVNKPSQVLHLIDQIERLGIFYHFKREIDEQLEQIHKSYSQLVHGDFKGDDLHMIALIFRLLRQQGYNVSSVVFNKFKNSEGNFRESLITDVRGLLSLYEACHLRCHGDSILEEALPFAITHLESINESKVSTSLAKQVKRALRQPLRKGLPRLEARYYVPFYQEEPSHDQVLLALAKLDFNLLQEQHQKELGNITRWWKDIDVATKFPFARDRIVELFFWISGAYFEPEFAVARDILTKVIALTSILDDMYDVYGTLEELVILTEAIEKWDVDAMDRLPKYMQAWYKVLLNVYDAIGNEVATKGRSYRFTYAKEAMKKQARVFFHEAKWFHTNYTPTLEEYMPLALLTTGYEMLAITSLVGMGDVVTKHAFEWLLGDCKILKASQIICRLMDDIASHQFEQKRGHVASSVELFMKEHNVSEQETEKELRKQVVDAWKDINEALLRPTAVPMPILMRTLNLSRVIHVLYSDGDNYTHSGTSLKDHVTSLFISPLPVSHLSRGAQEGTRQDYG

>EgranTPS019

MHQRSIQKKGDMSVPVSAIPSSSPNKGSSRVVERRSADYHPSIWGDYFLVYASPTNSMELKYVGRAEEQIEGLKGEVRKMLTDAVNKPSQVLPLIDQIERLGIFYHFKREIDEQLEQIHKSYSQLVHGDFKGDDLHMIALIFRLLRQRGYNVSSVVFNKFKNSEGNFRESLITDVRGLLSLYEACHLRCHGDSILEEALPFAITHLESINESKVSTSLAKQVKRALRQPLHKGLPRLEARYYVPLYQEEPSHDQVLLALAKLDFNLLQEQHQKELGNITRWWKDIDVATKFPFARDRIVELFFWISGAYFEPEFAVARDIFTKVIALTSILDDMYDIYGTLEELVIFTEAIEKWDVDAMDGLPEYMQAWYKVLLNVYDAIGNEVATKGRSYRLTYAKEAMKKQARVYFHEAKWFHTNYTPTLEEYMPLALLTTGYEMLAITSLVGMGDVVTKHAFEWLLGDCKILKASQIICRLMDDIASHQFEQKRGHVASSVELFMKEHNVSEQEAEKELRKRVVDAWKDINEAFLRPTVVPLPILMRTLNLSRVIHVLYSDGDNYTHSGTSLKDHVTSLFISPLPVSHLSRGAQEGTQQDYGGK

>EgranTPS020

MLSINNTLQLLIFCNADEGQIEELKREVNKMLTDVVDKPLQKLHLIDQIQRLGIEYHFECEVDEHLEQIHKSYSRLDHEDFKVDDLHTVALIFRLLRQHGYNISSEVFDKFKDSKGNFRESLISDVQGLLSLYEACHLRCHGDSILDEALPFSTTHLESINESKVSTSLAKQVSHALKQPLRKGLPRLEASHYIPLYQEEPSHDEVLLTLAKLDFNLLQEQHQKELGKITRWWKNLDVPRKFPFARDRIVELFFWTTGIYFEPEFATAREILTKVISLTSIMDDIYDVYGTLEELALLNEAIQKWDFDAMDGLPEYMQAYFKEFLQLYEYIGNQLAAKGKSYRLIYAKEVMKKLVGAYFQEAKWFHTNYIPTLEEYMPLQLITTGYGMLSTTSLIGMGDVVTEHVLKWSVGDCKSVKATQSICRLMDDVSSHEFEQKRGHVVSAVELLMKYRGISEQEAAEELHKGVIDAWKDTNEEFLRPTAVPMSVLTRMLNFSRVIDVLYSDGDNYTHSKTKLKDYVTSLFINPLSM

>EgranTPS021

MSLPISTIPSSLATQDKSHVVERHSANFHPSIWGDYFLKYASCSCSMENGHDQHAEQEIQKLDDEVKRMLCADADKPSLKLDMIDQIQRLGIAHHFASDIDHVLKQLSETCFACNNGDRDIDDLYTAALLFRLLRQQGYRVSSDIFNKFKDPSGKFSEKHASDVRGLLSLYEASHLSVHGEDVLDQALSFSLTHLESVKEQLSPPLATQVRHALKQTIRKGVPRLEARQYISMYEAEPLHNEVLLSLAKLDFNRLQKQHQKELFDITRWWMGLDFKRKLPFARDRLVEGYFWIVGVHFEPELAVARRMMTKVIAVTSVLDDIYDVYGTYEELELFTQAIQRWDIDCIHELPEYMQVFYKALINIYVEIEEILACTGKSYCLCYAVEAMKRQARSYFAEAKWLHQQHKPTMDEYMSIALVSSGYPLLAVTSFVGMPDIVTKDDLDWLFNDPKILKASTIICRLMDDLATHKFEQSRGHVDSAVQCYMKQYGVTEQEAENNLRKQVNDSWKDINEECLRPTAVAMPLLVGILNLSRVMDVLYKDGGDHYTNPHIALKDYIHSVLIDPVQ

>EgranTPS022

PDLQVSTIPRSSPNIGTNHVIERRSAGYHPSIWGDYFLKYAFPSNSVRLLRQQGYNVSSEIFNKFKDSEGNFQESLVTDARGLLSLYEACHLRCHGDSILDEALPFATTHLESIDESKVSTSLAKQVSHALEQPLRKGLPRLEARRYIPLYQEEPSHDEVLLALAKLDFDLLQEQHQKELGEITRWWKEIDVPRKFPFARDRIVELFFWISGIYFEPEFAMARNILTRVISLTSILDDIYDVYGTLEELALLTEAIQKWDVDAMDGLPVYMQAYYKELLHLYEYIGNELATKERSYRLVYAKEVMKKLARAYFQEAKWFHTNYIPTLEEYMSLQLITTGYGMLATTSLVGMGDVVTKHALEWSVSDCKIVKAAQTISRLMDDIASHQFEQKRGHVVSAMELLMKYHGVSEQEAGEELQKGVIDSWKDINEEFLRPTAVPMPILTRMLNFSRVMDVLYSDGDNYTHSETKLKDYVMLLFVSPLLM

>EgranTPS023

MSLQISRVPSSSPAEKTSQVPEGRSEIFHLTIWGDYFLKYASDSNLTVAFKLLRLLRQQGYNVSCEIFNRFKDSEGNFNKSSIADVQGMLSLFEACHLSYHGDDILNDALAFTISHLESIEKKKVSPNLVKQVSHALHQPIQKGLPRLEARRYIQFYQEEPLHNEVLLSLAKLDFNSLQEQHQKELGNLTRWWKDINIEEFPFARDRLGELYVWMLGIYFEPEYEIARGIVTKMMVILSILDDIYDVYGTLEELELFTEAIERWDVDAKEGLPKCMQVFYKTLLDFYDEISNELARKGRSYRLFYAKEVMKIQVRAYLAEAKWFHHSHVPTMEEYMPIALISIGTQLTFVTAFLGMGDIVTKDAFDWLLSSDPRIVKASQVIGRLMNDIAGHFEQERGHVASSVECFMKQYSVTEEEAKKELCKQVANAWKDINEELRRPTAVPMVLLMRIINLARATHAVYEDETDHYVNAGTNFKEFVTLLLVNPCQCDGSEEHG

>EgranTPS024

VLFRRSPARTCRLVTGACAAPAGHSQRFGRVAPTRSGLWPRVWARVASSGDARHVAGLEGQIEELKGEVNKMLTDVVDKPLQKLHLINQIQRLGIEYHFEREVDEQLEQIHKSYSRLDHEDFKVDDLHTIALIFRLLRQHGYYISSEVFYKFKDSEGNFRESLISDVCGLLSLYEACHLRCHGDSILDEALPFATTHLESINESKVSTSLVKQVSHALKQPLRMGLPRLEASRYIPLYQEEPSHDEVLLTLAKLNFNLLQEQHQKELGKITMWWKNIDVPRNFPFTRDRIVELLFTTGVYFEPEFVMAREILTKVISLTSIMDDIYDVYGTLEELALLNEAIQKWDVDAMDGLPENIQAYFKEFLQLYKYIGNQLAAKGRSYRLIYAKEVMKKLVRAYYQEAKWFHTNIPTLEEYMSLQLITTGYGMLVTLVRMGDVVTEHVLKWSVGDYKIMKAAQTICRLMDDVSSHEFEQKRGHVVSIVELLMKYHGISKQEAGEELLKRVIDAWKDINEEFLCLTIVPMSVLMRILNFSRVVDVLYSDGDNYTHSKTKLKNYVTSLFVNPLPM

>EgranTPS025

MTCQGLHFVDLLRFPSVPPHLLYGNNRPHINTCSLEGQIEELKGEVKKMLIDAVDKPLPKLHLIDQIQRLGIEYHFEREVDEQLEQVHKSYSRLDHEDFKVDDLHTVALIFRLLRQHGYNISLEVFDKFKDSKGNFRESLISDVQGLLSLYEACHLRCHGDSILDEALPFATTHLESINESKVSTSLAKQVSHALKQPLRKGLPRLEASCYIPLYQEEHSHDEVLLTLAKLDFNLLQEQHQKELGKITRWWKNIDVPRKFPFARDRIVELFFWTTGIYFEPEFAMAREILTKVISLTSIMDDIYDVYGTLEELALLNEAIQKWDFDAMDGLPKYMQAYFKEFLQLYEYIGNQLAAKGKSHRLIYAKEVMKKLVGAYFQEAKWFHTNYIPTLEEYMPLQLITTGYGMLSTTSLIGMGDVVTEHVLKWSVGDCKSVKATQTICRLMDDVSSHEFEQKRGHVVSAVELLMKYRGISEQEAAEELHKGVIDAWKDTNEEFLRPTAVPMSVLTRMLNFSRVIDVLYSDGDNYTHSNTKLKDYVTSLFINPLPM

>EgranTPS026

MSQVSATPCAPSNKGTGHVIERRSAGYHPSVWGDYFLKYDSPSNSVKFKFLGRVEGQIEELKGEVKKMLTDIMDKPLQKLHLIDQIQRLGIEYHFEREIDEQLEQIHKSYSRLDHEDFKVDDLHIVALIFRLLRQHGYNISSEVFDKFKDSGGNFRESLISNVLGLLSLYEACHLRCHGDSILDEALLFATTHLESINESKVSTNLAKQVSHALKQPLRKGLPRLEARHYIPLYQEEPSHDEVLLTLAKLDFNLLQEQHQKELGKITRWWKNIDVPRKFLFARDRLVELFFWTTGVYFEPEFAMARDILTKVISLTSIIDDVYDVYGTLEELALFNEAVQKWDVDAMDGLPEYMQALFKEFIQLYEYIGNELATKGRSYYLVYAKEVMKKLVSAYFQEAKWFHTNYIPTLEEYMSLQLITSGYEMLATTSLMGMGNVVTEHALKWSISDCKIRKAAQTIGRLMDDIVSHEFEQKRGHVVSAVELLIKYRGVSEQEAVEELQKRVIDAWKDTNEEFLRPTAVPMPILTRVLNLSRVVDVLYSDGDNYTHSETKLKDYVTSLFVNPLPM

>EgranTPS027

MSQVSATPCAPPNKGTGHVIERRSAGYHPSVWGDCFLKYDSPSNSVEGQIEKLKGEVKKMLTDIMDKPLKKLHLIDQIQRLGIEYHFEREIDEQLEQIHKSYSRLDHEDFKVDDLHIVALIFRLLRQHGYNISSEVFDKFKDSKGNFRESLISDVRGLLSLYEACHLRCHGDSILDEALPFATTHLESINESKVSANLAKQVSHALKQPLRKGLPRLEASCYIPLYQEEPSHDEVLLSLAKLDFNLLQEQHQKELAKITRWWKNIDVPRKFSFARDRIVELFFWTTGIYFEPEFAMAREILTKVISLTSIMDDIYDVYGTLEELALLNEAIQKWDFDAMDGLPEYMQAYFKEFLQLYEYIGNQLAAKGRSYRLIYAKEVMKKLVGAYFQEAKWFHTNYIPTLEEYMPLQLITTGYGMLSTTSLIGMGDVVTEHVLKWSVGDCKSVKATQTICRLMDDVSSHEFEQKRGHVVSAVELLMKYRGISEQEAAEELHKGVIDAWKDTNEEFLRPTAVPMSVLTRMLNFSRVIDVLYSDGDNYTHSKTKLKDYVTSLFLNPLLISL

>EgranTPS028

LDSQVSAILRSSPNKGTNHVIERRSAGYHPSIWGITSLNMLSTPEEQIKKLKGKVRKMLAGVMDKPSQMLHLIDQIQRLGIDYHFEHEVDEQLEQINKSYSQLHLEDFKVDDLHMAALIFQLLQQQGYNVSSEIFNKFKDSEGNFRESLVTDARGLLSLYEACQLRCRGDSILDEALPFATTHLESIDERKMSTSLLKQVSHALEQPLRKGLPRLEARHYISLYQVELSHDEVLFTLAKLDFNLLQEQHQKELGKITRWWKEIDVPRKFPFARDRIVELFFISGLYFELEFVMAKNILTKVISLTSILNDIYDVYGTLEELALLTEAIKWDVDAMDGLPVYMQAYYKELLQLYEYIGNELATKERSYRLVYAKEVMKKLARAYFQEAKWFHTNYVPTLEEYMSLQLITTSYGMLATTSLVRMGDVVTEHALEWSIGDCKIVKTAQIICRLMDDIASHQFEQKRGHVVSAVELLMKYHGVLEQEAGEELQKGVIDAWKDINEEFLCLTAVLMPILTRMLNFSWVMDVLYSDGDNYTHSETKLKYYVTLLFVSPLPM

>EgranTPS029

MLKFIYKINRTCRFQQFHVLSPNKGTYHVIERRSAGYHPSIWGDFFLKYAFPSSLEEQIEELKGKVRKMLAGAVDKPSQMLHLIDQIQHLGIDYHFEHEVVEQLEQIHKSYSQLHLEDFKVDDLHMVALIFQLLQQQGYNVSSEIFNKFKDSEGNFRESLVTNARGLLSLYEACHLRCHSDSILDEALPFATTHLESIDESKVSTSLAKQVSHALEQPLRKGLSRLEARHYIPLYQEEPSHDEVLLALAKLDFNLLQEQHQKELGEITRWWKEIDVPRKFPFARGRIVELFFWILGIYFEPEFAMARNILTKVISLTSILDDIYDVYGTLEELALLTEAIQKWDVDAMDGLPVYMQAYYKELLQLYEYIGNERSHLLVYAKEVMKKLVKAYFHEAKWFHTNYVPTLEEDMSLQLITTGYGMLATTSLVGMIDVVTEHALEWSVGDCKIVKVAQTICRLMDDIASHQAKEGHVLSAVELLMKYHGILEQEAGEELQKGVTDAWKDINEEFLHPTAVPMPILTQMLNFPRVIDVLYSDGDNYTHSETKLKDYVTSLFISPLLM

>EgranTPS030

MSLLVKTITLSFCFLPIVELHIMEWRHCVTVWSWCGFGPLTSMFPFFSEERIERLKGEVRKMLTSAMDKPSQKLNLIDQIQHFGLAYHFEFEIVEQLEQIHRSYFEFHCGDNDDNLHTIALLFRLLRQQGYNVSCKIFNRFKDSEGNFSKSVIANVQGLLSLFEACHLSYCSDDILNDALTFTITAKLDFNSLQGQHRKELGNLTRWWKDIDIEREFPFARDRLVELYTWMLGVHFEPEYEIARGFMTKMNVFLTIIDDIYDVYGTLEELELFTAIERWDVDAKEGLPECMQVIYKILLDFYDEIGYELIRKGRSHHLFYAKEAMKIQVRAYLAEAKWFHHSHVPMMEEYMPIALTTIGIQMALVASFLGMGDTVTKDVFDWLLSSDTKIVKALRVIGRLMNDIAGHFVRRFYQRKNNFDDIYIFCSLSKREVMWKMSKRLIVRNMTRS

>EgranTPS031

MSVRISRVPSSYPAEKTSLVPEGRSAIFHPTIWADYFLKHASNSNPTEEHIERLKEVRKMLMGAMDKPSQKLNLIDQIQRLGFAYHFEHEINERLEQIYRSYFEFHYGDNDDNLHTVAILFLLQQQGYNVSCIFNRFKDSEGNFNKSSIADVQGMLSLFEACHLSYHGDDILNDALAFIISHLESIEKKKVSPNLVKQVSHALHQPIQKGLLRLEARCYIQFYQEEPSHNEVLLSLAKLDFNSLQEQHQKELGNLTRWWKDIDIEREFPFARDRLGELYVWMLGIYFEPVYKIARGIVTKMMVILSILDDIYDVYGTLEELELFTEAIERWDVDAKEGLPKCMQVFYKTLLDFYDEIGNELARKGRSYCLFYAKEVMKIQVRAYLAEAKWFHHSHVPTMEEYMPIALISIGSQLTFVTAFLGMGDIVTKDAFDWLLSSDPRIVKASQVIGRLMNDIAGHFEQERGHVASSVECFMKQYSVTEEEAVGEIFLKYFEVVKTFPSV

>EgranTPS032

MSLPISGVPFPSPAEETSPVAERRSAIFHPTIWTDYFLKYASDSTSTSSEGIVEEQIKRLKGEVRKMLTGAMDKPSQKLNLIDRIQRLGLADHFEHEIDEQLEQIHRSYFAFHCEDNNNNLHTIALLFRLLRQQGYNISCEIFNRFKDNEGNFSKSIIADVQGLLSLYEACHLSYHGEDILNDALTFTITHLESIDKRKSEPNLEKQVSHALHQPIQKGLPRLEARRYIQFYQEEPSHNEVLLSLAKLDFNSLQEQHRKELGNLARWWKDIDIEREFPFARDRLGELYIWNLGVHFEPEYEISRGILTKMMAILTILDDIYDVYGTIEELELFTEAIERWDLDAKEGLPECMQVIYKILLNFYDEIGYELTRKGRSYCLFYAKEAMKIQVRAYLAEAKWFHHSHVPTMEEYLPIALTSIGIQMALVASFLGMGDTVTKDVFDWLLSSDTKIVKALRVIGRLMNDIAGHKFEQERGHVASSVECFMKQYKVTEEEAKKELCKQVADAWKDINEELCRPTAVPRVLLMRIINLAGAIHAVYEDETDNFVNAGTNFKEFVTCLLVNPCQCDVLKDEV

>EgranTPS033

MSLQISAIPSRPSAKAASPVAERRSASYHPSIWGDYFLRYASDSCSTVATLLIGLNQPCCFCNIRALAFRVNSDSEEQIEELKEEVRKMVAAIVPTSQKLELIDQIQRLGIAYHFQGVIEEQLEQIHESYFELNDGDHDHNDLHMVALLFRLLRQRGYGISCEMFEKFKDSDGNFRESLTADILGILSLYEACHLRVHGEDVLDGALSFTVTHLESIDKNQVSPTLAKQVSQALKQPIYKGLPRLEAMQYIPIYQEEPSHNEVLLSLAKLDFNLMQEQHQKELGHIARWWKELDVARNFPFARDRVVECYFWILGVYFEPEFVLARKFMTKVIAMTSIIDDIYDVYGTLEELELFTGAMERWSIDAIDGLPKYMQVCYKALLDVYDDIEKAIAENGTSYGLYHAKEAMKNLVRAFTEAKWFHQGHVPTMEEYMAVALVTSAYEMLATTSFVGMGDLATQDAFDWLLSGPKMVKASTTICRLMDDIVSHFEQKRGHVASAVECFVHQHGVTEQEAKEELWRRVVEAWKDVNEECLAPTAIPARLLTLILNLTRVIDVLYTDEDNYTNAGTKLKNYVASLLIYPLPM

>EgranTPS034

MSPQISAMPSPSPALETSHVAERRSGNFHPSIWGDYFLKYASDSGERIERLKGEVKKMLTSAMDKLSQKLNLIDQIQRLGLAYHFEIEIDKELEQIHRSYFEYHRGDNDEDLHTTALLFRLLRQQGYDVSCEIFNKFKDNEGHFSKSLIADVRGLLSLFEACHVGFHSDDILNDALAFTVTHLESIDKEKVSRNLEKEVSHALSQPIHKGLSRLEARHYIQLYQEEPLHNEVLLSLARLDFNLLQKQHQKELGNITRWWKDLDGERKFPFARDRLVELYFWMSGVYFEPKYEATREILTKMIVIVSIFDDMYDMYATLEEVEVFTEAIERWDVNAKDGLPKYMQVCYETLLDLYDEFGDKFTRKGQSYSLFYAKEVMKNHLKAYFAEAKWFHQNHMPTMEEYMPIASTSIGCELLLGTSFLGMGDIVTKNDFDWLLYSDSKMVKASKVVARLMDDIAGHSEQERGHSPSSVECFMKQYRVTEEEAKEELRKQVVNAWKDMNEELRRSSAVPKLLRTRILNFAQVFDVVYNDEKDHYSHAGTKFKEHVTSLYVDPLPM

>EgranTPS035

PNLQVSAIPHSSPNKGTDHVIERRSAGYHPSIWGDYFLKYAFPSNSVVSNSSVIFYFCFLAIAFCRLTSISEEQIEELKGEVRKMLAGIVDKPSQMLHLIDQIQRLGIDYHFEHEVDEQLEQIHKNYSQLHLEDFKVDDLHTVALIFRLLRQQGYNVSSEIFNKFKDSEGNLWESLVTDARGLLSLYEACHLRCHGDSILDEALPFATTHLESIDESKVSTSLAKQVSHALEQPLRKGLPRLEARRYIPLYQEEPSHDEVLLTLAKLDFNLLQEQHQKELGEITRWWKEIDVPRKFPFARDRIVELFFWTSGIYFKPEFAMARNILTKVISLTSILDDIYDVYGTLEELALLTEAIQKWDVDAMDGLPMYMQAYYKELLQLYEYIGNELATKERSYRLVYAKEVMKKLARAYFQEAKWFHTNYVPALEEYMSLQLITTGYGMLATTSLVGMGDVVTEHALEWSVGDCKIVKAAQTICRLMDDIVSHQFEQKRGHVVSAVELLMKYHGVSEQEAGEELQKGVIDAWKDTNEEFLRPTMVPMPILTRMLNFSRVMDVLYSDGDNYTHSETKLKDYVTLLFISPSLM

>EgranTPS036

MSLPVSTIPSPSPAQETSQVAERRSGNFHPSIWGDYFLKYASDSNSMSSQGVAKERIEKLKGEARKMLTSAMDKPSQKLNLIDQIQRLGLAYHFEIEIDEQLEQIHRSYFEFHCGDNDSNLHTTALLFRLLRQHGYNIPCEIFNKFKDNEGNFSKSLIADVQGLLSLFEACYLGFHGDVILNDALAFTITLLESIDKGKVTGNLEKQVTHALNQPIHKGLPRVEARHYIQLYQEEPSHNEVLLSLAKLDFNLLQEQHQKELGNITRWWKDLDGERKFPFARNRLVELYFWMSGVYFEPEYEATREILTKVMVIVSIFDDVYDIYATLEELELFTKAIERWAVDAKDGLPEYMQVCYKTLLDLYDEIGYEVTRKGQSYCLFYAKEVMKNHMRTYLAQAKWFQQNYVPTMEEYLPIASLSIGCELLLGTSFLGMGDVVTKSYFDWLLLSDNKMVKASDVISRLMNDIAGHKFEQERGHTASSVECFMKQYKVTEQEAKDALRKQVINAWKDMNKELCHPTSVPKPLLVRILNFARVFHVVYNDEVDHYSHAGTKLKEFVTSLLVDPLPM

>EgranTPS037

MSLPVSTIPSPSPAHETSQVAERRSGNFHPSIWGITFSNMLLTPTQCSQGVAKERIEKLKGEVRKMLTSAMDKPSQKLNLIDQIQRLGLAYHFEIEIDEQLEQIHRSYFEFHCGDNDNNLHTTALLFRLFIFSHAPFRCIEIFNKFKDNEENFSKSLIANVQGLLSLFEACHLGFHGDVILNDALAFTITLLESIDKGKVTGNLEKQVTHALNQPIHKGLPRVEARHYIQLYQEEPSHNEVLLSLAKLDFNLLQEQHQKELGNITRWWKDLDGERKFPFARNRLVELYFWMSGVYFEPEYEATREILTKVMVIVSIFDDVYDIYATLEELELFTKAIERWAVDAKDGLPEYMQVCYETLLDLYDEIGYEVTRKDNHTPLLCKRVMKNHMRTYLAQAKWFQQNYVPTMEEYLPIASLSIGCELLLGTSFLGMGDVVTKSYFDWLLLSDNKMVKASAVISRLMNDIAGHKFEQERGHTASSVECFMKQYKVTEQEAKDALRKQVINAWKDMNKELCHPTSVPKPLLVRILNFARVFHVVYNDEVDHYSHAGTKLKEFVTSLLVDPLPM

>EgranTPS038

MSLETSANRPLPAKENSQMVERRSVDYHPSLWEDYLAKYSSPSNSMDDGHDQQAEQEIQKLKDEVKRMLCAHADKPSLKLDMIDQIQRLGIAYHFASEIDNVLKKLSQTYFVSNNGNYDNDDLYTVALLFRLLRQQGCRISCDIFNKFKDTSGKFTEKHASDVRGLLSLYEASHLSVHGEDVLDQALSFSLKHLESIDKEQLSPPLAAQVQHALKQTIRRGVPRLEARRYISMYEAEPLHNKVLLSLAKLDFNHLQKQHQEELFDLARWWMGLDFKSKLPFARDRLVEGYFWILGVHFEPELAPVRRMMTKVIAMTSVLDDIYDVYGTYEELELFTRAVQRWDIDCINELPEYMQVFYKALIDVYVEIGEKLACTGRSYGLDYAKEAMKRQARSYFAEAKWLHQQHKPTMDEYMSVALVSSGYPLLAITSFVGMQDIVTKDDLDWLFNDPKILKASTVICRLMDDLATHKFEQGREHADSAVQCYMKQYNVTEQEAENDLRKQVDDAWKDLNEECLCPTAVAKPLLMGILNLTRVMDVLYKDGGDHYTNPHIMLKDYIRSVLMDPVQTF

>EgranTPS039

DIFNKFKDTSGKFGEKHASDIRGLLSLYEASHLSVHGEDVLDQALSFSLKHLESIDKEQLSPPLAAQVQHALKQTIHRGVPRLEARQYISMYEAEPLHNKVLLSLAKLDFNHLQKQHQKELFDLARWWMSLDFKNKLPFARDRLVEGYFWILGVHFEPELALVRRMMTKVIAMTSVLDDIYDVYGTYEELELFTRAVQRWDFDCINELPEYMQVFYKALIDVYVEIGEKLASAGRSYGLDYAKEAMKRQDRSYFAEAKWLHRQHKPTMDEYMSVALVSSGYHLLAITSFVGMQDIVTKDDLDWLFNDPKILKASTVICRLMDDLATHKFEQGREHADSAVQCYMKQYNVTEQEAENDLRKQVDDAWKDLNEECLCPTAVAKPLLMGILNLTRVMDV

>EgranTPS040

MSLETSANRPLPAKGNSQMVERRSVDYHPSLWEDYLAKYSSPSNSMDDGHDQQAEQEIQKLKDEVKWMLCAHADKPSLKRDMIDQIQRLGIAYHFASEIDNVLIKLSETYFVSNNGDHDNDDLYTVALLFRLLRQQGYRISCDIFNKFKDTSGKFGEKHASDIRGLLSLYEASHLSVHGEDVLDQALSFSLKHLESIDKEQLSPPLAAQVQHALKQTIHRGVPRLEARQYISIYEAEPLHNKVLLSLAKLDFNHLQKQHQKELFDLARWWMGLDFKNKLPFARDRLVEGYFWILGVHFEPELALVRRMMTKVIAMTSVLDDIYDVYGTYEELELFTRAVQRWDIDCINELPEYMQVFYKALIDVYVEIGEKLASAGRSYGLDYAKEAMKRQDRSYFAEAKWLHRQHKPTMDEYMSVALVSSGYHLLAITSFVGMQDIVTKDDLDWLFNDPKILKASTVICRLMDDLATHKFEQGREHADSAVQCYMKQYNVTEQEAENDLRKQVDDAWKDLNEECLCPTAVAKPLLMGILNLTRVMDVLYKDGGDHYTNPHIMLKDYIRSVLMDPVQTF

>EgranTPS041

MSQVSATPCAPPNKGTGHVIERRSAGYHPSVWGDYFLKYDSPSNSVVRNPSVIFFFCFLAIARLLRQHGYNISSEVFDKFKDSKGNFRESLTSDVRGLLSLYEACHLRCHGDSILDEALPFATTHLESSNKSKVSTSLAKQVSHALKQPLRKGLPRLEASRYIPLYQEEPSHDEVLLTLAKLDFNLLQEQHQKELGKITRWWKNIDVPRKFPFARDRIVELFFWTTGIYFEPEFAMAREILTKVISLTSIMDDIYDVYGTVEELALLNEVIQKWDVDAMDGLPEYMQTYFKEFLQLYEYIGNQLAAKGRSYRLIYAKEVMKKLVRAYFQEAKWFHTNYIPTLEEYMPLQLITTGYGMLATTSLVGMGDVVTEHVLKWSVSDCKSVKATQTICRLMDDVSSHEFDIQFEQKRGHVVSAVELLMKYHGVSEQEAGEELLKGVIDAWKDTNEEFLRPTAVPMSVLTRMLNFSRVIDVLYSDGDNYTHSKTKLKDYVTLLFVNPLPM

>EgranTPS042

MSARFSVIPSSSLPQETGCVEGRRSANFHPSIWGDYFLKYASDSNSLHSHVIAEERIERLKGEVGKMLTSAMYKPAEKLNLIDQIQRLGIAYHFELEIDKELEQIRRGYFEYQCDDNDNDLDTVALLFRLLRQRGYHVSCEIFNKFKDGDGNFGKSLIADVQGLLSLFEACHLRYHGDDNLEDALAFTTTHLESVDKRKASLHLGKKVSHALNQPIHKGMSRLEARRYIPLYQEEPSHNEVLLSLAKLDFNLVQEQHRKELGNLTRWWKGLDVQRKFPFARDRLVEMYVWWLGEYYEPEHEVAREILTKLSSVISIIDDIYDVYGTWEELELFTEAIERWDVDAKDGLPEYMQECYKIVLDLYDEIGYEFSRKGHSYRLFYAKEVMKNQARAYLVEAKCFHRNHVPTMEEYMSIALPSSGVVSILAWSFLGMGDIVTKDVFDWLLFNDPKMVKASSVIGRLMDDIAGHKFEQERGHVASAVECFMKQYRVTEEEAKEELRKQVTNAWKDINEELRRPTVIPMPILVRILNLTQAMHMMYNGETDNYTHAGTKMKEHVTSLLVNPLPM

>EgranTPS043

MPSPSPALETSHVAERRSGNFHPSIWGDYFLKYASDSSSKSSQDIAGERIERLKGEVKKMLTSAMDKLLQKLNLIDQIQRLGLAYHFEIEIDKELEQIHRSYFEFHCGDNDDDLHMTALLFRLLRQQGYDVSCEIFNKFKDNEGHFSKSLIADVRGLLSLFEACHVGFHGDDILNDALAFTVTHLESIDKGKVSRNLEKEVSHALSQPIHKGLSRLEARHYIQLYQEEPLHNEVLLSLARLDFNLLQKQHQKELGNITRWWRDLDGERKFPFARDRLVELYFWMSGVYFEPKYEATREILTKMIVIVSIFDDMYDVYATLEEIEVFTEAIERWDVDAKDGLPKYMQVCYETLLDLYDEFGNKFTRKGQSYCLFYAKEVMKNHLKAYFAEAKWFHQNHMPTMEEYMPIASTSIGCELLLGTSFLGMGDIVTKNDFDWLLYSDSKMVKASKVVARLMDDIAGHKSEQERGHSPSSVECFMKQYRVTEEEAKEELRKQVVNAWKDMNEELRRSSAVPKLLRTRILNFAQVFDVVYNDEKDHYSHAGTKFKEHVTSLYVDPLPM

>EgranTPS044

MSVPVSAIPSPSPNKESSRVVERSRADYHPSIWGDYFLVYASPTNSMEFKYVGRVEEQIEGLKGEVRKMLTDAVNKPSQVLHLIDQIERLGIFYHFKREIDEQLEQIHKSYSQLVHGDFKGDDLHMIALIFRLLRQQAYNVSSVVFNKFKNSEGNFRESLITDVRGLLSLYEACHLRCHGDSILEEALPFAITHLESINESKVSTSLAKQVKRALRQPLRKGLPRLEARYYVPFYQEEPSHDQVLLALAKLDFNLLQEQHQKELSNITRWWKDIDVATKFPFARDRIVELFFWISGAYFEPEFAVARDILTKVIALTSILDDMYDVYGTLEELVILTEAIEKWDVDAMDGLPEYMQAWYKVLLDVYDAVGNEVATKERSYHLTYAKEAMKKQARVYFHEAKWFHTNYTPTLEEYMPLALLTTGYEMLVITSLVGMGDVVTKHAFEWLLGDYKILKASQIICRLMDDIASHQFEQKRGHVASSVELFMKEHDVSEQETEKELRRRVVDAWKDINEAFLRPTAVPVPILVRILNLSRVIHVLYSDGDNYTHSGTLLKDHVTSLFISPLPVSNLSRGAQEGTRQDYR

>EgranTPS045

MSLPISRVPSSSPAEKTSLVPEGGSAIFHPTIWADYFLKHASNSNSTRLLRQQGYNVPCEIFNRFKDSEGNFNKSSIADVQGMLSLFEACHLSYHGDDILNDALAFTISHLESIEKKKVSPNLVKQVSHALHQPIQKGLPRLEARRYIQFYQEEPSHNEVLLSLAKLDFNSLQEQHQKELGNLTRWWKDIDIEREFPFARDRLGELYVWMLGIYFEPVYEIARGIVTKMMVILSILDDIYDVYGTLEELELFTEAIERWDVDAKEGLPKCMQVFYKTLLDFYDEIGNELARKGRSYRLFYAKEVMKIQVRAYLAEAKWFHHSHVPTMEEYMPIALISIGSQLTFVTAFLGMGDIVTKDAFDWLLSSDPRIVKASQVIGRLMNDIAGHGVWFHVASSVECFMKQYSVTEEEAKKELCKQVANAWKDINEELCRPTAVPMILLMRIINLARATHAVYEDETDHYVNAGTNFKEFVTLLLVNPCQCDGLEEQG

>EgranTPS046

MSLQISAIRSSSQAQEKSGVVERQWANFHPSIWGDYFLKHASHSYSMEQEIQKLKDEVKRMLYADADKLSLKLDMIDQIQRLGIAHHFTTDIDHVLKQLNETCFVCNNGDRDIDDLYTAALLFRLLRQQGYRVSFDIFNKFKDPSGKFSEKHGSDVRGLLSLYEASHLSVHGEDVLDQALSFSLTHLKSIKEQLSPPLATQVGHALKQTIHRGVPRLEARWYISMYEAEPSHNEVLLSLAKLDFNRLQKQHQKELFDLARWWIGLDFKRKLPFARDRLMEGYFWIMGVHFKPELVVARRMMTKVIAVTSVLDDIYDVHGTYEELELFTQAIQRWDIDCIHELPEYMQEFYRALIDIYVEIEEILACTGKSYCLYYAVEAMKRQTRYYFAEAKWLHQQHKPTMDEYMSIALVSSGYPLLAMTSFVGMPDIVTKDDLDWLFNDPKILKASTIICRLMDDLATHKFEHSRGHVDSAVQCYMKQYGVTEQEAENNLHKQVNDAWKDIDEECLRLTAVAMPLLMGILNLSRVMDVLYKDGGDHYTNPHITLKDYIHLVLIDPVH

>EgranTPS047

MSQVSATPCAPPNKGTGHVIERRSAGYHPSVWGDYFLIYDSPSNSVEELKREVNKMLTDVVDKPLQKLHLIDQIQRLGIEYHFECEVDEQLEQIHKSYSRLDHEDFKVDDLHTVALIFRLASRYIPLYQEEPSHDEVLLTLAKLNFNLLQEQHQKELGKITRWWKNIDIPIKFPFARDRIVELFFTTGVYFEPEFAMAREILTKVISLTSIMDDIYDVYGTLEELALLNEAIQKWDVDAMDGLPEHMQAYFKEFLQLYEYIGNQLAAKGRSYRLSTQKKLMKKLVRAYYEAKWFHTNYIPTLEEYMSLQLITTGYGLLATTSLVGMDDVVTEHVLKWSVGDYKIMKAAQTICRLMDDVSSHEFEQKRGHVVSTVELLMKYHGISEQEAREELLKRVIDAWKDINEEFLHPTIVPMSVLMRILNFSRVVDVLYSDGDNYTHSKTKLKNYVTSLFVNPLPM

>EgranTPS048

MSLPLSGVPSSSPAKRTSPNAERRSAIFHPTIWTDYFLKYASDSNSMEERIKRLKGEVRKMLTSAMNKPSQKLNLIDQIQQLGLAYHFEFEIAEQLEQIHRSYFEFHCGDNDDNLHTIALLFRLLRQQGYNVSCEIFNRFKDSEGNFSKSVIANVQGLLSLFEACHLSYRSDDILNDALTFTITHLESIDKRKVSPNLEKQVSHALNHPIRKGLPRLEVRRYIQFYQEEPSHNEVLLSLAKLDFNSLQGQHRKELGNLTRWWKDIDIEREFPFARDRLVELYTWMLGVHFEPEYEIARGFMTKMNVFLTIIDDIYDVYGTLEELELFTKAIERWDVDAKEGLLECMQVIYKMLLDFYDEIGYELTRKGRSHHLFYAKEAMKIQVRAYLAEAKWFHHSHVPTMEEYLPIALTSIGIQMALVASFLGMGDTVTKDVFDWLLSSDTKIVKALRVIGRLMNDIAGHFEQERGHVASSVECFMKQYKVTEEEAKKELCKQVADAWKDINEELCRPTAVPRVLLIRIINFAGAIHAVYEAETDHFVNAGTNFKEFVTCLLVNPCQCDGLNDEV

>EgranTPS049

MSQVSATPCAPPNKGTGHVIERRSAGYHPSVWGDYFLKYDSPSNSVKFKFLGRVEGQIEELKGEVKKMLIDVVDKPLPKLHLIDQIQRLGIEYHFEREVDEQLEQIHKSYSRLDHEDFKVDDLHMVALIFRLLRQHGYNISSEIFDKFKDSKGNFRESLISDVRGLLSLYEACHLRCHGDSILDEALPFATTHLESINESKVSTSLAKQVSHALKQPLRKGLPRLEASRYIPLYQEEPSHDEVLLTLAKLDFNLLQEQHQKELGKITRWWKNIDVPRKFPFARDRIVELFFWTTGIYFEPEFAMAREILTKVISLASIMDDIYDVYGTLEELALLNEAIQKWDFDAMDGLPEYMQAYFKEFLQLYEYIGNQLAAKGRSYRLIYAKEVMKKLVGAYFQEAKWFHTNYIPTLEEYMPLQLITTGYGMLSTTSLVGMGDVVTEHVLKWSVSDCKSVKATQTICRLMDDVSSHEFEQKRGHVVSAVELLMKYRGVSEQEAAEELQKGVIDAWKDTNEEFLRPTAVPMSILTRMLNFSRVIDVLYSDGDNYTHSNTKLKDYVTSLFVNPLPK

>EgranTPS050

ISLTAEQHQKELGAITRWWKNIDVARKFPFARDRIAEMFFWMVGVYFQPEFAVARNILTRVTALISILDDIYDAYGTLEELVPYTEAIEKWDVDAMDGLPEYMQAHYKEILNLYDEIGNDLATKGRSYRLTYAKEAMKKQAKWYFHEAKWFHTGYTPTLEEYIPLALLTTGYEALSITSLVGMGDVVTRDAFEWLLVDCKILRASQIICRFMDDISSHKFEQKRGHVASSVELFMKENHASEQEAEEELQKRVVDAWKDINEEFLRPTTAPMPVLTAILNLSRVMDVLYSNGGDHYTHSKTELKEHITSLFVSPLPI

>EgranTPS051

MIIWTIPVLFYHKKSRQLVTVYDYLDYPVLFYHKKSRTCPRNSSPFRTNFKFPLLFQEIQKLDDEVKRMLCADADKPSLKLDMIDQIQRLGIAHRFASDIDHVLKQLSETCFVCNNGDRDIDDLYTAALLFRLLRQQGYRVSSEIFNKFKDPSGKFSEKHASDVRGLLSLYEASHLSVHGEDVLDQALSFSLTHLKSVKEQLSPPLATQVHHALKQTIRKGVPRLEARRYISMYEAEPLHNEVLLSLAKLDFNRLQKQHQKELFDITRWWMGLDFKRKLPFARDRLVEGYFWILGVHFEPELAVARRMMTKVIAVTSVLDDIYDVYGTYEELELFTQAIQRWDIDCIHELPEYMQVFYKALINIYVEIEEILACTGKSYCLCYAVEAMKRQARYYFAEAKWLHQQHKPTMDEYMSIALVSSGYPLLAVTSFVGMPDIVTKDDLDWLFNDPKILKASTIICRLMDDLATHKFEQSRGHVDSAVQCYMKLYGVTEQEAENNLRKQVNDSWKDINEECLRPTAVAMPLLVGILNLSRVMDVLYKDGGDHYTNPHIALKDYIHSVLIDPVQ

>EgranTPS052

NDALTFITHLESIDKRKVSPNLEKQVSHALNHPIRKGLPRLEARRYIQFYQEEPSHNEVLLSLAKLDFNSLQGQHQKELGNLTRWWKDLDIKREFPFTRDRLAELYVWMLGVHFEPDYEIARGIVTKMMVIISILDDIYDVYGTLEKLEIFTEAIEKWDVDAIEGLPECMQVYKIIFEFYDEIGYELTRKGRSYCLFYAKEAMKIQVRAYLVEAKWFHKSHIPTMEEYMPIALTTIGNQMAFVASFLGMRDIVTKDTFDWLLSSNHKIVKASKVIGRLMNDIAGHFEQERGHVASSVECFMKQYKVTEEEAKKEIRKLVADAWKDIDEELRHPTAVPMVVLMRIVNLAGAIHAVYEDETDHYVNAGTNFKEFVTCLLVNPCQCDGLEEQV

>EgranTPS053

MEQVQRLKEEVRGLFDREINQVAKLEFIDVVQRLGLGYHFEMEIKNALSSIYNNTEDAQISDNLYATSLRFRLLRQHGYNVPQDVFQRFMSKMGTFNELLHEDVKGLLGLYEASFHGLEGETILDEGWNFASKHLNDLNLDKVPTNIASHVSHALDMPIHWRPNRLEAQWFMDMYGKQQDMIPSLLRLAKIDFNLVQSIHRKEVSNLARWWVELGANKMTFSRDRLVENYFWSCLFVFEPQYTAFRELSTRIGCMVSLIDDIYDIYGTPEELELLTDLILRWDITNIDKLPPTIRDGFMVLYNTTNKLGYWTMRERGINPIPYLRKLWADECKAYMKEVYWYNKGIKPTLKEYMDVGVDSIGGLILLLDSYFLTTDKLTEEGLDYVSKIPGVMHSSAKILRFNDDLSTSSHELARGDNSKALECYMNETGASEEAAREHIRHLVRETWKKMNKEVFEDYPFSGFGPFLSACLNLARASHCFYDYGDGHGLPGHQTKDHVVSTIFEYVPL

>EgranTPS054

TCAVTIENPEIVRRSANWKPTVWDYEFLKRLLRQHGYKIPQDVFQQFMNKTGTFNESLNKDVRGLLSLYEASFHGLEGETILDEARNFASKHLKDLNLDKVPTMLASYVRHALDMPIHWRPNRLEARWFMDMYEKQQDMIPSLLRLAKLDFNLVQSVHKKEVSNMARWWVELGANKMTFFRDRLVEHYFWCCAMVFEPQYTAYREMTTKLTCMVTLIDDVYDVYGTQEELELLTDFLVRWDITEIDKLPPIIRDSYMALYNTTNEIGYWTMRELGINTIPYMQKVWADECKAYIKEVHWYNKGIKPTLKEYMDNAVDSIGGLIMLLGSYFLTTDKLTEEGLDYVSKIPSVMHCSAKILRLNNDLSTSSYELARGDNFKALECYMNETGASEQATREHVKQMVHETWKRMNKDVFEDYPYSGFGPFLGACLNLARASQCFYQYGDGHGLPDNETKDHLVRALFDPVPL

>EgranTPS055

TCALKIEDQEIARRSANWEPSVWDYGVVMEQVQRLKEEVRGLFDREINQVAKLEFIDVVQRLGLGYHFETEIKNALSSIYNNTEDAQISDNLYAASLRFRLLRQHGYNIPQDVFQRFMSKMGTFNESLHEDVKGLLGLYEASFHGLEGETILDEGWKFASKHLKDLNLNEVPTDIASNVSHALDMPIHWRPNRLEARWFMDMYGKQQDTIPSLLRLAKIDFNLVQSIHRKEVSNLARWWVELGANKMTFFRDRLVENYFWSCIFVFEPQYTAFRELNTRIGCLVTLIDDVYDIYGTPEELELLTDFILRWDITNIDKLPPTIRESFMVLYNTTNEVGYWTMRERGINPIPRKVWADECKAYMKEVYWYNKGIKPTLKEYMDVAVDSIGGLILMLDSYFLTTDEVTEEGLDYVSKIPGVMHSSARILRFNDDLSTSSHELARGDNSKALECYMNETGASEEAAREHIKHLVLETWKKMNKEVFEDYPFSGFKPFLGACLNLARASHCFYDYGDGHGLPGHQTKDHLVSTIFKSVPL

>EgranTPS056

TCAVTIENPEIVRRSANWKPNVWDYEFLAEQVPRLKEEIRGLFNREMNQVAKLEFIDAVQRLGLGYHFETEIKNALSSIYDNAGYAQLLNDLHAVSLGFRLLRQHGYKISQDVFQQFMNKMDTFNESLNKDARGLLGLYEASFHGLEGETILDEARNFASKHLKDLNLDKVPAMLASYVSHALDMPIHWRPNRLEARWFMDMYEKQQDMIPSLLRLAKLDFNLVQSVHKKEVSNMARWWVELGANKMTFFRDRLVEHYFWTCAMVFEPQYTAYREMTTKLTCMVTLIDDVYDVYGTQEELELLTDFLVRWDITEIDKLPPTIRDSYMALYNTTNEIGYWTMRELGINTIPYMQKVWADECKAYIKEVHWYNKGIKPTLKEYMDNAVDSIGGLIMLLGSYFLTTDKLTEEGLDYVSKIPSVMHCSAKILRLNNDLSTSSYELARGDNFKALECYMNETGASEEATREHVRQMVHETWKRMNKDVFEDYPYSGFGPFLGACLNLARASQCFYQYGDGHGLPDNETKDHLVRALFDPVPL

>EgranTPS057

TCAVTIENPKIVRRLANWKPNVWDYEFLAEQVQRLKEEIRGLFNREMNRVAKLEFIDAVQRLGLGYRFETEIKNALSSIYDNTGYAQLLNDLHAVSLGFRLLRQHGYKIPQDVFQQFMNKTGTFNESLNKDVRGLLGLYEASFHGLEGETILDEARNFASKHLKDLNIDKVPAMLASYVSHTLDMPIHWRPNRLEARWFMDMYETQQDMIPSLLRLAKLDFNLVQSVHKKEVSNMAKWWVELGTNKMTFFRDRLVEHYFWCCAMVDEPQYTAYREMTTKLTCMVTLIDDVYDVYWTQEELELLTNFLVRWDITEIDKLPPIIRDSYMALYNMTNEIGHWTMRELGINTIPYLQKVWADECKAYIKEVHWYNKGIKPTLKEYMDNAVDSIGGLIMLLGSYFLTTDKLTEEGLDYVSKIPSVMHCSAKILRLNNDLSTSLYELAGDNFKALECYMNETGASEEATREHVRQMVHKTWKRMNKDVFEDYPPSGFGPFLGACLNLAQASKCFYQYEDRHGLPDNETKDHLVRALFDPVPL

>EgranTPS058

TCASKIEDQEIARRSANWEPSVWDYGVVQSLSRLLRQHGYNVPQDVFQRFMSKMGTFNESIHEDVKGLLGLYEASFHGLEGETILDEGWKFASKHLKDLNLNEVPTNIASNVSHALDMPIHWRPNRLEARWFMDMYGKQQDMIPSLLRLAKIDFNLVQSIHRKEVSNLARWWVELGANKMTFFRDRLVENYFWSCIFVFEPQYTAFRELNTRIGCLVTLIDDVYDIYGTPEELELLTDFILRWDITNIDKLPPTIRDSFMVLYNTTNEVGYWTMRERGINPIPYLRKVWADECKAYMKEVYWYNKGIKPTLKEYMDVAVDSIGGLILMLDSYFLTTDEVTEEGLDYVSKIPGVMHSSARILRFNDDLSTSSHELARGDNSKALECYMNETGASEEAAREHIKHLVRKTWKKMNKEVFEDYPFSGFKPFLGACLNMARASHCFYDYGDGHGLPGHQTKDHLVSTIFESVPL

>EgranTPS059

TCTLKIEAQEIGRRSANWQPNVFDYDFLNRLLRQHGFNISQDVFQRFMSKSGTFNESLNEDVKGLLGLYEASFHVLEGETILDEAWTFASKHLKDLNLDKIPTNLATHVDHALEMPIHWRPNRLEARWFIDMCEKQQDMIPSLLRLAKLDFNSVQSIYRKEVSTLARWWVELGANKMTFGRDRLMENYFWSMIMVFEPQHIAFREMNGKIASMVTLIDDVYDVYGTPEELELLTDFIVRWDITDVDRLPPIIRDSFMALYNTTNEIGYWTMRERGINPIPHLQKLWAEECKAYLKEVHWCSKGIKPTLKEYMDVATYSTGGLVMLLASYFLTTDKLTEEGLNYVSKIPSIMHCSCKMLRLINDFSTSSYELARGDNLKALECYMNETGASEEAAREHIMHKVREGWKLMNRAVFEDYPIPGLRPFLGACLNQARVSHTFYRYGDGFGRPDNDTKDYLASAIYKPVPL

>EgranTPS060

TCAVTIENPEIVRRSANWKPNVWDYEFLAEQVQRLKEEIKGLFNREMNQVAKLEFIDVVQRLGLGYHFETEIKNALSSIYDNTGYAQLLNDLYAISLGFRLLRQHGYNIRQDVFQQFMNKTGTFNESLNKDVKGLLGLYEASFHGLEGETMLDEARNFASKHLKDLNLDKVPTMLASYVSHTLDIPIHWRPNKLEARWFMDMYEKQQDMIPSLLRLAKLDFNLVQSVHKKEVSNMARWWVELGANKMTFFRDRLVEHYFWNCTMVFEPQYTAYREMTMKLACMVTLIDNVYDVYGTLEELELLTDFLVRWDITEIDKLPPTIRDSYMALYNTTNEIGYWTMRELGINTIPYMRKVWADECAYIKEAHWYNKGIKPTLKEYMDNALDSIGGPIMLLGSYFLTTDKLTEEGLDYVSKIPSVMHCSAKILRLNNDLSTSSYELARGDNFKALECYMNETGASEEAAREHVKQMVHETWKRMNKDVFEDYPYFGLGPFLDACLNFARASQCFYQYGDGHGLPDNETKDHLVRALFDPVPL

>EgranTPS061

TCTLKIEAQEIGRRSANWQPSVWDYDFVSEEAQRLKKEVKGQFDREMNSVAKLEFIDVVQRLGLGYQFETEIKNALSSIYNNTEVAQLLDDLDAVSLRFRLLRQHGFNVSQDVFQRFMSKSGTFNESLNEDVKGLLGLYEASFHVLEGETILDEAWTFASKHLKDLNLDEIPTNLVSQVDHALEMPIHWRPNRLEARWFIDMCEKQQDMIPSLLRLAKLDFNLVQSIYRKEVSGLARWWVELGANKMTFCRDRLVENYFWSSIMVFEPQHTAFREMNGKIASMVTLIDDVYDVYGTLEELELLTDFIVRWDITDIDRLPPTIRDSFMALYNTTNEIGYWTMRERGINPIPHLQKVWADECKAYLKEVHWCSKGIKPTLKEYMDVATYSAGGLVMLLASYFLTTDKLTEEGLNYVSKIPSIMHCSSKMLRLINDFSTSSYELARGDNLKALECYMNETGTSEEAAREHIMHMVREAWKWMNRAVFEDYPIPGLRPFLGACLNLARVSHTFYRYGDGFGLPNNDTKDYLVSAIYKPVPL

>EgranTPS062

VDPNTNEQSPVARRSANYMPSVWDYDILSRLLRQHGYDLSQDAFQRFINKTGTFEESLKKDVKGLLGLYEASFHGLEGENILDEAQDFASKHLKNLNLNEIPTCLAKQVLHALDMPIRWRPNRLEARWFMDMYGKQQDMIPSLLRLAKLDFNLVQTIHRKEVSNLARWWVELGANNMTFSRDRLVENYFWSCLMVFEPQYTAYREMTTKIGCMVTLIDDVYDVYGTLEELVLLTDFIVRWDITDIDNLPPTIRNSFMALYNTTNEIGHWTMREQGINPIPYMRKVWADECRAYIKEVHWYNEGIKPTLKEYMSNAVDSIGGLIMLLHSYFLTTDNLTKEGLDYMSKIPRIMHCSAKILRLNDDLGTSSYELARGDNFKALECYMNETGASTEAAQQHIKHLVRETWKTMNKDVFEDYPFPGFKPFLGACLNLARASQCFYQYGDGHGLPGHETRDHIVSTLFKPVPL

>EgranTPS063

KCSLTIEDQDTARRSANWKPSVWDYGSVTEQVQRLKEEVKGLFHREINQVAKLEFIDVVQRLGLGYHFETDIKNALSSIYNNTEDAQLSDDLYAVSLRFRLLRQHGYNLQQDVFQRFMNKMGTFNESLKEDVRGLLSLYEASFHGLEGETIVDEAWNFASKHLKDLNLDEVPANLASNVSHALDMPIHWRPNRLEARWFMDTYEKQQDKIPCLLRFAKVDFNIVQSIHKKEVSNMARWWVELGANKMTFFRDRLVEHYFWCCAMVFEPQYTEFREMTTKLTCMVTLIDDVYDVYGTLEELEVLTDFIVRWDITDVDKLPLKIRACFLALYNTTNEIGYWMMRERGINPIPHMRKVWADECKAYIKEARWYNKGIKPTLDEYVDNGVTSIGGLIMLLGSYFLTTDKPTEEGLDYVSNIPSVMHCSAKILRLNNDLSTSSYELARGDNFKALECHMNETGASEEATREHIKHLVRKTWKRMNRDVFEDYPYSGFGPFLGACLNLARASQCFYQYGDGHGLPDHETRAHIVSALFDPVPL

>EgranTPS064

TCASKIEVQEIGRCPANWQPSIWDYDFVSEEVQRLKKEVKGLFDREMNQVAKLEFIDMVQRLGLGYQFKMEIKNALSSIYTEDAQLSDDLEAVSLRFRLLKQHGYNVPQDVFQRFMSKTDTFNESLNEDVKGLLGLYEASFHGLEGETILDEAWTFASKHLKDLNLDEIPTNLASHVSHALDMPIHWRPNRLEARWFIDMYKKQQDMIPSLLRLAKLDFNLVQSIYRMEVSRLARWWVELGANKMTFCRDRLVESYFWSNTMVFEPQHTAVREMNGKIASMVVLIDDVYDIYGTLEELELLTDFIVRWDITNIDSLPPTIRDSFMALYNTTNEIGYWTMRERGINPIPYLQKVWADECKAFLKEVHWCSNGIKPTVKEYMDVATYSAGGLVLLLASYFLTTDKLTKEGLDYVSKIPSIMHCSSKMLRLINDFSTSSYEVARGDNLNALECYMNETGASEEAAREHITHMVREAWKWMNRAVFEDYPIPGIGPFLGACLNLARVCHTFYRYGDGFGHPSNDTKHYLVSAIYEPVPL

>EgranTPS065

ANQPEVVDQMHQRRSANYKPNIWNYDFLEEVKPMLSEAVDSLAKLELIDCMTKLGLSNLFENEMKEALETVASIHNGVFTMEEHLYASALRFRLLRQHGHIVSQDELRRFKEGSILFNRSNGEDVKTMIELLEASHLALEGENILHEAKAFSTGILRERVSSLDGRLFKRTVHALELPLHWRVQWFDIKWQISLYEQREDKQSNLLELAKLNFNTVQATHQRDLREISRWWRDLGLMEHVDFTRDRLVESFLCALGLSQETRLSSLRKSLTKVVILILVIDDVYDLYGSLEELECFTSAINRWDSEQIQQLPECMKVCFRALNDVIHEIAYDIGKDEDWHRLPHLAKAWADFCKALLTEAKWDNKGYTPSLEEYLSNAWTSSSGPLIMSHASFFVGHMNLEDLADLLERNKDLIYNVSMIIRLCNDLGTSTSERDRGDAPSSVVCYMQEANVPEDVARKHIKELINQAWKSINAHCFGNVETPFVRTFIDVTVNASRVAHMLYQFGDGFGVQDGDIRRQILSAVIHPIAL

>EgranTPS066

ANLPEVVGTMNQRRTANYKPNIWNYDFLKERIEGLVEEVKRILSEVVDSLAKLELIDSMTKLGLSNLFENEMKEALETVASINNGVFTMEEHLYASALWFRLLRQHGHIISQDELRSFKEGSILFNRSNCEDVEYVELLEASHLALEGENILHEAKTFSTGILRKRVSSLDGRLFKRAVHALELPMHWRVQWFDIKWQISLYEQREDKQSNLLELAKLNFNTVQATHQRDLREISRWWRDLGLMEHVDFTRDRLVESFLCALGLSQEPRLSSLRKSLTKVVILILVIDDVYDLYGSLEELECFTSAITRRDSEQIQQLPECMKVCFRALNDVIHEIAYDIGKDEDWHRLPHLAKWEDFCKALLTEAKWDNMGYTPSLEEYLSNAWTSSSGPLIMSHASFFVGHMNWEDVADLLERNKDLIYNVSMIIRLCNDLGTSTAERDRGDAPSSVVCYMREANVPEDIARKHIKELINQEWKSINAYCFSNADTPFVRTFIDVTANAARVAHMLYQFGDGFGVQDGDIRRQILSAVIHPLAL

>EgranTPS067

HQRRSANYKPNIWNYDFLKERMEGLVEEVKPMLSKSVDSLAKLELIDSMTKLGLSNLFENEMKEALERVASNNNGVFTMEEHLYASALRFRLLRQHGHIVSQNELRRFKEGSILFNRSNCEDVEAMIELLEASHLALEGENILHEAKAFSTGILRERVSSLDGRLFKCTVHALEIPLHWRVQWFDIKWQISLYEQREDKQSNLLELAKLNFNTVQATHQRDLREISRWWRDLGLMEHVEFTRDRLVESFLCALGLSQETRLSSLRKSLTKVVILILVIDDVYDLYGSLEELECFTSAITRWDSEQIQQLPECMKWADFCKALLTEAKWDNMGYTPSLEEYLSNAWTSSSGPLIMSHASFFVGHMNLEDVADLLERNKDLIYNVSMIIRLCNDLGTSTAERDRGDAPSSVVCYMREANVPQDVARKHIKELINQAWKSINAHCFGNVETPFVRTFIDVTVNASRVAHMLYQFGDGFGVQDGDIRRQILSAVIHPVAL

>EgranTPS068

AHLQQVVGTMHQRRSANYKPSIWNYNFLEEVKRMLSEVVNSLAKLELIDIMTKLGLSNLFENEMKEALETVASINNGVFTMEEHLYANALRFRLLRQHGHIISQDELRSFKEGSILFNRSYCEDVEAMIELLEASHLALEGENILHEAKTFSTGILRKRVSSLDGRLFKRAVHALELPMHWRVQWFDIKWQISLYEQREDKQSNLLELAKLNFNTVQATHQRDLAEISRWWRDLGLMEHVEFTRDRLVESFLCALGLSQEPRLSSLRKSLTKVVILILVIDDVYDLYGSLEELECFTSAITRRDSEQIQQLPECMKVCFRALNDVIHEIAYDIGKDEDWHRLPHLAKAWADFCKALLTEAKWDNMGYTPSLEEYLSNAWTSSSGPLIMSHASFFVGHMNLEDVADLLERNKDLIYNVSMIIRLCNDLGTSTAERDRGDAPSSVVCYMREANVPEDIARKHIKELINQEWKSINAYCFSNAETPFVRTFIDVTVNAARVAHMLYQFGDGFGVQDGDIRRQILSAVIHPLAL

>EgranTPS069

ANLPEVVGTMHQRRTANYKPNIWNYDFLKERMEGLVEEVKRILSEEVDSLAKLELIDSMTKLGLSNLFENEMKEALETVASINNGVFTMEEHLYASALRFRLLRQHGHIISQDELRSFKEGSILFNRSNCEDVEAMIELLEASHLALEGENILHEAKAFSTGILRKRVSSLDGRLFKRAVHALELPMHWRVQWFDIKWQISLYEQREDKQSNLLELAKLNFNTVQAAHQRDLREISRWWRDLGLMEHVDFTRDRLVESFLCALGLSQEPRFSSLRKSLTKVVILILVIDDVYDLYGSLEELECFTCAITRRDSEQIQQLPECMKACCRVLNDVIHEIAYVIGKDEDWHRLPHLWAEFCKALLTEAKWDNMGYTPSLEEYLSNAWTSSSGPLIMSHASFFWEGVNWEDVAEGLERNKDLIYNVSMIIRLCNDLGTAERDRGDAPSSVVCYMREANVPEDIARKHIKELINQEWKSINAHCYSNAETPFVRTFIDVTVNAARVAHMLYQFGDGFGVQDGDIRRQILSAVIHPLAL

>EgranTPS070

ANLPEVVGTMHQRRSANYKPNIWNYDFLDLVREGLVEEVKPMLSEVVDSLAKLELIDSMTKLGLSNLFENEMKEALETVASINNGVFTMEEHLYASALQFRLLRQHGHIISQDELRSFKEGSILFNRSNCEDVEAMIELLEASHLALEGENILHEAKAFSTGILRERVSDLDGRLFKRTVHALELPLHWRVQWFDIKWQISLYEQREDKQSNLLELAKLNFNTVQATHQRDLREISRWWRDLGLMEHVDFTRDRLVESFLCALGLAQEPRLSSLRKSLTKVVILILVIDDVYDLYGSLEELECFTSAITRRDSEQIQQLPECMKVCFRALNDVIHEIAYDIGKDEDSHRLPHLWADFCKALLTEAKWDNMGYTPSLEEYLSNAWTSSSGPLIMSHASFFVGHMNLKDVADLLERNKDLIYNVSMIIRLCNDLGTSTAERDRGDAPSSVVCYMREANVPEDIARKHIKELINQEWKSINAYCFSNAETPFVRTFIDVTVNAARVAHMLYQFGDGFGVQDGDIRRQILSSVIHPLAL

>EgranTPS071

HQRRSANYRPNLWKYDFLKEQVERLVEEVKPTLSEAVNSLLKLELIDKMKKLGLSNLFGNEIKEVLQTVASTNNGVFNMEDHLYASALQFRLLRQHGHVVSQDALRSFRDGRNTLSGSNCGDVEAMIELLEASYLAMEGENILEEAKAFSTGILQERVSGLDGQLLKRAVHALELPMHWRVQWFDIKWQIDLYEQQEDKQSSLLPLAKLNFNIVQATHQRDLRDISRWWRDLGLIEHVDFARDRLVESFFCALGLSQEPQFSSFRKSLTKVIILILVIDDLYDLYGSLEELECFTDAITRWDLEQIQQLPECIKVCFQALRDVSYEIAHEIGKDEDWHQVPHLMKVWADFCKALLNEAKWDKMGYTPSLEEYLSNAWTSSSGPLILSHAYYLMGQMKLEDAADFLKRNKDLIYNVSIIIRLCNDLGTSTAERERGDAPSSVACYMREANVSEDIARKHIPILINQAWNSINAHCFGNAEKPFLRPFINVTVNAARVVHMLYQFGDGFAIPDGDIQQQILSIVIKPLAL

>EgranTPS072

ANSRQAVDSMHQRRSANYKPNIWKYDFLEDVKAMLAEAVDSLTKLELIDWMRKMGLSNLFDKEMKEALETVASINNGIFAKEDHVYASALRFKLLRQQGHVVSQDELRSFKEESDTFNRSNCEDIEAMMQLLEASHLALEGEDILDEGKAFSAEILRERVSSLDGPLLKGAVHALELPMHWRVQWFDIKWQIDLYEQQEDKQSNLLELAKLNFNTVQATHQRDLIEISRWWRDLGLIERVDFTRDRPVESFLYALGLSQEPRFSSLRKSLTKVVIFILLIDDVYDLYGSLEELECFTSAVTRWDSESIQQLPECMKVCFRALQDVTYEIAHEIGKEEDWHRLPHLTKAWADFCKALLTEAKWDLLGYTPSLGEYLSNAWTSSSGPLLLSHAYFFVGHMKLEDAAESVERNKDLIYNASMIIRLCNDLRTSKAEIERGDAPSSMVCYMREANVSEDIARKHIKGLIDQAWKNINAHCFVNAETPFLRPYIDVTVNAARAAYMIYQSGDGFGVQDGTIGQQMLSAVIEPLAL

>EgranTPS073

TCASRIEVQEIGRHSANWQPSVWDYDYLSEEVQRLKKEVKVLLDGEMNQVAKLKLIDAVQRLGLGYQFEMEIRNALSSIYNNTEDAQLSENLDVVSLRFRLLRQHGYNIPQDVFQRFMSKTGTFNESLNEDVKGLLGLYEASFHGLKGETIIDEAWTFASKHLKDLNLNEIPTNLASHVSHALVMPIHWRLNRLEARWFIDMYKKQQDMIPSLLRLAKLDFNLVQSVYRKEVSNLARWWVELGANKMTFCRDRMVESYFWSNSMVFEPQHTAFREMNGKIASMVVLIDDVYDIYGTLEELELLTDFIVRWDITDIDRLPPIIRDSFMAMYNMTNEIGYWTMRERGINPIPYLRKVWADQCKAYLKEVHWRSKGIKPTLKEYIDVATNSSGGLVLLLPSYFLTTDKLTEEGLDYVSKIPSIMRCSCKMTRLINDFSTSSHELARGDNLKALECYMNETGVSEEAAREHIMHMVREAWKWMNRAVFEDYQIPGLRPFLGACLNMARICHTFYGCGDGFGQPSNDTKDSLASAIYEPVPL

>EgranTPS074

TCASKIEVQEIGRHSANWQPSVWDYDFLTRLLRQHGYNTPQDVFQRFMSKTGTFNESLNEDVKGLLGLYEASFHGLEGETILDEAWTFASKHLKDLNLNEIPTNLASHVSHALDMPIHWRLNRLEARWFIDMYKKQQDMIPSLLRLAKLDFNLVQSVYTKEVSNLARWWVELGANKMTFCRDRIVESYFWSNSMVFEPQHTAYREMNGKLASMVVLIDDVYDIYGTPEELELLTDFIVRWDITDIDRLPPIVRDSFMAMYNTTNEIGYWTMRERGINPIPYLRKVWAEECKAYLKEVHWRSKGIKPTLKEYIDVATNSSGGVVLMLPSYFLTTDKLTEEGLDYVSKIPSIMRCSSKMLRLINDLSTSSHEVARGDNLKALECYMNETGVSEEAAREHIMHMVREAWKWMNRAMFEDYRIPGLGPFLGACVNTVRICHTFYGCGDGFGQPSNITKDSLASAIYDPVPL

>EgranTPS075

TCASKIEVQEIGRHSANWQPSVWDYDFLSEEVQRLKKEVKGLFDREMNQVAKLKFIDVVQRLGLGYQFETEIKNALSSIYNNTEDAQLSDNLDVVSLQFRLLRQHGYNIPQDVFQRFMSKTGTFNESLNEDVMGLLGLYEASFHGLEGETIIDEAWTFASKHLKDLNLNEIPTNLVSHVSHALDMPIHWRLNRLEARWFIDMYKKQEDMIPSLLRLAKLDFNLVQSVYRKEISNLARWWVELGANKMTFCRDRIVESYFWSNSMVFEPQHTAYREMNGKIASMVVLIDDVYDIYGTPEELELLTDFIVRWDITDIDRLPPIIRDSFMAMYNTTNEIGYWTMRERGINPIPYLRKVWAEECKAYLKEVHWRSKGIKPTLKEYVDVATNSSGGVVLMLPSYFLTTDKLTEEGLDYVSKIPSVMRCSSKMLRLINDLSTSSHEVARGDNLKALECYMNETDASEEAAREHIMHMVREAWKWMNRAVFEDYRIPGLGPFLGACVNTARICHTFYGCGDGFGQPSNITKDSLASAIYEPVPL

>EgranTPS076

VCPVRSIWEPPCRGLCSGDESWGLRFQNGRVQMLEQEVRSAMKDESAELSTILALVDDIQRLGLVFLFEEDVKRALRRYHSPDGGYKNRDQKTLHGTALFFRILRQNGFEVSPDVFRIFMDERGTFMESLGRDVEGLLSLYEASHLAFEEEGILLEAKEFAVKHLKRLNDIDISKDLEYRVNHGSVPPLHQRMPLLEARQSIEAYSPQRDAERRLLELAVYNFNMVQSILQRDLQEMSRWWNDVSLANELSFARDRLMECFFWTVGMAYEPQFSNLRKGLTKVTALVTTIDDVYDVYGSLDELELFTDAVHRWDVNALSNLPSCMKLCFLALYNAVHEMAYDVLKQNGENIIPCLTKVWSDMFKAFLQEAKWKHDKVTPTFEEYMNNGWISVSGLVILIHAFFLSTPDVRKEEIESIETHGHDLLKSPAIIFRLCNDLGTSSAELERGETANSILCYMQDTGVSENVAREHIKELIDIEWKNMNRYQVDYSMFGKSFVRLAFNLARIAHYTYQDGDAHGDPDDRAKYRIHSLLIDPISL

>EgranTPS077

MLEQEVRSAMKDENAELSTILALVDDIQRLGLIFLFEEDVKRALRRYHSLDGGYKNRDQKTLHGTALYFRILRQNGFEVSPDVFWIFMDEQGTFMESLGRDVEGLLSLFEASHLAFEDEDILHEAKAFAIEHLKRLNNIDVNKDLEYQVNWGLALPLHQRMPLLEARRSIEAYRTRRDADHRLLELAVYNFNMVQSILQRDLQEMSRWWNDVSLANKLSFARDRLMECFFWTVGMAYEPQFSNLRRGLTKVTALVTTIDDVYDVYGSLDELELFTDAVHRWDVDAVSSLPGCMKLCFLALYNAVHEMAYDVLKQNGENIIPCLTKAWSDMLKAFLQEAKWKHNKVTPTFEEYMNNGWISVSGLVILIHAFFLSTPHIRKEELELIETYGHDLLKSPSIIFRLCNDLGTSSAELERGETANSILCYMQDTGVCENVAREHIKELIDTAWKKMNRYQVDNSLFGKSFVRLAFNLARIAHYTYRDGDAHGAPDDRSKYRIHSLLIDPISL

>EgranTPS078

STSTKAAFPQGGRRSANYQPSVWTHNDLQREKAQMVEEVRGALHDENAELITIFALVDDIQRLGLGRHFEEDISRALHRCLSVYKGLQKSLHGTALSFRILRQHGFEVSQDVFKIFMDESGSFMKNLGNDVQGMLSLYEASHLAFEEEDILHEAETFTIEHLKNHNRDINKDLQGEVNHELEWPLHRRMSLLEARRFIEAYSRRRYTSHRILKFSATNFNTLQSTLQGDLQEVFRWWDNVGLANELNFARDRLVECFFAAVAVADEHPLSNCRKGLTKANILNVIIDDVYDIYGTLDELELFTDAVRRWEINAVEDLPGYMKLCFLALYNCVNELAYDTLKETRENVIPYLTKVWYDACEAFLQEAKWSHNKITPRVEEYLNNGWISVSGHVMLIHAYFLSSPSMRKEELESLEHYHDLLRLPSMIFRLTNDLATSSAELERGETTNSIWCYMQEMGVSELEARKYVIKMIDTTWKKLNKYLVNDSTFNQSFVRMAFNLARMAHCMYHDGDAVGAPDDLSMNRVHSLIIDPVSL

>EgranTPS079

MALQIFSLPNVCSKRIPAQVPRSASAKAAVSQGGGRSPQYQPTLWTYDYLQSLPIGVHRRVVINSHFVRSQSEQQNGRVQMLEQEVRSAMKDENAELSTILALVDDIQRLGLIFLFEEDVKRALRRYHSLDGGYKNRDQKTLHGTALYFRILRQNGFEVSPDVFWIFMDEQGTFMESLGRDVEGLLSLYEASHLAFEEEDILHEAKAFAIEHLKRLNNIDVSKDLEYFQVNWGLALPLHLRMPLLEARRSIEAYSTRRDADRRLLELAVYNFNMVQSILQRDLQEMSRWWNDVSLANKLSFARDRLMECFFWTVGMAYEPQFSNLRRGLTKVTALVTTIDDVYDVYGSLDELELFTDAVHRWDVDAVSSLPGCMKLCFLALYNAVHEMAYDVLKQNGENIIPCLTKAVWSDMLKAFLQEAKWKHNKVTPTFEEYMNNGWISVSGLVILIHAFFLSTPHIRKEELELIETYGHDLLKSPSIIFRLCNDLGTSSAELERGETANSILCYMQDTGVCENVAREHIKELIDTAWKKMNRYQVNNSLFGKSFVRLAFNLARIAHYTYQDGDAHGAPNDRSKYRIHSLLIDPISLE

>EgranTPS080

STSTKAAFPQGGRRSANYQPSVWTHNDLQREKAQMVEEVRGALHDENAELITIFALVDDIQRLGLGRHFEEDISRALHRCLSVYKGLQKSLHGTALSFRILRQHGFEVSPDVFKIFMDESGSFMKTLGNDVQGVLSLYEASRLAFEDEDILHEAETFTIEHLKNHNRDINKDLQGEVNHELEWPLHRRMSLLEARRFIEAYSRRRYTSHRILKFSATNFNTLQSTLQGDLQEVFRWWDNVGLANELNFARDRLVECFFAAVAVADEHPLSNCRKGLTKANILNVIIDDVYDIYGTLDELELFTDAVRRWDINAVEDLPGYMKLCFLALYNCVNELAYDTLKETRENVIPYLTKAWYDACEAFLQEAKWSHNKITPRVEEYLNNGWISVSGHVMLIHAYFLSSPSMRKEELESLEHYHDLLRLPSMIFRLTNDLATSSAELERGETTNSIWCYMQEMGVSELEARKYVIKMIDTTWKKLNKYLVNDSTFNQSFVRMAFNLARMAHCMYHDGDAVGAPDDLSMNRVHSLIIDPVSL

>EgranTPS081

SGSTQVSDTQGGRRSANYQPTVWTHNYLQREKAQMLEEVRGALNDEKAEPMTIFALVDDFQRLGLGQHFKEDISRALRRCLSNDAVNKSRQKSLHSTALSFRILRQHGFEVSQDDFKIFMDKSGNFMKTLGGDVQGMLSLHEASHLAFEEEDILQEARSFAIEHLRNLNCKVDKDLQDQVKHELELPLHCRMPMLEARRSIEAYRRCGYTNHRIPEFAATNFDTLQSILQRDLQEMSRWWNDVSLARNLNFVRDRLTECFFWAAGVADEPTLTNCRKRLTKVTSLITIMDDVYDVYGTLDELELFTDAVRRWDINAVDDLPGYMKLCFLALFNSVNEIAYDTLKETGKIVIPYLAKSWYDLCKSFLQEAKWSYKKTNPRFEEYLNNGWISSSGHVILIHAYFLSGPSMGREELESLEHYHDILRLPSMIFRLTNDLVTLSAELERGETTNSITCYMQEMGVSESEARDYVMKLIDTSWKQMNKYLVNGSTFDQSFVRMAYNLARTTHFMYQDGDAHGSPDNRSRNRMHSLIIEPISL

>EgranTPS082

PSPTANVESIQARRSLILKKDGDRPIQREKAQMLEEVRGVLNDEKAEPMTIFALVDDIQRLGLGRHFEEGISRALRRCLSKNAVNKSRQKSLHGTALSFRILRQHGFEVSQDVLRIFMDESGSFMKTLGGDVQGMLSLHEASHLAFEEEEILHGARSFAIEHLRNLNRKVNKDLQDRVKHELELPLHCRMPLLEARQSIEAYSRGYTNHRILEFVVMNFNMSQPILQRDLQEMSRWWNNVGLANNLNFARDRLVECFAAVAVADEHPLSNCRKGLTKVNMLIVIIDDVYDIYGTLDELELLTDAVRRWDINAVEDLPGYRKLCFLALYNSVNELAYDTLKETRENVIPYLTKVWYDSCKAFLQEAKWSHNKITPRVEEYLNNGWISISGHVMLIHAYFLSSPSMRKEELESLEHYHDLLRLPSMIFHLTNDLATSSAELERGEATNSIWCYMQEMGVSELEAGQYVIKRIDTTWKKLNKYLVSDSTFNQSFVRMAFNLARMAHCIEEPCALTNN

>EgranTPS083

SASTQVSDTQGGRRSANYQPTVWTHNYLQREKAQMLEEVRGALNDEKAEPMTIFALVDDIQRLGLGQHFEEDISRALRRCLSNDAVNKSRQKSLHGTALSFRILRQHGFEVSQDVFKILMDESGSFMKTLGGDVQGMLSLHEASHLAFEEEDILQEARSFAIEHLRNLNCNVDKDLQDQVKHELELPLHCRMPLLEARWSIEAYRRCRYPDHRIPEFAAMNFNTLQSILQRDLQEMSRWWNDVSLARNLNFVRDRLMECFFWAAGVADEPTLANCRKRLTKVTSLITIIDDVYDVYGTLDELELFTDAVRRWDINAVDDLPGYMKLCFLVLFNSVNEIAYDTLKETGKIVIPYLAKSWYDLCKSFLQEAKWSYNKTNPRFEEYLNNGWISSSGHVILIHAYFLSSPSMRREELESLEHYHDILRLPSMIFRLTNDLVTSSAELERGETTNSIMCYMQEMEVSESEARDYVMKLIDTSWKQMNKCLVNGSTFDQSFVRMAYNLARTTHFMYQDGDAHGAPDNRSRNRMHSLIIEPISL

>EgranTPS084

SASTQISDPQEGRRSANYQPSVWTYNYLKEKVQILEEEVRGALNDEKAETFTIFATVDDIQRLGLGDHFEEDISNVLRRCVSVFMSLQKSLHGTALGFRLLRQHGYEVSQDVFKIFLDESGSFVKTLGGDVQGVLSLYEASHLAFEEEDILHKARSFAIKHLENLNSDVDKDLQDQVKHELELPLHRRMPLLEARRSIEAYSRRGYTNPQILELALTDFNVSQSTLQRDLQEMLGWWNNTGLAKRLSFARDRLIECFFWAVGIAHEPSLSICRKAVTKAFALILVLDDVYDVFGTLEELELFTDAVRRWDLNAVEDLPVYMKLCYLALYNSVNEMAYETLKEKGENVIPYLAKAWYDLCKAFLQEAKWSNSRIIPGVEEYLNNGWVSSSGSVMLIHAYFLASPSMRKEELESLEHYHDLLRLPSLIFRLTNDIASSSAELERGETTNSIRCFMQEKGISELEARECVKEEIDTAWKKMNKYMVDRSTFNQSFVRMTYNLARMAHCVYQDGDAIGSPDDLSWNRVHSLIIKPIPA

>EgranTPS085

TRALKIEGQEIVRRSANWQPSIWDYGLVQSLGRLLRQHGYHVPQDVFQRFMNMTGTFNESLSKDAKGLLGLYEASFHGLDGETILDEAWNFASKHLKDLNLDKVPSNLASNVSHALDMPIHWRPNRLEARWFMDMYEKQQDMIPSLLRLAKIDFNLVQSIHRKEVGNLARWWVELGANKMTFFRDRLVESYFWTCIMVFEPQYTAFREMCTKIGFMVTLIDDVYDIYGTPEELELLTDFIIRWDITDIDKLPPTIRNSFMVLYNTTNEVGYQTMRDQGINPIPYLRKVWADECKAYMKEVHWYNSGIKPALKEYMDVAVDSIGGLILLLHSYFLTTDKLTKEGLDHVSKIPSVMHSSTKILRFNDDLSTSLHELARGDNSKALECYMNESGASEEVAREHIRHLVRNIWKKMNKDVFEDYPFSGFGPFLGACLNLARASHCFYEYGDGHGLPGHQTKDHLVSTIFESVPL

>EgranTPS086

TCAATIENPEIVRRSANWKPNVWDYEFLAEQVQRLKEEIKGLFNREMNQVAKLEFIDVVQRLGLGYHFETEIKNALSSIYDNTGYAQLLNDLYAISLGFRLLRQHGYNIRQDVFQQFMNKTGTFNESLNKDVKGLLGLYEASFHGLEGETMLDEARNFASKHLKDLNLDKVPTMLASYVSHTLDIPIHWRPNRLEARWFMDMYEKQQDMIPSLLRLAKLDFNLVQSVHKKEVSNMARWWVELGANKMTFFRDRLVEHYFWNCTMVFEPQYTAYREMTTKLACMVTLIDDVYDVYGTLEELELLTDFLVRWDITEIDKLPPTIRDSYMALYNTTNEIGYWTMRELGINTIPYMRKVWADECKAYIKEAHWYNKGIKPTLKEYMDNAVDSIGGSIMLLGSYFLTTDKLTEEGLDYVSKIPSVMHCSTKILRLNNDLSTSSYELARGDNFKALECYMNETGASEEAAREHVKQMVHETWKRMNKDVFEDYPYSGLGPFLDACLNFARASQCFYQYGDGHGLPDNETKDHLVRALFDPVPL

>EgranTPS087

QAVDQTHQRRSANYKPNIWKYDFLEQRMERLMEDVKPMFPKAVDSLAKLELIDRMRKMGLSNLFDNEMKEALETVASTKNGIFDMENHVYARALRFRLLRQHGYVVSQDEMRSFKEESKTFNRSNCEDVEAMMQLLEASHLAVEGENILDEGKAFSTGILRERVSSLDGRLLKCAVHALELPMHRRLQWFDVKWQIDLYEQQEDKQSNLLELAKLNFNTVQATHRRDLIEISRWWRDLGLIEHVDFTRDRPVESFLCALGLSQEPRFSSLRKSLTKVIIFILVIDDVYDLYGSLEELECFTSVVTRWDSEPIQQLPECMKFCFRALHDLTYEIAHEIGKEEDWHRLPYLMKAWADFCKALLTEAKWDHLGYTPSLEEYLSNAWTSSSGPLLLSHAYFFVGHMKLEDAAELAEIERGDAPSSMVCYMREANVSEDIARKHIKGLIDQAWKNINAHCFVNAETPFLRPYIDVTVNAARAAYMIYQSGDGFGVQDGTIGQQMLSAVIEPLAL

>EgranTPS088

VNLPEAVGTMHQRRSANYKPNIWNYDFLKERMQRLVEEVKPMLSEVVDSLAKLELIDSMTKLGLSNLFENEMKEALETVASINNGVFTMEEHLYASALQFILLRQHGHIISQDELRSFKEGSILFNRSNCEDVEAMIELLEASHLALEGENILHEAKAFSTGILHERVSGLDSRLFKCAVHALELPMHWRVQWFDIKWQISLYEQREDKQSNLLELAKLNFNTVQATHQRDFREISRWWRDLGLMEHVDFTRDRLVESFLCALGLSQEPRFSSLRKSLTKVVILILVIDDVYDLYGSLEELECFTCAITRRDSEQIQQLPECMKVCFRVLNDVIHEIAYDIGKDEDWHRLPHLWAEFCKALLTEAKWDNMGSTPSLEEYLSNAWTSSSGPLIMSHASFFVGHMNWEDVADLLERNKDLIYNVSMIIRLCNDLGTSRAERDRGDAPSSVVCYMREANVPEDIARKHIKELINQEWKSINAHCYSNAETPFVRTFIDVTVNAARVAHMLYQFGDGFGVQDGDIRRQILSAVIHPLAL

>EgranTPS089

MTDGEISISAYDTAWVALVEDVSGSGSPQFPEALRWIVDNQLPDGSWGDDLIFSPHDRIINTLACVNNLEGMEGLNWEKLLKLQSADGSFLFSPSSTAFALMQTKDANCLDYLSRAVQRFNGGVPNVYPVDLFEHLWAVDRLQRLGVSRYFKDEIKGCMSYVQRYWSEKGICWARNSRISDIDDTAMGFRLLRLHGHEVSADVFEQFKRGDVFSTFMGQSTEAVTGMFNLYRASELIFPGEKILEDAKSHAVKFLRRKREANELLDKWIRTKDLPGEVGYALDVPWYASLPRVETRFYIDQYGGENDVWIGKTLYRMGYVNNNVYLELAKLDYNNCQALHLSEWDNFQRWYCESKLVDFGTSGKTLLYSYFTAAASIYEPERARERLAWAKTSVLVDAIASYLEGEGTSRERRRGFVREFQQFSKKQKHINGRKYCSRSSIPSKGGQGLIAALLGTLDQLSLEVLVARGKDIGHALRTAWEKWLVGYEEEGDKYKGVVELLVQMIALGSGNSSFSDGRSSHPQYRHLCNLTNTICRHLAHRQTQKACENEDLPIQMAMQELVQLVLQDSAHRLDRDVKDAFFSVTRSFYYTAHCDPATIKSHIAKVLYER

>EgranTPS090

IDVPIMKWQEIGEDDLGVEGVKVASPSSEIGERVKAIKLMFWSMSDGKISTSAYDTAWVALVEDVGGNGNPQFPEALQWIANNQHPDGSWGDDLLFCPHDRILNTLACVVALKYWKIHPDKCDKGMSFFKDNISKLAEEKPEHMPIGFEVTFPSLIETAWKLEIEICDDSPVFREIYANRNTKLNKIPSDLLHQVPTSLLHSLEGMKELKWEKLLKLKNADGSFLFSPSATAFAFLQTRDLNCLNYLSRTVQRFHGGVPNVYPVDLFEHLWAVDRLQRLGVSRYFKEEIKECMSYVYRYWSDKGISWARNSNVADIDDTSMGFKLLRLHGYEVSAEVFENFKSGKEFFSFMGQLIVPVTGMLNLYKASELIFPGEKILEDANRHAAKFLRRKQEANELFDKWIITKDLPGEVGYALDVPWYANLPRVESRFYIDQCGGQNDVWIGKTLYRMEYVDNDVYLELAKLDYNNCQALHLSEWDNFQGWYCESKLVDFGISGKTLLYSYFTAAASIYEPERAGERLAWAKTRVLVDAIASYLEREGATREQRGAFVRAFQQFSRKQKHTSGRSSILDERGEGLIAALLGTVDQLSLEVLEARGKDISHPLRAAWEKWLVGYGEEGDKYKGVVELLVQMIALGACNSLLSDDLSSHPECHRLCNLTSTICRQLAHHQTQKVRKNENDQIEMAMQELAQLVLQNSTRGLNRDVKDAFFAVTRSFYYTAHCDTGTINSHIAKVLFERVN

>EgranTPS091

MSVLLLQLSEVSKERVRDMSNKVELSISSYDTAWVAMVPSPGSPKSHRFPRSLILGVSPTIIRNRHRLLIKDALLSTIACVLALEQWGVGERQINCGLEFNILLSSMIEQAGRLNLNLPLRSANLDSMSYERNLELKRNGESDWEMIKKHQRKNGSLFNLPSTSAAALTHLQNVGCLRYLKLVVEKFGDAAPTIYPLGIYARLCMTENLERLGINCHFRKEIIDTLDDTYRCWLPGEENIFLDVATSAMAFRILRSHGYDVSSDALSQFAEEDQFYNTLEGHEKDAGWVKLAYCYFSAAETFLARQLLDTHISWAKNSVVVTVADDLFDVSGSAEGNEVLIRLLRKWNVNLSADGCSEQCQQWMNTLLMHVSFGLGPTVLPALYLVGPKLSEKQVESPEYHNLFRLMSTCRRLLNDIQSYERESKQGKLHAVTLQMLDGSGTSEREAIERISSIIISMRRELLKLVLQEKDSIIPRVCKDLSWKMSAVMHLFYMDADGFAPDEKTSAVKALLDQPITFNEL

>EgranTPS092

MSFSSYGNGQLTDAGCRGEEEIFQHIATSAMAFRILCSHGYNVSSDNLFSCAIDQWKVDDALRFPLEESLTRAANRRNIQLHNTNSTRILKCKLRSHNFHNKDFLNLAMHDFNMCQAICQDMETWGGDVSADCCSEETGCKLIAWQGRDVTGHRRTKAVPKMDEYMAHPSHLVWALSFSWLAGPRLSEKLIKSLEFHYFFGAVSTFGHLWNNRWGAEEGKPTAVMMQMLDGSSTSQEEAIQRISQMAVDETRKLLKLVLQEKESIVPRPCKDLFWNLIAVLHFFYREEDGFSSP

>EgranTPS093

MTHSHFSSLGLSAPISGAFPLDFGLKSVKQSSSCVAVALGEGSKERISDMFNKVELSLSSYDTAWVAMVPSPCSPQSPLFPRSVSWLMNNQLCDGSWGLPDHHPLLIKDALLSTLACVLALKQWGVGERQINKGLEYIASNSASVIDDRQHTPIGFNILLSGMIEQADCLNLNLPLRPADVDSVSYKRNLEVKRGLSGISGRYLSYVAEGMGSSADWEMIMKYQRKNGSLFNSPSTTAAALTHLQNASCLHYLESVLEKFGDAVPTIYPLEIYARLCMIENLERLGIDRYFRKEVIHVLDDTYRCWLQGKEEIFLDIATTAMAFRMLRSHGYDVSSDALNQYAEEDQFCNTLEGYVKDAGSVLELYRASQLIINDDKIILDKINSWTYDFLRKGLHTGKMHANRLESYICGEVDDALKFPLHANLERVANRRNIELYNIDSTRVLKCGLRSCNFCNKDFLNLAVNDFNNCQAIYQEELKYLERWVKEKRLDKLKFARQKLAYCYFSAAASFSPPQLSEARISWAKNGVLTTVVDDFFDVGGSAEELENFIWLVKRWNVNLSADCCSEQVQIIFSALHSTISEIGDKAVTWQGRNVTGHVAQIWLELLESMLTEAKWTRKKAVPTMDEYMANAYVSFALGPIVLPALYLVGPKLSEEQVESLEYHKLFKLMSTCGRLLNDMQGFKRESREGKLNAVTLQMLDRSSTSEREATERISSIIISKRRELLKLVLQERDSIVPRACKDLFWKMSTVLHFFYMDDDGFTSDEKTSAVKALLDQPITLNELELP

>EgranTPS094

MESQKSSIQSLINMIKRDLLSVIGSHSFLSPSPYDTAWLAMIPDPRRHDRPMFEGCLNWVLHNQNEEGFWGYHDCDRHEMPDGVGCLASTLVCMIVLKKWYAGSPLIEKGLKFIHKNAELLLSRYTHGKFSRWIAIVLPGMVELARASSLEVVFPESVDRALEDLFINRRQILKMEELVDKNQYCPLLSYLEALPSTYKISHETILKHLDSDGSLFQSPSATSRAYLSTGNEACLAYLQSLASNCASSGIPSFYPVDEDLTKLSMVHQLVRLGLTEYFDRENDEILAKIYRLLYKDCLEFWLLRMHGYRVSPSSFCWFLDHEEIEHELGLPWMARLDHLEHRTWMEEKDACVLWMGKFQYNRPSFVHNQDIVQLALQNYVLRQSVYRMELDVVKRWSETTGLRKMGFGREKTLYSYFAVAASISLPCNSDVRVLVAKSAIMITVADDFFDMEGSLEDLEKLTNAVQRWDGEGLTAHAKTIFEALDDLVTDFRMKCFKQSGKDIKKNLQEIWGETFHSWLMEAKWSKSGGAPPTQEYLDVGMTSIAAHILVLPSSCLASPTTPLHQLWSNAYQPITKLLMVITRLLNDIQSYEAKAKSGRCSLSGACLLLLLLWTYIHSLILSPCFYDFLTLQKEEKQGKLNFVLLYLKENPEASIEDSINFVQLLLEQLKKEFLLHVLEELCNLPEPSRRLHLGCLKVFHMFFNSSNRYDSETGMLHDIQKALVVPPRVPKLKPLRPLPEQLGPKPREFVTKSLYGQVGLERFPRKSFVGYRISSRTGPVDRWEKMYKSSNFKLCFA

>EgranTPS095

MEFQKPSIQSLVQMIKREILPNMSSHSFLSSSPYNTAWLAMIHNPHQHDLPMFKGCLNWVLHNQNEEGFWGDYDHDEDEMSNKVECLASTLICMTMLKKWHVGLPSIEKGLKFIHENVELLLPGNKHGKFPRWIAIVLPGMVDLARASGLEVIFPESAEHIIADLFSNRQQILKREKLVDKKQYFPLLSYLEALPPTYKVSHETILKHLDSDGSLFQSPSATASAYLATGNEACLAYLQSLALNCASNGVPSLYLVDEELTKLSMVYQLVRLGLTEYFDREKDEFLAQIYRNYKHEKPIVKSIHSIAAELYKDCLGFWLLRMHGYKVSPSSVCWFLDREEVRDHIEKHYEYFSSVLLNIYRASNLMLPNEHELEKARTFSKKFLEKIASRGTRDSSIISSSHCRMIKHELGLPWMAHLDHLEHRMWMEEKDACVLWMGKFSCHRSSLVHNQDILQLALQNFEQRQSIYRMELDVLKRWSETTGLSKMGFGREKTVYSYFAVAASVSVPCNSDVRVLVAKGAVILTVADDFFDMEGSLEDLEKLTEAVQRWDGEGLSAHAKTIFEALDDLVTDFRMKCFKQLGKDIKKNLQNIWGETFHSWLIEAKWSRSGDVRPTQEYLDVAMTSVGAHVLVLPPSCLASPTTSLHQLWSNPYQPITKLLMVISRLLNDIQTYEKEEKQGKLNFVLLYLKENPGASIEDSINFVQLLLDQLKKEFLQHVLEEPCSVPELSRLLHLACLKVFNMFFNSSNRYDSDTDMLHDIQKALVVPPRVPKLKPLRPLPEKLRLKPRVFETKSLSGQYGLEHFPRKSFFGYQMSSRTGPVNRWEKMYKSSSFKLCFA

>EgranTPS096

MRGQIFLIESLVNTIKREILLSNIVSSSFLSPSPYNTAWLVMIPDPHWHDCPMLNVCLNWVLHNQNEEGFWGDYDYEEHEMSDGGECLASTLVCMTVLKKWHVGSTLIEKGLKFIHGNAELLFSRNKRGNSPRWIAIILPGMIDLARAVGLEIIFPESTESAIVDLFRNRQRILEREKLVDRNQYYPLLSYLEALPPTYKISNETILKHVDGDGSLFQSPSATASAYLSTGHKACLAYLQSLASNCASNGIPSIYLVDKKLAKLSLVYQLVRLGLTEYFDQEKDDILAQIYRLEKPITKTVHWIATELYKDCLEFWLLRMHGYSVSPSSFCWFLDREEVRDHIEKHYEYFSSVLLNVYRASNLMLSDEQQLERVRTFSRKLLEKILSGETRDECIISSSHRRMIEHELGLPWMARLDHLEHRMWLEEKDACVLWMGKLSWHRSSLVHNQDILQLALQDFVLRQSTYRMELDVVKGWSETTGLSKMEFCREKTTYSYFAVAASISLPCNSDIREVVAKSAIIVTVADDFFDMEGSLEDLEQLTDAVQRWDGEGLSGHAETIFKALVDLVTDFRWGETFHSWLMEAKWSRSGGAPPMQEYLHVGMKSIATHILPITKFLMVITRLLNDIQSYQKEEKQGKLNFVLLYLKENPEASIEDSINFVQHLLHQLKKEFLQHVLAEPCSLPKPSRQLHLGCLKVFHMFFNSSNRYDSDMDMFHDIQKALVVPPRVPKLKSLMPLPEQPGPKPRVSVTKDLSSQFRLGRFPRKSFVGCQMPPHSGPATRWEKMYKLSSFKLCFA

>EgranTPS097

MVDLAHASGLEVVFPESVERIIADLFSNRQRILKREKLVDKKQYFPLLSYLEALPPTYKVSHETILKHLDSDGSLFQSPSATASAYLATGNEACLAYLQSLALNCASNGAVPSLYLVDEELTKLSMVYQLVRLGLTEYFDQEKDEILAQIYRNYKHEKSIVKSIHSIAAELYKDCLGFWLLRMHGYRVSPSSVCWFLDREEVRDHIEKHYEYFSSVLLNIYRASNLMLPNEHELEKARTFSKKFLEKIASRGTRDSSIISSSHCRMVSGNIQHFLSFSIIKHELGLPWMARLDHLEHRMWMEEKDACVLWMGKFSCHRSSLVQNQDILQLALQNFEQRQSIYRMELDVLKRWSETTGLSKMGFGREKTVYSYFAVAASVSVPCNSDVRVLVAKGAVILTVADDFFDMEGSLEDLEKLTEAVQRWDGEGLSAHAKTIFEALDDLVTDFRMKCFKQLGKDIKKNLQNIWGETFHSWLIEAKWSRSGDVRPTQEYLDVAMTSVGAHVLVLPPSCLASPTTSLHQLWSNPYQPITKLLMVITRLLNDIQTYEKEEKQGKLNFVLLYLKENPGASIEDSINFVQLLLHQLKKEFLQHVLEEPCSVPELSRLLHFACLKVFHMFFNSSNRYDSDTDMLPDIQKALVVPPRVPKLKPLRPLPQQPGPKPRVFETKSLSGQYGLERFPRKSFIGYRMSSQTGPVNRWEKMYKSSSFKLCFA

>EgranTPS098

MGSSSFLSPSSYDTAWLAMNPNLHQPNCPMFKGCLNWVLHNQNEEGFWGDYDYEEHEMSDGGKCLASTLVCRTVLKKWHVGSPLIEKGLKFIHENAELLCPKNKHGKFPRWIAIILPGMVDLARAVGLEIIFPESTERVVADLFSNRQRILEREKLVDRNQYYPLLSYLEALPPTYQISHETILKNLDSDGTLFQSPSATASAYLSTGNEACLAYLQSLASNCASNGAVPSLYLMDEELTKLSVVYQLMRLGLTEYFEQEKDEILAQIFRNCKYEKPIVKSIHLITTELYKDCLEFWLLRMHGYRVSPSSFCWFLDHEEVRDHTEKHYEYFSSVLLNIYRASNLMLPNEHELEKARTFSKKFLEKIAFRGTRDSSIILLSHRGMIKHELGLPWMARLDHLEHMMWMEEKDACVLWMGKFSCHRSSLVHNQDILQLALQNFVQRQSITRMELDVLKRWSETTGLSKMGFGREKTLYSYFAVAASVSVPRNSDVRVLVAKSAIIVTVADDFFDMEGSLEDLEKLTEAVQRWDGEGLSAHAKTIFEALDDLVTNIRTFHSWLIEAKWSRSGDVRPTQEYLDVAMTSVGAHVLVLPPSCLASPTTSLHQLWSNPYQPITKLLMVITRLLNDIQTYEKEEKQGKLNFVLLYLKENPGASIEDSINFVQLLLDQLKKEFIQHVLEEPCSVPELSRLLHLACLKVFHMFFNSSNRYDSDTDMLHDIQKVLVVPPRVPKLKPLRPLPEKLRLKPRVFETKSLSGQYGLEPFPRKSFFGYQMSSQTGPVNRWEKMYKSSSFKLRFA

>EgranTPS099

MVYQLVRLGLTEYFDQEKDEILARIYRNYKHEKPITKSIHGIAVELYKDCLEFWLLRMHGYCVSPLSFCWFLDHEEVRDHIEKHYEYFSNVLLNVYRASNLMLPDEHKLEKVRTFSKKFLEKIASGGTRGESIISSSHRKMIEHELSLPWMARLDHLEHRMWLEEKDAFILWMGKLSCHRSSLVHNQDILQLALHNFVLRQSIYRMELDVVKGWSETTGLSKMGFGREKTTYSYFAVATSVSLPCNSDIREVVAKSAIIITVAEDFFDMEGSLEDLEKLTNAVQRWDGEGLSGHAKTIFKAVVDLVTDFRVKCFKQSGKDIKKILQDIWGETFHSWLMEAKWSRSGGAPSTQEYLDMGMTSIAAHILVLPSSCLASPTTLLHQLCSSPYQPITKLLMVITRLLNDIQSYQKEEKQGKLNFVLLYLKENPKASIEDSINFVQYLLEQLKKEFLKHVLEEPCSLPKPSRRLHLGCLKVFHMFFNSSNRYDSDMDMFHDIQKALVVPPLIPKLKSPMPLPEQLGPKPR

>EgranTPS100

MIDLARAVGLEIIFHESTECAIVDLFRNRQQILEREKLVDRNQYYPLLSYLEALPPTYKISNETILKRVDGDGSLFQSPSATASAYLSTGHKACLAYLQSLASNCASNGIPSIYLVDEKLAKLSLVYQLVRLGLTEYFDREKDDILAQIYSFCWFLDREEVRDHIEKHYEYFSSVLLNVYRASNLMLSDEQQLERARTFSRKLLEKILSGETRDECIISSSHRRMIEHELGLPWMARLDHLEHRMWLEEKDACVLWMGKLSWHRSSLVQNQDILQLALQDFVLRQSAYRMELDVVKGWSETTGLSKMEFCREKTTYSYFAVSTSISLPCNSDMREVVAKSAIIVTVADDFFDMEGSLEDLEKLTNAVQRWDGEGLSGHAETIFKALVDLVTDFRAKCFKQSGKDIKKNLQDIWSETFHSWLMEAKWSRSGGAPTMQEYLDVGMTSIATHILVLPSSCLASPTTPLHQLWSSPYQPITKLLMVITRLLNDIQSYQKEEKQGKLNFVLLYLKENPQASIEDSINFVQHLLDQLKKEFLQHVLEEPCSLPKPSRRLHLGCLKVFHMFFNSSNRYDSDMDMFHDIQKALVVPPRVPKLKSPMPLPEQPRPKPRVSVTKDLSGQFRLGRFPRKSFVGCQMPSHSGPANRWEKMYKLSSFKLCFA

>EgranTPS101

RGRHERRLEEVKQLLKQVRGDSLESLVTVDALRRLAIDYHFEDEIEAILQRHLLISSSRSHSRPIDADNLHEAALRFRLLRQAGYPGNDILGFTSLFEASQLGIEGEDALDQVGESTRQRLHSSLADLDHVQTRFVRNSLGNPFHKSLARFTANDFLRNFVGHSSWTKNLGELAHLDMNIVRSVHQREILQVSNWWKELGMAKELKYARNQPMKWYIWPMAILTETGLSQERVLVTKPISFIYIIDDIFDVYGTIGDLTAFTDVVNRWECTEKDNIPDYMRTCFHALDDITNEFSLAVYKNQGGNPLCPLRKPWASLLNAFLVEARWLASGHSPTTQDYLDNAIVSSGVHVLLVHLFFILGERITPESVDHLENIPEIVSSTASILRLWDDLGSAEDEFQDGRDGSYVECYKREFGSSEEAARDHVKKMISEAWKRLNKACLYPQTFTKSFSRASLNTARMVPLMYNYDDSHSLPLLEHHMKSLLFGGPTF

>EgranTPS102

RGRHERRLEEVKQLLKQVRGDSLESLVTVDALQRLAIDYHFEDEIEAILQRHLLISSSRSHSRPIDADNLHEAALRFRLLRQGGYPVPSDVFQRFLHKGTQNKKPQGNDILGFTSLFEASQLGIEGEDALDQVGESTRQRLHSSLADLDHVQARFVRNSLGNPFHKSLARFTANDFLRNFVGHSSWTKNLGELAHLDMNIVRSVHQREILQVSSWWKELGMAKELKYARNQPMKWYMWPMAILTETGLSQERVLVTKPISFIYIIDDIFDVYGTIGDLTAFTDVVNRWECTEKDNIPDYMRMCFHALDDITNEFSLAVYKNHGWNPLCSLRKTTTQDSLDYAIVSSGVHVVFVQAFFILGERITESVDHLENIPEIVSSTASILRLWDDLGSAEFQDGRDGSYVECYKREFGSSEEAARDHVKKMISEAWKRLNKACLYPQPFTNSFSKASLNTARMVPLMYNYDDSHSLPLLEHHMKSLLFKGPTF

>EgranTPS103

GIKHDQKIKSLIDFLNKVVNEPVESLIIVDMIQRLGVKSLFREQIKAILAWQYTHFSSLNHGKDDVYEIALRFRLLRQEGYCVPADVFEYFNEKGKGFVMKLEGNIKGMMELYEASQMSTEGEDILDEAECFSSKCLNALLTCDLDNEQARMIESTLQYPYRKSFARLAPQSFVNDMQGANSWMEDLLEVANRKRRIDQYVHQKEIHQINRWWKELGLGEKMEFARDQPLKWYMWSMAILTDPSLSELRVELSKPISLVYIIDDIFDVHGTVDELILFTELIKWDNACAEQLPEYMKICFKVLNDIANDFGRIIFEKHGWNPTRFLKQMWANLCNAFLVEFQWNASGKLPKADDYLKNAIITSGVPLVLTHLLFLMGQNIANQSMDSKKEELPNIIFLIAEILRLWDDLGSMKDENQNGYDGSYVDCYLGENGSSYQSAREHVMKLISKLWKLLNKECLSPCPSSAPFLEACVNAAKMVSLMYNYEDKHGLGLLQDHMKSLTCDHETL

>EgranTPS104

LPRTILAQLERETLPPRFVCPFFSCNSFCSAHRRFRKFHSRLLRQGGYPVPSDVFQRFLHKGTQNKKPQGNDILGFTSLFEASQLGIEGEDALDQVGESTRQRLHSSLADLDHVQARFVSNSLGNPFHKSLARFTANDFLRNFVGHSSWTKNLGELAHLDMNIVRSVHQREILQVSSWWKELGMAKELKYARDQPMKWYMWPMAILTETGLSQERVLVTKPISFIYIIDDIFDVYGTIEDLTAFTDVVDRWECTEKDNIPDYMRMCFHALDDITNEFSLAVYKNHGWNPLCSLRKTWASLLNAFLVEARWLASGHSPTTQDYLDNAIVSSGVHVLLVHLFFILGERITPESVDHLENIPEIVSSTASILRLWDDLGSAEFQDGRDGSYVECYKREFGSSEEAARDHVKKMISEAWKRLNKACLYPQPFTKSFSKASLNTARMVPLMYNYDDSHSLPLLEHHMKSLLFKGPTF

>EgranTPS105

GTKHEEKIKSIIGFLNNVGGEPVESLIIVDMIQRLGIKPLFQEQIKAILRWQYTHFTSLNQGKDNVYEIALRFRLRQEGYDVPCDVFEYFKDKGKGFIMKVEGNVKGMMELYEASQMSIEGDDILDEAKCFSSKCLNELLTCDLDPEQVRMIESTLRYPYRKSFARLAPLSFVNDMPGVNSWIEDLLEVANRERRIVQSMHQKENHQINRWWKESGLGEEMKFARDQPLKWYLWSIAILTDPGLSELRVELVKPISLVYIIDDIFDVYGKVDELILFTGVIKWDDACAEQLPEYMKKCFKVLSDITNDFGNIIFEKHGWNPTRFLKQMWANLCNAFLVEFQWNASGTLPKADDYLKNGIITSGVPLVLAHLFFQMGQNIANQSMDSKKEELPNTIFLVAEILRLWDDLGSSKNENQNGYDGSYVECYLRENGSSYQSAREHVMELISKSWKLLNKECLSPCPFSAPFLEACVNAAKMVSLMYNYEDKNGLGLLQDHMKSLTCDHETL

>EgranTPS106

GIKHDEKTKSLIDFLNKVVNEPVESLIIVDMIQRLGVESLFREQIKAILKWQYTHFSSLNHGKDDVYEIALWFRLLRQEGYRVPADVFEYFNDKGKGFVMKLEGNIKGMMELYKASQMSTEEEDILDEAECFSSKCLNALLTCDLDHEQARMIESTLQYPYRKSFARLAPQSFVNDMPGANSWMEDLLEVANKKRRIDQFVHQKEIHQINRWWKELGLGEKMKCARDQPLKWYMWSMAILTDPSLSELRVELIKPISLVYIIDDIFDVHGTVDGLILFTEAIKWDIACAEQLPEYMKKCFKVLNDIANDFGKIIFEKHRWNPTRFLKHMWANLCNAFLVEFQWNASGKLPKADDYLKNAIITSGVPLVLTHLFFLMGQNIANQSMDSKKEELPNIIFLMAEILRLWDDLGSLKDENQNGYDGSYVDCYLRENGSSYQSASEHVMKLISKLWKLLNKECLSPCPFSVPFLEACVNAAKMVSLMYNYEDKHGLGLLQDHMKSLTCDHETL

>EgranTPS107

RIKHDEKTKSLIDFLNKVVNEPVESLIIVDMIQRLGVKSLFREHIKAILRWQYTHFSSLNHGKDDVYEIALRFRLLRQEGYHVPADVFEYFNDKGKGFVMKLEGNIKGMMELYEASQMSTEGEDILDEAGCFSSKCLNALLTCDLDHEQARMIESTLPYPYRKSFARLAPQSFVNDMPGANSWMEDLLEVANRKRRIDQYVHQEEIHQINRWWKELGLGEKMEFARDQPLKWYMWSMAILTDPSLSELRVELIKPISLVYIIDDIFDVHGTVDELILFTEVIKWDNACAEQLPEYMKICFKVLNDIANDFGKIIFEKHGWNPTRFLKQMWANLCNAFLVEFQWNASGKLPKADDYLKNAIITSGVPLVLTHLFFLMGQNIANQSMDSKKEELPNIIFLVAEILRLWDDLGSLKDENQNGYDGSYVECYLRENGSSYQSAREHVMKLTSKLCKLLNKECLSPCPFSAPFLEACVNAAKMVSLMYTMKTNMVLDSYKTI

>EgranTPS108

RLLRQEGYYVPADVFEYFKDKGKGFIMKLEGNAKGMMELYEASQMSIEGVDILDEAECFSSKCLNELLTCDLDPEQVRMIESTLRYPYRKSFARLAPLSFVNDMLVANSWMEDLLEVANRERRIIQSLHRKEIHQINRWWKELGLGEEMEFVRDQPLKWYMWSMAILTDPSLSELRVELIKPISLVYIIDDIFDVYGKADELILFTEVIERWDNACAEQLPQYMKKCFKVLSDITNDLGNIIFEKHGRNPTRFLKQMWANLCNAFLVEFQWNASGKMPKADDYLKNAIITSGVPLVLAHLFFLMGQNIANQSMDSKKEELPNTIFLVAEILRLWDDLGSLKDENQNGYDGSYVECYLRENGSSYQSARKHVMELISKSWKLLNKECLSPCPFSAPFLEACVNAAKMVSLMYNYEDKHGLGLLQDHMKSLTCDHETL

>EgranTPS109

GIKHEEKIKSIIGFLNNVGGEPVESLIIVNMIQRLGIKPLFQEQIKAILRWQYTHFTSLNHGKDNVYEIALRFRLLRQEGYDVPADVFEYFKDKGKGFIMKVEGNVKGMMELYEASQMSIEGEDILDEAECFSSKCLNELLTCDLDPEQVRMIESTLRYPYRKSFARLAPLSFVNDMLGLNSWIEDLLEVANRERRIVQSVHQKENHQINRWWKESGLGEEMKFARDQPLKWYLWSIAILTDPSLSELRVELVKPISLVYIIDDIFDVYGKVDELILFTGVIKWDDACAEQLPEYMKKCFKVLSDITNDFGNIIFEKHGWNPTRFLKQMWANLCNAFLVEFQWNASGTLPKADDYLKNGIITSGVPLVLTHLFFLMGQNIANQSMDSKKEELPNTIFLVAEILRLWDDLGSSKDENQNGYDGSYVECYLRENGSSYQSAREHVMELISKSWKLLNKECLSPCPFSAPFLEACVNAAKMVSLMYNYEDKHGLGLLQTI

>EgranTPS110

RIKHDEKTKSLIDFLNKVVNEPVESLIIVDMIQRLGVKSLFREQIKAILRWQYTHFSSLNHGKDDVYEIALRFRLLRQEGYHVPADVFEYFNDKGKGFVMKLEGNIKGMMELYEASQMSMEGEDILDEAGCFSSNCLNALLTCDLDHEQARMIESTLQYPYRKSFARLAPQSFVNDMPGANSWMEDSLEVANRKRRIDQYVHQEEIHQINRWWKELGLGEKMEFARDQPLKWYMWSVAILTDPSLSELRVELIKPISLVYIIDDIFDVHGTVDELILFTEVIKWDNACAEQLPEYMKICFKVLNDIANDFGKIIFEKHGWNPTRFLKQMWANLCNAFLVEFQWNASGKLPKADDYLKNAIITSGVPLVLTHLFFLMGQNIANQSMDSKKEELPNIIFLVAEILRLWDDLGSLKDENQNGYDGSYVECYLRENGSSYQSAREHVMKLISKLWKLLNKECLSPCPFSAPFLEACVNAAKMVSLMYNYEDKHGLGLLQDHMKSLTCDHETI

>EgranTPS111

RIKHDEKTKSLIDFLNKVVNEPVESLIIVDMIQRLGVKSLFREQIKAILRWQYTHFSSLNHGKDDVYEIALRFRLLRQEGYHVPADVFEYFNDKGKGFVMKLEGNIKGMMELYEASQMSMEGEDILDEAGCFSSNYLNALLTCDLDHEQARMIESTLQYPYRKSFARLAPQSFVNDMLGANSWMEDLLEVANRKRRIDQYVHQEEIHQINKWWKELGLGEKMEFARDQPLKWYMWSMAILTDPSLSELRVELIKPISLVYIIDDIFDVHGTVDELILFTEVIKWDNACAEQLPEYMKICFKVLNDIANDFGKIIFEKHGWNPTRFLKQMWANLCNAFLVEFQWNASGKLPKADDYLKNAIITSGVPLVLTHLFFLMGQNIANQSMDSKKEELPNIIFLVAEILRLWDDLGSLKDENQNGYDGSYVECYLRENGSSYQSAREHVMKLISKWKLLNKECLSPCPFSAPFLEACVNAAKMVSLMYNYEDKHGLGLLQHHMKSLTCDHETI

>EgranTPS112

RGRHERRLEEVKRLLKQVRGDSLESLVMVDALQRLAIDYHFEDEIEAILQGHLLISSSRSHSRPIDADNLHEAALRFRLLRQGGYPVPSDVFQRFLHKGTQNKKPQGNDILGFTSLFEASQLGIEGEDALDQVGESTRQRLHSSLADLDHVRDRFVRNSLGNPFHKSLARFTANDFLRNFVGHSSWTKNLGELAHLDMNIVRSVHQREILQVSSWWKELGMAKELKYARDQPMKWYMWPMAILTETGLSQERVLVTKPISFIYIIDDIFDVYGTIEDLTAFTDVVNRWECTEKDNIPDYMRMCFHALDDITNEFSLAVYKNHGWNPLCSLRKTWASLLNAFLVEARWLASGHSPTTQDYLDNAIVSSGVHVLLVHLFFILGERITPESVDHLENIPEIVSSTASILRLWDDLGSAEFQDGRDGSYVECYKREFGSSEEAARDHVKKMISEAWKRLNKACLYPQPFTNSFSKASLNTARMVPLMYNYDDSHSLPLLEHHMKSLLFKGPTF

>EgranTPS113

MIESALQYPYRKSFARLAPQSFVNDMSGVKSWMEDLLEVANRNRRIDQSVHQKEIHQINKWWKELGLGEKMKFARDQPLKWYMWSMAILTNPSLSELRVELIKPISLVYIIDDIFDVHGMVDELILFTEVIKRWDYACAEQLPEYMKKCFKVLNDIANDFGKIIFEKHGWNPTRFLKHMWANLCNAFLVEFQWNASGKLPKADDYLKNGIITSGVPLVLTHLFFLMGQNIANQSMDSKKEELPNIIFLIAEILRLWDDLGCAQDENQNGYDGSYVDCYLGENGSSYQSAREHVMKLISKLWKLLNKECLSPCPSSAPFLEACVNAAKMVSLMYNYEDKRGLGLLQDHMKSLTCDHETI

>Os01g23530

NDKDLNLVSLRFYLLRKNGYNMSSGIFLSFKDNEGNFIVDDTRSLLNLYNAANLRVYGEKVLDEAATFTISRLEGVLESSDSILSEVSFALEAPIFRRARIVEMRNYIPIYEIEATRNETILEFAKLNFNLLQLLYCEELNKITLWWKELKVKSNLSFSRDRIVEMYFWMNGALYEPHYSHSRIILTRVTAFMTIIDDIFDTYGTTEESMLLAEAINRWDESAIGLPEYIRGFYAYLLKTFDSFEEELGPEKRYRLKRLVQAYTKELKWRDEDYTPKTLEEHFEVSMRSSGGFTLAAASFVGMDDIATKDIFEWILSYPSLFKTFDIFVRLSNDIVSNKREQTGDHYASTIQCYMKEHGTTIHETYQRLRELIEDSWKDMVEHCTDQPLIVPQTVVNFARTVTTMY

>Os01g42610

YSSNLHEVALRFRLLRQQGYWVSPDEFNKFKCEDGSFKSDINNDPKGLLSLYHAAYLLTHNERALKEAILFATHHLELLSGSLEFPLAEQVKRALQIPLPRTLKRVEALNFIFEWWKDVSSDINLDYTRDRVVECYFCAYIVYYEKEYARARMMLAKKIMLISLLDDTYDVHATLEEARKFNEALQRWDKNAVSLVPEGLKRFFLSIMSNFRDFEDELEPHEKYRNAYNIKAFQILSNNYLQEAEWFHQKYIPSFTEHATVSLVTGGAIELPVSIIVGMGDIATKDAFDWALSYADAGRAFGEVSRFMDDLAVSQNGREKMDVANAVECYMKEHGVTSDVAEAEISEMVEGAWRTLNQARFVYLPFVQRIANVSMSIALLF

>Os02g02930

AHGGDLLDATLAFRLMREAGHHVSADEVGRFTDDNGEFRLDYRKDIRGLLSLQDISHMNIGQEASLCKAKEFSTRNLESAINYLEPNLARYVRQSLDHPYHVSLNQYKARHHLSYLQTLPIRCTAMEELALADFQLNKLLHQMEMQEIKRWWMDLGLAQEIPVARDQVQKWFVWMMTAIQGASLSRCRIELTKIVSFVYIVDDIFDLVGTREELSCFTQAIRMWDLAAADSLPSCMRSCFRALHTVTNDIADMVEREHGVNPINHLKKAWAMLFDGFMTETKWLSAGQVPDSEEYLRNGVVTSGVPLVFVHLLFMLGHDVSQNAAEFVDHIPPVISCPAKILRLWDDLGSAKDEAQEGLDGSYKELYLKENGLAAGEAEEHVRRLIAGEWEELNRECFTFPAGFTQAALNAARMVGVMY

>Os02g17780

ARNSNVKEVDDTAMAFRLLRLHGYNVSPSVFKNFEKDGEFFCFVGQSTQAVTGMYNLNRASQISFPGEDILQRARNFSYEFLREREAQGLHDKWIISKDLPGEVQYTLDFPWYASLPRVEARTYIGQYGGNDDVWIGKTLYRMPIVNNATYLELAKQDFNRCQALHQHELQGLQKWFIENGLEAFGMTPEDVLRAYFLAAACIFEPNRASERLAWARVSVLANTISRHFDMSSMKRMERFMWSSEENGNLGGYAGILARTLCQLIDLLSQETPPVREGQKCIHNLIRCAWIEWMMQQINMKDGRYDKGRVMHPGSCTVHNKETCLLIAQIVEICAGREMINNTEGSWFIQLASSICDSLHAKMLLSQDKKNETTINQIDKEIELGMQELAQYLLVDDRRINNKKQTFLSIVKSCYY

>Os02g36140

KDEEIMLDVTTCAMAFRLLRMNGYDVSSDELSHVAGASGFRDSLQGYLNDRKSVLEVYKTSKHSISENDLILDSIGSWSGSLLKEMLCSNGKGTPGREEIEFALKYPFYSTLERLVHRKNIVLFDAKGSQMLKTECMPVHDSQDFLALAVDDFCISQSNYQNELNYLESWVKDNRLDQLHFARQKITYCYLSGAATTFRPEMGYARTSWARTAWLTAVIDDLFDVGGLEQEQENLLALMEKWEEPGEDEYSEDVKIVFQALYNTVNEIGAKASALQGHDVTKYLVDVWLHVVRCMKVEAEWQRSQHLPTFEEYMESGMVSLGQGCTVMSALFLIGEKLPEGIVELEEYDELFRLMGTCGRLLNDIRGIEREESDGKMTNGVSLLVHASGMSVDEAKTEVMKRIDASRRKLLSLVVPIPRPCKQLFWKMCKILHLFY

>Os02g36210

GGLCPVKDIDDTAMAFRLLRLHGYNVSSSVFNHFEKDGEYFCFAGQSSQSLTAMYNSYRASQIVFPGDDDGLEQLRAYCRAFLEERRATGLMDKWVIANGLPSEVEYALDFPWKASLPRVETRVYLEQYGASEDAWIGKGLYRMTLVNNDLYLEAAKADFTNFQRLSRLEWLSLKRWYIRNNLQAHGVTEQSVLRAYFLAAANIFEPNRAAERLGWARTAILAEAIASHLRQYSANGAADGMTERLIDWDWESKDSAARSLLYALDELIDLHAFGNASDSLREAWKQWLMSWTNESQGSTGGDTALLLVRTIEICSGHGSAEQSLKNSADYARLEQIASSMCSKLAKILAQNGGSMDNVGIDQEVDVEMKELIQRVYGSSSNDSVTFLDVVKSFCYV

>Os02g36220

NEEELMLDMGTCAMAFRLLRMHGYDISSDGMAQFVEQSSFDDSIHGYLNDTKALLELYRSSQIRCLEDDLILQDIGSWSARVLQEKISSKMTHKSEMLEVEYALKFPVYATLERLEQKRNIEQFKTKEQLKIEGFKLLKSGYRGAITHDEILALAVDEFHSSQSVYQQELQDLNSWVAQTRLDELKFARLMPSITYFSAAATMFPSELSEARIAWTQNCILTTTVDDFFDGDGSKEEMENLVKLIEKWDGHGEIGSSECVEILFYAIYNTSKQIAEKAVPLQKRNVVDHIAESWWFTVRGMLTEAEWRMDKYVPTTVEEYMSAAVDSFALGPTITSAALFVGPELSEEVFRSKEYIHLMNLANTIGRLLNDMQTYEKEIKMGKVNSVMLHALRGSEASMEEAKREMRRVLQGSRCDLLRLVTVVPPPCRKLFWFMSKVLHFVY

>Os02g36264

NEEELMLDMRTCAMAFRLLRMHGYDITSDGMAQFVEQSSFDDSIHGYLNDTKALLELYKSSQLRCLEDDLILEEIGSWSARVLLEKISSKMIHISELPEVEYALKCPVYAILERLEQKRNIEQFKTKEQLKIEGFKLLKSGYRGVIPNDEILALAVDEFHSSQSVYQQELQDLNSWVAHTRLDELKFARLMPSITYFSAAAVLLPSESARIAWTQNCILTTTVDDFFDGEGSKEEMENLVKLIEKWDDHGEIGSSECVEILFYAVYNTSKQIAEKAMPLQKRNAVDHIAESWWFTVRGMLTEAEWRMDKYVPTTVEEYMSAAVDSFAVGPIITSAALFVGPELSEEVFRSEEYIHLMNLANTIGRLLNDMQTYEKEIKMGKVNSVMLHALRGSEASMEEAKREMRRVLQGCRFELLRLVTVVPPPCRKLFWLMSKVLHFVY

>Os03g22634

PSLHDVALRFRLLRQQGLWVSSDVFNKFKHRDGSFIIDITNDPKGLLSLYNAANLLTHNEEALQEAILFSRHHLELMKSNLKSPLAEQVSRALQIPLSRNLKRVEALSYILEYNVHEQTYNPSILELAKLDFNLLQHIHQRELKTITQWWEDLSNDIGLDYIRDRIVECYFWSYSMYFEEEYTRARMILAKFFMLTSLLDDTYDTHATLEECRNLNVAIQSWDESDISVLPDYLKKFFLKVMSNFVEFENELEPHIRHRNAYNRKVFQLLSGYYLQEAEWFHHNYVPSFKEQIEVSVMSAVTEETLEWAIGNNDAVRAGGEVARFMDDMAAFKNGRNKLDVASSVECYIKEYNVTSEVALAKIGSLVEDAWKTINQAHIRELLPFVHRVTNLSRSMAILF

>Os03g24640

DFAGSDDDLYTVALRFRLLRQHGVWVSACVRDKFRDGTCSFSSSLRDDPRGLLSLYNAAHMAAPVEIALDDIIAFARCHLEALSMEGELKSPLAEQVSRAPDIPLPRFPRRLETMSYLVEYEQEDEHDDMLLELARLEFELTRCLHLEELKALSLWWRELYESVKLSYARDRLVESYFWTCGVFHEEEYSRARIMFAKVFGLLSLMDDTYDVHATLEECYKLNEAIQRWDEGAISILPEYLRMFYIKLLSNFDELEASLEPHEKFRVSYAKNAFKLSSEYYLREAKWSNTKYTPSFAEHLEVSVMSSGFPMLAPVVLMGVHDVATAAAFEWATGVPDVVIAASGEVARFLNDIASHRVGKNEKDVPSSVESYMAEHGVGEEAALAAVAAMAEHGWRTINRALMEMDPGLLPAIVNLTRTLEVIY

>Os03g24680

GSDVADDDLHVTSLRFRLLRQHGLGISADVFDKFRDNGGSFRASLSSDTRGLLSLYNAAHLAMPGEEVLDDAIAFSRRHLRSMKTAGKLRSPMAEQVSRALDIPLPRTPRRLEAMRYIHEYGDEPGFDGVVLELARLDFELVKSLHLRELKALTLWWKDFYDNVKLSYTRDRIAEVFFWVSGVYYEEKYSRARIMLAKVFGLITLMDDTYDVQDTLDECCRFNETIQRWDNGAVSILPEYMHAYYIKLLSNFDEMENSLEPNEKHRVSYAITMYKQLSEYYLQEARWSSHRYLPSFAEHLYVSSISSGIPALAPAVLMGVHDVATKEALEWACAIPDLLLASGEEGRASLVETYMTEHGAGGDAAVAAVAAASERAWRRINRACVAVEPALLPALVNLTSTMEVVY

>Os03g24690

AGFDDGDQLYLESLRFRLLRQHGFWVSADVFDKFKDSTGCFRESLSTDARGLLSLYNAAHLAMPGEAALDDAIAFSRRSLQSLQGALRSPMAKQVSRALDIPLPRAPKLLETMHYITEYEQEAAHDGMVLELARLDFELVRSLYLKELKALSLWWRQLYDSVQLSYARDCLVESYFWTCAMFHGEDYSRARIIFAKVFQLMTMTDDIYDIHATLEECYKFNEAVQRWDKSAVSILPEYLRNFYIRILNDFDEMEDSLEPDEKHRMSYVKSSFKQQSEYYLREAQWSSDKHMPSFAEHLDVSFMSIGYPTMAVVVLLCARDAASMEASEWAPSLVRAGGEVTRFLNDIASYKTGKSGKDAASTIECYMAERGVGGEEAVAAVAALVESAWRTINRACVEMDPNLLPALVNLATTPEVIY

>Os03g31430

ANDDFHAISLQFRLLRQQRRYMPCDAFKEFIDKQGNLNGTLCSDTRALLALYEAAHLGTPNEEILREAQVETTNQLKRIVDCIEKPLSNKVRHALETPSFRRMKRLEARLYIPLYEEDKEECNEMILELAKLDFYLLQRLHREEVKEICEWYHGLESPRELFYARHRPAEAYFWALGVYYEPEYAKPRKLLAKFIATITPYDDTFDNYGLWKELQPFADVMQRWDEKGAEQLGRCYKEYAQFMFGTMNEIEGALPKGTPRKNVNVIKDIITEVCKGYVTEIDWRDSKYIPPLKEHLQITLVTCFYWAINCTAFVVFQEGVTEEVMIWMSGFPQIVKDSCIVSRLMDDIVAHAFETERNNVATAVTCYMKEYDSTKEEAIKALWNDVENAWKDMNEEYLSIPSSLLIQVSIMNNRVSKLAS

>Os04g09900

TKNCPVKDIDDTAMGFRLLRLYGYQVDPCVKKFEKDGKFFCLHGESNPSVTPMYNTYRASQLKFPGDDGVLGRAEVFCRSFLQDRRGSNMKDKWAIAKDIPGEVEYAMDYPWKASLPRIETRLYLDQYGGSGDVWIGKVLHRMTLFCNDLYLKAAKADFSNFQKECRVELNGLRRWYLRSNLEKFGGTDPQTLMTSYFLASANIFEANRAAERLGWARVALLADAVSSHFRNSTSNEELISLVPFDDAYSGSLREAWKQWLMAWTAKESSQESIEGDTAILLVRAIEIFGGRVLTGQRPDLWEYSQLEQLTSSICCKLSRVLAQENGESTEKVEEIDQQVDLEMQELTRRVLQGCSAINRLTRETFLHVVKSFCYV

>Os04g10060

RDEEVMLDLPTCAMAFRLLRMNGYGVSSDDLSHVAEASTFHNSVEGYLDDTKSLLELYKASKVSLSENEPILEKMGCWSGSLLKEKLCSDDIRGTPILREVEYALKFPFYATLEPLDHKWNIENFDARAYQKIKTKNMPCHVNEDLLALAAEDFSFCQSTYQNEIQHLESWEKENKLDQLEFTRKNLINSYLSAAATISPYELSDARIACAKSIALTLVADDFFDVGSSKEEQENLISLVEKWDQYHKVEYSENVKAVFFALYSTVNQLGAMASAVQNRDVTKYNVESWLDYLRSLATDAEWQRSKYVPTMEEYMKNSIVTFALGPTILIALYFMGQNLWEDIVKNAEYDELFRLMNTCGRLQNDIQSFERECKDGKLNSVSLLVLDSDMSVEEAKEAINESISSCRRELLRLVVVIPKSCKEMFWNLYKTSHVFY

>Os04g26960

TSSNLHEVALRFRLLRERGFWVSPDIFNKFKGDDGNFLNEFAEDPRSLLSLYNAAHLFIHGEPELEEAISFARNHLESMSSHSVLKAPLADQVKRHLRLPLPRTHKRVEMLHYMFEYDQENEHNPVLLELAKLDFNLLQQVHLKELKEISRWWKDVLAYMGLDHIRDRVIECYTWSYAVYHEKDLALARMIFAKLVALTSVLDDTYDVHASIEECRMLNVAIQGWDDSAALLVPEYLRKFYEIILRTFREFEDQIPRNQRYLAAFSKAEFQKLTSNYLEAAEWYHRNHKPSFNDQVALGTATTGTRSLAAGLMLGMGDATTKQAFQWAVTSTDAIISCGKIGRLMNDISGFKLGQNKADMACAVEAYIEEHKVTADVAIARINEVLEDEWKTTNQARVAVLPVVQRMTLGIQLFY

>Os04g27070

ASSKLHEVALRFRLLREHGFWVSPDVFNKFKGDHGTFNNELSLADDPRGLLSLYNAAHLFIHGEPELVEAISFARHHLESFNRRNVLKAPLADQVKRALHLPLPRTHRRVEMVSYMFEYGREDGHNPVILELAKLDFNLLQRVHLKELKEISRWWKDVSGYMGINHIRDRVIECYTWSYAVYHEEEMSFARMLFAKIVVIIALLDDTYDVHGSIQECRMLNAAIQGWDDSAVLLVPEYLRKFYEFILRCFREFEDQVPSNQKYLIAFSKTELQRLSSYYLEGAEWSHRKHMPSFSEQVALATMTTGTRPLAAGLMVGMSEMTTKQAYEWAVNSTDAIISCGKTGRFMNDIAGFKLGQNKADMPCSVESYINEHKVTADVAIAKINELVEDEWKTTNQARILPVVQRLINITMAIPLYY

>Os04g27190

TSSSLHEVALHFRLLREHGIWVSPDVFEKFKGEDGRFINTIADEPRALLSLYNAAHLLVHDEPELEEAMSFARHHLESMRDGSRLKAPLDNQINRALHLPLPRTYKRVEMLHYMLEYGQEEECIVVLLDLAKLEFNLLQHVHLKELKAFSQWWKDLYGYVELSHVRDRAVESYLWSYALFYEENLTLTRMILAKIIVFIVLMDDTYDDHATIEECRKLNEAIQRWDESAISLLPEYMKKFYRALLNYFRETEAQVEASDKYRVTCMKKEFQNLSTYYLQEFEWLHQNYKPAFKERVALSTLSSTVPLLCVTAAVGQGDAVTKESFELTTVRSSAVIACAKIMRFMNDIAAFKSGKNKGDAANTVECYINEHKVTSEVALDKIESMIESEWRTLNQVQKQFHVVQRVMNLAVAVPFFY

>Os04g27340

TSSSLHEVALHFRLLREHGIWVSPDVFEKFKGEDGRFINTIADEPRALLSLYNAAHLLVHDEPELEEAMSFARHHLESMRDGSRLKAPLDNQINRALHLPLPRTYKRVEMLHYMLEYGQEEECIVVLLDLAKLEFNLLQHVHLKELKAFSQWWKDLYGYVELSHVRDRAVESYLWSYALFYEENLTLTRMILAKIIVFIVLMDDTYDDHATIEECRKLNEAIQRWDESAISLLPEYMKKFYRALQNYFRETEAQVEASDKYRVTCMKKEFQNLSTYYLQEFEWLHQNYKPAFKERVALSTLSSTVPLLCVTAAVGQGDAVTKESFELTTVRSSAVIACAKIMRFMNDIAAFKSGKNKGDAANTVECYMNENKVTSEVALDKIESMIESEWRTLNQVHQQFPVVQRLLNLAVSVPFFY

>Os04g27400

ISSSLHDVALRFRLLRQHGFHVSPDVFNKFKGDDGRFVSGITNDPRGLLSLYNAAHLLTHDEPELEEAISFATQHLASLSSGTDLNPHLIDQINRALDVPLPRTYRRMETLCYMPEYRQEEGHIPILLELAMLDFNLLQHVHLKELKAISEWWKDLYGYMGLSYIRDRVVESYVWSYVVFYEEDSALARMIFTKIIAFIILMDDTYDSYATIQECRKLNEAIQRWDESATALPEYIKKFYSALLKTFKEFEIHVEDDGQYRIDHTKKAFQNLSAYYLQEAEWSYQNYKPSFEEQVALSTVTSTVPLLCVSTTVGRGDALTNEAFEWAANDIGAKIACAKITRFMNDIAAFQRGKNRGDVVSTVECYMNENKVTSEGAFTKIDLMIEDEWRTINQALCLPAVQQVLNLAICATFFY

>Os04g27540

TSSSLHEVALRFHLLREHGLWVSPDVFNKFKADDGKFIDEHNDLKQPLFDKFSRALHLPLPRTYKRVETLHYFLEYGQEEGHIPILLDLTKLDFNILQRVHLKELKAISEWWKDLYKYIGLTYISDRAVESYIWSHTMLFGEGLALTRMICAKIIILLVIMDDTYDAHATIEESRKLNEAIQRWDESAIPRVPEYLKKFYIKLLNNFKEIEDQVMDNEKYKVAYAKKEFQKLSHYYLQEVEWLHQNHKPSFQEQVDLSTKTSTAHLMFVSTTVGLGDAVTKEALEWAESSTAIVAVGKIMRFMNDIAAFKHGKNKGDVTSTMECYMNEHKVISDVAFMKLTSLIEHEYRTINQAELSLPAAQRVVVVSLMFFY

>Os04g27720

TSSSLYEVALRFRLLREHGFWVPPDAFNKFKGDDGRFRNEIANDPRGLLSLYNAAHLLIHGEPELEEAITFAREHLKLMSQDNVLNPPLACQVRRALTLPLPRTFKRVETICYMLEYQLEEGNIPILLDLARLDFNLLQHIHLKELKAISEWWKDLYGYMGLSYIRDRTIEGYTWSYMMFYEEGFAFTRMFVAKLIALCRWDKSAISILPEYLKKYYSKLLINFKEFQDQVTDNEKYMVACTKEEFQKQSTYYLQEAEWSNQKYKPGFKDQVVLSTKSSAVQLLCVAAMVGWGGTMTTEAFEWVASGNAAVIACAKIGRFMNDIAAFKRGKNKRDVASSVECYMNENGVTSEAAFAKINALVEDEWRSTNQTRLTLLPMVQRIVNFTVSMALFY

>Os04g27790

TSSSLHEVALRFRLLREHGLWVSPVTFNKFKGDDGRFMNGIADEPRGLLSLYNAAYLLVHDEPELEEAISFSRYHLKSMMQGNNLKHPLSDQVKRALNTPLPRTSKRTETLHYLSEYGQEEGHMSILLDLAKVEFNLLQGVHLKELKAISEWWRDLNEHVELSYLRDRVVESYTCSHMLFYEEGLAFTRITFTKIIVLIIMMDDTYDSHATIQECRKLNEAIQRWNESAVSVLPEYLKNFYHKLLNNFKEFENQVVVSEKYRVAHAKKEFQILSHYFLQEAEWSHNNYKPSFEEQLALSTKTSTVQLLCVSTTVGRGDAITNEAFMWAASSTTVTSCAKIMRFMNDIASFERGKNKGEIASTVECYMNEHNIISEVAFAKLDSLVEDEWRTINQARCQLLPVVQRVVNLAICIMFFY

>Os04g52210

RDEEVMLDITTCAMAFRILRMNGYDVSSDDLCHIAEVSDFHSSHQGYLSDTRTLLELYKASEVSVADNEFILDRIGSWSGRLLKEQLSSGALQRTSSIFEEVEHALDCPFYATLDRLVHKRNIEHFAAMSYISYAQNNIPDELERIDSWVKENRLHELKFARQKSAYFYLSAAGTVFDPEMSDARIWWAINGVLTTVVDDFFDVGGSREELENLISLVEMWDEHHKEEYSEQVEIVFFAIFNSVNQLGAKVSAVQGRDVTKHLIEIWLDLLRSMMTEVEWRISNYVPTPEEYMENAAMTFALGPIVLPALYLVGPKIPESVVRDSEYNELFRLMSTCGRLLNDVQTYEREDGEGKVNSVSLLVIQSGVSIEEARREIMKPIERCRRELLGLVLAVPGPCKELFWKMCKVCYFFY

>Os04g52230

RDEEIMLDITTCAMAFRLLRMNGYHVSSVELSPVAEASSFRESLQGYLNDKKSLIELYKASKVSKSENESILDSIGSWSGSLLKESVCSNGVKKAPIFEEMKYALKFPFYTTLDRLDHKRNIERFDAKDSQMLKTEYLLPHANQDILALAVEDFSSSQSIYQDELNYLECWVKDEKLDQLPFARQKLTYCYLSAAATIFPRELSEARIAWAKNGVLTTVVDDFFDLGGSKEELENLIALVEKWDGHQEEYSEQVRIVFSAIYTTVNQLGAKASALQGRDVTKHLTEIWLCLMRSMMTEAEWQRTKYVPTMEEYMANAVVSFALGPIVLPTLYFVGPKLQEDVVRDHEYNELFRLMSTCGRLLNDSQGFERESLEGKLNSVSLLVHHSGISIDEAKMKAQKSIDTSRRNLLRLVLAVPRPCKQLFWKMCKIVHMFY

>Os04g52240

RDEEIVLDMQTCGMAFRMLRMNGYDVSSDELSHFSEPSSFHNSLQGYLNDTRSLLELHKASKVSIAEKEVEYALEFPFYTILDRLDHKRNIEHFDITSSQMLETAYLPCHSNEEIMALGVRDFSSSQFIFQEELQQLNSWVKESRLDQLQFARQKLDYFYFSAAATIFTPELSDVRILWAKNGVLTTVVDDFFDVGGSKEELENLVALVEKWDKNDKTEYSEQVEIVFSAIYTSTNQLGSMASVVQGRDVTKHLVEISDILWNYFFSEVRTDKLNIVASVNLCIRIAEVYDDRGRVEAEPHCILLGPKMPDSVIRSQECSELFRLMSKCGRLLNDVQSYEREGSQGKLNSVSLLALHSGVSMEEAVKQIQRPIEKCRRELLKLVAVPRPCRELFWSMCKVCHFFY

>Os07g11790

NSSDLYEVALRFRLLRKQGYWVSPDEFNKFKAEDGSFSSDDITNDPKGLLSLYNAAHLLTHNEKALEEAILFARHHLQLLRGNLAYPLDEQVTRALEIPLPRTMKRVEVLNYIFEYSAEEKMFNPSILELAVLDFNILQKVHQNELKEICQWWENLSSDIRLDYVRERVVECYFCAYAAYYEKEHARARMIFAKRCMLFSLLDDTYDVRATLEEARKFNDALQRWDKSDVSLLPEDLKRFFLSIISNFREFEDELEPHEKYRNSYNIKAFQILSSNFLQEAEWFHQNYIPCFTDHVTVSLQTGGAIELPVSLIVGMGDIATKEVLDWALANPDAGRAFAEVARFMDDLAASHSGRDKMDVASTVECYMNEHGVTREVAEAKIAGMAEDGWKSMNQIRFAFLPFVQRIANLCMSATLLY

>Os08g04500

DTDNHYDLHTTALRFYLLRKHGYYASPDVFQRFRDEEGNFTRDDTRSMLSLYNAAHLRIHGEEILDDAIVFTRNYLQSVVKHLQSPMADEVCSALRTPLFRRPRRVEARHYISVYDKLPTRNETILEFAKLDFGILQSLYCEELNILTMWWKELQLQDHLSFARDRMVEMHFWMLGVLFEPQYSYGRTMLTKLFIFVSIFDDIYDNYSTLEESKLFTEAIERWDEEAAEELPGYMKFFYKKVLTTMKSIETDLKLQGNKHVDYVKNLLIDATRCFYNEVKWRSEDQVAATVEEHLKISVPSSCCMHVPVYAFVAMGDVTTDDAINWGMAYPKIITSSCIVGRLLNDIASHEREQGSSSSSSSVEACMREHGITKEEAYAKLRELVEESWMDIAGECLAQPPPLLEAVVNATRVLDFVY

>Os08g07080

NDKDLHLVSLRFYLLRKNGYDVSSDMFQHFKDKEGSFVADDVRSLLSLYNAAFLRTHGEKVLDEAIVFTTNRLRSELEHLKSPAADEVSLALNTPLFRRVRILEIRNYIPIYESATTRNESILEFAKLNFNLLQLIYCEELKSITGWWKELNVESNLSFIRDRIVEMHFWMIGACSEPHYSLSRIILTKMIAFITILDDIFDTYATTEESMMLAKAIYMCNETATVLLPKYMKDFYLYYLKTFDSFEEALCPNKSYRVCYLKELFKRLVQEFSQEIKWRDDHYIPKTIEEHLELSRKTVGAFELACASFVGMGDLVAKETLDCLLTYPELLKSFTTCVRLSNDIASTKREAGDHHHASTIQSYMLQHGATAHEACVGIKELIEDSWKDMMKEYLDLQPKIVARVIDFARTGDYMY

>Os08g07100

NDKDLHLVSLRFYLLRKNGYDVSSDIFQHFKDKEGSFVADDTRSLLSLYNAAYMRTHGEKVLDEAVVFTTNRLRSELKHLKSPVADEVSLALDTPLFRRVRIIETQNYIPIYESATTRNEAILEFAKLNVNLLQLIYCEELKTITRWWKELNVESNLSFIRDRIVEMHFWMTGACSEPHYSLLRIILTKMTAFITILDDIFDTYATTEESMMLAKAIYMCNESATVLLPKYMKDFYLYYLKTFDSFEEALGPNKSYRVLYFKELREQAGDHYASTIQCYMLQHGTTIHEACIGIKELIEDSWKDMMKEYLNLQPKIVARVIDFARTGDYIY

>Os11g28530

DEEEIMLDMATCAKAFRLLRMHGYDVSSEGMARFAERSSFDDSIHAYLNDTKPLLELYKSSQVHFLEEDFILENIGSWSAKLLKQQLSFNKISKSLMPEVEYALKYPFYATVEVLEHKGNIERFNVNGFQRLKSGYCGSGADKEILALAVNKFHYAQSVYQQELRYLESWVAEFRLDELKFARVIPLQSLLSAVVPLFPCELSDARIAWSQNAILTAVVDDLFDGGGSMEEMLNLVALFDKWDDHGEIGCSSNVEIMFNAVYNTTKRIGAKAALVQKRCVIDHIAEQWQVMVRAMLTEAEWAAGKHIPATMGEYMSVAEPSFALGPIVPVSAYLLGEELPEEAVRSPEYGRLLGLASAVGRLLNDVMTYEKEMGTGKLNSVVLLQPASRGASVEAARAEVRRAIQASWRDLHGLVFIIPRPCREVFWHTGKVASVFY

>Os12g30824

NEEEVMLDIPTCAMAFRLLRTHGYDITSDEMAHFSEQSSFDDSIHGYLNDTKTLLELFKTSQIRFSCEDLVLENIGTWSAKLLKQQLLSNKLSTSAQEVEYVLKFPLHSTLDRLEHRRNIEQFKVEGSKVLKSGYCGSHSNEEILALAVDYFHSSQSVYQQELKYFESWVKQCRLDELKFARVMPLIVHFSSAATIFAPELADARMVLSQTCMLITVYDDFFDCPESREEKENYIALIEKWDNHAEIGCSKNVEIVFYAVYNTYKQIGEKAALKQNRSIMDQLVEDLVSSAKAMMVEADWTATKYIPATMEEYMSNAEVSGAFASFVCPPLYFLGLKLSEEDVKSHEYTQLLKLTNVIGRLQNDSQTYRKEILAGKVNSVLLRALTDSGNTSPESIEAAKEIVNRDAESSMVESLPIPRPCKDRFWEMCKIVFYFY

>Pt0001s31550

DNREMKEDLYGTSVEFRLLRQHGYNVPQVFNSFKDEQGNFKNCLRDDVKGMLNLYEASYYLVNGESILEEARDFSEKHLKEYSKEQNEDHYLSLLVNHSLELPLHWRMQRMEARWFIDAYGRKRDLNPILLEFAGLDFNMVQAKYQEDIRHASRWWTSMDLGNKLFYTRDRLMENTLWAVGEVFEPQFGYYRKMATRITALITALDDAYDVYGTLEELEVFTDVIESWDVNALDQLPYYMKISFFALFQSINEIGYNILKEQGINVVPSLKKLWGDLCRAFLKEAKWYYAGYTPTLQEYLDNAWLSVSGQVILGHAFFLVTNQLTEEAVRCCMEYPDLIRHSSTIVRLADDLGTSSDEIARGDNPKSIQCYMHETGATEQEAREHVRYLIYETWKKLNVEILPFSKKFMGIPMDLARTAQCFY

>Pt0001s31570

DNREMKEDLYATSVEFRLLRQHGYNVPQDVFNSFKDEQGNFKNCLRDDVKGMLNLYEASYYLVNGESILEEARDFSEKHLKEYSKEQNEDHYLSLLVNHSLELPLHWRMQRMEARWFIDAYGRKRDLNPILLEFAGLDFNMVQAKYQEDIRHASRWWTSMDLGNKLFYTRDRLMENTLWAVGEVFEPQFGYYRKMATRITALITALDDAYDVYGTLEELEVFTDVIESWDINALDELPYYMKISFFALFQSINEIGYNILKEQGINVVPSLKKLWGDLCRAFLKEAKWYYAGYTPTLQEYLDNAWLSVSGQVIIGHAFFLVTNQLTDEAVRCCMEYPDLIRHSSTIVRLADDLGTSSDEIARGDNPKSIQCYMHETGATEQEAREHVRYLIHETWKKLNAEILPFSKKFMGIPMDLARTAQCFY

>Pt0001s31580

DNREMKEDLYGTSVEFRLLRQHGYNVPQDVFNSFKDEQGNFKNCLRDDVKGMLNLYEASYYLVNGESILEEARDFSEKHLKEYSKEQNEDHYLSLLVNHSLELPLHWRMQRMEARWFIDAYGRKRDLNPILLEFAGLDFNMVQAKYQEDIRHASRWWTSMDLGNKLFYTRDRLMENTLWTVGEVFEPQFGYYRKMATRVNALITTLDDAYDVYGTLEELEVFTDVIESWDINALDQLPYYMKISFFALFQSINEIGYNILKEQGINVVPSLKKLWGDLCRAFLKEAKWYYAGYTPTLQEYLDNAWLSISGQVILGHAFFLVTNQLTEEAVRCCMEYPDLIRYSSTIVRLADDLGTSSDEIARGDNPKSIQCYMHETGATEQEAREHVRYLIHETWKKLNAEILPFSKKFMGIPMDLARTAQSFY

>Pt0001s44080

DDDHHHNDLYAISLKFRLLRQQGYKISCDVFGKFKNSQGNFNDSLVNDTRAILSFYEATHLRVHGDEVLEEALVFTTSHLEFLATHSSSPLRAKINHALKQPIRKNIPRLEARNYFSVYQEDPSCSEVLLNFAKLDFNILQKQHQKELSDIAKWWKELDFAKKLPFARDRVIECYFWILGVYFEPEHFLARRMLTKVIAMTSVIDDIYDVYGKPEELELFTDAIERWEITAVDLLPEYMKVTYKALLDVYTEIEENMVNEERSYRVYYAKEAMKNQVRAYYHESKWFHQKHTPTMEEYMAVALVTSAYAMLAATSFVGMGVVTKDSFDWLFRGPKILKASEIICRLMDDIVSHKFEQKRGHVASSIECYMKQHGTTEQETVHEFRKQVTDAWKDVNEEFLAVPMPLLTRMLNLARVIDVVY

>Pt0002s05300

ARNSEVHDIDDTAMGFRVLRLNGHHVSADVFKHFEKGGEFFCFAGQSTAAVTGMFNLYRASQLLFPGEKILEKAKEFSFKFLREKQAANLLDKWLITKDLPGEVGFALEIPWHASLPRVESRFYIEQYGGEDDVWIGKTLYRMPYVNNNEYLQLARLDYNNCQALHRIEWANFQKWYEECNLRDFGISRKTLLYSYFLAAASVFEPERSNERLAWAKTTILLEMIHSYFDDDNSGAQRRTFVHEFSTGISRSGKTRKELVKMLLGTLNQLSFGALEVHGRDISHSLRHAWERWLISWELEGDRRRGEAELLVQTIHLTAGYKVSEELLVYHPQYEQLADLTNRICYQLGHYKNKHDNGSYSTIGSTDRITTPQIESDMQELMQLVIQKGIDPKIKQTFLQVAKSFY

>Pt0004s02970

SPNHLPLDVFGVALRFRLLRQEGYNVSQEVFNNFKNEEGNFHLIQENDVKGLMALYEASQLSMESEDILDEAGEFSAKLLNHHESEIVANTLKHPYHKSLARFMVKNFLNNIDIRNENIKVFSELAKIDCEIVRSIHQKEILQISNWWKDLGLAKELKFARDQPLKWHMWSMSVLIDPNLSEQRVELTKPISLVYIIDDIFDLYGTLNDLSIFTEAVNEWDLTAANQLPESMKISLKALFDITESISTKILEKHGWNPIESLQKSWKKLCNAFLEEAKWFASGKLPKPEEYLRNGIVSSGVHVVLVHMFFLLGQGINKETVDFVVGFPPIISFTATILRLWDDLGTAKDENQDGHDGSYLECYIREHNVTVERAREHVSHLICDAWKKLNQECLPFSPSFTKACLNVARMIPLMY

>Pt0004s02990

SPNHLPLDVCGVALRFRLLRQEGYNVSQEVFNNFKNEEGNFHLIQENDVKGLMALYEASQLSMESEDILDEAGEFSAKLLNHHESEIVANTLKHPYHKSLARFMVKNFLNNIDIRNENIKVFSELAKIDCEIVRSIHQKEILQISNWWKDLGLAKELKFARDQPLKWHMWSMSVLIDPNLSEQRVELTKPISLVYIIDDIFDLYGTLNDLSIFTEAVNEWDLTAANQLPESMKISLKALFDITESISTKILERHGWNPIESLQKSWKKLCNAFLEEAKWFASGKLPKPEEYLRNGIVSSGVHVVLVHMFFLLGQGINKETVDFVDGFPPIISFTATILRLWDDLGTAKDENQNGHDGSYLECYIREHNVTVERAREHVSQLICDAWKKLNQECLPFSRSFTNACLNVARMIPLMY

>Pt0004s03810

WTNSMATQLYKDSLAFCLLRMHGFRVSPGMFCWFLLEEVQDQIESNHEYFSSVILNVYRATDLMFPGDHELEEARSFSRKLLEKTTSMGNEDQHTVPFPSHSVIKHELRFPWMARLDHLEHRMWMEEEEHWNIVMMWTNIQRFDTCRLSCLHNDKLKQLAVKNYEFRQTTYKSELEELTRWSKSWGLSDMGFGREKTAYCYFAVAASTSLPQDSEIRMMVAKSAIVITVADDFYDMEGSLDDLEKITDAVQRWDATLSGHSKTIFDALDSLVNELARKYFRQHGTDITNSLRDIWSETFASWFTEAKWSKSGFIPAAEEYLETGMTSIASHTLVLPASCFLSPSIPDYKLNPVQYESITKLLMVIPRLLNDIQSYKKEQKEGKTNFVLLHLKENEADIEDSIAYAREILDKKKKELLEHMDDFSKPCRHLHLSCVKVFQMFF

>Pt0005s09830

GNNFDDDLFTVALRFRLLRQYGYNVSSDIFNEFKDGKGNFKDNLIDDVEGLLSLYEASFLGGHGEDTLDKALSFCKTHLESAVAHLVSPLADKVSHALKRPLLKGVPKHEQWHHILIYQQDEACTGAVLKLAKLDFNVVQKCYQDELRIISRWWIDLDFATKLPFARDRVIECFFWGLGAFLEPQFVLARRFITKVLIFLSILDDIYDVHGTIEELELFTEKIERWDTSMEDLPDYMKLFFEALIGFFDEIEQETGKEGRPYCVHYSREMLKNQARAYLIEARWFNQDCVPQLEEYRRGGVYTSCYPMAAVAWLCGMATGSKEVFEWMLKNPKIVVASSDIGRLMDDITSHEFEQERGHVASAVECCMKQYGVSKKEAYDMLNKMVESDWKDINEELLTVPRQVLILMLNLARIIDVVY

>Pt0005s23190

ARNSNVHDIDDTAMGFRILRLHGHQVSADVFKHFEKGGEFFCFAGQSTGAVTGMFNLYRASQVLFPGEKILEDAKEYSFEFLREKQAANLLDKWIITKDLPGEVGFALEIPWYASLPRVETRFFIEQYGGEDDVWIGKTLYRMSYINNSEYLQLAKLDYNNCQALHRIEWENFQKWYEECNLRDFGISRRTLIFSYFLAAASIFEPERSKERLAWATTTVLLDIVGSYFNHNNSSEQRRAFIHEFSYGINGREDLCLVCQELVKLLLGTLNQLSLGALVVHGRDISHSLRHAWEKWLLIWELEGDRRQGEAELLVQTINLTAGYLVSEELLAHHPQYEQLVDLTNRICYQLDHYKKNKVHYNGSYSTITSNTDRITTPQIESDMQELVQLVVGIDSNIKQTFLQVAKSFYY

>Pt0007s02810

GDLHTTSLHFRLLRQHAFSVSTDVFGKFRSRDGKFKDSIRTDVAGLLSLYEASYLGVPGEDHVLEEAKNFSSKHLKSLLETIKDEFLAQVKQSLEVPRHWKMPRIEARDFIDIYSSDNTRNLDLLELAKLDYNLVQSQHQRELKELARWWGALGFKEKLSFSRDRLMENYLWAMGMVFEPQFSKCRIGLTKFVCILTTIDDIYDVYGLPEELELFTKLVNRWDSMAIDDLPDYMKICYLALFNFVNEMAYDVMRDHGLFVLPYLVEEWANLCGSYLVEARWFSNKYSPTLSEYLENARTSIGSPAALAHACMLLGSVAQSSLMDCFKHGNDQLIYWSSLITRLSDDLGTYTAESERGDVTKSIQCYMIEKGASEKESKEHIKGLINQAWKELNKEKCSLPKPLVNMSLNMARTAQCIF

>Pt0007s02920

SYVEAEKSLRATALCFRLLRQHDIFHGFIDDQGNFMASLHNDIEGMLSLYEASHLACEGEEILNKANKQTSIYLRNHLGNSDSITARVSHALEVPLHHKMIMLEARWHIESYGKREDANPTLLQLSKFDFNMMQSVLQRDLQDMSRWWHDLGLKNKLRFSRDRLMECFFWTVGMAFEPEFNSCRKGLTKVTSFITTIDDVYDVYGALDELEAFTEAVERWDVSAVRNLPDYMKLCFLALFNTVNEMAYDHLKEQGEDAIPCLTKAWADLCKAFLQEAKWSYNNITPSFEEYLENAWRSVSGTVILIHAYFLMGENISKQALDYLVNYDELLRWPSIIFRLSNDLATSSAEIARGETANSISCYMYETGASEAEARKHIEKLIQKAWRNMNKCQIPFARSFVGTINLARIAQCTY

>Pt0007s07360

FLENNDHDLHTVALLFRVLRQYGCKVSSDVFKKFKDTNGEFKKTITSDVKGNLSLHEAAHLSVNGEQILDEALEFSRTNLESLATQSGPRLARHIKYALIRPIHKTVQRLEAREYISFYEEEDFRNETLLKFAKLDFNRVQLLHQQELSTLSSWWKDLNLVEELPYARDRIVEMYFWVNAMHFEPQYALARILSTKLGALITVIDDTYDAYSTYEELQHFTKAVIRCNIDAIDQLPDSMKALYRALLSYFDDVANEVSKNGKSFTAVNYVKEEMKEMIRTYIVEAQWCNDRFVPPLNEYVRNGKISIGFMATTTVFFVVETARIKELEWLTSKAKISEAGCLFLRLMNDIVTHEFEQKREHCASAIECYMKEYGVSMNEAVKELQKTCADAWKDINEDCLAISMNLLNVCVNNARATDVVY

>Pt0007s07410

LLENNDHDLHTVALLFRVLRQNGCKVSSDVFKKFKDTNGEFKKTITSDVKGNLSLHEAAHLSVNGEQILDEALEFSRTNLESLATQSGPRLARHIKYALIRPIHKTVQRLEAREYISFYEEEDFRNETLLKFAKLDFNRVQLLHQQELSTLSSWWKDLNLVEELPYARDRIVEMYFWVNAMHFEPQYALARILSTKLGALITVIDDTYDAYSTYEELQHFTKAVIRCNIDAIDQLPDSMKALYRALLSYFDDVANEVSKNGKSFTAVKYVKEEMKEMIRTYIVEAQWCNDRFVPPLNEYVRNGKISIGFMATTTVFFVVETARIKELEWLTSKAKISEAGCLFLRLMNDIVTHEFEQKREHCASAIECYMKEYGVSMNEAVKELQKTCADAWKDINEDCLAISMNLLKVCVNNARATDVVY

>Pt0008s08190

GEEEIFSDNATCALAFRILRLNGYDVSLDTLNQFSEDHFSNSLGGYLKDSGAALELYFPPLQVHDALNFSDHANLQRLAIRRRIKHYATDDTRILKTSYRCSTIGNQDFLKLAVEDFNICQSIQREEFKHIERWVVERRLDKLKFARQKEAYCYFSAAATLFAPELSDARMSWAKNGVLTTVVDDFFDVGGSEEELVNLIELIERWDVNGSADCSEEVEIIYSAIHSTISEIGDKSFGWQGRDVKSQVIKIWLDLLKSMLTEAQWSSNKSVPTLDEYMTTAHVSFALGPIVLPALYFVGPKLSEEVAGHPELLNLYKVTSTCGRLLNDWRSFKRESEEGKLNAVSLYMIHSGSTEEEAIEHFKGLIDSQRRQLLQLVLIIPRPCKDLFWNMIKLLHTFY

>Pt0008s08220

GEEEIFSDNATCALAFRILRLNGYDVSLEDHFSNSLGGYLKDSGAALELYRALQLSYPDESLLEKQNSRTSYFLKQGLSNVSLCGDRLRKNIIGEVHDALNFPDHANLQRLAIRRRIKHYATDDTRILKTSYRCSTIGNQDFLKLAVEDFNICQSIQREEFKHIERWVVERRLDKLKFARQKEAYCYFSAAATLFAPELSDARMSWAKNGVLTTVVDDFFDVGGSEEELVNLIELIERWDVNGSADCSEEVEIIYSAIHSTISEIGDKSFGWQGRDVKSHVIKIWLDLLKSMLTEAQWSSNKSVPTLDEYMTTAHVSFALGPIVLPALYFVGPKLSEEVAGHPELLNLYKVMSTCGRLLNDWRSFKRESEEGKLNAISLYMIHSGSTEEETIEHFKGLIDSQRRQLLQLVLIIPRPCKDLFWNMIKLLHTFY

>Pt0011s03440

KNLKVEENLYVTALRFKLLRLHGYEVSQGVFNGFFDGTFDKSKCTDVRGLIELFEASHLAYEGEATLDDAKAFSTRILTGINCSAIESDLAKHVVHVLELPSHWRVMWFDVKWHINAYENDKQTNRHLLALAKVNFNMVQATLQKDLRDVSRWWRNLGIIENLSFTRDRLVESFLCTVGLVFEPKYSSFRKWLTKVIIMILIIDDVYDVYGSLHELQQFTKAVSRWDTGEVQELPECMKICFQTLYDITNEMALEMQREKDGSQALPHLKKVWADFCKAMFMEAKWFNEGYTPSLQEYLSNAWVSSSGTVISVHSFFSVMTELETGEISNFLEKNQDLVYNISLIIRLCNDLGTSVAEQERGDAASSVVCYMREVNVSEEVARNHINNIVKKTWKKINGHCFAKSPTLQLLNTNMARVVHNLY

>Pt0011s14600

GAYVHDHDLQMVALRFRLLRQQGRYVSCDVFKKFKDTEGNYKVCLANDIQGMLSLYEAIHLRVHREDILEDALTFATTHLKSITTDMCPPPLLVKLRHALDQPIHKDLPWLGAKHYISIYEQEASHSEVLLKFAKLNFNFLQNMHQKELADMTMWWKKVDLSKKLPFARDRLVECYFWILGVCFEPQYSFARIIMTKVIAMTSVMDDVYDVYGTMEELVLFTDAIERWDISNIDHLPEYMKFFYKQLSDVYKEIETELAAQGRSYRVDYAKEAMKKQVQAYFVEARWLHENYMPTMDEYMRISLISSGYPLLTCISFVGMGDIVTKDAFEWLNKDPKIVKAASLIARLMDDIVSHKFEQERGHVASAVECYMNQHEVSEEQAYDELRRQVVEAWKDINEELLHVPIPLLTRVLNLARVMDVMY

>Pt0015s05270

DMEDDDLYNTALGFRLLRQHGYNVSCDIFNKFKDDKGYFKPSNDVRGILGLYEAAHLAVHGEDILDEALAFTTIHLKSMATSPNCPLTAKVSHALKQPIRRGVPRLESRRYISFYQDEPSCNKTLLRLAKLNFNVVQELHKEELSEITRWWKGLDFARRLPFARDRVVECFFWIVGAYFEPQYSLARKILTKVIAMTSIIDDIYDVYGTLEELELFTEAIDRWDTKSMDQLPDYMKICYEALLNVYSEIEEKVAKEGWSYRVHFGKQAMKVLVHAYFDEAKWFHENHIPTMEEYMQVALVTSGYSLLATVSFIGMGDMVTEQAFDWVFNRPKIVRASETISRLVDDVRSHKFEQERGHAASGVECYIRQYGLSEQEVYKEFHMQVVNAWKDINEECLAVPMPLLERILNLTRVIDVIY

>Pt0015s09710

DMEDEDLYNTALGFRLLRQHGYNVSCDIFNKFKDDKGYFKQSNDVRGILGLYEAAHLAVHGEDILDEALAFTTIHLKSMETSPNCPLTAKVSHALKQPIQRGVPRLESRRYISIYQDEPSCNKTLLRLAKLNFNLVQELHKEELAEITRWWKGLDFARRLPFARDRVVECFFWIVGVYFEPQYSLARKILTKVIAMTSIIDDIYDVYGTLEELELFTEAIDRWDTKSMDQLPDYMKICYEALLNVFSEIEEKVAKEGWSYRVHYGKDAMKVLVHAYFNEAKWFHENHIPTMEEYMQVALVTSGYSMLTTVSFIGMGDMVTKQAFDWVFNHPKIIRASETIGRLMDDVKSHKFEQERGHAASGVECYIRQYGLSEQEVYKEFHMQVVNAWKDINEECLAAPMPLLERILNLSRVIDVIY

>Pt0017s06920

FDAVTKTSLHATALSFRLLRQHGFEVSQEAFGGFKDQNGNFMENLKEDIKAILSLYEASFLALEGENILDEAKVFAISHLKELSEEKIGKDLAEQVNHALELPLHRRTQRLEAVLSIEAYRKKEDADQVLLELAILDYNMIQSVYQRDLRETSRWWRRVGLATKLHFARDRLIESFYWAVGVAFEPQYSDCRNSVAKMFSFVTIIDDIYDVYGTLDELELFTNAVERWDVNAIDDLPDYMKLCFLALYNTINEIAYDNLKEKGENILPYLTKAWADLCNAFLQEAKWLYNKSTPTFDDYFGNAWKSSSGPLQLVFAYFAVVQNIKKEEIENLKKYHDIISRPSHIFRLCNDLASASAEIARGETANSVSCYMRTKGISEELATESVMNLIDETWKKMNKEKLLFAKPFVETAINLARQSHCTY

>Pt0019s01270

ENTETVHDLYATALEFRLLRQRGYHVPQEVFNHFKDEQGNFRACIHDDLKGMLNLYEASYFLVDGENILEDARDFTTKNLENYVKKCNTTEYLSELVSHALELPLAWRMLRLEAHWFINLYETKTDMEPVLLELAKLDFNMVQAVYQEDLKDSSRWWKMTGRGEKLDFARDRLVVYLLWSVGIIFEPQFGNIRRMITKLNSLITTIDDVYDVYGTLDELELFTDAVVRWDLNFMDHLPDYMKLCFFALFNSINEIAYDILRDQGVDNLPYLKKTWADLCKSHLLEAKWYYSGYTPTLQEYLDNAWISVGAPLAIVHAYFYASNPTTKEASHFMEEYPDIIRWSSIILRLADDLGTSSDEMKRGDVSKSIQCYMYESEASEEEARDHIRKLISNAWKKINAYQFHISQTIIGVVVNLARAAQCIY

>Pt0019s01290

TVHDLYATALEFRLLRQRGYHVPQEVFNHFKDEQGNFRACIHDDLKGMINLYEPSYFLVEGENILEDARDFTTKNLENYVKKCNTTEYLSELVSHALELPLAWRMLRLEAHWFINLYETKTDMEPVLLELAKLDFNMVQAVYQEDLKDSSRWWKMTGLGEKLDFVRDRPMVWDVNFMDHLPDYMKLCFFVLFNLINEIAYDILRDQGVDSLPFLKKAWIYTNAWISIATSLAIVHTYFYASNPTTEEASHFMEEYPDIIRWSSIILRLADDLGTSSDEIKRGDVSKSIQCYMHETQASEEEARDHIKKLISNAWKKLNASQFHISQTIIGVAVNLPRTAQCIY

>Pt0019s01320

LLEKHDFDLYTVSLLFRVLRQHGFKMPCVVFDKFKDTNGEFKKTIINDVKGILSLYEASLLSVHGEQVLDEALVFTKANLESLAMQSNPRLADHIRNALIRPFHKGVPRIEARKYISFYEEEESRIDTLLKFAKIDFNRVQLIHRQELSILSRWWNDLNFSEEFPYARDRIVEIYFWANGIHFEPQYAFSRMMVTKYTKIVSLVDDTYDAYASFEEIQHFTNAIERCSMNAIDQLPDYMKVLYRALLNLFNETENDMGKQGRYYASYYVKEAFKELVRGYHAEAEWADKCHVPTFDEYVRNGLATSAYGVIMAASFLGMEEVAGGEEYEWLKSNPKIIKAGKMIGRLMNDIVGHEDEQKRGDCASGVECYMKQYDVSEKKAIEEIQKMDVNAWKDINEDCMNAPMLLLQHFVNLIRVTDVIY

>Pt0019s01340

ENTKTVHDLYATALEFRLLRQHGYKVPQEVFNHFKDEQGNFRAWIHDDLKGMLFLYEASYFLVEGESILEDARDFTTKNLEKYFKKCNPSEYLSKMVSHALELPLAWRMLRLESNWFINVYETKTDMEPVLLELAKLDFNMVQALHQEDLKHSSRWWKRTGLGEKLDFARDRLVENFLWTVGVIFEPQFGNCRRMLTKVNSLITTIDDVYDVYGTLDELELFTDAVVRWDLNFMDRLLDYMKLCFLAFYNSVNEMTYDILKYQGVDILPYLKKAWADLCKSYLLEAKWYFSGYTPTLQEYMENAWISISAPVILVHAYFYVSNPTTEEASQFMEEYPDIIRWSSMILRLADDLGTSTDELKRGDISKSIQCYMHEAGVSEEKAREHIRNLIENTWKKINDYQFRISQTFIGIAINLARMAQCMY

>Pt0019s03350

LLEKHDFDLYTLSLLFRVLRQHGFKMPCVVFDKFKDNNGEFKKTIINDVKGILSLYEASFLSVHGEQVLDEALVLTKTNLESLAMQSSPRLAHHIRYALIRPFHKGVPRIEARKYISFYEEEESRNDTLLKFAKIDFNRVQLLHRQELSILSRWWNDLNFSEEFPYARDRIVEIYFWANAIHFEPQYAFSRMMVTKYTKIVSLLDDTYDAYASFEEIQHFSDAIERCCMDAIDQLPEYLKVLYRALLNLFNETESDMGKQGRSYASYYVKEAFKELTRGYQVEAQWADVGHVPPFDEYVPNGLETTGYGVIMAASFVEMDEVAGEEEYKWLKSNPQIMKAAKMIGRLMNDIVGHEDEQKRGDCASGVECYMKQYDVSDKKAIEEIQKMVANGWKDINEDCMNAPMLLLQHIVNLARVTDVVY

>Pt0019s03980

ENTETVHDLYATALEFRLSRQRGYHVPQEVFNHFKDEQGNFRAWIHDDLKGMLNLYEASYFLVEGENILEDARDFTTKNLENYVKKCNTTEYLSELASHALELPLAWRMLRLEAHWFINLYETKTDMEPVLLELAKLDFNMVQAVLQEDLKDSSRWWKMTGLGEKLDFARDRLMENFLWSAGIIFEPQFGNCRRMLTKLNSLVTTVDDIYDVHGTLDELELFTDAIVRWDLNFMDVLPDYMKLCFFALFNSINEVAYDILRDQGVDSLPYLKKAWADLCKSYLLEAKWYYSGYTPTLQEYLDNAWISIGVPLAIVHAYFYAPNPTAEEASHFTEEYPDIIRWSSMIVRLADDLGTSSDEIKRGDVSKSIQCYMHETEASEEEARDHIKKLISYAWKKLNASQFHISQTIIGVAVNLPRTAQCIY

>Pt0019s03990

LLEKHDFDLYTVSLLFRVLRQHGFKMPCVVFDKFKDTNGEFKKTIINDVKGILSLYEASFLSVHGEQVLDEALVFTKANLESLAMQSNPRLADHIRNALIRPFHKGVPRIEARKYISFYEEDESRIDTLLKFAKIDFNRVQLIHRQELSILSRWWNDLNFSEEFPYARDRIVEIYFWANGIHFEPQYASSRMMVTKYTKIVSLMFRKGNGIFCLNFRCSMNAIDHLPDYMKVLYRALLNLFNETENDMGKQGRSYASYYVKEAFKELVRGYHAEAEWADKCHVPTFDEYVRNGLATSAYGVIMAASFLGMEEVAGGEEYEWLKSNPKIIKAGKMIGRLMNDIVGHEDEQKRGDCASGVECYMKQYDVSEKKAIEEIQKMDVNAWKDINEDCMNAPMLLLQHFVNLIRVTDVVY

>Pt0019s06190

LLEKHDFDLYTLSLLFRVLRQHGFKMPCVVFDKFKDTNGEFKKTIINDVKGILSLYEASFLSVHGEQILDDALVFTKANLESSAMQSSPRLADHIRNALIRPFHKGIPRIEARKYISFYEEDESHMDTLLKFAKIDFNRVQLLHRQELSILSRWWNDLNFAEEFPYARDRIVEIYFWVNAIHFEPQYAFSRMVVTKYTKFVSLLDDTYDAYASFEEIQHFTNAIERCCMDAIDQLPEYLKVLYRALLNLFSETESDMGKQGRSYASYYLKEAFKELARAYQVEAQWADEGHVPTFDEYVRNGLATSSYGVTTAASFVEMDEVAGREEYEWLNSNPKIIKAGKMIGRLMNDIAGHEDEQKRGDCASGVECYMKQYDASEKKAIEEIQNMVANGWKDINEDCMNAPMLLLQHIVNLVRVTDVMY

>Pt0019s06220

LLEKHDFDLYTLSLLFRVLRQHGFKMPCVVFDKFKDTNGEFKKTIINDVKGILSLYEASFLSVHGEQILDDALVFTKANLESSAMQSSPRLADHIRNALIRPFHKGVPRIEARKYISFYEEEESRNDTLLKFAKIDFNRVQLIHRQELSILSRWWNDLNFAEEFPYARDRIVEIYFWANGVHFEPQYAFSRMVVTKYTKIVSLLDDTCDAYASFEEIQHFTNAIERCCMDAIDQLPEYLKVLYRALLNLFSETESDMGKQGRSYALYYVKEAFKELARAYRVEAQRADEGHVPTFDEYVRNGLTTSAYGVITAVSFVGMDEVAGQEEYKWLKSNPKIMKAGKMICRLVNDIVGHEDEQKRGDCASGVECFMKQYDVSEKKAIEEIQKMVANGWKDINEDCMNAPMLLLQHIVNLVRVTEVTY

>Pt0092s00200

DNREMKQDLYATSVEFRLLRQHGYNVPQDVFNSFKDEQGNFKNCLRDDVKGMLNLYEASYYLVNGESILEEARDFSEKHLKEYSKEQNEDHYLSLLVNHSLELPLHWRMQRMEARWFIDAYGRKRDLNPILLEFAGLDFNMVQAKYQEDIRHASRWWTSMDLGNKLFYTRDRLMENTLWTVGEEFEPQFGYYRKMATRVNALITTLDDAYDVYGTLEELEVFTDVIESWDINALDQLPYYMKISFFALFQSINEIGYNILKEQGINVVPSMKKLWGDLCRAFLKEAKWYYAGYTPTLQEYLDNAWLSISGQVILGHAFFLVTNQLTEEAVRCCMEYPDLIRHSSTIVRLADDLGTSSDEIARGDNPKSIQCYMHETGATEQEAREHVRYLIHETWKKLNAEILPFSKKFMGIPMDLARTAQSFY

>Pt0121s00250

FDLSTVSLLFRVFRQHGFKMPCVVFDKFKDTNGEFKKTIINDVKGILSLYEASFLSVHGEQVLDEALVFTKANLESLAMQSNPRIADHIRNALIRPFHKGVPRIEARKYISFYEEDESRNATLLKFAKIDFNRVQLIHRQELSILSRWWNDLNFSEEFPYARDRIVEIYFWANGIHFEPQYAFSRMMVTKYTKIVSLVDDTYDAYASSEEIQHFTNAIERCSMNAIDQLPDYMKFKELVRGYHAEEEWADKCHVPTFDEYVRNGLATSAYGVIMAASFLGMEEVAGGEEYEWLKSNPKIIKAGKMIGRLMNDIVGHEDEQKRGDCASGAECYMKQYDVSEKKAIEEIQKMDVNAWKDINEDCMNAPMLLLQHFVNLIRVTDVIY
